# Supplementary material for: A ribose-functionalized NAD+ with unexpected high activity and selectivity for protein poly-ADP-ribosylation
Source: Nat Commun. 2019 Sep 13;10:4196. doi: 10.1038/s41467-019-12215-4 (PMC6744458; doi:10.1038/s41467-019-12215-4)
Supplement: Supplementary file 1 — Supplementary Information [file 41467_2019_12215_MOESM1_ESM.pdf]

Supplementary Information

# A Ribose-Functionalized NAD$^+$ with Unexpected High Activity and Selectivity for Protein Poly-ADP-Ribosylation

Zhang et al.

**Table of Contents**


Supplementary Figures ........................................................................................................ 3

Supplementary Tables……………………………..………………………………………..28

Supplementary Methods ..................................................................................................... 29

Supplementary References ............................................................................................... 104

## Supplementary Figures

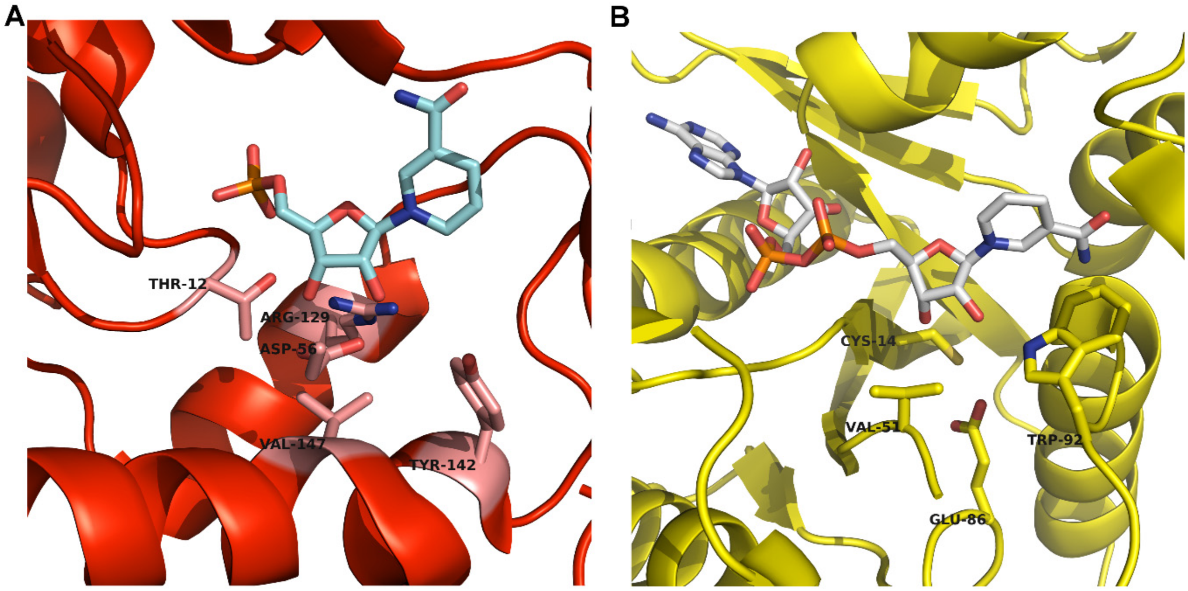


**Supplementary Figure 1**. X-ray structures of NAD$^+$-biosynthesis enzymes. (A) Active site of human NRK1 in complex with NMN (PDB ID: 2QG6). (B) Active site of human NMNAT1 in complex with NAD$^+$ (PDB ID: 1KQN). Residues adjacent to 2′- and 3′-OH groups of NR moieties are shown as sticks.

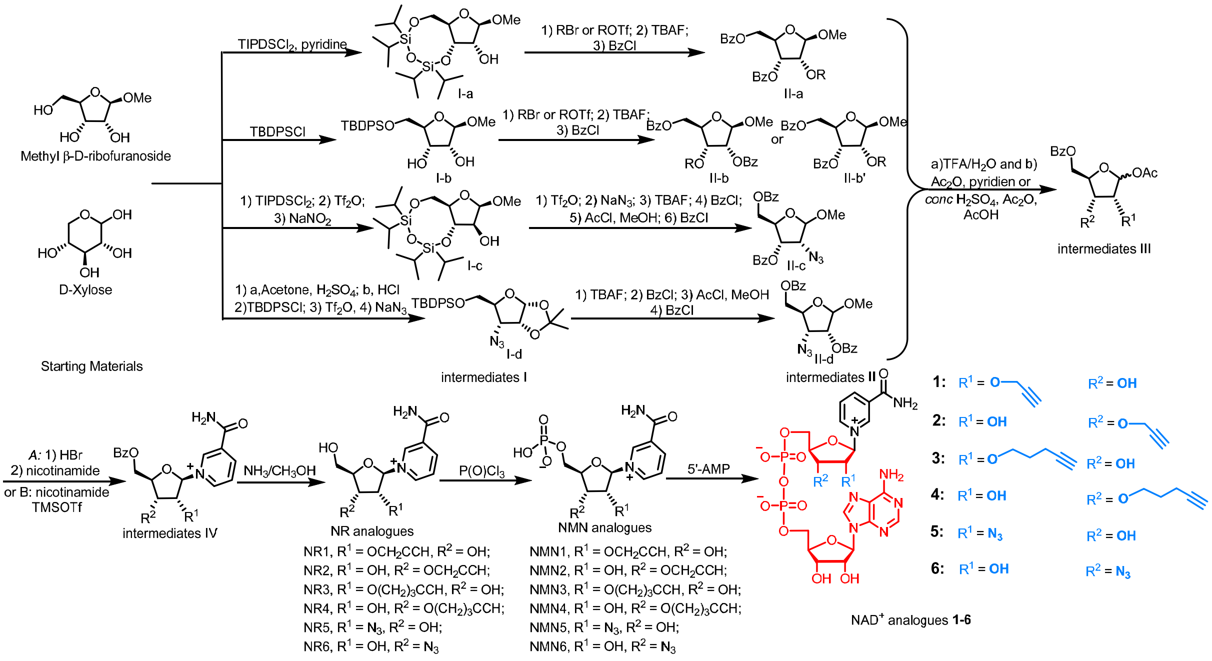


**Supplementary Figure 2**. Chemical synthesis of NAD$^+$ analogues **1-6**.

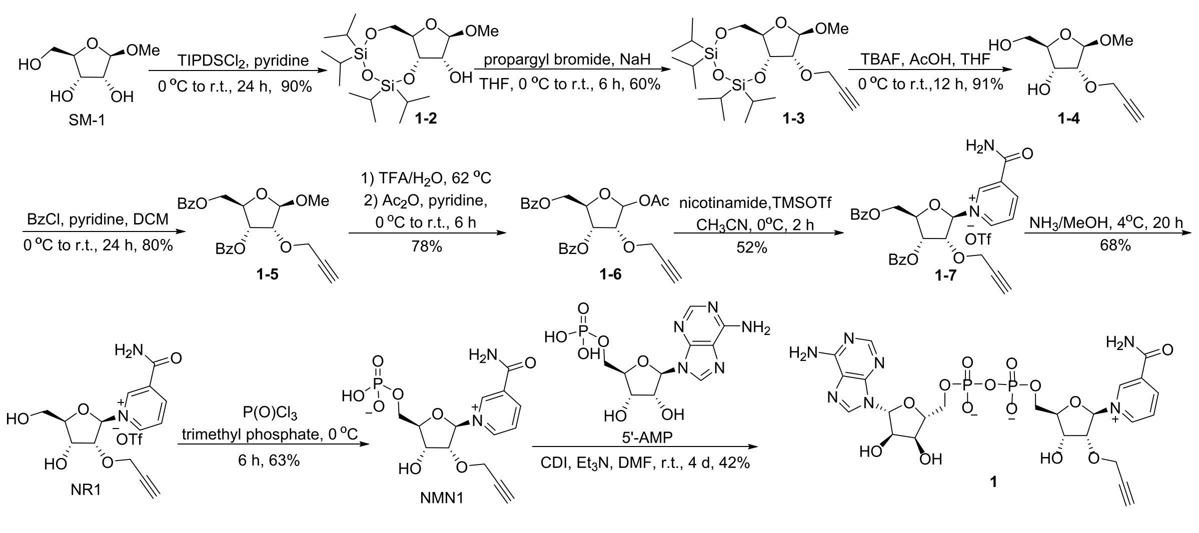


**Supplementary Figure 3**. Synthesis of NAD$^+$ analogue **1**.

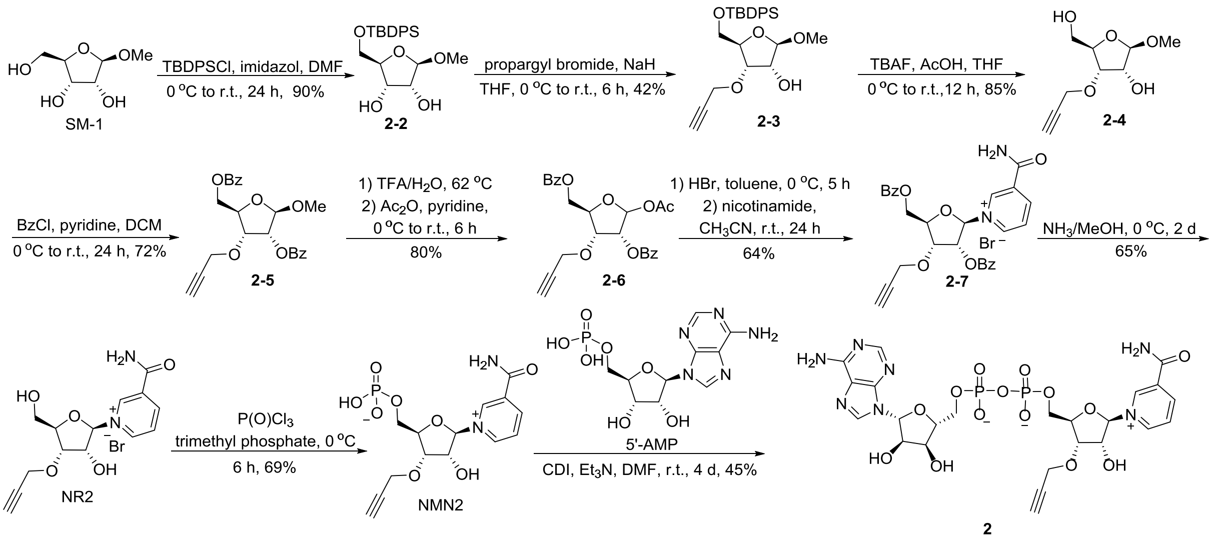


**Supplementary Figure 4**. Synthesis of NAD$^+$ analogue **2**.

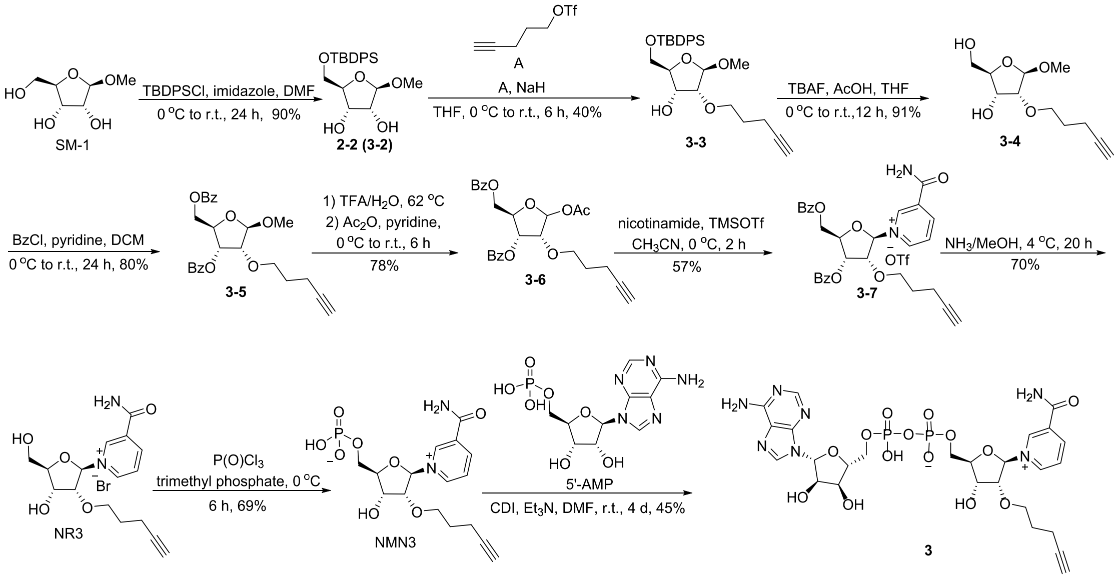


**Supplementary Figure 5**. Synthesis of NAD$^+$ analogue **3**.

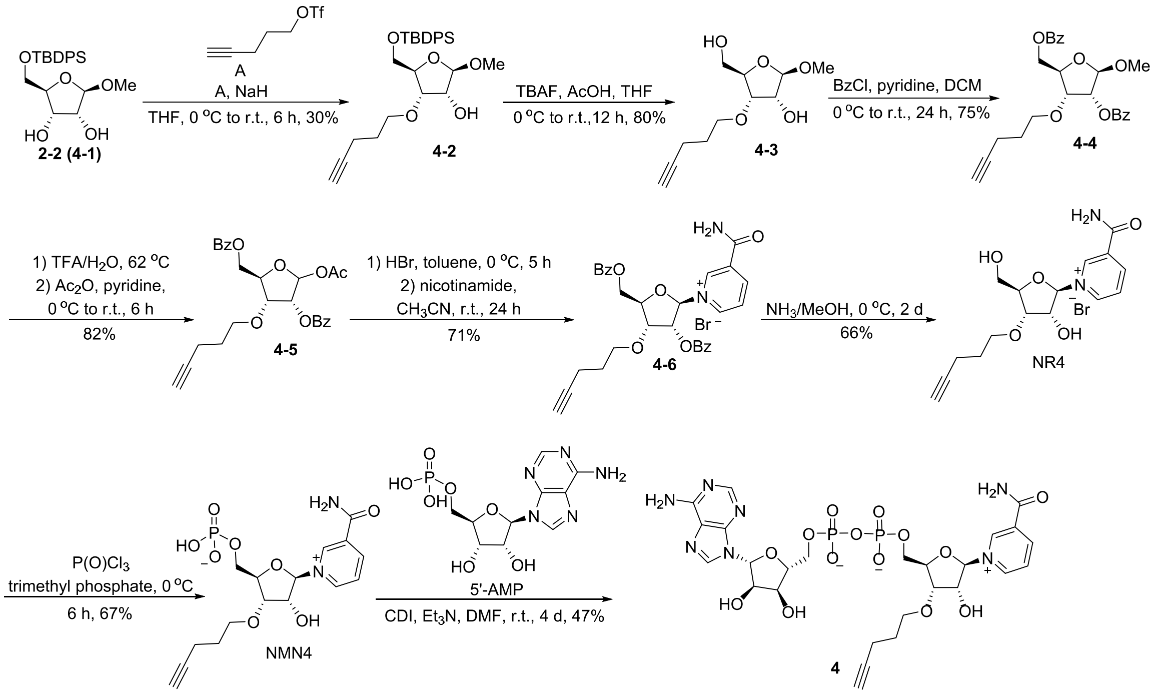


**Supplementary Figure 6**. Synthesis of NAD$^+$ analogue **4**.

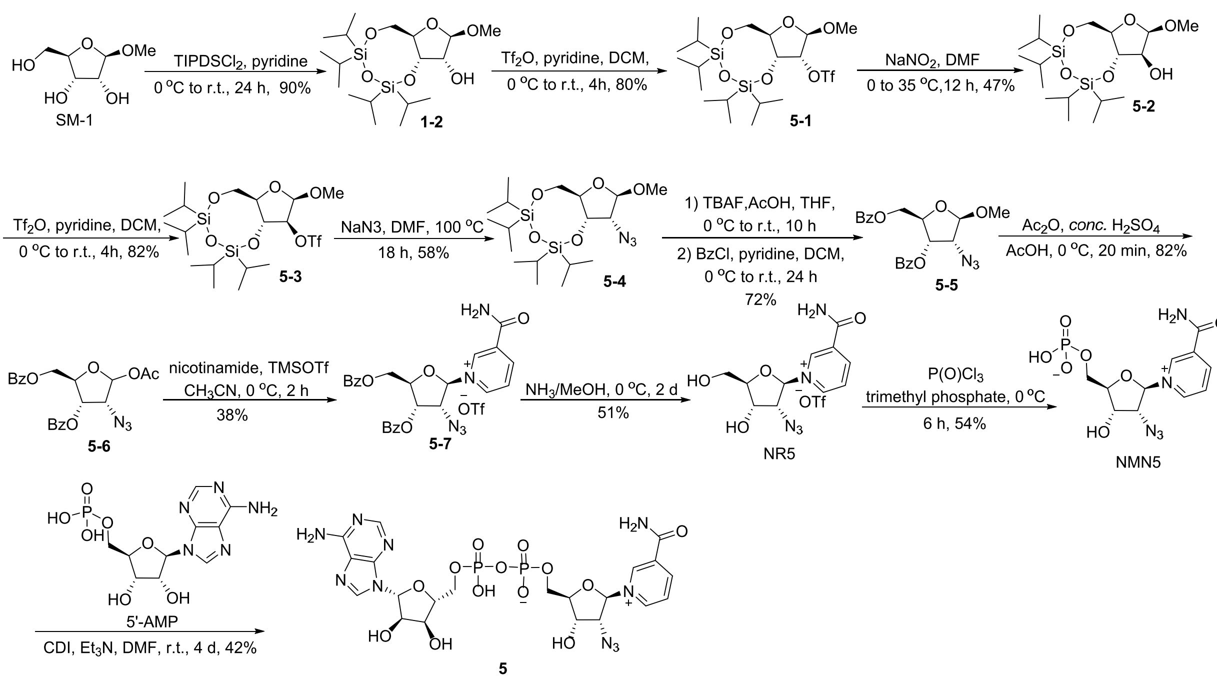


**Supplementary Figure 7**. Synthesis of NAD$^+$ analogue **5**.

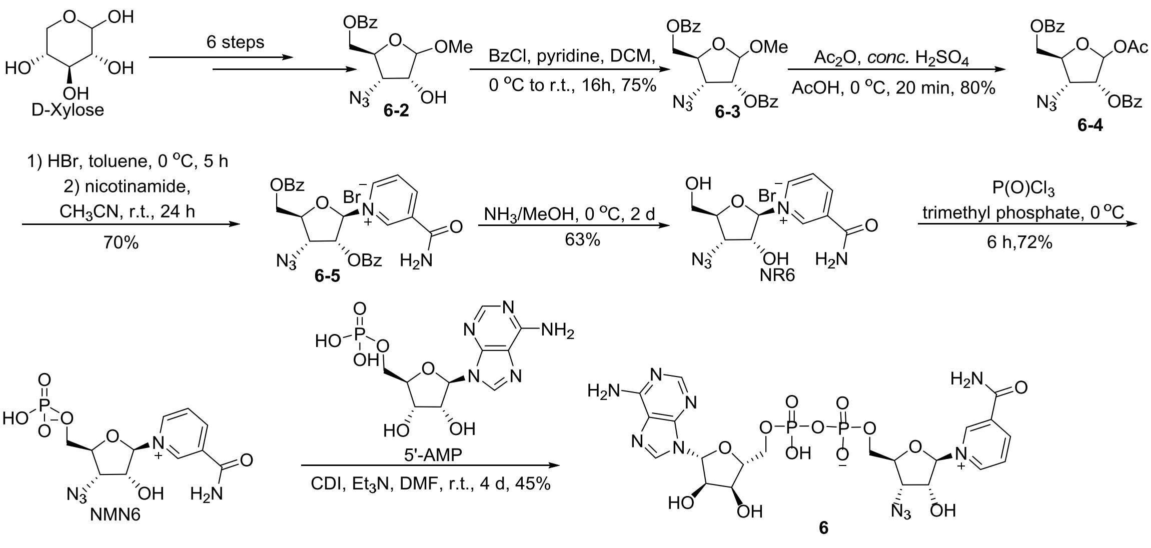


**Supplementary Figure 8**. Synthesis of NAD$^+$ analogue **6**.

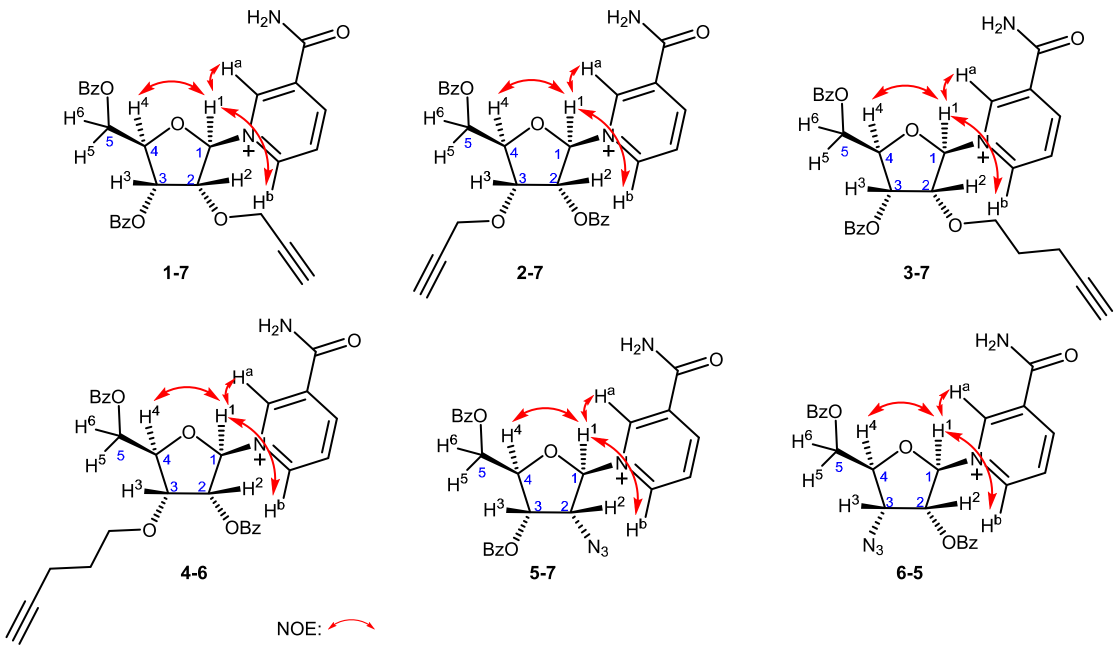


**Supplementary Figure 9.** Observed $H^1$ correlations in NOESY experiments. Since the configurations at C4 of **1-7**, **2-7**, **3-7**, **4-6**, **5-7**, and **6-5** are the same as in corresponding starting materials, the configurations at C1 of these compounds could be determined using NOESY spectroscopy. As shown in the Supplementary Figures 9, 11, 13, 15, 17, 19 and 21, the proton $H^1$ of each compound has no correlation with the $H^3$, $H^5$, and $H^6$, but correlates with the $H^4$ proton, supporting the *cis* relationship between the $H^1$ and $H^4$.

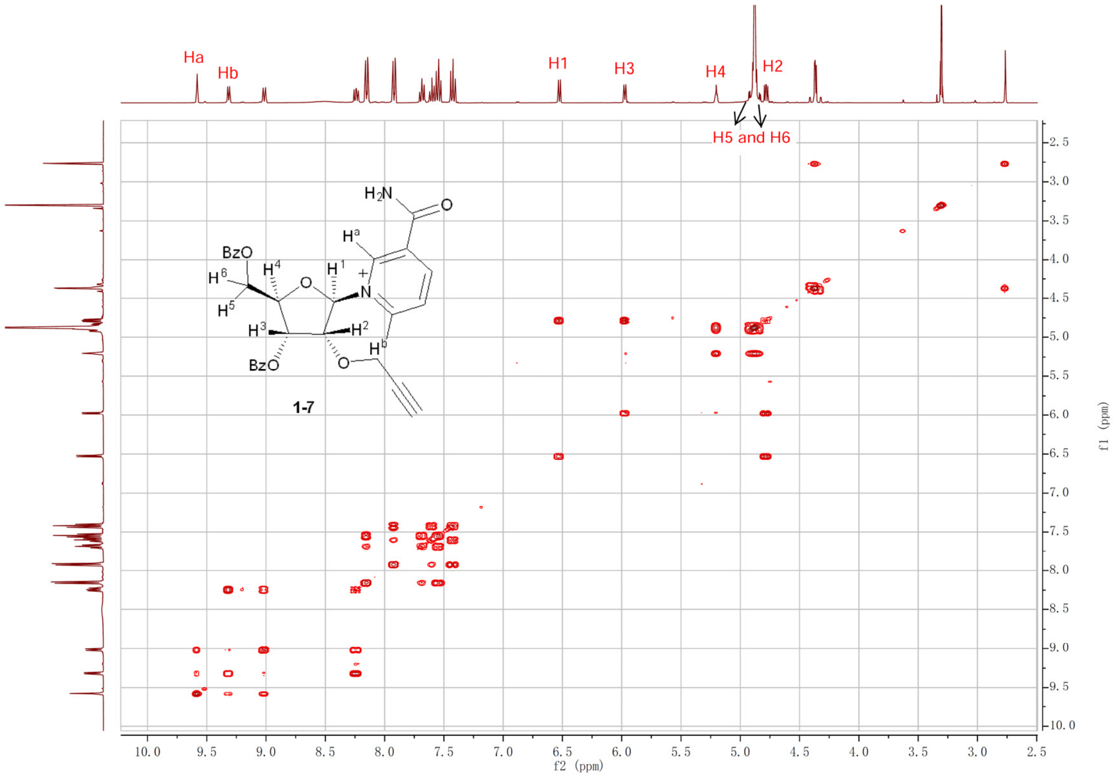


**Supplementary Figure 10.** g-COSY spectra of **1-7**. The solvent was $CD_3OD$.

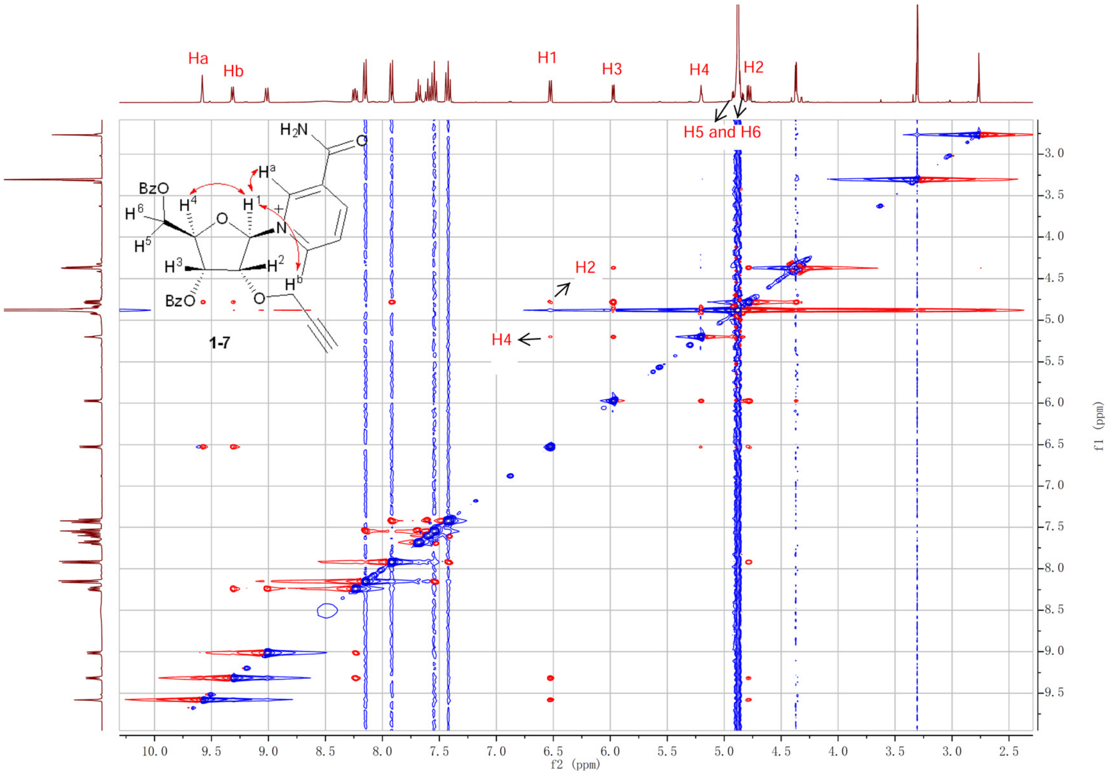


**Supplementary Figure 11.** NOESY spectra of **1-7**. The solvent was $CD_3OD$.

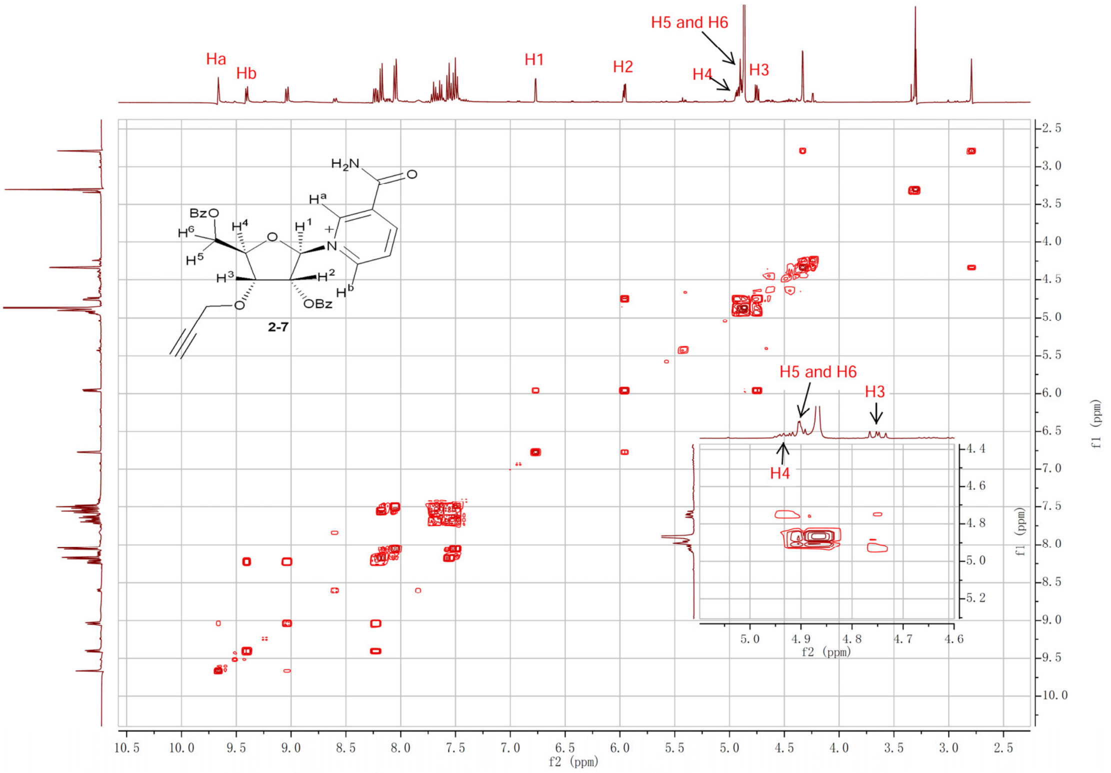


**Supplementary Figure 12.** g-COSY spectra of **2-7**. The solvent was $CD_3OD$.

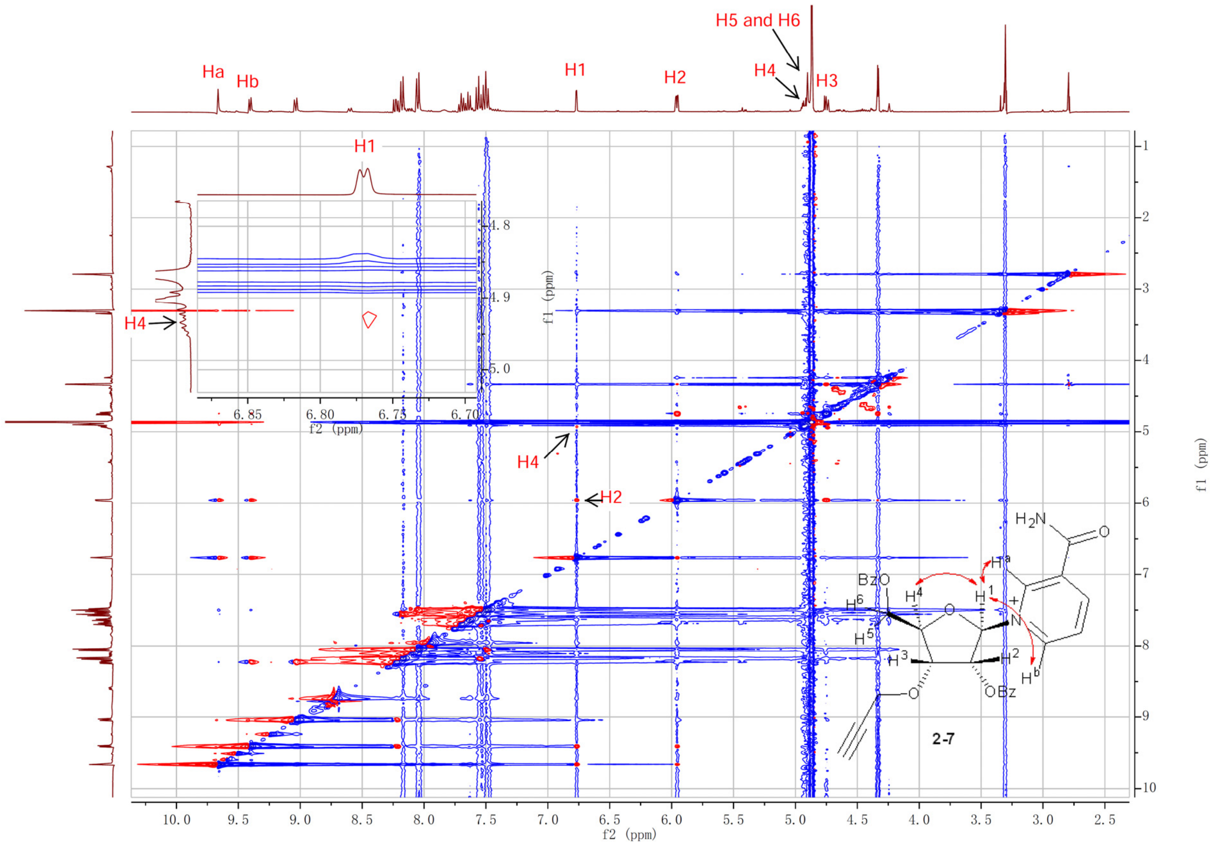


**Supplementary Figure 13.** NOESY spectra of **2-7**. The solvent was $CD_3OD$.

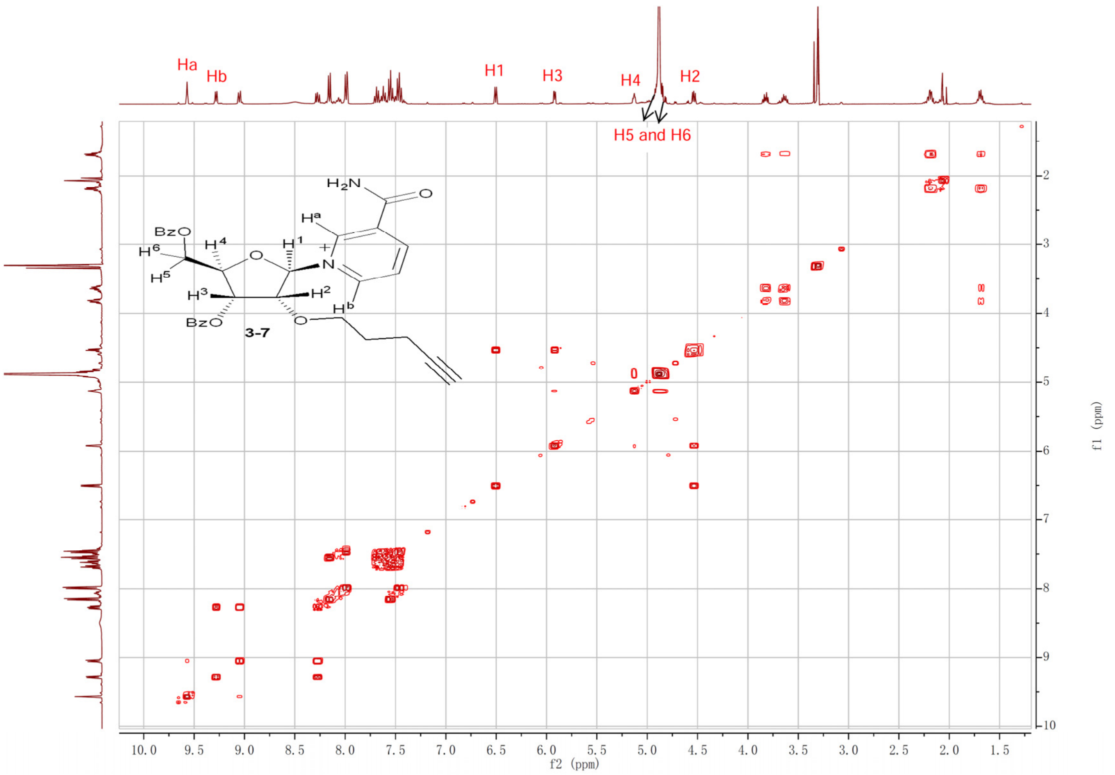


**Supplementary Figure 14.** g-COSY spectra of **3-7**. The solvent was $CD_3OD$.

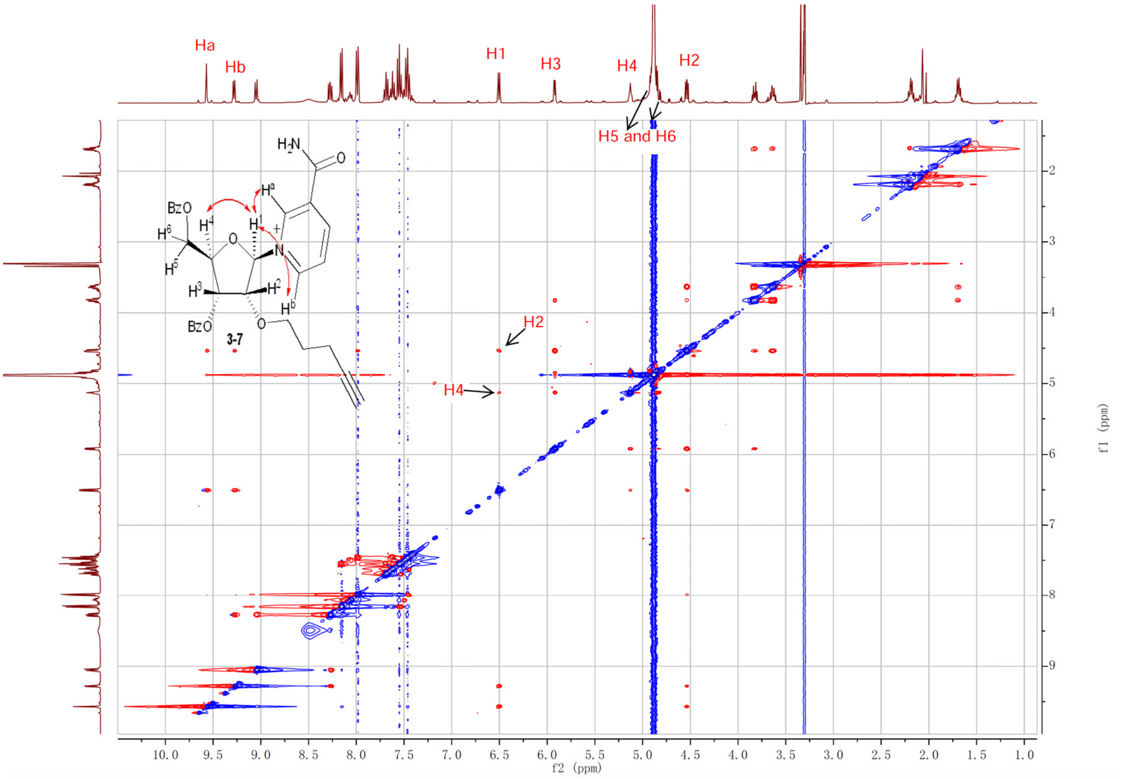


**Supplementary Figure 15.** NOESY spectra of **3-7**. The solvent was $CD_3OD$.

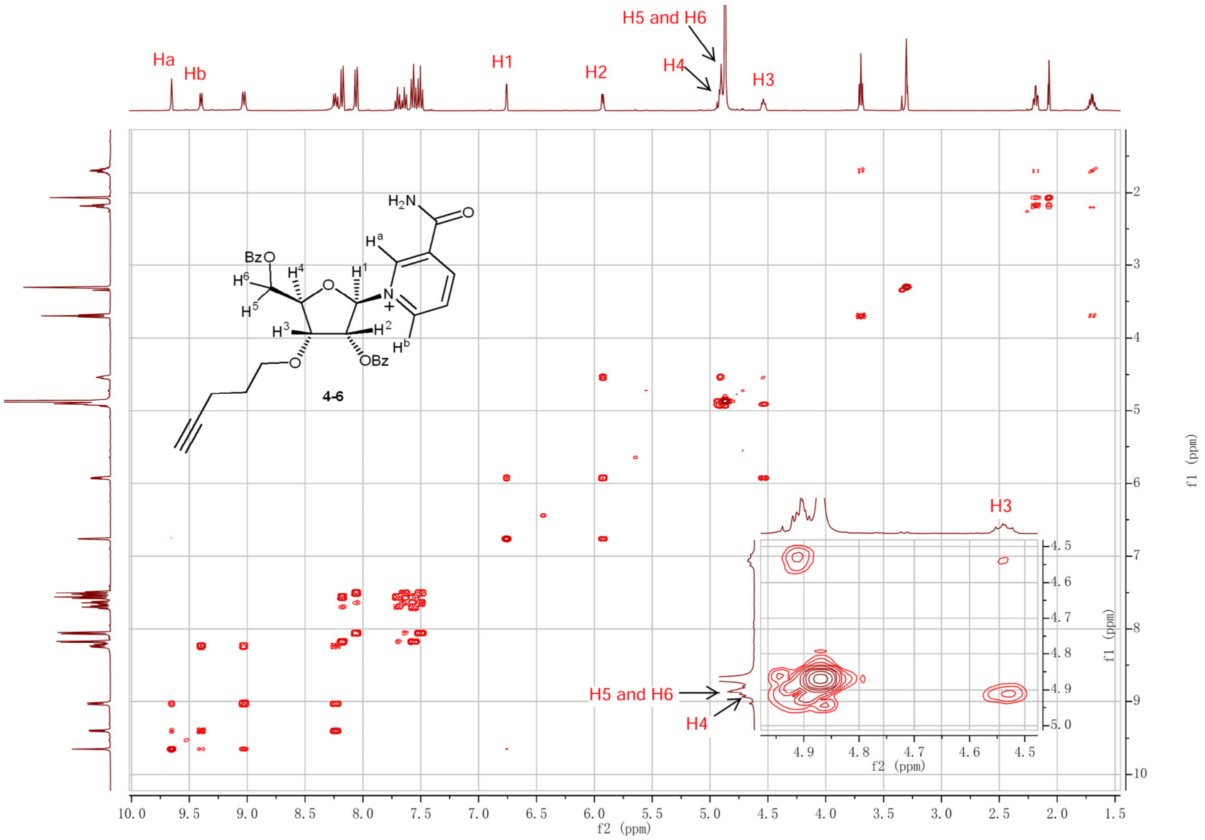


**Supplementary Figure 16.** g-COSY spectra of **4-6**. The solvent was CD3OD.

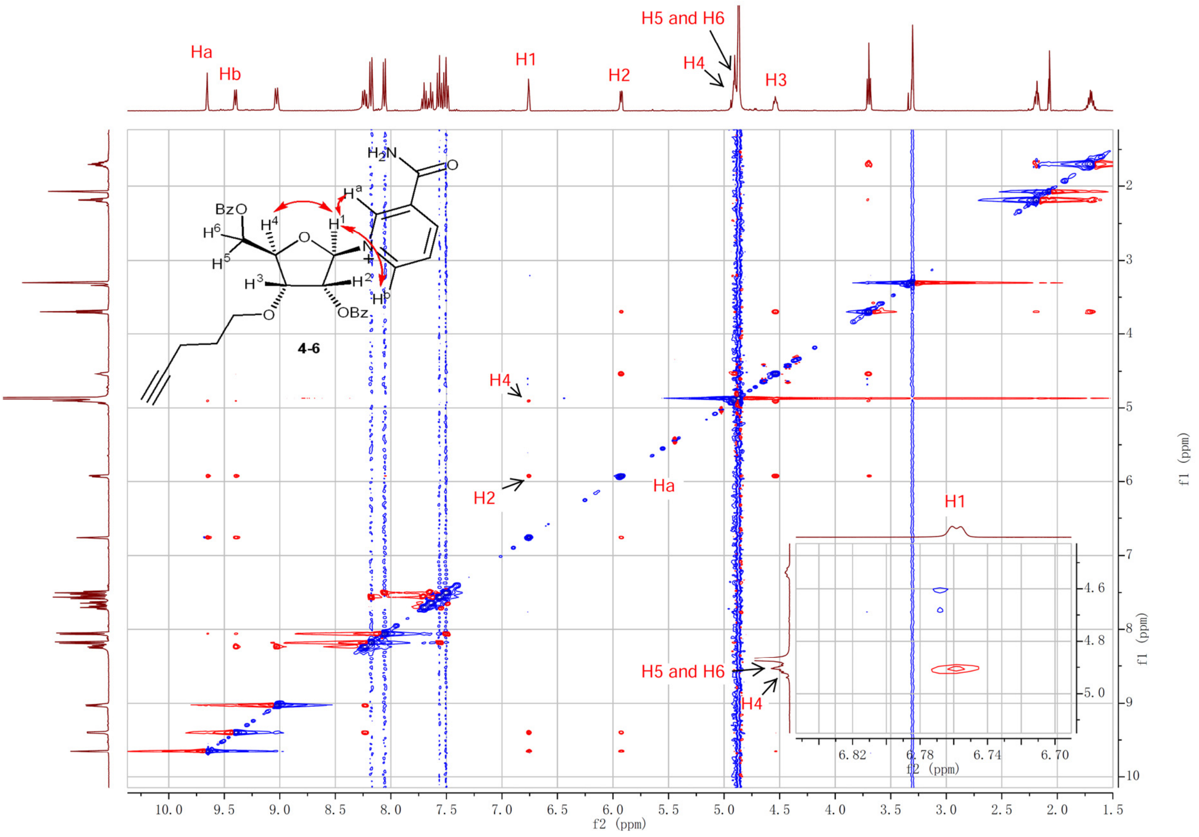


**Supplementary Figure 17.** NOESY spectra of **4-6**. The solvent was CD3OD.

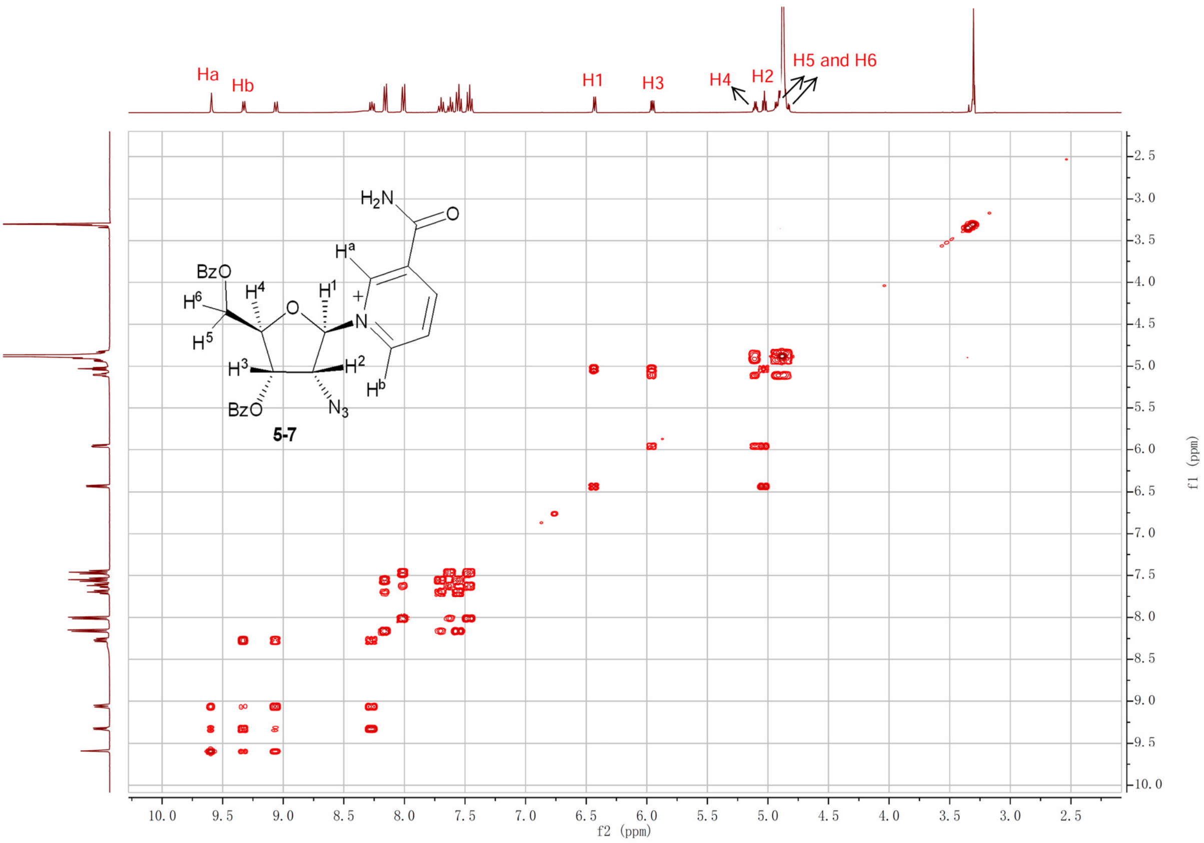


**Supplementary Figure 18.** g-COSY spectra of **5-7**. The solvent was $CD_3OD$.

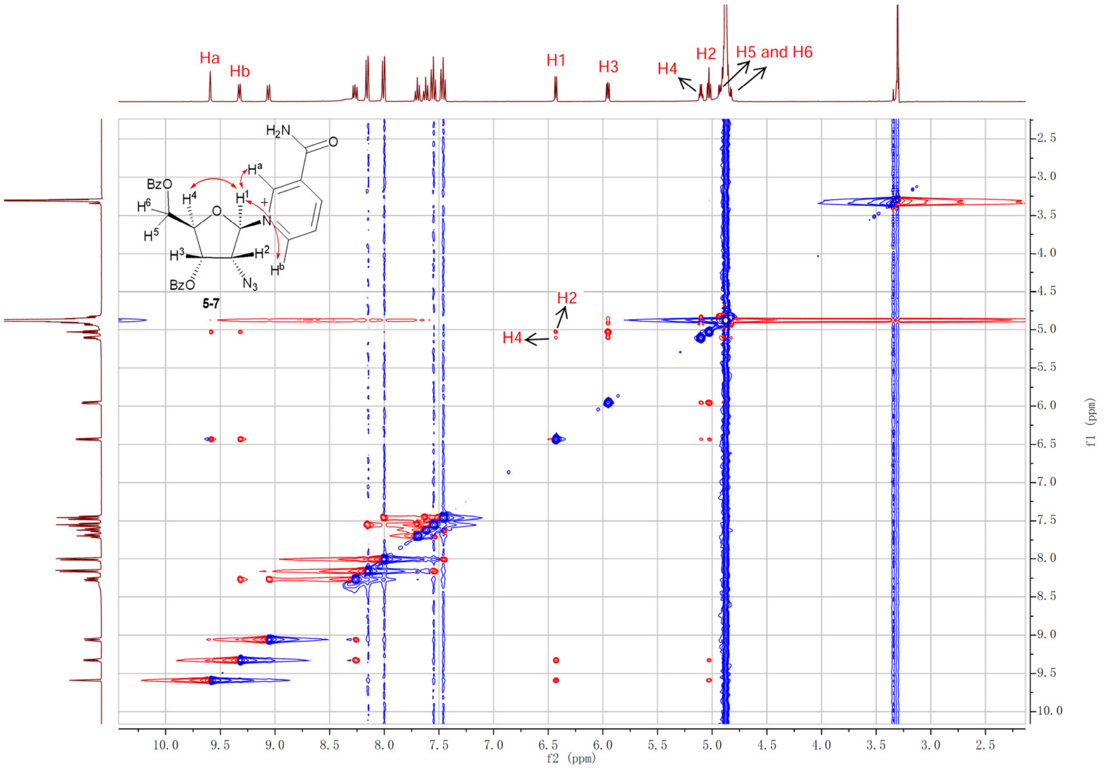


**Supplementary Figure 19.** NOESY spectra of **5-7**. The solvent was $CD_3OD$.

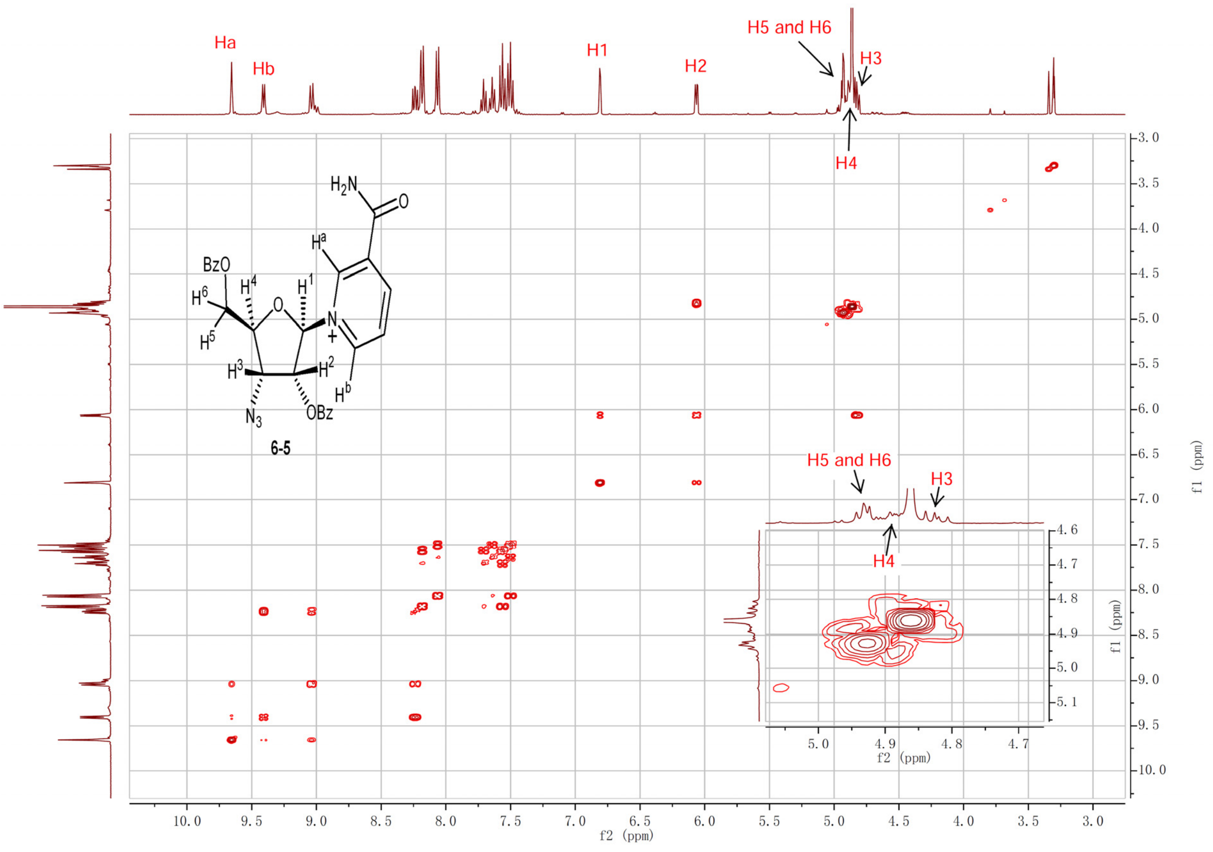


**Supplementary Figure 20.** g-COSY spectra of **6-5**. The solvent was CD3OD.

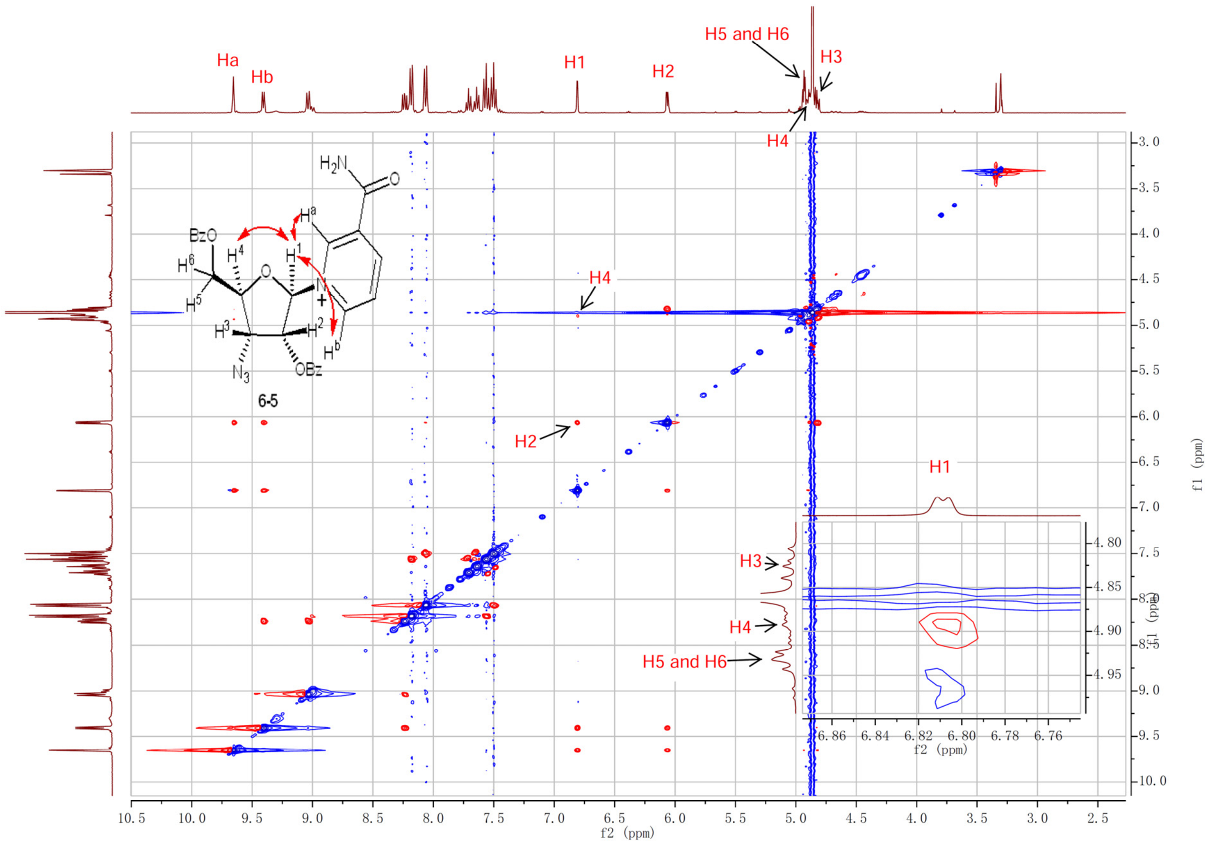


**Supplementary Figure 21.** NOESY spectra of **6-5**. The solvent was CD3OD.

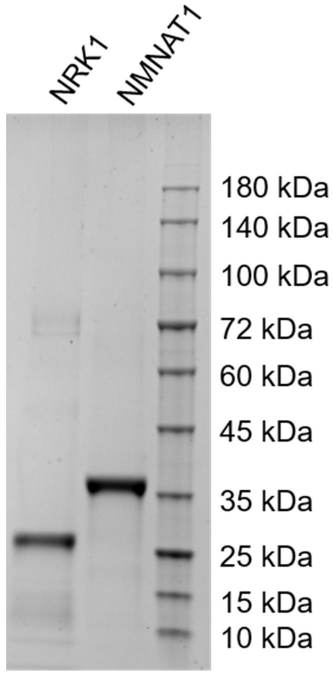


**Supplementary Figure 22**. SDS-PAGE gel of purified human NRK1 and NMNAT1. The gel was stained with Coomassie blue. Proteins loaded were normalized to 3 µg per well. Source data are provided as a Source Data file.

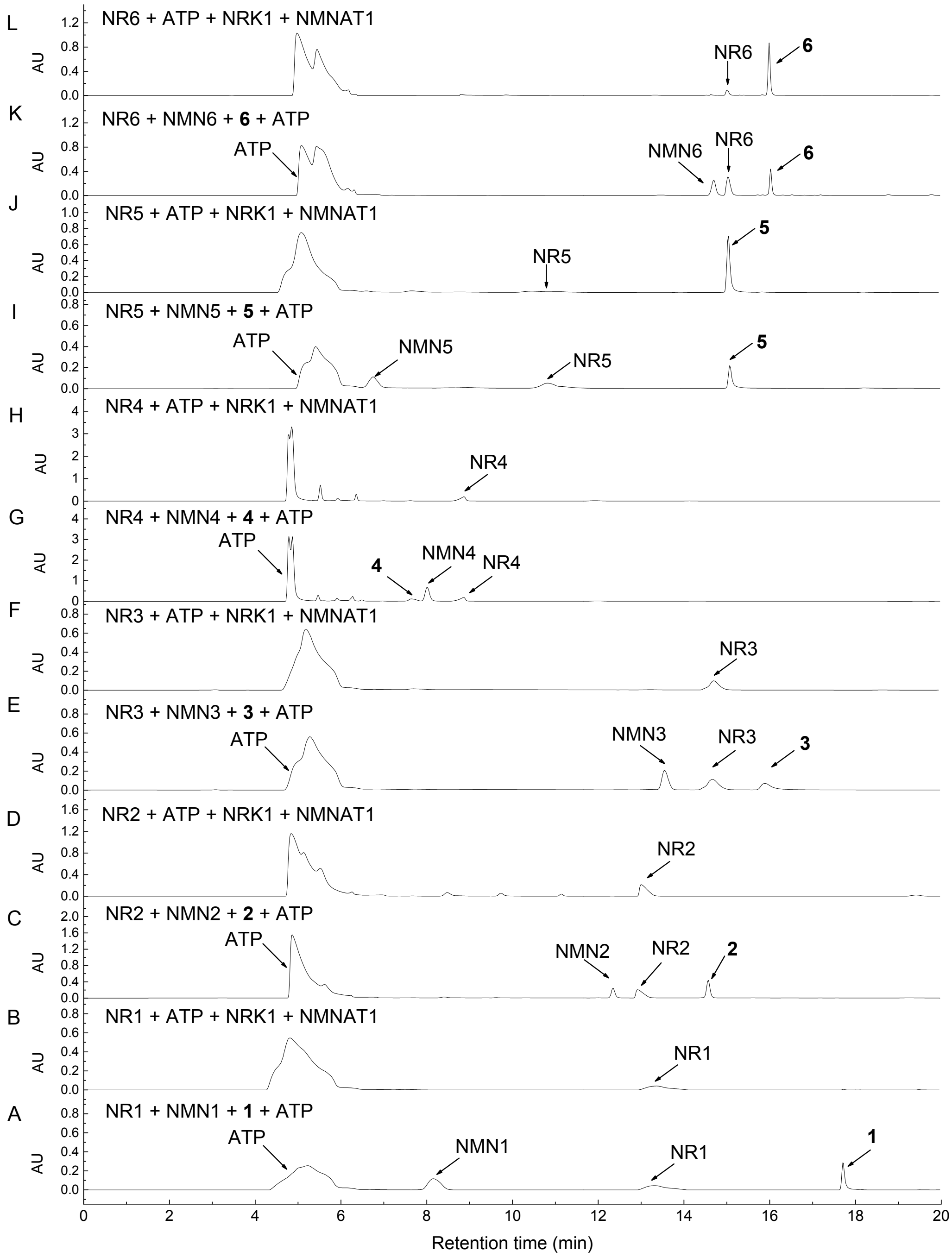


**Supplementary Figure 23**. HPLC analysis of the enzymatic conversion of NR1-6 to **1-6**. Reactions were conducted in the presence of purified NRK1 and NMNAT1 and analyzed using HPLC by UV absorbance at 260 nm. (A), (C), (E), (G), (I), and (K) show the assigned peaks for standard compounds. (B), (D), (F), (H), (J), and (L) NR1-6 were separately incubated with NRK1 and NMNAT1 in the presence of ATP in reaction buffer (50 mM Tris-HCl, pH 7.5, 100 mM NaCl, 20 mM $MgCl_2$, 1 mM DTT). After incubation at r.t for 24 h, 50% TCA was added to a final concentration of 10% to stop the reaction, followed by HPLC analysis. AU: absorbance unit.

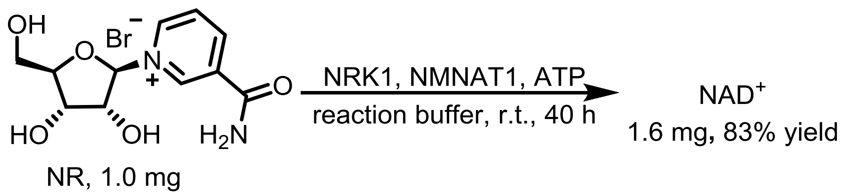


**Supplementary Figure 24**. Enzymatic synthesis of $NAD^+$ from NR. 1 mg of NR was added to the reaction with NRK1 (5 μM), NMNAT1 (5 μM), and ATP (5 mM) in reaction buffer (50 mM Tris-HCl, pH 7.5, 100 mM NaCl, 20 mM $MgCl_2$, 1 mM DTT) to the final concentration of 1 mM. After incubation at r.t. for 40 h, the reaction was stopped by adding 50% TCA to a final concentration of 10% and purified by HPLC.

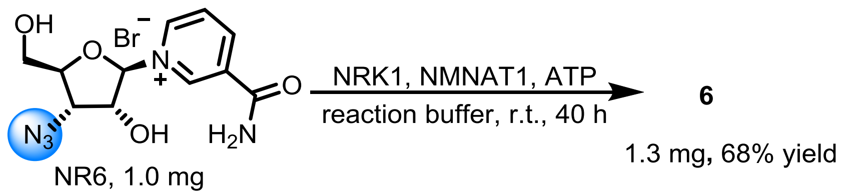


**Supplementary Figure 25**. Enzymatic synthesis of **6** from NR6. 1 mg of NR6 was added to the reaction with NRK1 (5 μM), NMNAT1 (5 μM), and ATP (5 mM) in reaction buffer (50 mM Tris-HCl, pH 7.5, 100 mM NaCl, 20 mM $MgCl_2$, 1 mM DTT) to the final concentration of 1 mM. After incubation at r.t. for 40 h, the reaction was stopped by adding 50% TCA to a final concentration of 10% and purified by HPLC.

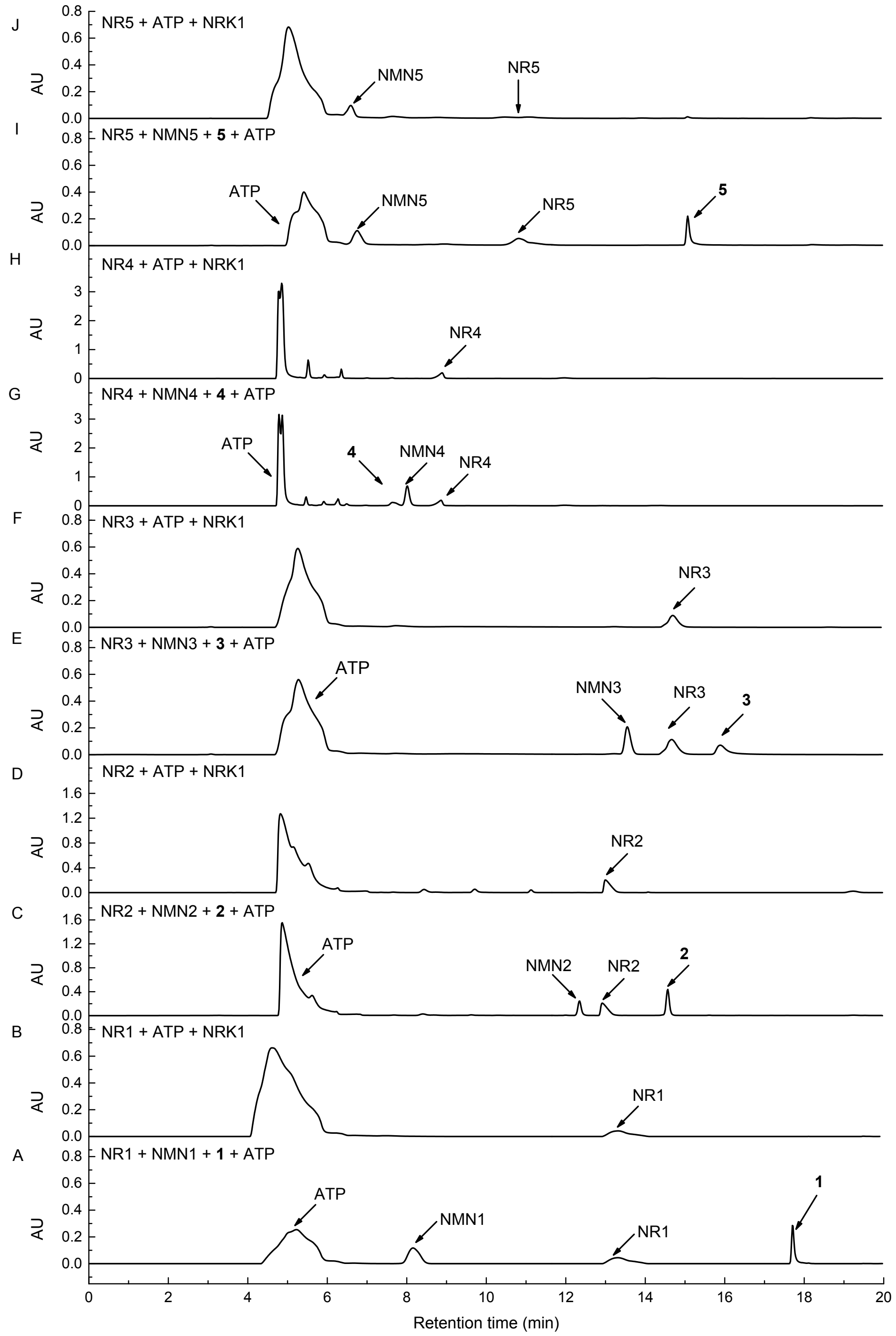


**Supplementary Figure 26**. HPLC analysis of the enzymatic conversion of NR1-5 to NMN1-5. Reactions were conducted in the presence of purified NRK1 and analyzed using HPLC by UV absorbance at 260 nm. (A), (C), (E), (G), and (I) show the assigned peaks for standard compounds. (B), (D), (F), (H), and (J) NR1-5 were separately incubated with NRK1 in the presence of ATP in reaction buffer (50 mM Tris-HCl, pH 7.5, 100 mM NaCl, 20 mM $MgCl_2$, 1 mM DTT). After incubation at r.t for 24 h, 50% TCA was added to a final concentration of 10% to stop the reaction, followed by HPLC analysis. AU: absorbance unit.

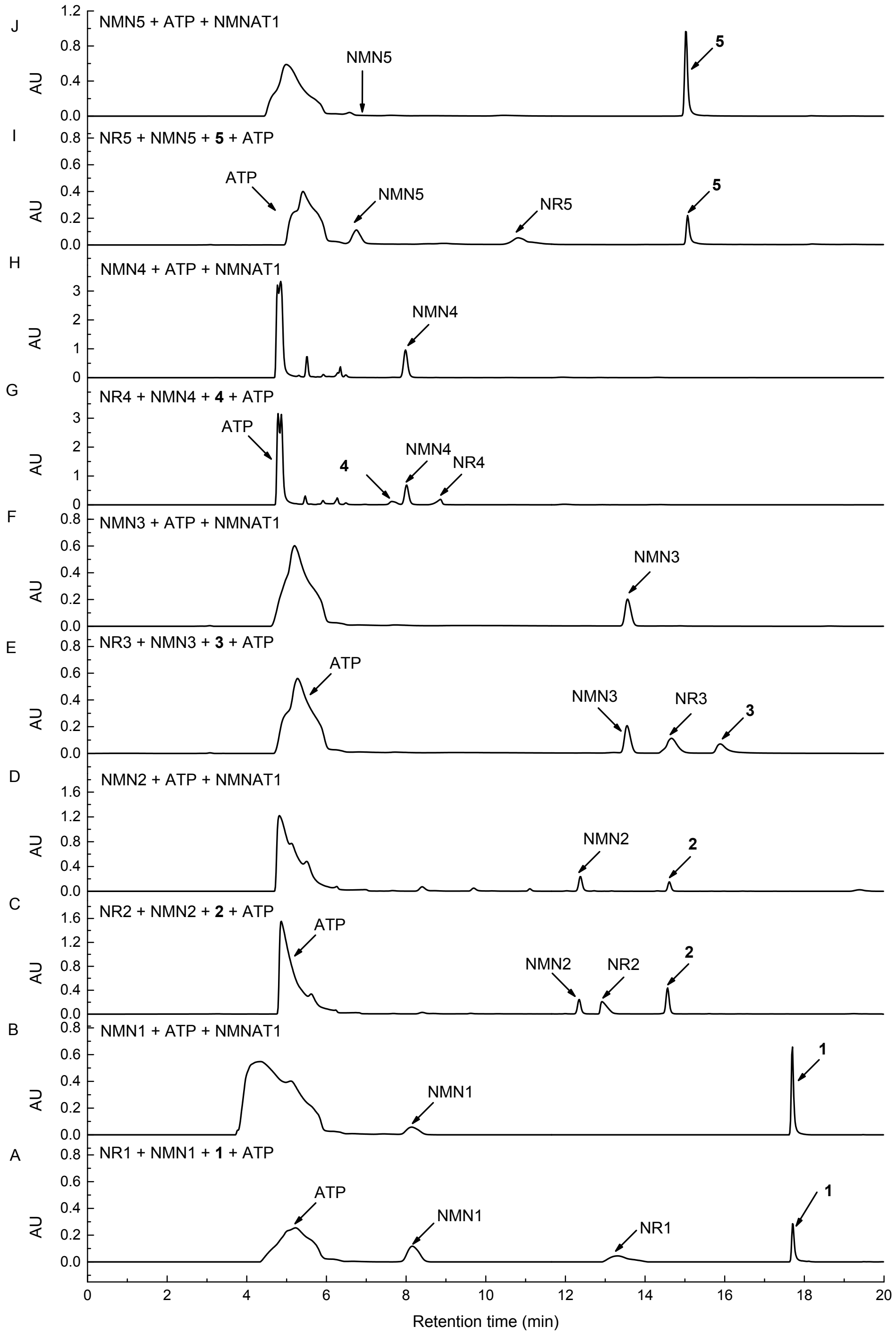


**Supplementary Figure 27**. HPLC analysis of the enzymatic conversion of NMN1-5 to **1-5**. Reactions were conducted in the presence of purified NMNAT1 and analyzed using HPLC by UV absorbance at 260 nm. (A), (C), (E), (G), and (I) show the assigned peaks for standard compounds. (B), (D), (F), (H), and (J) NMN1-5 were separately incubated with NMNAT1 in the presence of ATP in reaction buffer (50 mM Tris-HCl, pH 7.5, 100 mM NaCl, 20 mM $MgCl_2$, 1 mM DTT,). After incubation at r.t for 24 h, 50% TCA was added to a final concentration of 10% to stop the reaction, followed by HPLC analysis. AU: absorbance unit.

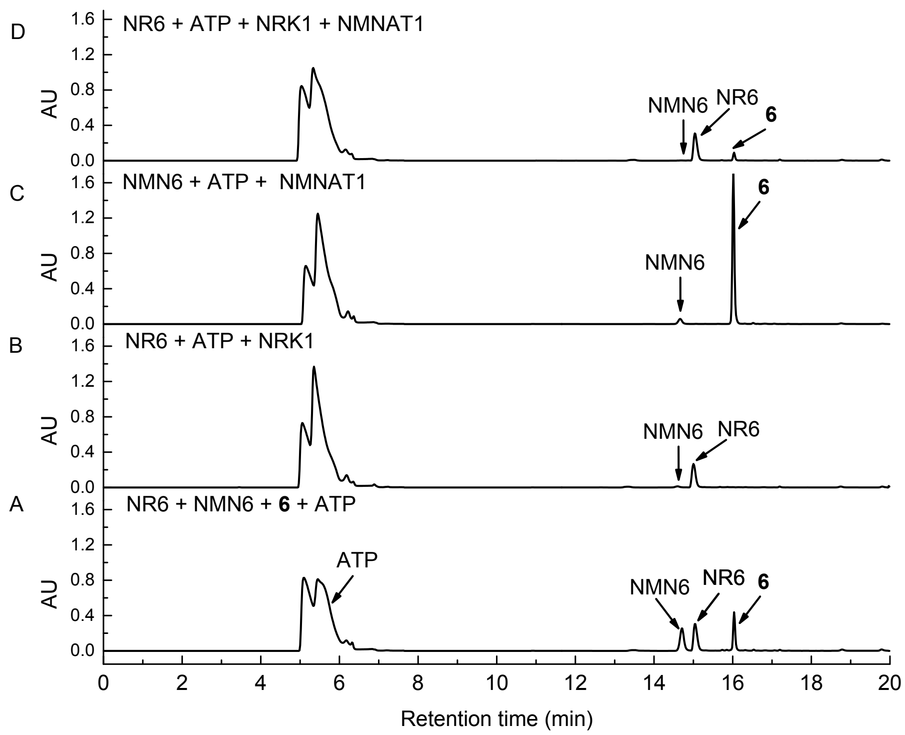


**Supplementary Figure 28**. HPLC analysis of the step-wise conversion from NR6 to **6**. (A) Assigned peaks for standard compounds. (B) and (C) NRK1 and NMNAK1 was used separately to catalyze the conversion process from NR6 to NMN6 (B), and NMN6 to **6** (C). (D) Both enzymes were then used together for the conversion from NR6 to **6.** All reactions were performed at r.t for 1 h in reaction buffer (50 mM Tris-HCl, pH 7.5, 100 mM NaCl, 20 mM $MgCl_2$, 1 mM DTT) with ATP, and were stopped by adding 50% TCA to a final concentration of 10%, followed by HPLC analysis as measured by UV absorbance at 260 nm. AU: absorbance unit.

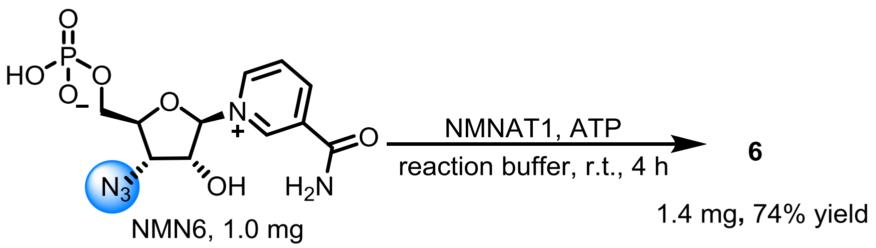


**Supplementary Figure 29**. Enzymatic synthesis of **6** from NMN6**.** 1 mg of NMN6 was added to the reaction with NRK1 (5 μM), NMNAT1 (5 μM), and ATP (5 mM) in reaction buffer (50 mM Tris-HCl, pH 7.5, 100 mM NaCl, 20 mM $MgCl_2$, 1 mM DTT) to the final concentration of 1 mM. After incubation at r.t. for 4 h, the reaction was stopped by adding 50% TCA to a final concentration of 10% and purified by HPLC.

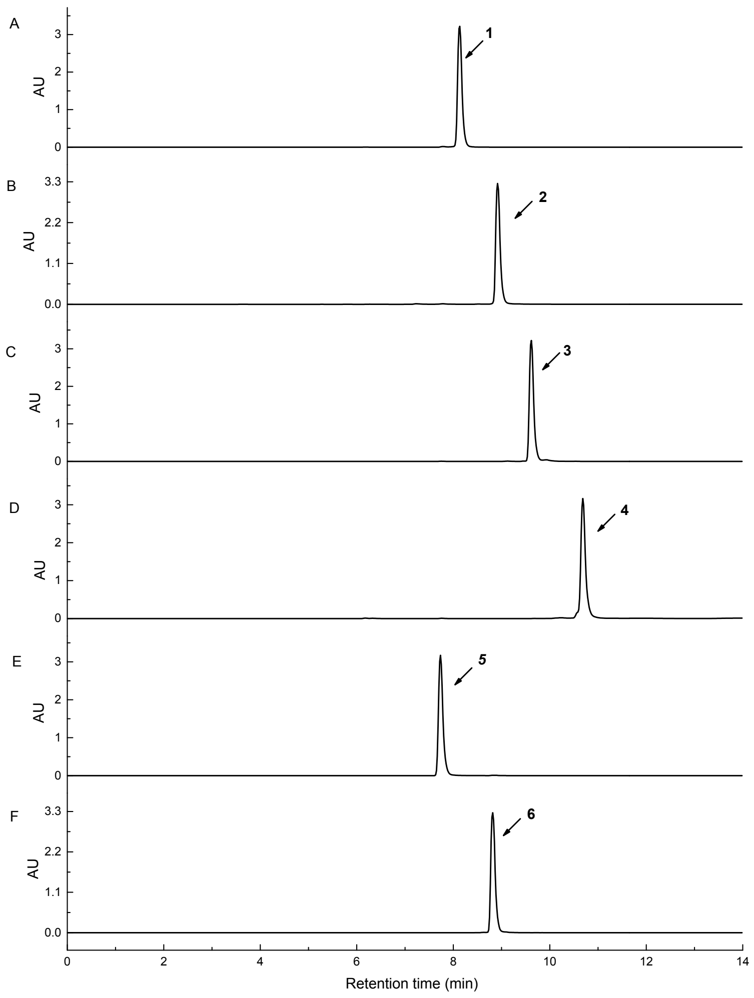


**Supplementary Figure 30.** HPLC purity analysis of **1-6**. (A), (B), (C), (D), (E), and (F) show separate analysis of $NAD^+$ analogue **1-6** (500 μM) by HPLC as measured by UV absorbance at 260 nm. The purities of **1-6** are summarized in Supplementary Table 2. AU: absorbance unit.

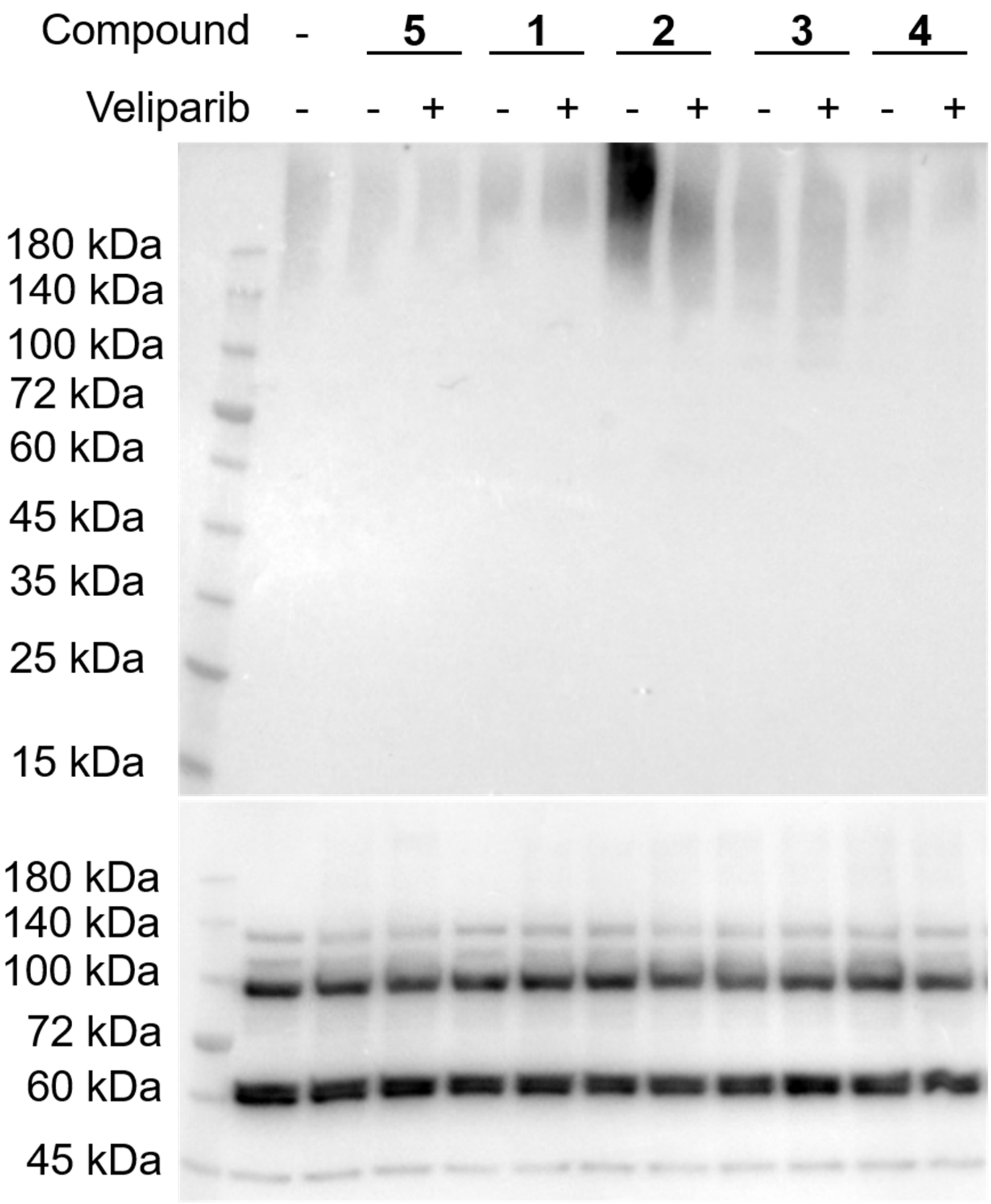


**Supplementary Figure 31.** Substrate activities of **1-5** for purified human full-length PARP1. Auto-PARylation of human PARP1 with **1-5** was performed by incubating purified human PARP1 with **1-5** with or without 100 μM veliparib at 30°C for 2 h, followed by immunoblot analysis as detected by the anti-PAR antibody (top panel). Bottom panel: PARP1 loading controls as detected by the anti-His$_6$ antibody. Source data are provided as a Source Data file.

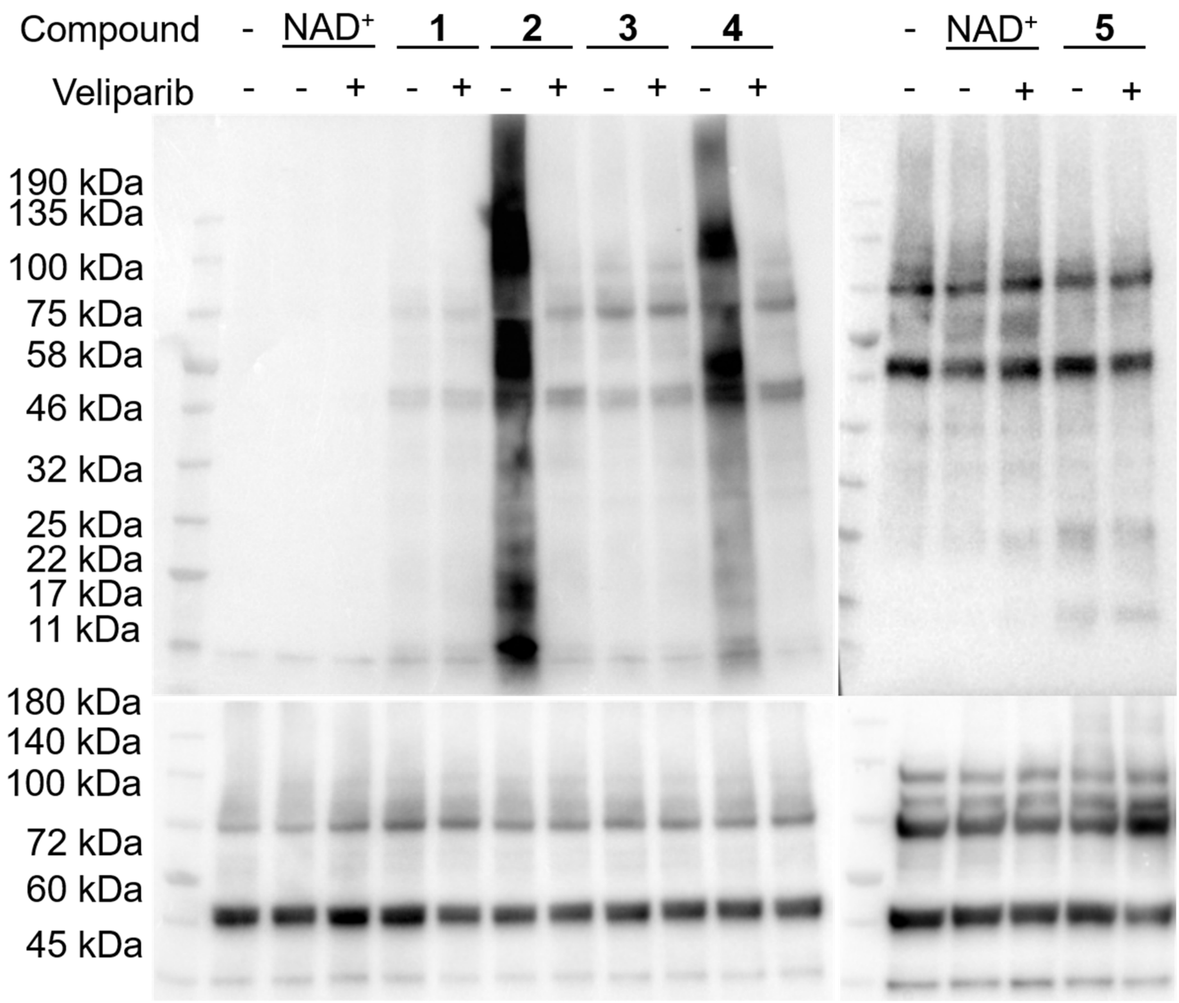


**Supplementary Figure 32**. Substrate activities of NAD$^+$ and **1-5** for human full-length PARP1. Auto-PARylation of PARP1 with NAD$^+$ or **1-5** was carried out by incubating purified PARP1 with 400 μM NAD$^+$ or **1-5** in the absence and presence of 100 μM veliparib at 30$^o$C for 2 h. The reaction mixtures were further labeled with azido-biotin (for NAD$^+$ and **1**-**4**) or alkyne-biotin (for NAD$^+$ and **5**) through click chemistry, followed by immunoblot analysis as detected by the streptavidin-HRP conjugate (top panel). Bottom panel: PARP1 loading controls as detected by the anti-His$_6$ antibody. Source data are provided as a Source Data file.

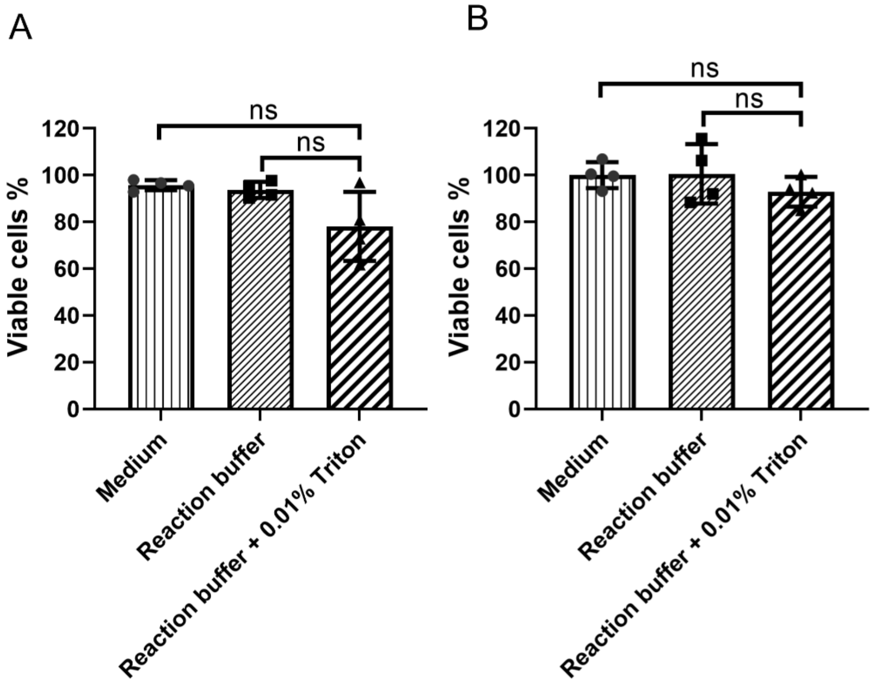


**Supplementary Figure 33**. Cell viability post-transient permeabilization. HeLa cells were incubated with reaction buffer with 0.01% Triton-X-100 for 15 minutes at 4°C. DMEM medium and reaction buffer were used as controls. Cells were then washed with PBS for three times and incubated with fresh medium at 37°C. Cell viability was evaluated by trypan blue method following 1 h incubation (A) or by MTT assay following 24 h incubation (B). ns = not significant, $P > 0.05$ by one-tailed unpaired *t* test. Error bars represent standard deviation of four replicates. Source data are provided as a Source Data file.

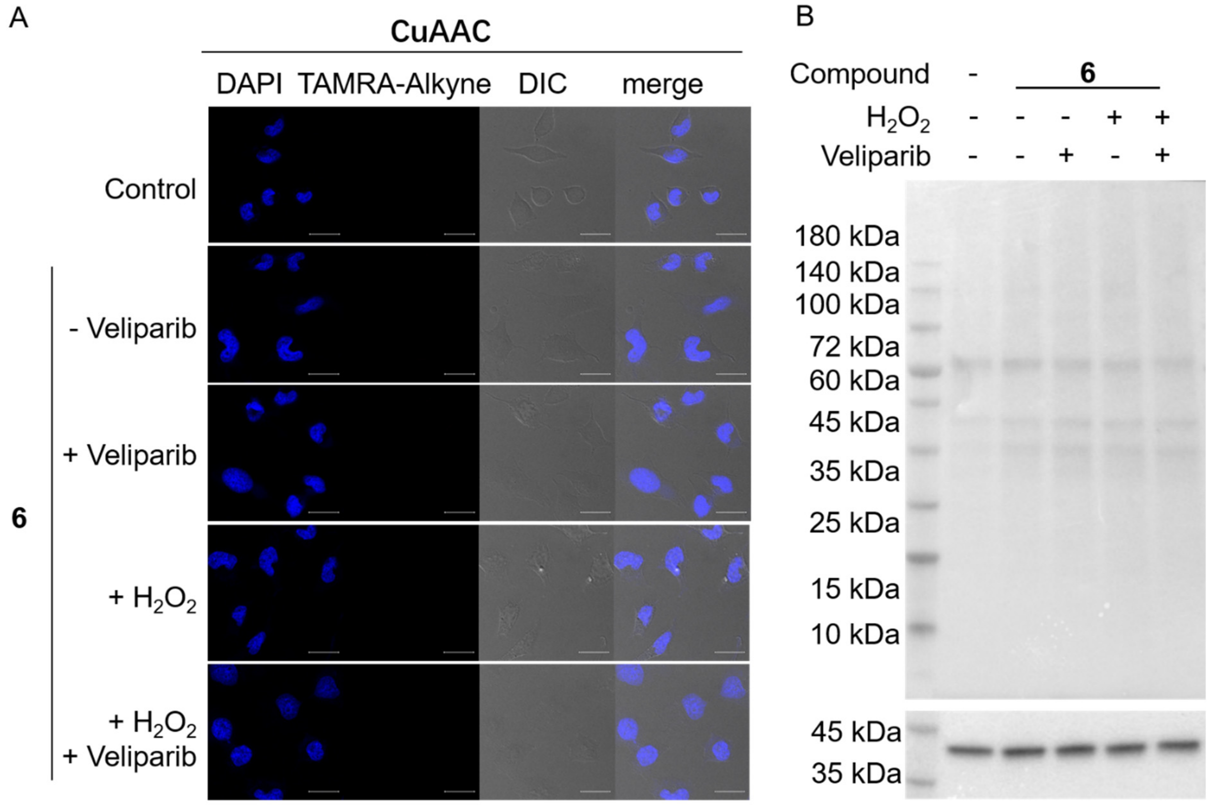


**Supplementary Figure 34**. Lack of labeling by **6** for cells without transient permeabilization. HeLa cells were pretreated for 20 min in the absence and presence of $H_2O_2$ and 1 μM veliparib. HeLa cells were then incubated with 100 μM **6** for 1 h, followed by (A) confocal microscopic analysis through fixation, permeabilization, and fluorescent staining via click chemistry, CuAAC: copper(I)-catalyzed azide alkyne cycloaddition. DIC: differential interference contrast. Scale bars: 20 μm, and by (B) immunoblot analysis of cell lysates as detected by a streptavidin-HRP conjugate following biotin conjugation via click chemistry (top panel). Bottom panel: GAPDH loading controls as detected by an anti-GAPDH antibody. Source data are provided as a Source Data file.

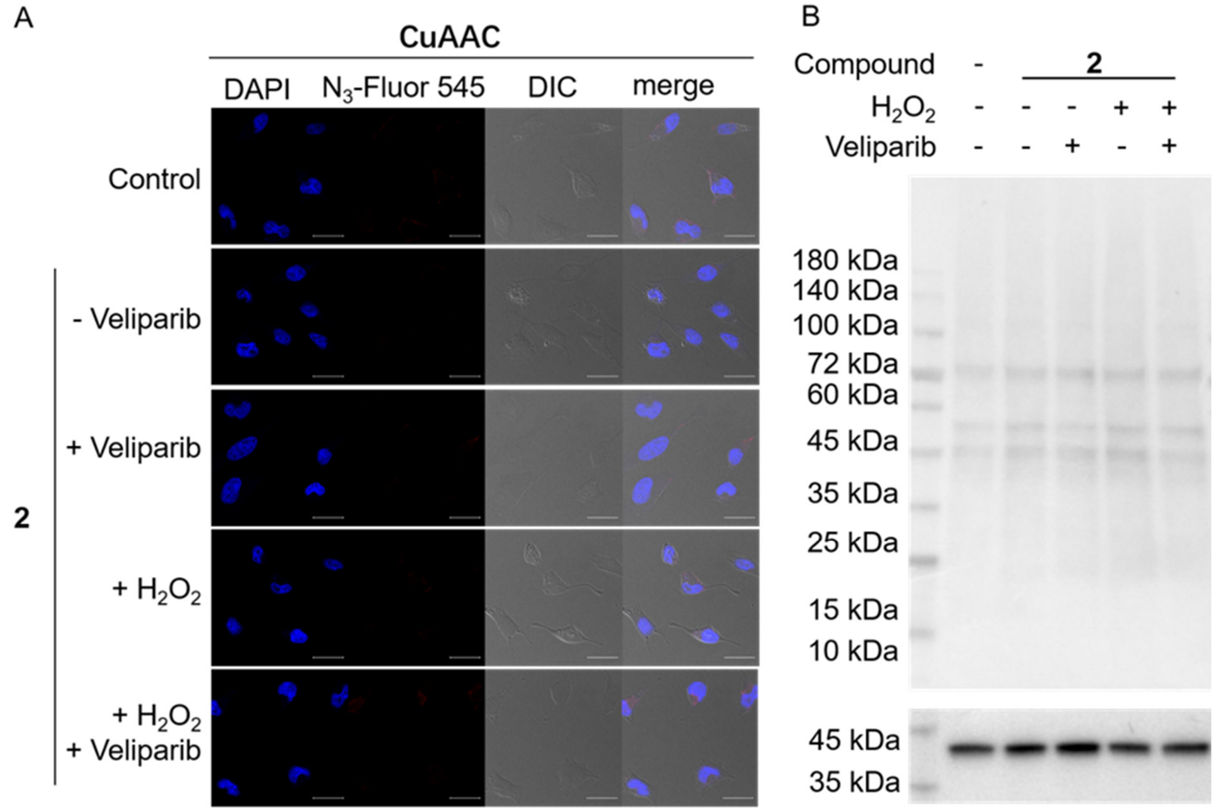


**Supplementary Figure 35**. Lack of labeling by **2** for cells without transient permeabilization. HeLa cells were pretreated for 20 min in the absence and presence of $H_2O_2$ and 1 μM veliparib. HeLa cells were then incubated with 200 μM **2** for 1 h, followed by (A) confocal microscopic analysis through fixation, permeabilization, and fluorescent staining via click chemistry, CuAAC: copper(I)-catalyzed azide alkyne cycloaddition. DIC: differential interference contrast. Scale bars: 20 μm, and by (B) immunoblot analysis of cell lysates as detected by a streptavidin-HRP conjugate following biotin conjugation via click chemistry (top panel). Bottom panel: GAPDH loading controls as detected by an anti-GAPDH antibody. Source data are provided as a Source Data file.

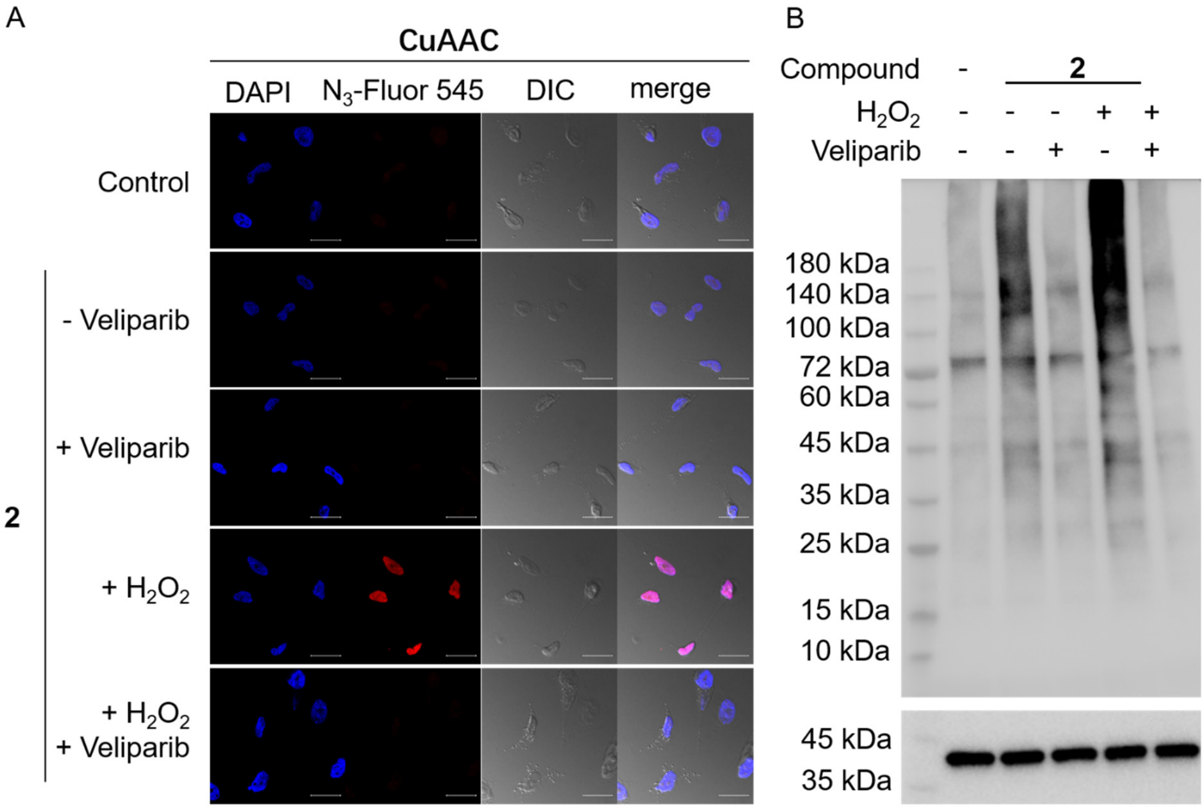


**Supplementary Figure 36**. Labeling of PARylation by **2** through transient permeabilization. HeLa cells were pretreated for 20 min in the absence and presence of $H_2O_2$ and 1 µM veliparib. Following transient permeabilization with 0.01% Triton-X-100, cells were incubated with 200 µM **2** for 45 min, followed by (A) confocal microscopic analysis through fixation, permeabilization, and fluorescent staining via click chemistry, CuAAC: copper(I)-catalyzed azide alkyne cycloaddition. DIC: differential interference contrast. Scale bars: 20 µm, and by (B) immunoblot analysis of cell lysates as detected by a streptavidin-HRP conjugate following biotin conjugation via click chemistry (top panel). Bottom panel: GAPDH loading controls as detected by an anti-GAPDH antibody. Source data are provided as a Source Data file.

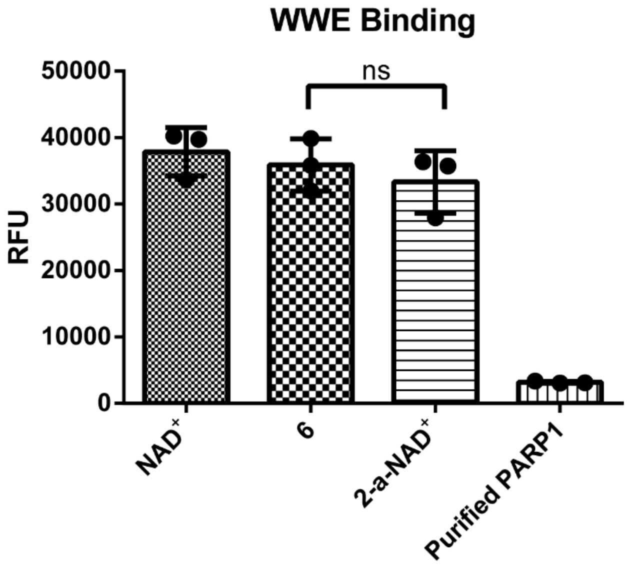


**Supplementary Figure 37.** WWE binding to automodified PARP1 by $NAD^+$, **6**, and 2-a-$NAD^+$. Purified PARP1 was used as a control. ns = not significant, $P > 0.05$ by one-tailed unpaired *t* test. Error bars represent standard deviation of three replicates. RFU: relative fluorescence unit. Source data are provided as a Source Data file.

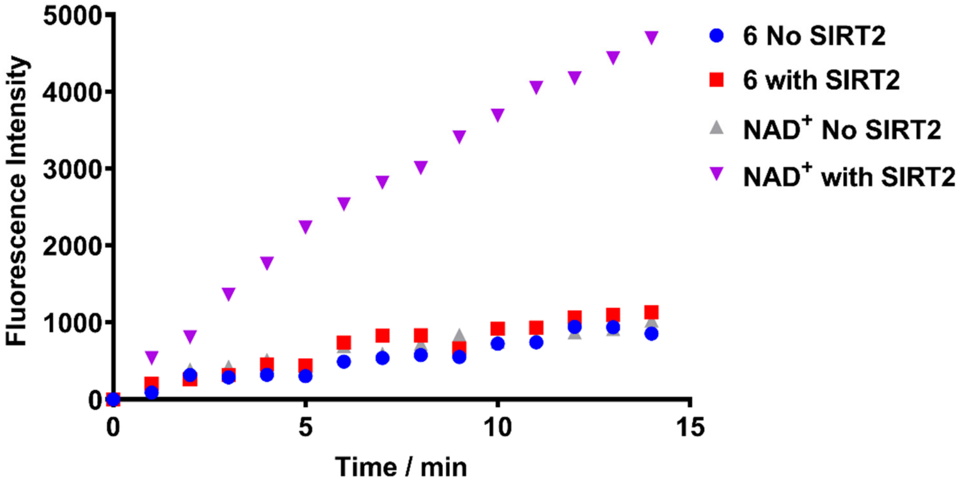


**Supplementary Figure 38**. Substrate activities of $NAD^+$ and **6** for human SIRT2. The deacetylation activity for SIRT2 was measured with a trypsin-coupled fluorescence-based assay at 460 nm based on the release of fluorescent 7-amino-4-methylcoumarin (AMC) from a deacetylate peptide substrate cleaved by bovine trypsin. $NAD^+$ or **6** (0.5 mM) was incubated with Ac-Arg-Gly-Lys(Ac)-AMC peptide and SIRT2 for overnight, followed by treatment with 50 nM bovine trypsin and measurements of fluorescence signals. Source data are provided as a Source Data file.

**Supplementary Tables**

**Supplementary Table 1**. Primers used for molecular cloning of human NRK1 and NMNAT1. Restriction enzyme sites for NcoI and XhoI are underlined and in italics.

| Name | Sequence |
|---|---|
| NRK1-Fw | 5′-*CCATGG*ATGAAAACATTTATCATTGGAATCAGTGG-3′ |
| NRK1-Rv | 5′-*CTCGAG*TGCTGTCACTTGCAAACACTTTTG-3′ |
| NMNAT1-Fw | 5′-*CCATGG*ATGCACCACCACCACCACCACGAAAATTCCGAGAAGACTGAAGTG-3′ |
| NMNAT1-Rv | 5′-*CTCGAG*CTACTACTATGTCTTAGCTTCTGCAGTGTTTC-3′ |

**Supplementary Table 2**. HPLC purity of $NAD^+$ analogues **1-6** used in biological experiments.

| $NAD^+$ analogues | HPLC purity |
|---|---|
| **1** | 99.3% |
| **2** | 98.5% |
| **3** | 98.7% |
| **4** | 97.9% |
| **5** | 99.8% |
| **6** | >99.8% |

## Supplementary Methods

### Synthesis of NAD$^+$ analogue 1 (Supplementary Figure 3).

To a stirred solution of methyl β-D-ribofuranoside (**SM-1**) (3.1 g, 19.0 mmol) in pyridine (24 mL) was added the TIPDSCl$_2$ (9.0 g, 28.5 mmol) at 0ºC. The reaction mixture was allowed to warm to room temperature. After stirring at this temperature for 24 hours, the reaction mixture was diluted with EtOAc (100 mL), and the organic phase was washed successively with ice-water (50 mL), aq 1M HCl (2×50 mL), $H_2O$ (2×50 mL), dried over anhydrous $Na_2SO_4$, filtered and concentrated to give a residue. The residue was purified by a flash column chromatography on silica gel to afford the compound **1-2** (7.0 g, 90%) as a colorless oil. $^1$H NMR (400 MHz, $CDCl_3$): δ 1.06-1.10 (m, 28H, 4CH+8CH$_3$), 3.31 (s, 3H, OCH$_3$), 3.75 (dd, 1H, *J* = 10.8, 8.8 Hz, CH$_2$), 3.99-4.07 (m, 3H, CH$_2$+2CH), 4.50 (t, 1H, *J* = 5.2 Hz, CH), 4.82 (s, 1H, CH) (Supplementary Figure 39); $^{13}$C NMR (100 MHz, $CDCl_3$): δ 12.5, 12.8, 13.25, 13.27, 16.94, 16.97, 17.0, 17.2, 17.36, 17.38, 17.4, 17.5, 54.9, 66.2, 75.0, 75.7, 82.7, 107.2 (Supplementary Figure 40).

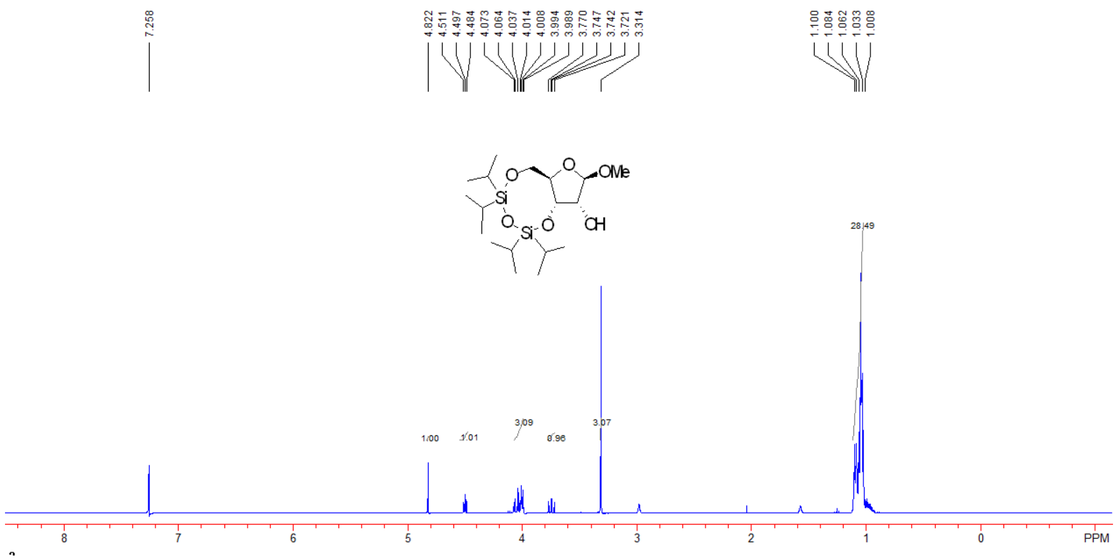


**Supplementary Figure 39**. $^1$H NMR spectrum of compound **1-2**.

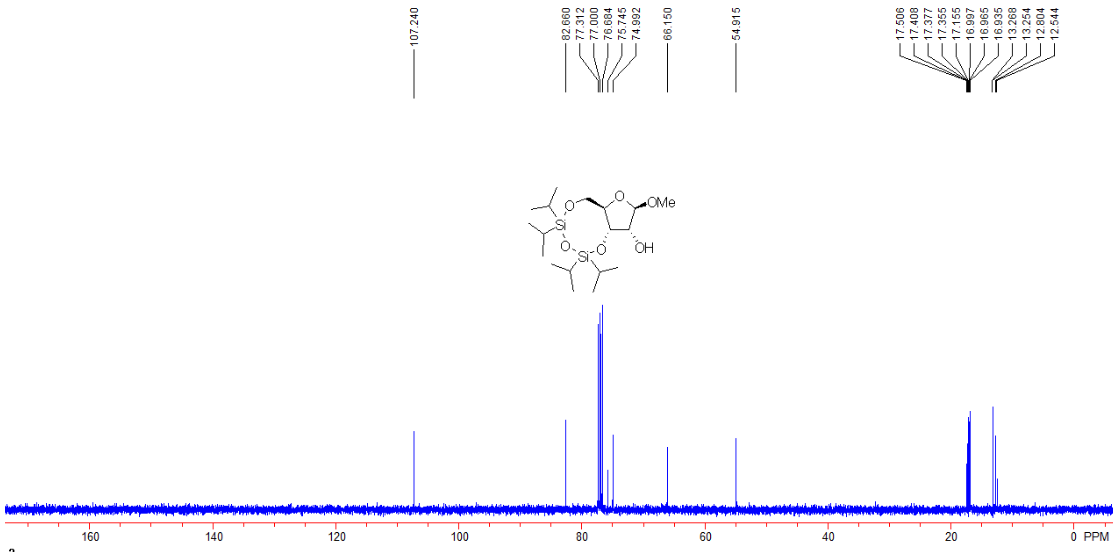


**Supplementary Figure 40**. $^{13}$C NMR spectrum of compound **1-2**.

To a stirred solution of compound **1-2** (1.5 g, 3.7 mmol) in anhydrous THF (25 mL) was added NaH (180 mg, 4.5 mmol, 1.2 eq, 60% dispersion in mineral oil) at 0°C followed by the addition of propargyl bromide (660 mg, 5.6 mmol, 1.5 eq) at the same temperature. Then, the reaction mixture was allowed to warm to room temperature. After stirring at this temperature for 6 hours, the reaction mixture was quenched with saturated aqueous $NH_4Cl$ (20 mL) and extracted with EtOAc (3×50 mL). The combined organic layers were washed water (3×50 mL), dried over anhydrous $Na_2SO_4$, filtered and concentrated and purified by a flash column chromatography on silica gel to afford the compound **1-3** (987 mg, 60%) as a colorless oil. $^1$H NMR (400 MHz, $CDCl_3$): δ 1.02-1.08 (m, 28H, 4CH+8$CH_3$), 2.43 (t, 1H, $J$ = 2.4 Hz, CH), 3.32 (s, 3H, $OCH_3$), 3.84-3.88 (m, 1H, $CH_2$), 3.96-4.02 (m, 3H, $CH_2$+2CH), 4.42-4.51 (m, 3H, $CH_2$+CH), 4.77 (s, 1H, CH) (Supplementary Figure 41); $^{13}$C NMR (100 MHz, $CDCl_3$): δ 12.6, 12.7, 13.1, 13.3, 16.97, 17.01, 17.09, 17.17, 17.29, 17.30, 17.4, 54.7, 58.2, 63.6, 73.9, 74.8, 79.7, 80.6, 80.9, 105.9 (Supplementary Figure 42).

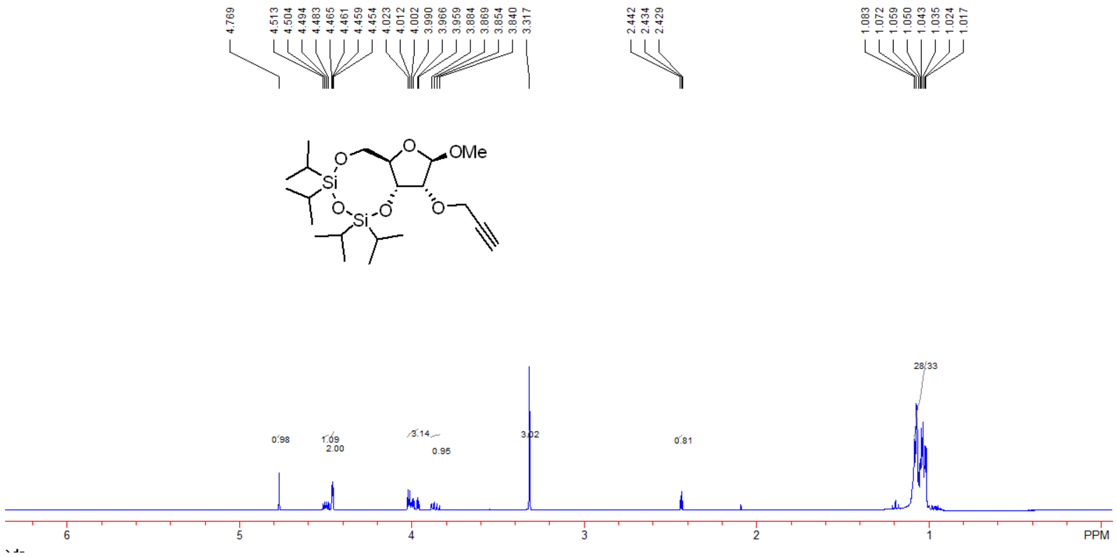


**Supplementary Figure 41**. $^{1}$H NMR spectrum of compound **1-3**.

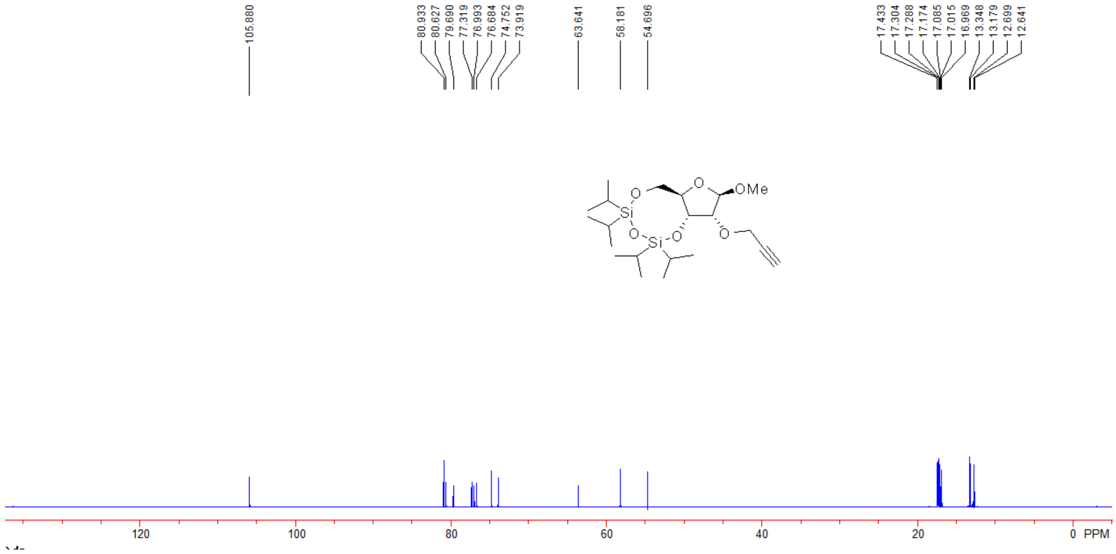


**Supplementary Figure 42**. $^{13}$C NMR spectrum of compound **1-3**.

To a 0°C solution of compound **1-3** (934 mg, 2.1 mmol) in anhydrous THF (25 mL) was added AcOH (180 μL, 3.2 mmol, 1.5 eq) followed by the addition of TBAF (3.2 mL, 3.2 mmol, 1.0 M in THF, 1.5 eq). Then, the reaction mixture was allowed to warm to room temperature. After stirring at this temperature for 14 hours, the reaction mixture was concentrated under reduced pressure to give a residue. The residue was purified by a flash column chromatography on silica gel to afford the compound **1-4** (386 mg, 91%) as a colorless oil. $^{1}$H NMR (400 MHz, $CDCl_3$): δ 2.52 (t, 1H, $J$ = 2.4 Hz, CH), 3.43 (s, 3H, $OCH_3$), 3.64 (dd, 1H, $J$ = 12.4, 3.6 Hz, $CH_2$), 3.80 (dd, 1H, $J$ = 12.4, 2.4 Hz, CH), 3.99 (dd, 1H, $J$ = 5.6, 1.2 Hz, CH), 4.05-4.08 (m, 1H, CH), 4,28-

4.39 (m, 3H, $CH_2$+CH), 4.98 (s, 1H, CH) (Supplementary Figure 43); $^{13}C$ NMR (100 MHz, $CDCl_3$): δ 55.9, 58.5, 63.0, 70.9, 75.7, 78.7, 82.7, 85.6, 106.5 (Supplementary Figure 44).

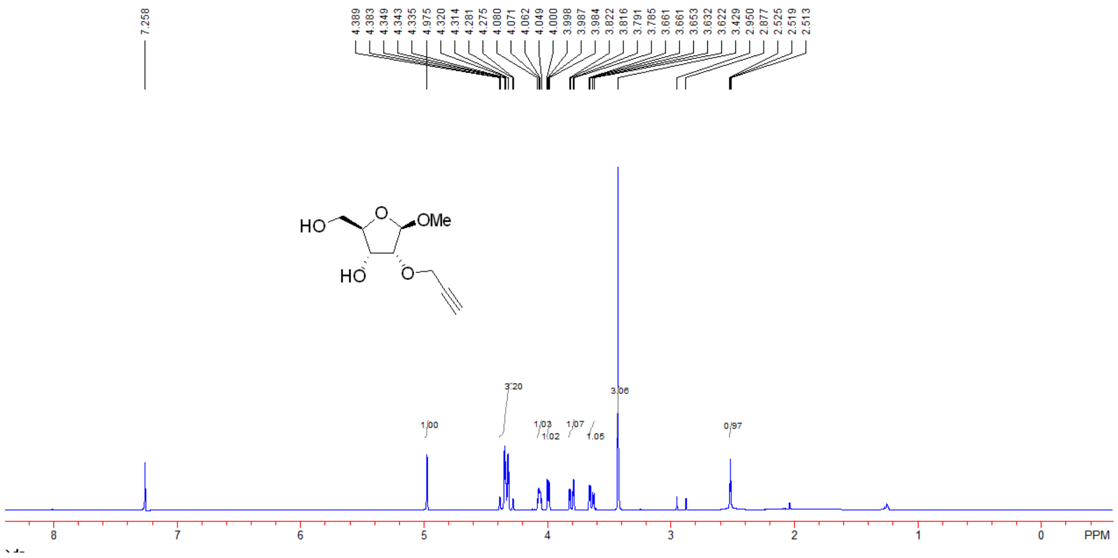


**Supplementary Figure 43**. $^1H$ NMR spectrum of compound **1-4**.

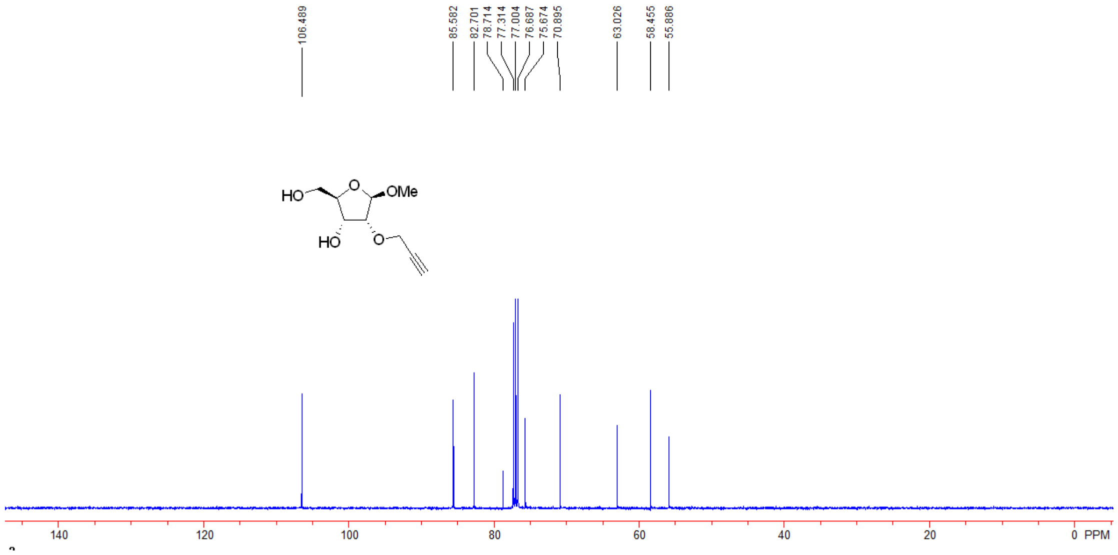


**Supplementary Figure 44**. $^{13}C$ NMR spectrum of compound **1-4**.

To a solution of compound **1-4** (320 mg, 1.7 mmol) in a mixture of anhydrous DCM (10 mL) and anhydrous pyridine (10 mL) was added BzCl (588 µL, 5.1 mmol, 3 eq) at 0°C. Then the reaction mixture was allowed to warm to room temperature. After stirring for 24 hours, the reaction was quenched with MeOH (10 mL) and the mixture was concentrated under reduced pressure to give a residue. The residue was dissolved in EtOAc (50 mL), and the organic phase

was washed successively with saturated aqueous $CuSO_4$ (3×50 mL), brine (50 mL), dried over anhydrous $Na_2SO_4$, filtered, concentrated and purified by a flash column chromatography on silica gel to afford the compound **1-5** (558 mg, 80%) as a colorless oil. $^1$H NMR (400 MHz, $CDCl_3$): δ 2.39 (t, 1H, *J* = 2.4 Hz, CH), 3.38 (s, 3H, $OCH_3$), 4.23 (dd, 1H, *J* = 16.0, 2.4 Hz, $CH_2$), 4.29 (dd, 1H, *J* = 16.0, 2.4 Hz, $CH_2$), 4.40 (dd, 1H, *J* = 4.8, 1.2 Hz, CH), 4.43-4.49 (m, 1H, $CH_2$), 4.59-4.66 (m, 2H, $CH_2$+CH), 5.08 (d, 1H, *J* = 0.4 Hz, CH), 5.55 (dd, 1H, *J* = 6.4, 4.8 Hz, CH), 7.39 (t, 2H, *J* = 8.0 Hz, ArH), 7.45 (t, 2H, *J* = 8.0 Hz, ArH), 7.51-7.56 (m, 1H, ArH), 7.57-7.61 (m, 1H, ArH), 8.05-8.07 (m, 4H, ArH) (Supplementary Figure 45); $^{13}$C NMR (100 MHz, $CDCl_3$): δ 55.4, 58.5, 64.7, 73.6, 75.3, 78.7, 78.9, 80.4, 106.8, 128.3, 128.5, 129.3, 129.73, 129.76, 129.84, 133.1, 133.4, 165.8, 166.2 (Supplementary Figure 46).

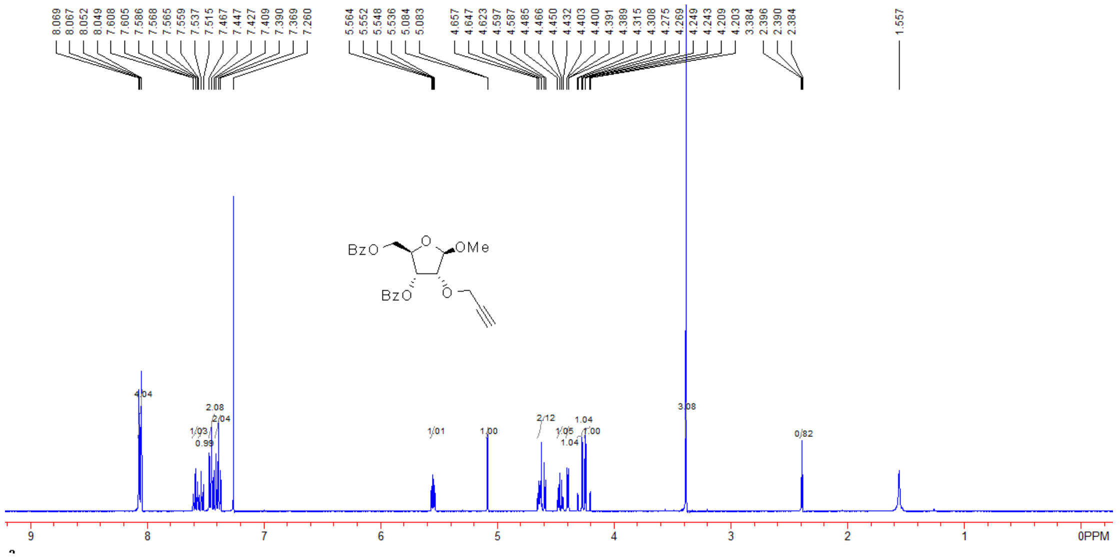


**Supplementary Figure 45**. $^1$H NMR spectrum of compound **1-5**.

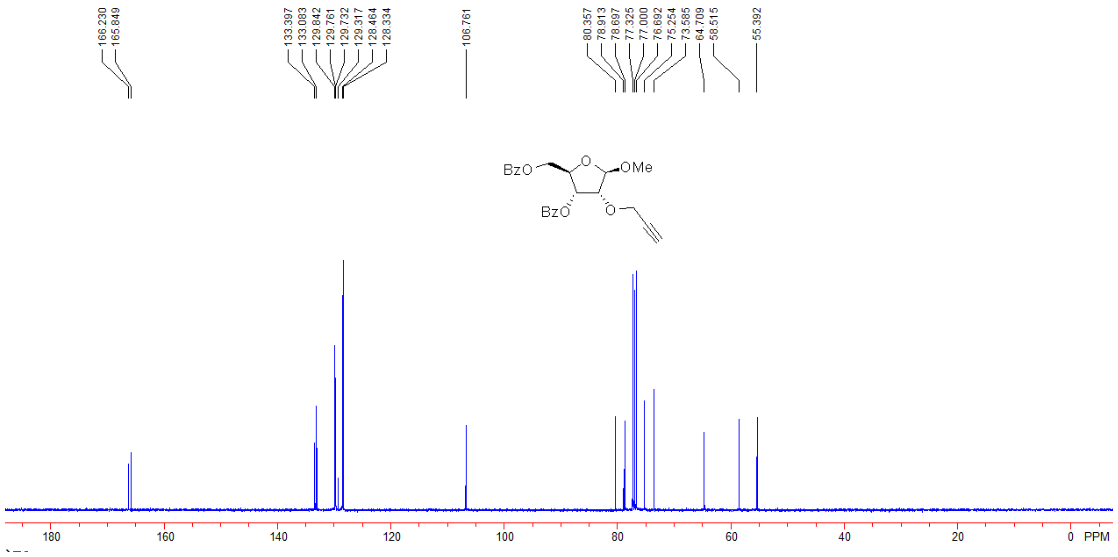


**Supplementary Figure 46**. $^{13}$C NMR spectrum of compound **1-5**.

Compound **1-5** (534 mg, 1.3 mmol) was dissolved in a mixture of TFA/$H_2O$ (9/1, 15 mL) and the resulting mixture was stirred at room temperature until the reaction completed (monitoring by TLC). Then the reaction was diluted with DCM (60 mL) and the solution was added dropwise to a stirred mixture of ice and saturated aqueous $NaHCO_3$. Solid $NaHCO_3$ was added during the addition to maintain a pH of 7. The mixture was extracted with DCM (3×50 mL), and the combined organic extracts was washed with $H_2O$ (50 mL), brine (50 mL), dried over anhydrous $Na_2SO_4$, filtered, concentrated to give a residue. The residue was dissolved in pyridine (15 mL) and cooled to 0°C. $Ac_2O$ (0.5 mL) was added dropwise and then the resulting mixture was allowed to warm to room temperature. After stirring for 6 hours, the reaction was quenched with MeOH (10 mL) and the mixture was concentrated under reduced pressure to give a residue. The residue was dissolved in EtOAc (50 mL), and the organic phase was washed successively with saturated aqueous $CuSO_4$ (3×50 mL), brine (50 mL), dried over anhydrous $Na_2SO_4$, filtered, concentrated and purified by a flash column chromatography on silica gel to afford the compound **1-6** (445 mg, 78%) as a colorless oil. $^1$H NMR (400 MHz, $CDCl_3$) of one isomer: δ 1.98 (s, 3H, $CH_3$), 2.39 (t, 1H, $J$ = 2.4 Hz, CH), 4.23-4.32 (m, 2H, $CH_2$), 4.43-4.48 (m, 1H, $CH_2$), 4.51 (d, 1H, $J$ = 5.2 Hz, CH), 4.67-4.73 (m, 2H, CH+$CH_2$), 4.53 (dd, 1H, $J$ = 6.8, 4.8 Hz, CH), 6.32 (s, 1H, CH), 7.40 (t, 2H, $J$ = 8.0 Hz, ArH), 7.46 (t, 2H, $J$ = 8.0 Hz, ArH), 7.55 (t, 1H, $J$ = 8.0 Hz, ArH), 7.58-7.62 (m, 1H, ArH), 8.05-8.07 (m, 4H, ArH) (Supplementary Figure 47); $^{13}$C

NMR (100 MHz, $CDCl_3$): δ 21.0, 58.5, 63.7, 72.3, 75.6, 78.5, 79.7, 79.8, 99.0, 128.4, 128.5, 129.0, 129.6, 129.8, 129.9, 133.2, 133.6, 165.9, 166.0, 169.5 (Supplementary Figure 48).

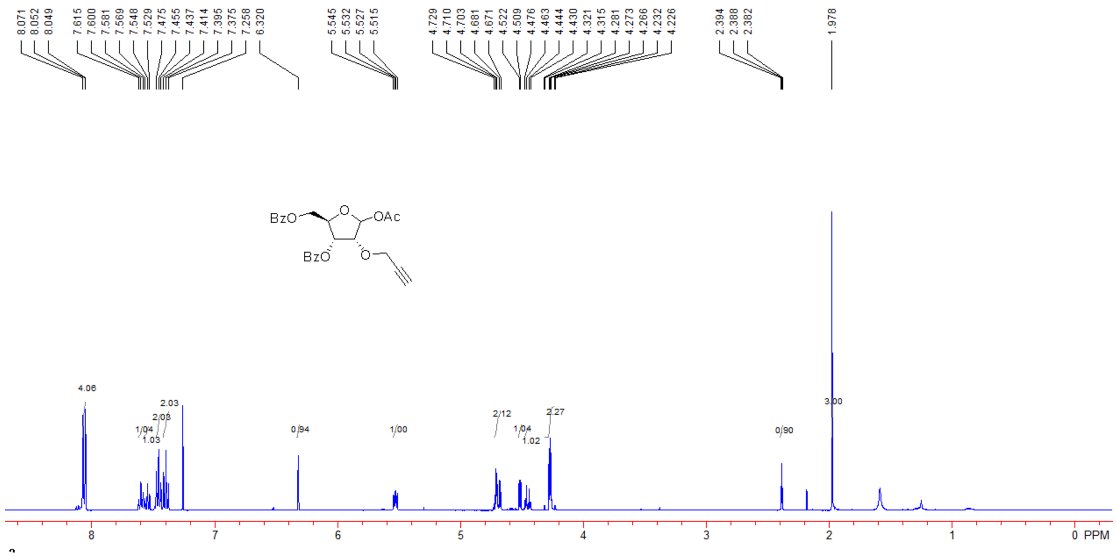

**Supplementary Figure 47**. $^1$H NMR spectrum of compound **1-6**.

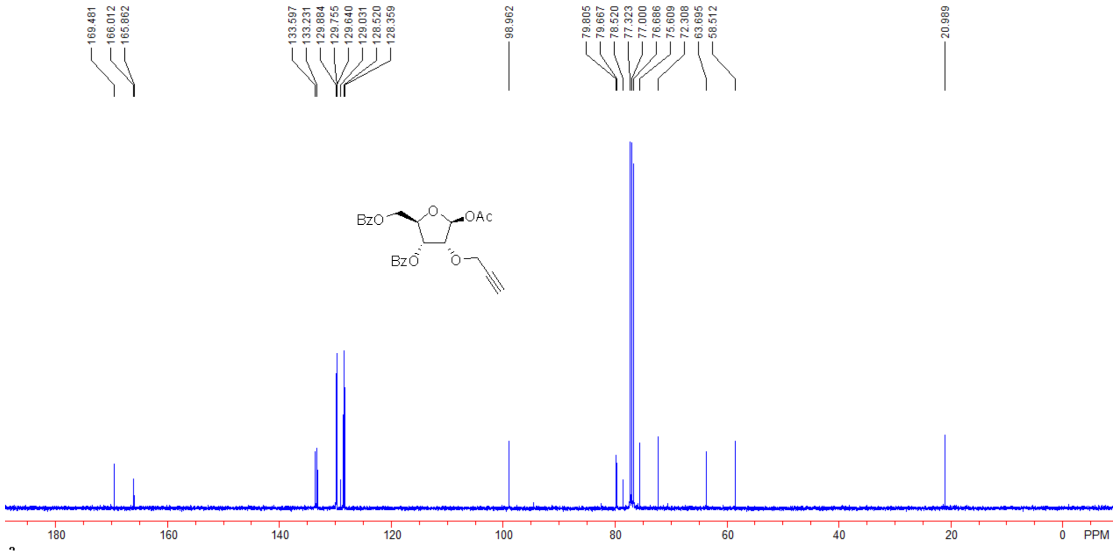

**Supplementary Figure 48**. $^{13}$C NMR spectrum of compound **1-6**.

To a stirred solution of compound **1-6** (307 mg, 0.70 mmol) and nicotinamide (128 mg, 1.05 mmol, 1.5 eq) in anhydrous $CH_3CN$ (15 mL), TMSOTf (506 µL, 2.8 mmol, 4.0 eq) was added dropwise at 0°C and the reaction mixture was stirred for 2 hours at the same temperature. Then the reaction was quenched with MeOH (1 mL) and the mixture was concentrated under reduced pressure to give a residue. The residue was purified by a flash column chromatography on silica

gel to afford the crude product which was then purified via preparative HPLC (C18-A column, 150×10.0 mm, 5 μm) (mobile phase A: 0.1% formic acid (aq), mobile B: 0.1% formic acid in acetonitrile; flow rate = 2.0 ml min$^{-1}$; 0-2 min: 0-4% B, 2-4 min: 4-10% B, 4-6 min: 10-20% B, 6-12 min: 20-50% B, 12-17 min: 50-100% B, 17-20 min: 100-0% B). Fractions containing the desired product were concentrated and lyophilized to yield the **1-7** (237 mg, 52% yield) as a colorless solid. $^{1}$H NMR (400 MHz, $CD_3OD$): δ 2.77 (t, 1H, *J* = 2.4 Hz, CH), 4.35 (dd, 1H, *J* = 16.4, 2.4 Hz, $CH_2$), 4.40 (dd, 1H, *J* = 16.4, 2.4 Hz, $CH_2$), 4.79 (dd, 1H, *J* = 6.4, 5.2 Hz, CH), 4.86 (dd, 1H, *J* = 12.8, 3.2 Hz, $CH_2$), 4.91 (dd, 1H, *J* = 12.8, 3.6 Hz, $CH_2$), 5.20-5.22 (m, 1H, CH), 5.98 (dd, 1H, *J* = 4.8, 1.6 Hz, CH), 6.53 (d, 1H, *J* = 6.4 Hz, CH), 7.41-7.45 (m, 2H, ArH), 7.53-7.57 (m, 2H, ArH), 7.59-7.63 (m, 1H, ArH), 7.67-7.71 (m, 1H, ArH), 7.91-7.94 (m, 2H, ArH), 8.15-8.17 (m, 2H, ArH), 8.24 (dd, 1H, *J* = 8.0, 6.4 Hz, ArH), 9.02 (dt, 1H, *J* = 8.0, 1.6 Hz, ArH), 9.32 (d, 1H, *J* = 6.4 Hz, ArH), 9.59 (s, 1H, ArH) (Supplementary Figure 49); $^{13}$C NMR (100 MHz, $CD_3OD$): δ 59.8, 65.2, 73.2, 78.0, 79.4, 84.0, 86.6, 99.2, 129.5, 129.81, 129.88, 130.37, 130.44, 130.57, 131.0, 134.9, 135.0, 135.9, 141.5, 143.8, 147.2, 164.4, 166.8, 167.4 (Supplementary Figure 50).

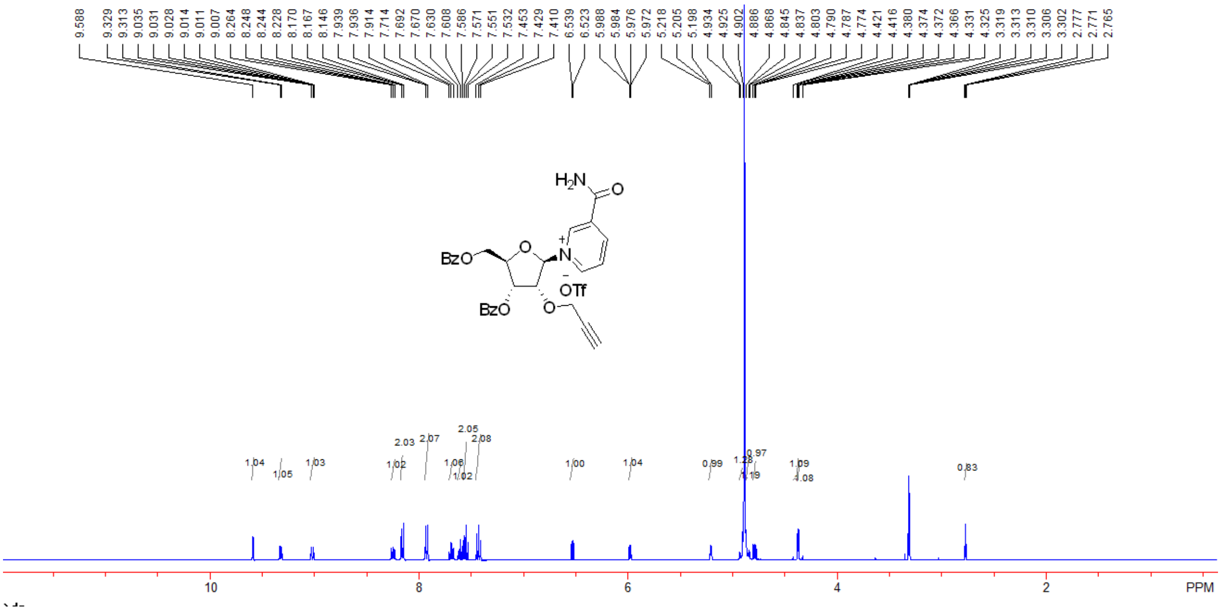


**Supplementary Figure 49**. $^{1}$H NMR spectrum of compound **1-7**.

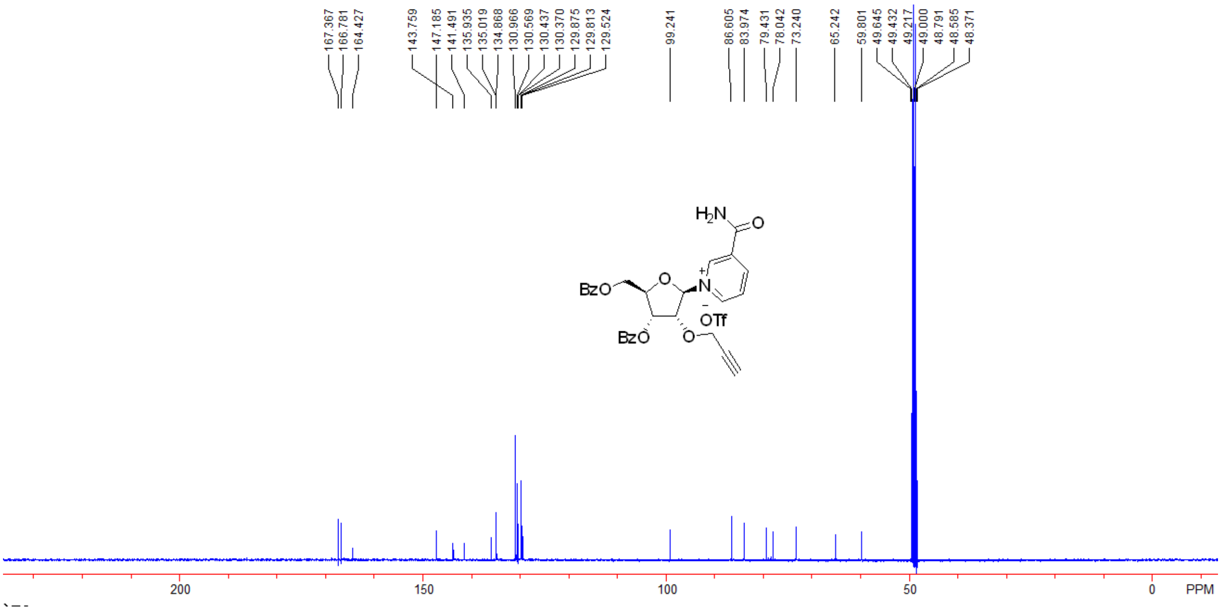


**Supplementary Figure 50**. $^{13}$C NMR spectrum of compound **1-7**.

Compound (**1-7**) (195 mg, 0.3 mmol) was dissolved in ammonia (15 mL, 7 N in MeOH) and the reaction was stirred at 4°C for 20 hours. The reaction was concentrated under reduced pressure and the crude product was purified via preparative HPLC (C18-A column, 150×10.0 mm, 5 μm) (mobile phase A: 0.1% formic acid (aq), mobile B: 0.1% formic acid in acetonitrile; flow rate = 2.0 ml min$^{-1}$; 0-2 min: 0-4% B, 2-4 min: 4-10% B, 4-6 min: 10-20% B, 6-12 min: 20-50% B, 12-14 min: 50-0% B. Fractions containing the desired product were concentrated and lyophilized to yield the NR1 (90 mg, 68% yield) as a colorless solid. $^{1}$H NMR (400 MHz, $D_2O$): δ 2.80 (t, 1H, $J$ = 2.4 Hz, CH), 3.88 (dd, 1H, $J$ = 12.8, 3.2 Hz, $CH_2$), 4.03 (dd, 1H, $J$ = 12.8, 2.4 Hz, $CH_2$), 4.33 (dd, 1H, $J$ = 16.0, 2.4 Hz, $CH_2$), 4.46 (dd, 1H, $J$ = 16.0, 2.4 Hz, $CH_2$), 4.50-4.55 (m, 3H, 3CH), 6.36 (d, 1H, $J$ = 4.0 Hz, CH), 8.28 (dd, $J$ = 8.4, 6.4 Hz, 1H, ArH), 8.98 (dt, 1H, $J$ = 8.4, 1.6 Hz, ArH), 9.28 (d, 1H, $J$ = 6.4 Hz, ArH), 9.60 (s, 1H, ArH) (Supplementary Figure 51); $^{13}$C NMR (100 MHz, $D_2O$): δ 58.4, 60.1, 68.6, 76.8, 78.4, 83.7, 88.6, 98.1, 119.5 (q, $J$ = 315.5 Hz), 128.3, 133.8, 140.4, 142.6, 145.7 (Supplementary Figure 52).

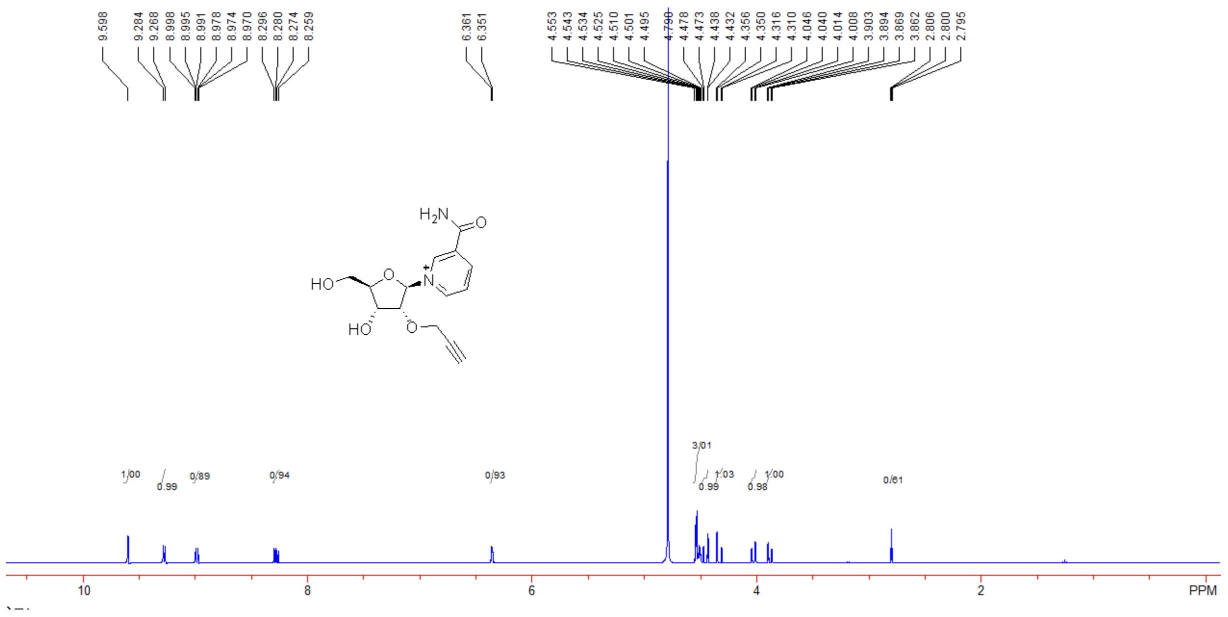

**Supplementary Figure 51**. $^{1}$H NMR spectrum of compound NR1.

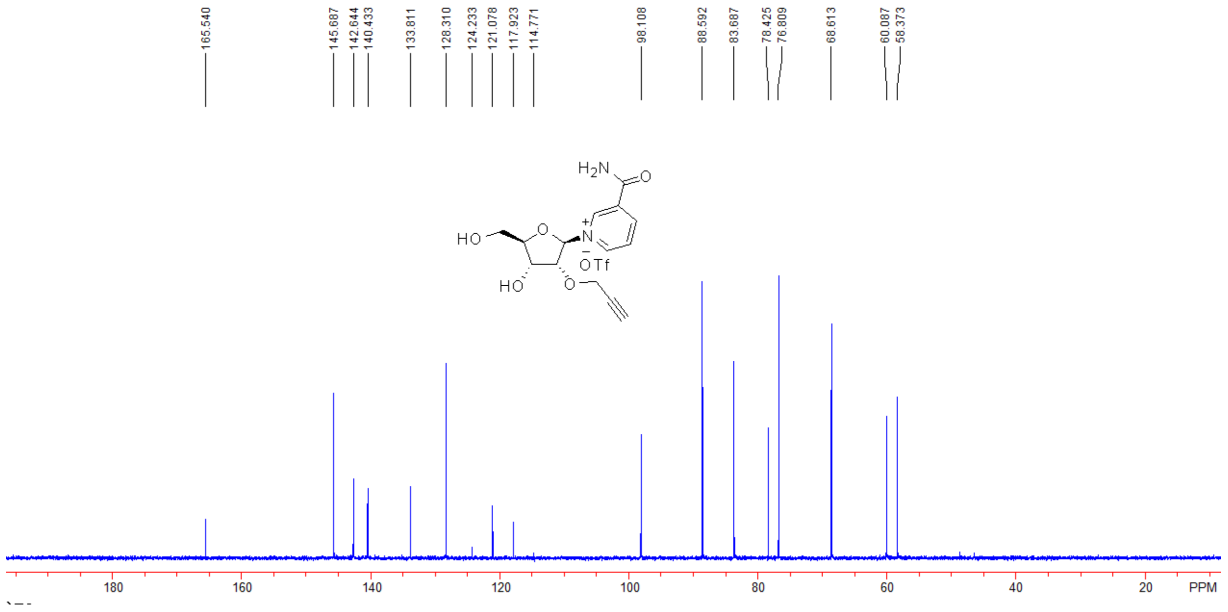

**Supplementary Figure 52**. $^{13}$C NMR spectrum of compound NR1.

To a stirred solution of compound NR1 (119 mg, 0.27 mmol) in trimethylphosphate (2 mL) was added P(O)$Cl_3$ (175 μL, 1.89 mmol, 7 eq) at 0°C and the resulting mixture was stirred at 0°C for 6 hours. A few drops of $H_2O$ were then added to quench the reaction. Trimethylphosphate was removed by extraction with methylene chloride (3×20 ml). The aqueous layer was concentrated *in vacuo* and the crude product was purified via preparative HPLC (C18-A column, 150×10.0 mm, 5 μm) (mobile phase A: 0.1% formic acid (aq), mobile B: 0.1% formic acid in acetonitrile; flow rate = 2.0 ml min$^{-1}$; 0-2 min: 0-4% B, 2-4 min: 4-10% B, 4-6 min: 10-20% B,

6-12 min: 20-50% B, 12-14 min: 50-0% B. Fractions containing the desired product were concentrated and lyophilized to yield the NMN1 (63 mg, 63% yield) as a colorless solid. $^1$H NMR (400 MHz, $D_2O$): δ 2.80 (t, 1H, $J$ = 2.4 Hz, CH), 4.11-4.16 (m, 1H, $CH_2$), 4.27-4.36 (m, 2H, $CH_2$+$CH_2$), 4.44 (dd, 1H, $J$ = 16.0, 2.4 Hz, $CH_2$), 4.60 (t, 1H, $J$ = 4.8 Hz, CH), 4.65-4.68 (m, 2H, 2CH), 6.32 (d, 1H, $J$ = 5.2 Hz, CH), 8.30 (dd, 1H, $J$ = 8.0, 6.4 Hz, ArH), 8.99 (d, 1H, $J$ = 8.0 Hz, ArH), 9.31 (d, 1H, $J$ = 6.4 Hz, ArH), 9.48 (s, 1H, ArH) (Supplementary Figure 53); $^{13}$C NMR (100 MHz, $D_2O$): δ 58.4, 63.9 (d, $J$ = 4.8 Hz), 69.4, 76.8, 78.5, 84.0, 88.2 (d, $J$ = 8.6 Hz), 98.3, 128.5, 133.8, 140.0, 142.6, 146.1, 165.6 (Supplementary Figure 54).

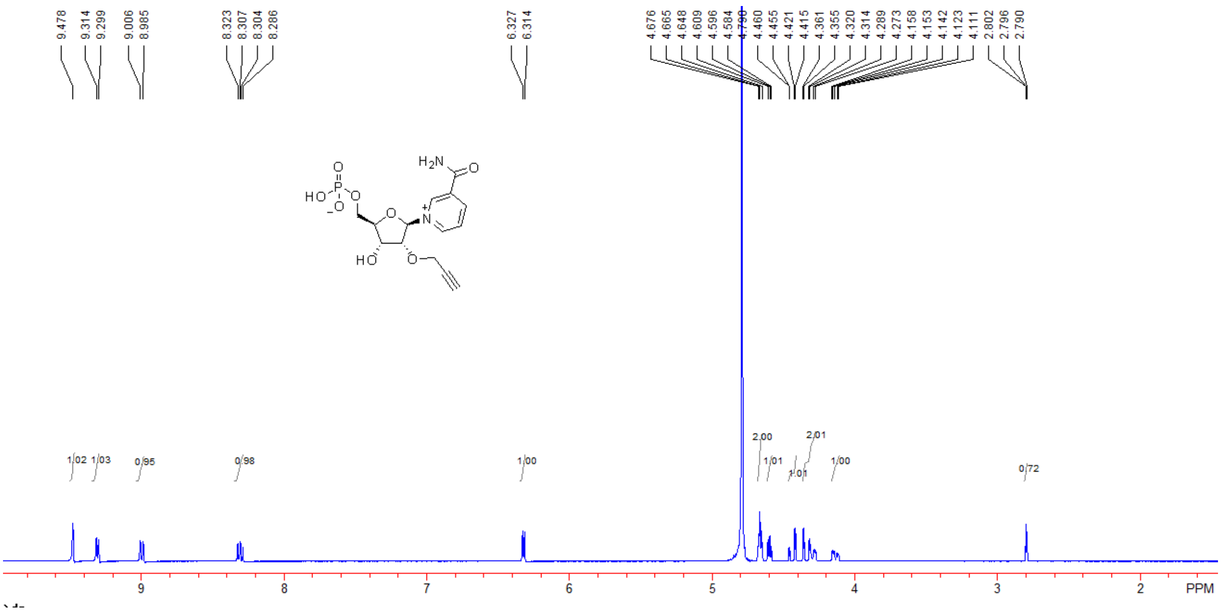

**Supplementary Figure 53**. $^1$H NMR spectrum of compound NMN1.

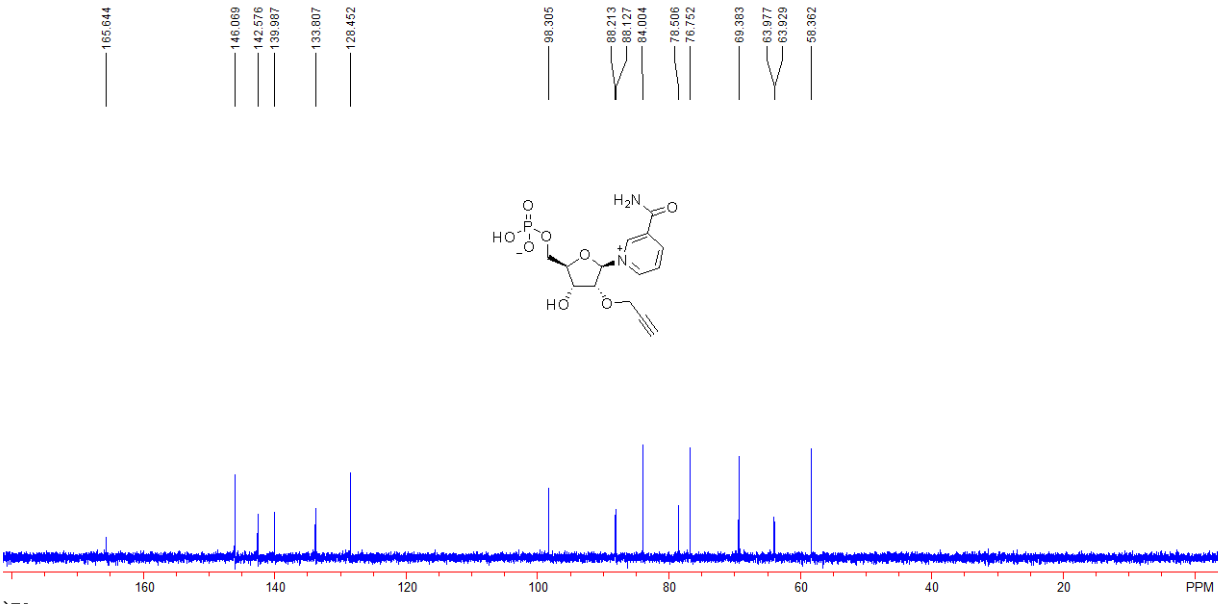

**Supplementary Figure 54**. $^{13}C$ NMR spectrum of compound NMN1.

To a stirred solution of adenosine 5′-monophosphate (5′-AMP) (52 mg, 0.15 mmol, 1.5 eq) in dried DMF (2 mL) were added 1,1-carbonyldiimidazole (CDI) (63 mg, 0.50 mmol, 5 eq) and triethylamine (23 µL, 0.16 mmol. 1.6 eq). The reaction mixture was stirred at room temperature for 14 hours, and then quenched with 0.100 ml dried methanol. The solvent was removed under vacuum and the residue was co-evaporated 3 times each with 1.00 ml of dried DMF. The activated 5′-AMP was dissolved in dried DMF (1 mL) and compound (NMN1) (37 mg, 0.10 mmol, 1.0 eq) was added. After stirring at room temperature for 4 days, $H_2O$ was added to quench the reaction at 0°C. The resulting mixture was continued stirring at room temperature for 24 hours. The reaction was then concentrated *in vacuo* and the crude product was purified via preparative HPLC (C18-A column, 150×10.0 mm, 5 µm) (mobile phase A: 0.1% formic acid (aq), mobile B: 0.1% formic acid in acetonitrile; flow rate = 1.0 ml min$^{-1}$; 0-16 min: 0-6.7% B, 16-18 min: 6.7-0% B). Fractions containing the desired product were concentrated and lyophilized to yield the **1** (29 mg, 42% yield) as a colorless solid. $^{1}H$ NMR (400 MHz, $D_2O$): δ 2.77 (t, 1H, $J$ = 2.4 Hz, CH), 4.18-4.27 (m, 3H, 2$CH_2$+$CH_2$), 4.30 (dd, 1H, $J$ = 16.0, 2.4 Hz, $CH_2$), 4.35-4.43 (m, 3H, $CH_2$+CH), 4.51 (t, 1H, $J$ = 4.0 Hz, CH), 4.55 (t, 1H, $J$ = 5.2 Hz, CH), 4.58-4.61 (m, 1H, CH), 4.63-4.66 (m, 1H, CH), 4.75 (t, 1H, $J$ = 5.2 Hz, CH), 6.13 (d, 1H, $J$ = 5.6 Hz, CH), 6.26 (d, 1H, $J$ = 5.6 Hz, CH), 8.28 (dd, 1H, $J$ = 8.0, 6.4 HZ, ArH), 8.39 (s, 1H, ArH), 8.60 (s, 1H, ArH), 8.94 (d, 1H, $J$ = 8.0 Hz, ArH), 9.28 (d, 1H, $J$ = 6.4 Hz, ArH), 9.43 (s,

1H, ArH) (Supplementary Figure 55); $^{13}C$ NMR (100 MHz, $D_2O$): δ 58.4, 64.63-64.69 (m), 65.00-65.04 (m), 69.0, 70.2, 74.4, 76.7, 78.5, 83.9, 84.00-84.08 (m), 87.66, 87.73, 98.3, 128.6, 133.7, 140.1, 142.6, 145.4, 146.1, 148.4, 150.4, 165.4 (Supplementary Figure 56); HRMS (ESI) for $C_{24}H_{28}N_7Na_2O_{14}P_2^{+1}$ (M+2Na-H)$^+$: Calcd.: 746.0965 Da; Obs: 746.0978 Da.

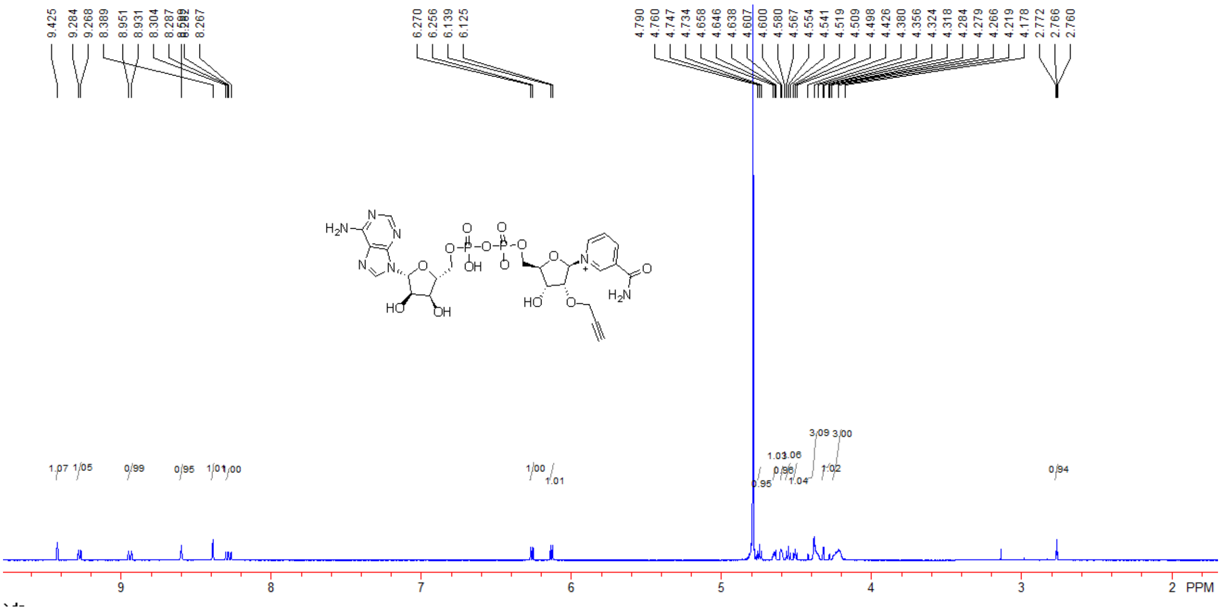

**Supplementary Figure 55**. $^1H$ NMR spectrum of compound **1**.

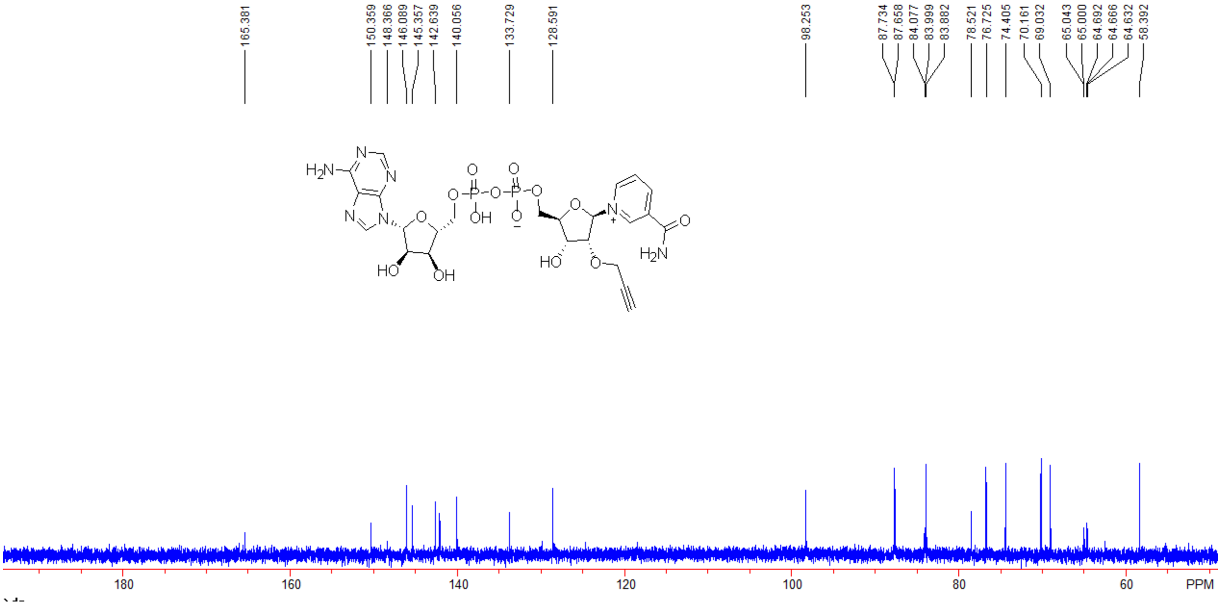

**Supplementary Figure 56**. $^{13}C$ NMR spectrum of compound **1**.

**Synthesis of $NAD^+$ analogue 2 (Supplementary Figure 4).**

To a stirred solution of methyl-β-D-Ribofuranoside (**SM-1**) (1.64 g, 10.0 mmol) and imidazole (1.36 g, 20.0 mmol, 2.0 eq) in anhydrous DMF (20 mL) was added TBDPSCl (3.02g, 11.0 mmol, 1.1 eq) at 0°C. Then, the reaction mixture was allowed to warm to room temperature. After stirring at this temperature for 24 hours, the reaction mixture was diluted with EtOAc (100 mL), and the organic phase was washed successively with ice-water (50 mL), aq 1M HCl (2×50 mL), $H_2O$ (2×50 mL), dried over anhydrous $Na_2SO_4$, filtered and concentrated to give a residue. The residue was purified by a flash column chromatography on silica gel to afford the compound (**2-2**) (3.62 g, 90%) as a colorless oil. $^1H$ NMR (400 MHz, $CDCl_3$): δ 1.07 (s, 9H, $3CH_3$), 2.30 (d, 1H, $J$ = 5.6 Hz, OH), 2.62 (d, 1H, $J$ = 3.2 Hz, OH), 3.31 (s, 3H, $OCH_3$), 3.77 (dd, 1H, $J$ = 10.8, 6.0 Hz, $CH_2$), 3.83 (dd, 1H, $J$ = 10.8, 4.4 Hz, $CH_2$), 4.01-4.05 (m, 2H, 2CH), 4.32-4.36 (m, 1H, CH), 4.84 (s, 1H, CH), 7.37-7.46 (m, 6H, ArH), 7.68-7.70 (m, 4H, ArH) (Supplementary Figure 57); $^{13}C$ NMR (100 MHz, $CDCl_3$): δ 19.2, 26.8, 55.2, 65.1, 72.7, 75.3, 82.9, 108.1, 127.75, 127.78, 129.80, 129.83, 133.2, 135.6 (Supplementary Figure 58).

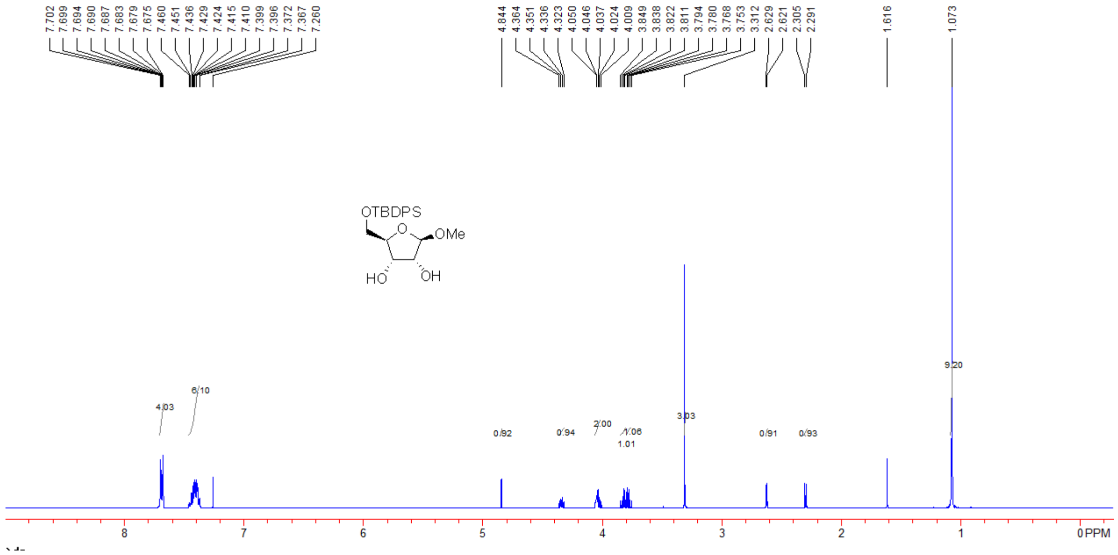


**Supplementary Figure 57**. $^1H$ NMR spectrum of compound **2-2**.

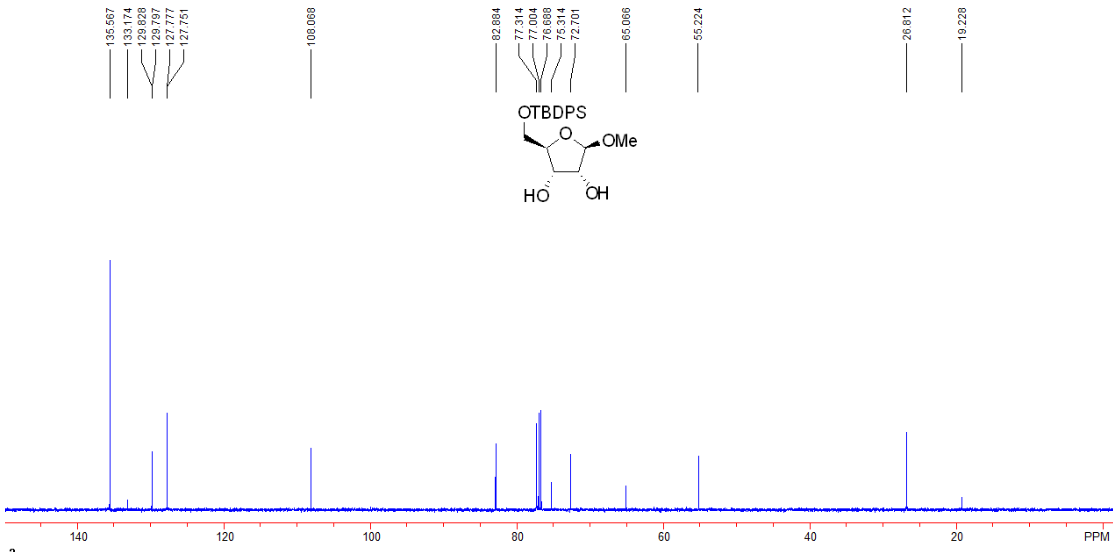


**Supplementary Figure 58**. $^{13}C$ NMR spectrum of compound **2-2**.

To a stirred solution of compound **2-2** (3.62 g, 9.0 mmol) in anhydrous THF (30 mL) was added NaH (432 mg, 10.8 mmol, 1.2 eq, 60% dispersion in mineral oil) at 0$^{o}$C followed by the addition of propargyl bromide (1.61 g, 13.5 mmol, 1.5 eq) at the same temperature. Then the reaction mixture was allowed to warm to room temperature. After stirring at this temperature for 6 hours, the reaction mixture was quenched with saturated aqueous $NH_4Cl$ (20 mL) and extracted with EtOAc (3×50 mL). The combined organic layers were washed water (3×50 mL), dried over anhydrous $Na_2SO_4$, filtered and concentrated and purified by a flash column chromatography on silica gel to afford the compound (**2-3**) (1.67 g, 42%) as a colorless oil. $^1H$ NMR (400 MHz, $CDCl_3$): δ 1.07 (s, 9H, $3CH_3$), 2.45 (t, 1H, $J$ = 2.4 Hz, CH), 2.59 (d, 1H, $J$ = 3.2 Hz, OH), 3.31 (s, 3H, $OCH_3$), 3.75 (dd, 1H, $J$ = 10.8, 4.4 Hz, $CH_2$), 3.80 (dd, 1H, $J$ = 10.8, 5.2 Hz, $CH_2$), 4.09-4.13 (m, 1H, CH), 4.14-4.19 (m, 2H, CH+$CH_2$), 4.24-4.29 (m, 2H, CH+$CH_2$), 4.87 (s, 1H, CH), 7.36-7.45 (m, 6H, ArH), 7.68-7.71 (m, 4H, ArH) (Supplementary Figure 59); $^{13}C$ NMR (100 MHz, $CDCl_3$): δ 19.4, 26.9, 55.3, 58.3, 64.6, 73.8, 75.6, 79.3, 79.7, 81.8, 108.4, 127.85, 127.87, 129.87, 129.91, 133.4, 135.76, 135.79 (Supplementary Figure 60).

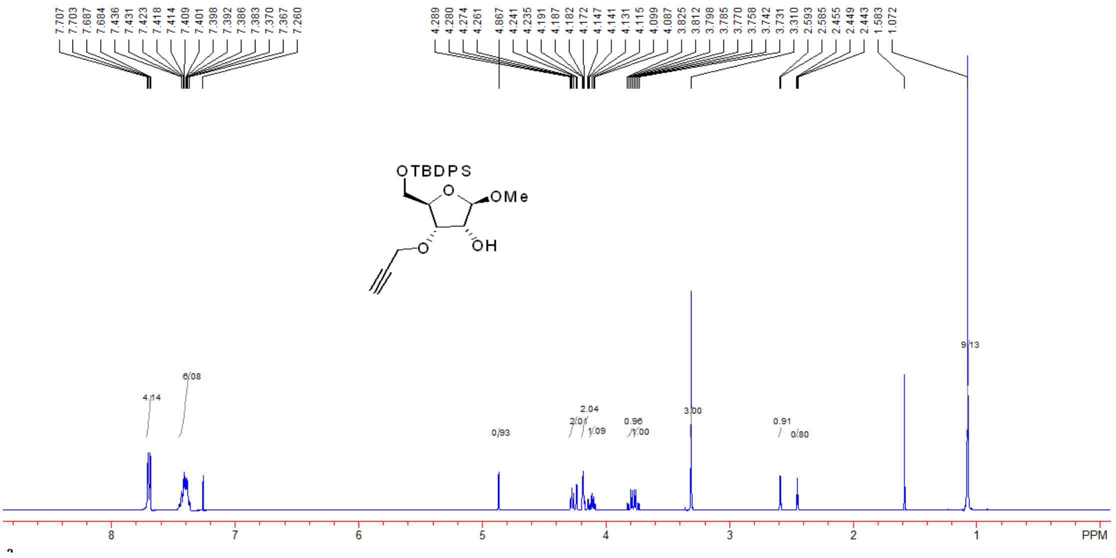

**Supplementary Figure 59**. $^{1}$H NMR spectrum of compound **2-3**.

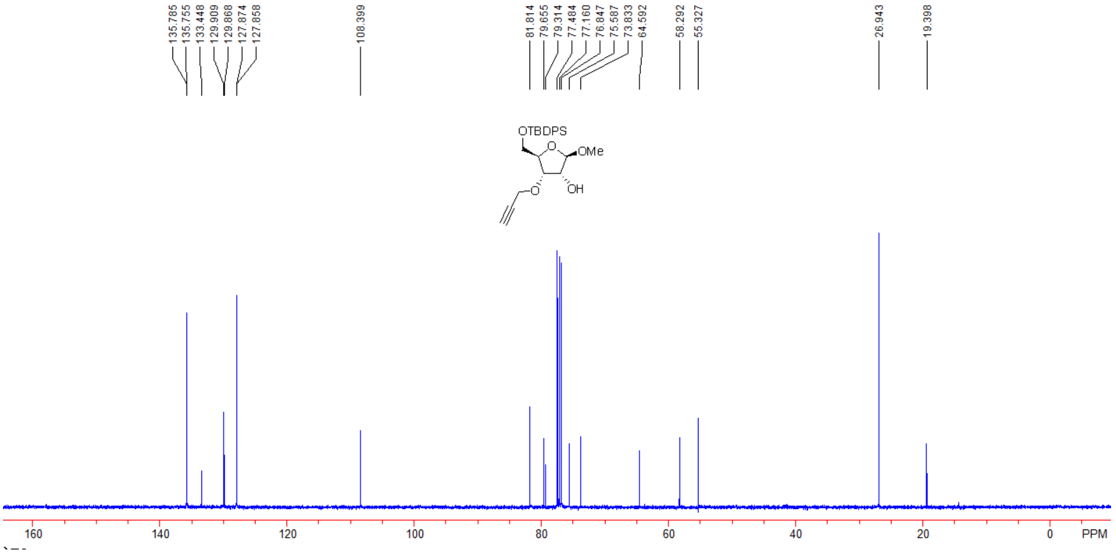

**Supplementary Figure 60**. $^{13}$C NMR spectrum of compound **2-3**.

To a 0°C solution of compound **2-3** (1.10 g, 2.5 mmol) in anhydrous THF (25 mL) was added AcOH (225 mg, 3.75 mmol, 1.5 eq) followed by the addition of TBAF (3.75 mL, 3.75 mmol, 1.0 M in THF, 1.5 eq). Then the reaction mixture was allowed to warm to room temperature. After stirring at this temperature for 14 hours, the reaction mixture was concentrated under reduced pressure to give a residue. The residue was purified by a flash column chromatography on silica gel to afford the compound **2-4** (430 mg, 85%) as a colorless oil. $^1$H NMR (400 MHz, $CDCl_3$): δ 2.53 (t, 1H, $J$ = 2.4 Hz, CH), 3.40 (s, 3H, $OCH_3$), 3.63 (dd, 1H, $J$ = 12.0, 4.0 Hz, $CH_2$), 3.81 (dd, 1H, $J$ = 12.0 3.2 Hz, $CH_2$), 4.14-4.19 (m, 2H, 2CH), 4.19-4.30 (m, 3H,

CH+CH2), 4.87 (s, 1H, CH) (Supplementary Figure 61); $^{13}$C NMR (100 MHz, $CDCl_3$): δ 55.7, 58.4, 62.7, 73.6, 75.7, 78.4, 79.0,82.3, 108.8 (Supplementary Figure 62).

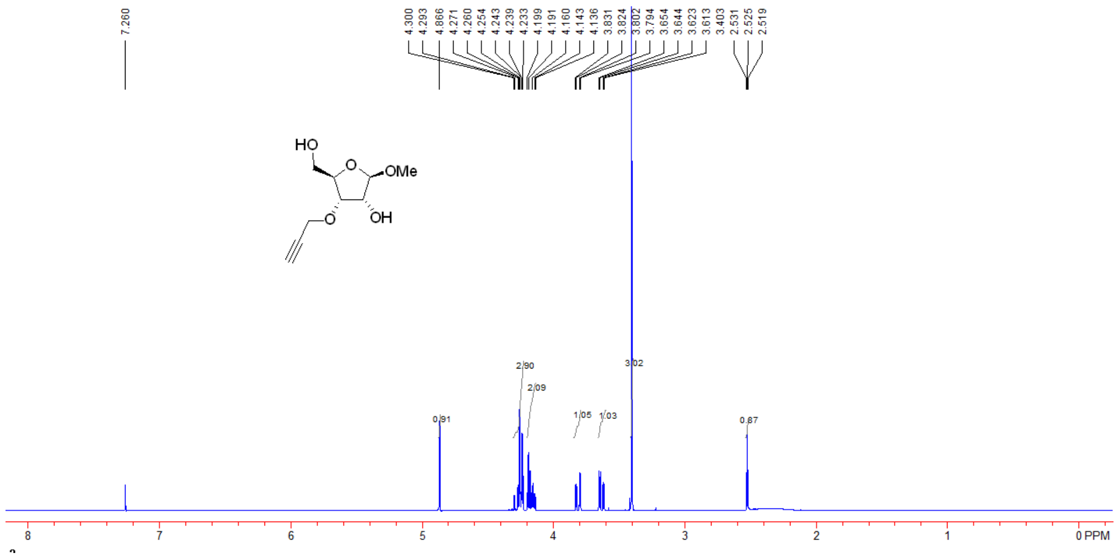

**Supplementary Figure 61**. $^{1}$H NMR spectrum of compound **2-4**.

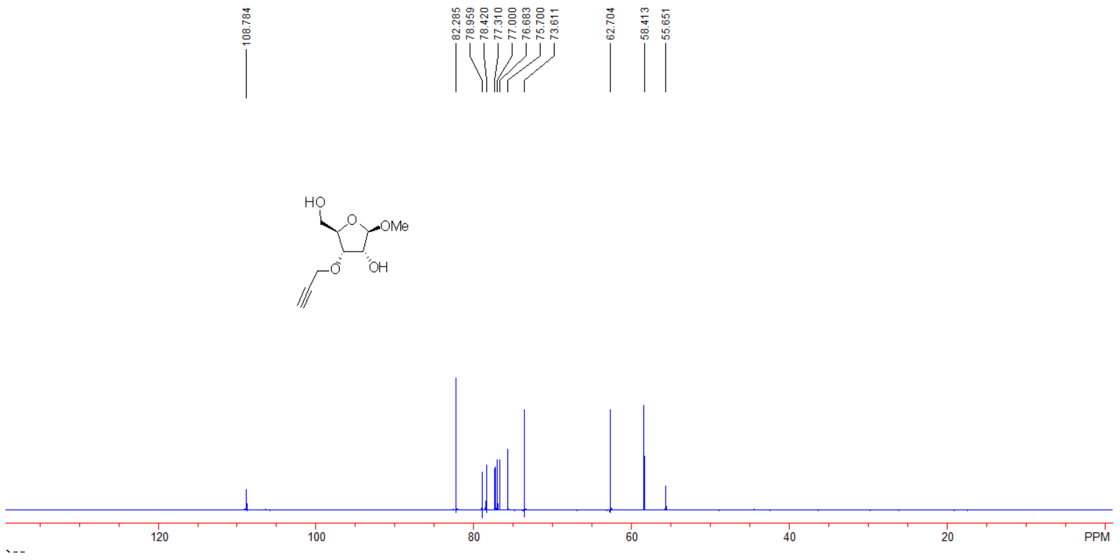

**Supplementary Figure 62**. $^{13}$C NMR spectrum of compound **2-4**.

To a solution of **2-4** (404 mg, 2.0 mmol) in a mixture of anhydrous DCM (10 mL) and anhydrous pyridine (10 mL) was added BzCl (691 µL, 6.0 mmol, 3 eq) at 0°C. Then the reaction mixture was allowed to warm to room temperature. After stirring for 24 hours, the reaction was quenched with MeOH (10 mL) and the mixture was concentrated under reduced pressure to give a residue. The residue was dissolved in EtOAc (50 mL), and the organic phase was washed

successively with saturated aqueous $CuSO_4$ (3×50 mL), brine (50 mL), dried over anhydrous $Na_2SO_4$, filtered, concentrated and purified by a flash column chromatography on silica gel to afford the desired product **2-5** (591 mg, 72%) as a colorless oil. $^1$H NMR (400 MHz, $CDCl_3$): δ 2.35 (t, 1H, $J$ = 2.4 Hz, CH), 3.36 (s, 3H, $OCH_3$), 4.22-4.23 (m, 2H, $CH_2$), 4.42-4.48 (m, 2H, CH+$CH_2$), 4.62-4.69 (m, 2H, CH+$CH_2$), 5.05 (s, 1H, CH), 5.49 (d, 1H, $J$ = 4.4 Hz, CH), 7.44-7.48 (m, 4H, ArH), 7.56-7.61 (m, 2H, ArH), 8.07-8.13 (m, 4H, ArH) (Supplementary Figure 63); $^{13}$C NMR (100 MHz, $CDCl_3$): δ 55.1, 58.3, 64.2, 73.9, 75.3, 76.8, 128.3, 128.4, 129.4, 129.7, 129.87, 129.93, 133.1, 133.4, 165.6, 166.39 (Supplementary Figure 64).

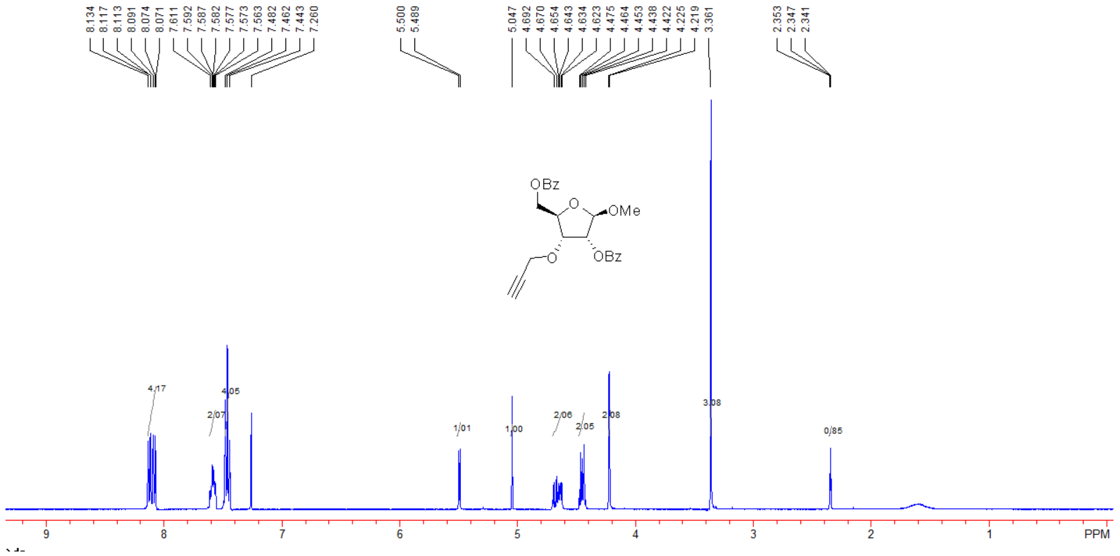


**Supplementary Figure 63**. $^1$H NMR spectrum of compound **2-5**.

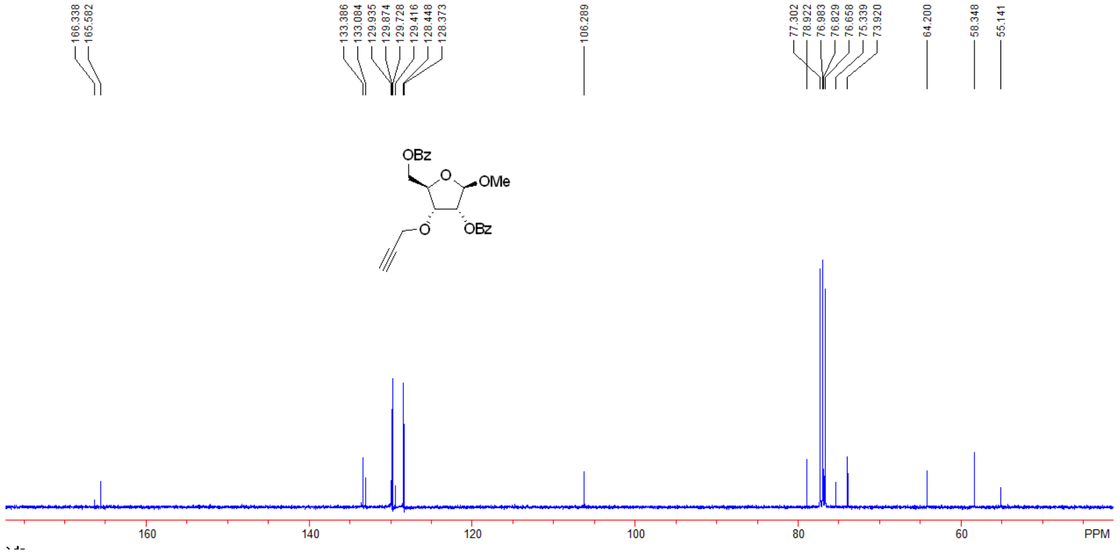


**Supplementary Figure 64**. $^{13}$C NMR spectrum of compound **2-5**.

Compound **2-5** (533 mg, 1.3 mmol) was dissolved in a mixture of TFA/$H_2O$ (9/1, 15 mL) and the resulting mixture was stirred at room temperature until the reaction complete (monitoring by TLC). Then the reaction was diluted with DCM (60 mL) and the solution was added dropwise to a stirred mixture of ice and saturated aqueous $NaHCO_3$. Solid $NaHCO_3$ was added during the addition to maintain a pH of 7. The mixture was extracted with DCM (3×50 mL), and the combined organic extracts was washed with $H_2O$ (50 mL), brine (50 mL), dried over anhydrous $Na_2SO_4$, filtered, concentrated to give a residue. The residue was dissolved in pyridine (15 mL) and cooled to 0°C. $Ac_2O$ (0.5 mL) was added dropwise and then the resulting mixture was allowed to warm to room temperature. After stirring for 6 hours, the reaction was quenched with MeOH (10 mL) and the mixture was concentrated under reduced pressure to give a residue. The residue was dissolved in EtOAc (50 mL), and the organic phase was washed successively with saturated aqueous $CuSO_4$ (3×50 mL), brine (50 mL), dried over anhydrous $Na_2SO_4$, filtered, concentrated and purified by a flash column chromatography on silica gel to afford the desired product **2-6** (456 mg, 80%) as a colorless oil. $^1$H NMR (400 MHz, $CDCl_3$) of one isomer: δ 1.95 (s, 3H, $CH_3$), 2.38 (t, 1H, $J$ = 2.4 Hz, CH), 4.25 (d, 2H, $J$ = 2.4 Hz, $CH_2$), 4.43-4.49 (m, 2H, $CH_2$), 4.66 (dd, 1H, $J$ = 8.0, 4.4 Hz, CH), 4.72-4.76 (m, 1H, CH), 5.56 (d, 1H, $J$ = 4.4 Hz, CH), 6.32 (s, 1H, CH), 7.43-7.48 (m, 4H, ArH), 7.56-7.60 (m, 2H, ArH), 8.06-8.12 (m, 4H, ArH) (Supplementary Figure 65); $^{13}$C NMR (100 MHz, $CDCl_3$): δ 20.9, 58.5, 63.1, 73.6, 75.6, 75.7, 78.8, 79.9, 98.5, 128.4, 128.5, 129.1, 129.7, 129.85, 129.93, 133.2, 133.6, 165.4, 166.0, 169.0 (Supplementary Figure 66).

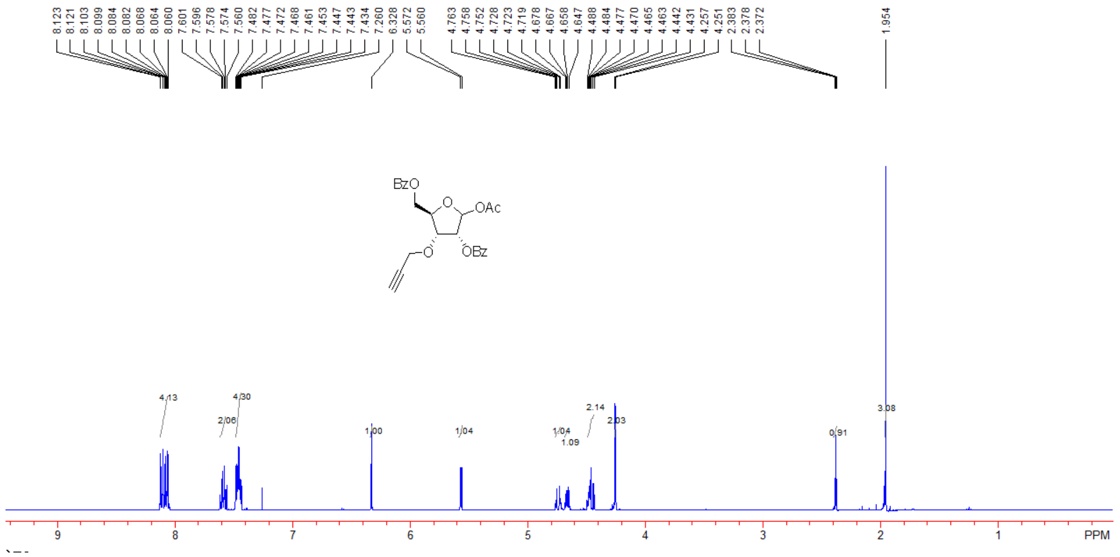

**Supplementary Figure 65**. $^{1}$H NMR spectrum of compound **2-6**.

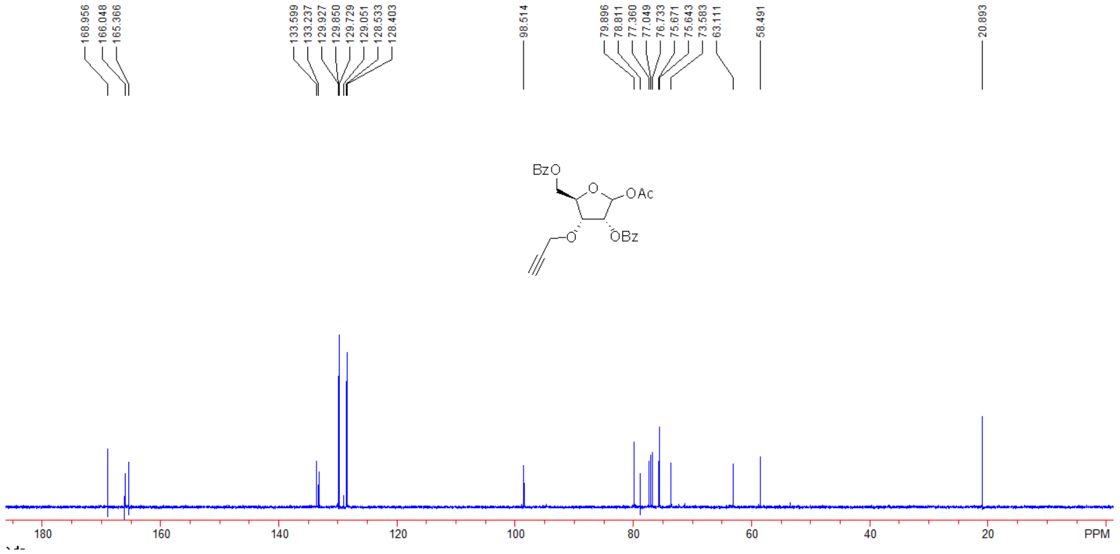

**Supplementary Figure 66**. $^{13}$C NMR spectrum of compound **2-6**.

Compound **2-6** (307 mg, 0.70 mmol) was dissolved in toluene (10 mL) and cooled to 0°C. HBr (33% (wt) in acetic acid) (257 mg, 1.05 mmol, 1.5 eq) was added dropwise and the reaction was stirred at 0°C for 5 hours. After the starting material was consumed, the reaction was concentrated under reduced pressure to give a residue. The residue was azeotroped with toluene (3×20 mL) to remove remaining acetic acid and dried *in vacuo*. The crude product and nicotinamide (103 mg, 0.84 mmol, 1.2 eq) was dissolved in $CH_3CN$ (20 mL). The reaction was stirred under Ar gas at room temperature for 24 hours. The reaction was concentrated *in vacuo* (the temperature was kept below 35°C) and purified by a flash column chromatography on silica

gel to afford the desired product **2-7** (260 mg, 64%) as a colorless solid. $^1$H NMR (400 MHz, $CDCl_3$): δ 2.34 (t, 1H, $J$ = 2.8 Hz, CH), 4.35 (dd, 1H, $J$ = 16.0, 2.0 Hz, $CH_2$), 4.43 (dd, 1H, $J$ = 16.0, 2.0 Hz, $CH_2$), 4.80-4.84 (m, 1H, CH), 4.91 (d, 2H, $J$ = 2.8 Hz, $CH_2$), 5.13 (dd, 1H, $J$ = 8.4, 4.8 Hz, CH), 6.33 (d, 1H, $J$ = 5.6 Hz, CH), 6.50 (br, 1H, NH), 6.70 (s, 1H, CH), 7.44-7.52 (m, 4H, ArH), 7.59-7.63 (m, 2H, ArH), 7.93-7.97 (m, 1H, ArH), 8.07-8.13 (m, 4H, ArH), 9.08 (d, 1H, $J$ = 7.2 Hz, ArH), 9.16 (br, 1H, NH), 9.25 (d, 1H, J = 6.0 Hz, ArH), 10.07 (s, 1H, ArH) (Supplementary Figure 67); $^{13}$C NMR (100 MHz, $CDCl_3$): δ 59.2, 62.7, 74.4, 75.8, 76.3, 79.2, 82.5, 98.5, 128.4, 128.6, 128.8, 128.9, 129.3, 130.0, 130.3, 133.9, 134.2, 134.6, 141.5, 142.0, 146.8, 163.6, 166.3, 171.3 (Supplementary Figure 68).

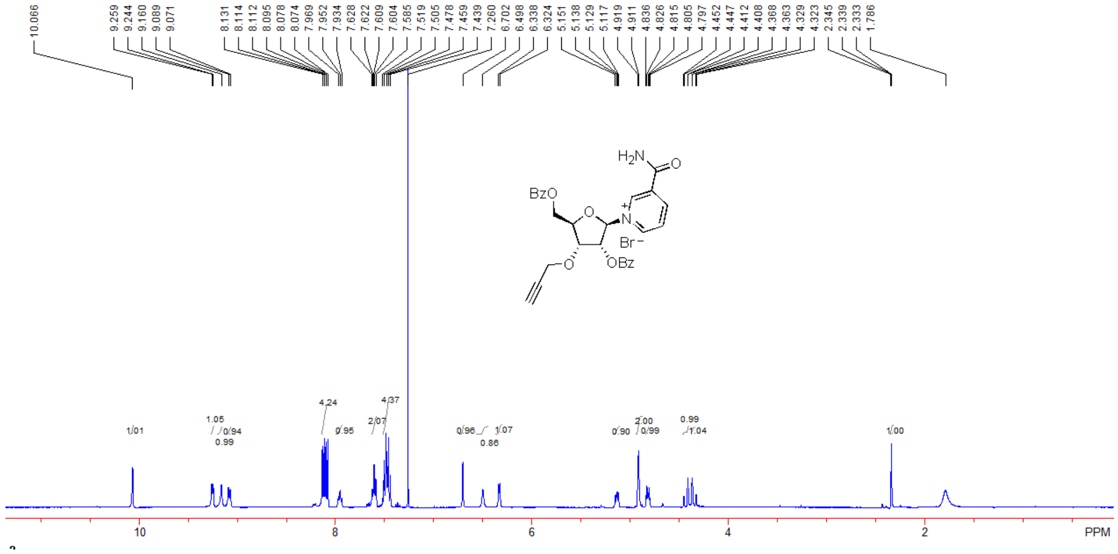


**Supplementary Figure 67**. $^1$H NMR spectrum of compound **2-7**.

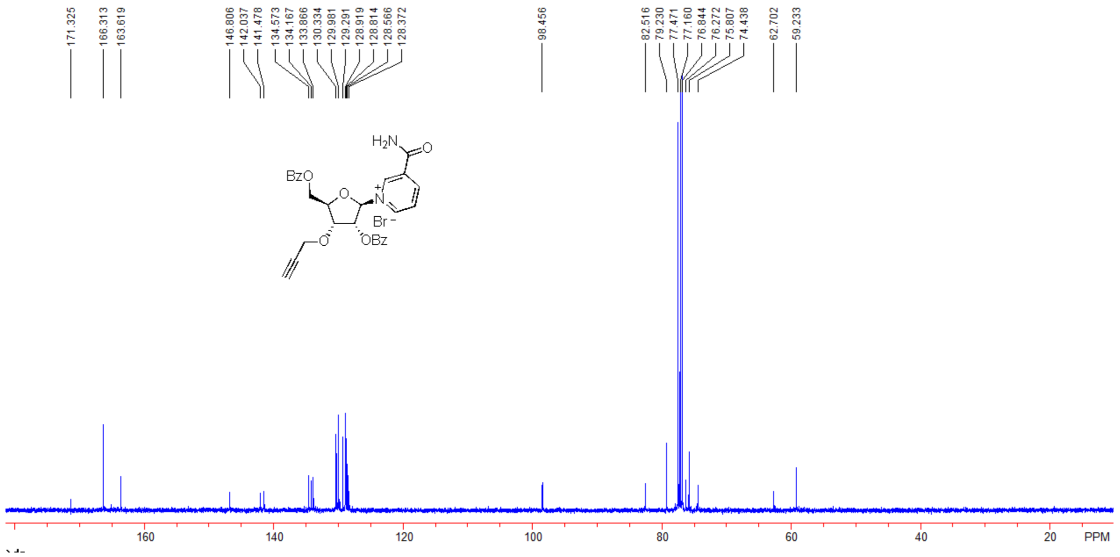


**Supplementary Figure 68**. $^{13}$C NMR spectrum of compound **2-7**.

Compound **2-7** (233 mg, 0.40 mmol) or was dissolved in ammonia (20 mL, 7 N in MeOH) and the reaction was stirred at 0°C for 48 hours. The reaction was concentrated under reduced pressure and the crude product was dissolved in MeOH (0.5 mL). Addition of ethyl ether (10 mL) resulted in ppt of the desired product. The procedure was repeated three times to yield the desired product NR2 (97 mg, 65%) as a colorless oil**.** $^{1}$H NMR (400 MHz, $D_2O$): δ 2.91 (t, 1H, $J$ = 2.4 Hz, CH), 4.15 (ddd, 1H, $J$ = 12.0, 5.2, 2.0 Hz, $CH_2$), 4.31 (ddd, 1H, $J$ = 12.0, 4.4, 2.4 Hz, $CH_2$), 4.10-4.34 (m, 3H, CH+$CH_2$), 4.66 (t, 1H, $J$ = 5.2 Hz, CH), 6.19 (d, 1H, $J$ = 5.6 Hz, CH), 8.28 (dd, 1H, $J$ = 8.0, 6.0 Hz, ArH), 8.97 (dt, 1H, $J$ = 8.0, 1.6 Hz, ArH), 9.26 (d, 1H, J = 6.0 Hz, ArH), 9.44 (s, 1H, ArH) (Supplementary Figure 69); $^{13}$C NMR (100 MHz, $D_2O$): δ 57.96, 57.99, 60.0, 76.1, 76.4, 76.5, 85.8, 99.9, 128.3, 133.9, 140.3, 142.6, 145.7, 165.7 (Supplementary Figure 70).

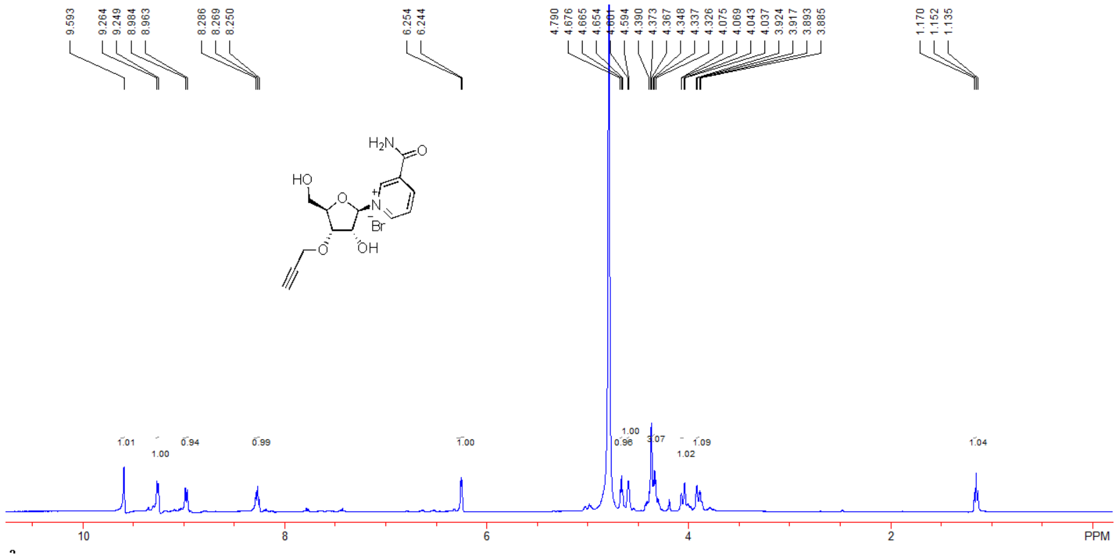


**Supplementary Figure 69**. $^{1}$H NMR spectrum of compound NR2.

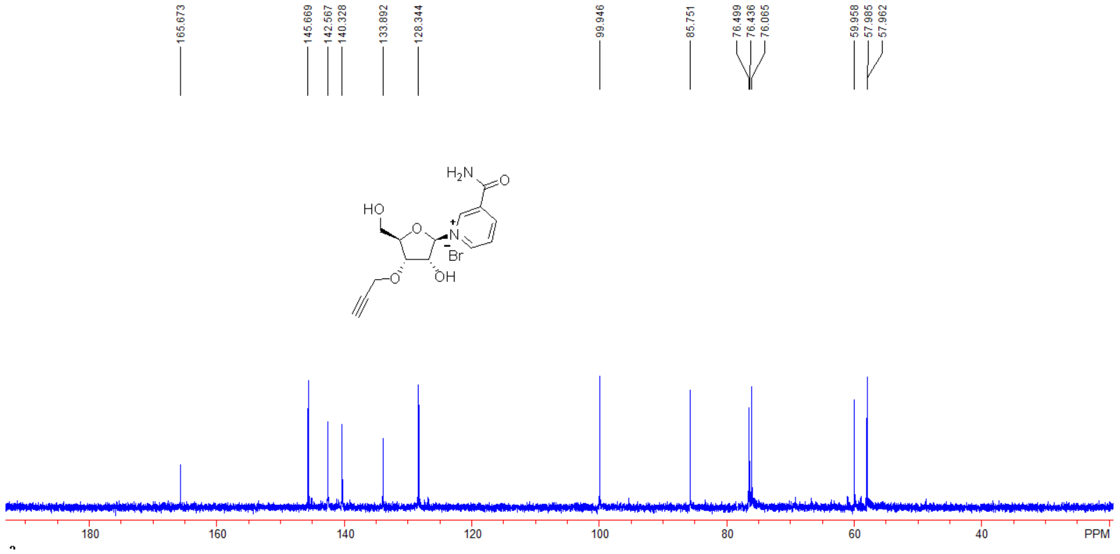


**Supplementary Figure 70**. $^{13}$C NMR spectrum of compound NR2.

To a stirred solution of compound NR2 (82 mg, 0.22 mmol) in trimethylphosphate (2 mL) was added $P(O)Cl_3$ (143 µL, 1.54 mmol, 7 eq) at 0$^{o}$C and the resulting mixture was stirred at 0$^{o}$C for 6 hours. A few drops of $H_2O$ were then added to quench the reaction. Trimethylphosphate was removed by extraction with methylene chloride (3×20 ml). The remaining trimethylphosphate was removed by a second extraction with THF (5 ml). The aqueous layer was concentrated *in vacuo* and the crude product was dissolved in MeOH (0.5 mL). Addition of ethyl ether (10 mL) resulted in ppt of the desired product. The procedure was repeated three times to yield the desired product NMN2 (57 mg, 69%) as a colorless oil. $^{1}$H NMR (400 MHz,

$D_2O$): δ 2.91 (t, 1H, *J* = 2.4 Hz, CH), 4.15 (ddd, 1H, *J* = 12.0, 5.2, 2.0 Hz, $CH_2$), 4.30 (ddd, 1H, *J* = 12.0, 6.4, 2.4 Hz, $CH_2$), 4.37-4.46 (m, 3H, CH+$CH_2$), 4.66 (t, 1H, *J* = 5.2 Hz, CH), 4.79-4.82 (1H, CH, overlap with the solvent residue peak), 6.20 (d, 1H, *J* = 5.6 Hz, CH), 8.28 (dd, 1H, *J* = 8.0, 6.0 Hz, ArH), 8.97 (dt, 1H, *J* = 8.0, 1.2 Hz, ArH), 9.26 (d, 1H, *J* = 6.0 Hz, ArH), 9.44 (s, 1H, ArH) (Supplementary Figure 71); $^{13}C$ NMR (100 MHz, $D_2O$): δ 58.1, 64.2 (d, *J* = 4.9 Hz), 76.5, 76.7, 77.8, 78.9, 85.2 (d, *J* = 9.3 Hz), 99.8, 128.5, 133.9, 139.8, 142.4, 146.0, 165.7 (Supplementary Figure 72).

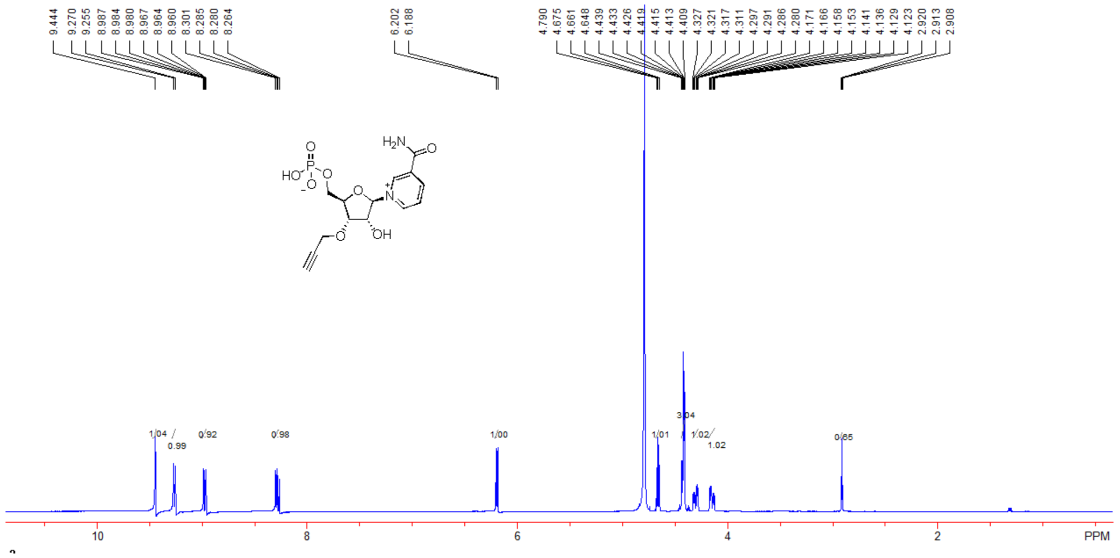


**Supplementary Figure 71**. $^1H$ NMR spectrum of compound NMN2.

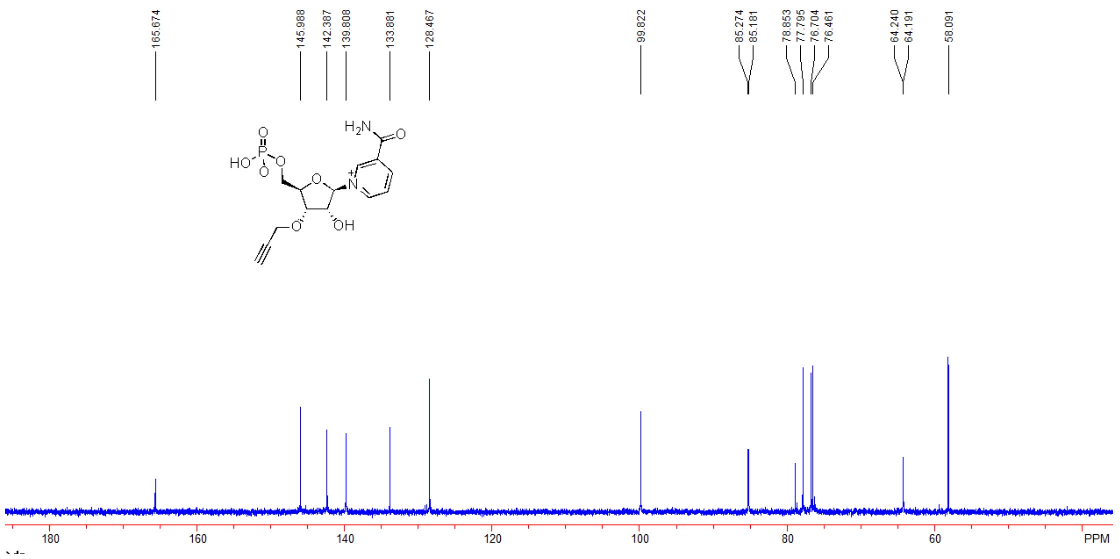


**Supplementary Figure 72**. $^{13}C$ NMR spectrum of compound NMN2.

To a stirred solution of adenosine 5′-monophosphate (5′-AMP) (52 mg, 0.15 mmol, 1.5 eq) in dried DMF (2 mL) were added 1,1-carbonyldiimidazole (CDI) (63 mg, 0.50 mmol, 5 eq) and triethylamine (23 μL, 0.16 mmol. 1.6 eq). The reaction mixture was stirred at room temperature for 8 hours, and then quenched with 0.20 ml dried methanol. The solvent was then removed under vacuum and the residue was coevaporated 3 times each with 1.00 ml of dried DMF. The activated 5′-AMP was dissolved in dried DMF (1 mL) and compound (NMN2) (37 mg, 0.10 mmol) was added. After stirring at room temperature for 4 days, $H_2O$ (5 mL) was added to quench the reaction at 0°C. The resulting mixture was continued stirring at room temperature for 24 hours. The reaction was then concentrated *in vacuo* and the crude product was purified via preparative HPLC (C18-A column, 150×10.0 mm, 5 μm) (mobile phase A: 0.1% formic acid (aq), mobile B: 0.1% formic acid in acetonitrile; flow rate = 2.0 ml $min^{-1}$; 0-2 min: 0-4% B, 2-4 min: 4-10% B, 4-6 min: 10-20% B, 6-12 min: 20-50%, 12-14 min: 50-0% B). Fractions containing the desired product were concentrated and lyophilized to yield the $NAD^+$ analogue **2** (32 mg, 45% yield) as a colorless solid. $^1H$ NMR (400 MHz, $D_2O$): δ 2.92 (t, 1H, *J* = 2.4 Hz, CH), 4.20-4.29 (m, 3H, $CH_2$), 4.37-4.45 (m, 5H, $CH_2$+CH), 4.52 (t, 1H, *J* = 4.8 Hz, CH), 4.68 (t, 1H, *J* = 5.6 Hz, CH), 4.74 (t, 1H, *J* = 5.2 Hz, CH), 4.77-4.79 (m, 1H, CH), 6.14 (d, 1H, *J* = 5.2 Hz, CH), 6.17 (d, 1H, 5.6 Hz, CH), 8.30 (dd, 1H, *J* = 8.0 Hz, ArH), 8.42 (s, 1H, ArH), 8.62 (s, 1H, ArH), 8.95 (d, 1H, *J* = 8.0 Hz, ArH), 9.27 (d, 1H, *J* = 6.0 Hz, ArH), 9.43 (s, 1H, ArH) (Supplementary Figure 73); $^{13}C$ NMR (100 MHz, $D_2O$): δ 58.1, 64.85-64.88 (m), 64.97-65.02 (m), 70.1, 74.5, 76.4, 76.7, 78.0, 78.9, 84.0 (d, *J* = 8.8 Hz), 85.3 (d, *J* = 8.8 Hz), 87.8, 99.8, 118.4, 128.6, 133.8, 139.8, 142.3, 142.5, 144.8, 146.1, 148.3, 149.9, 165.5 (Supplementary Figure 74); HRMS (ESI) for $C_{24}H_{28}N_7Na_2O_{14}P_2^{+1}$ (M+2Na-H)$^+$: Calcd.: 746.0965 Da; Obs: 746.0955 Da.

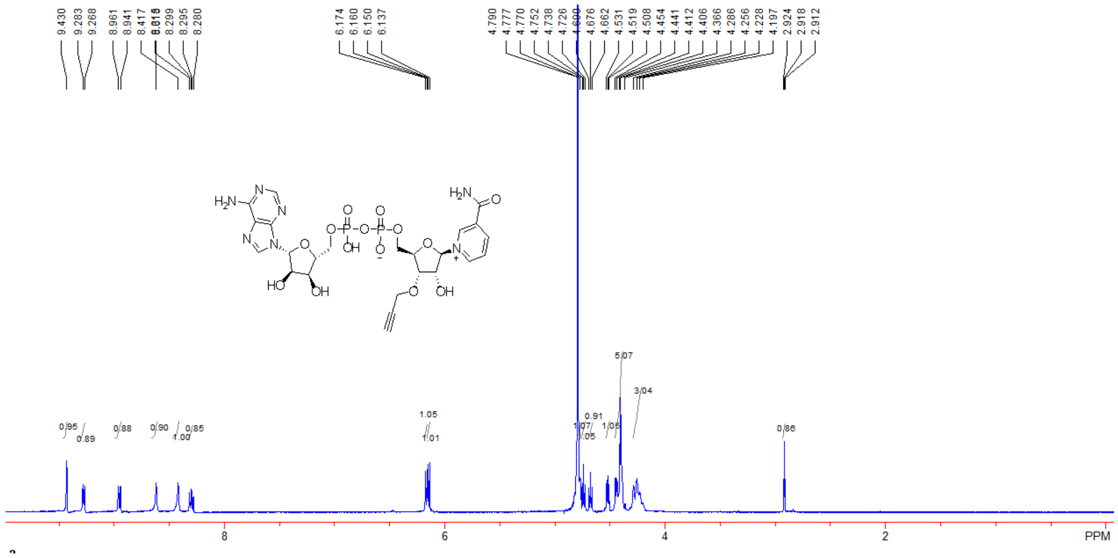

**Supplementary Figure 73**. $^{1}$H NMR spectrum of compound **2**.

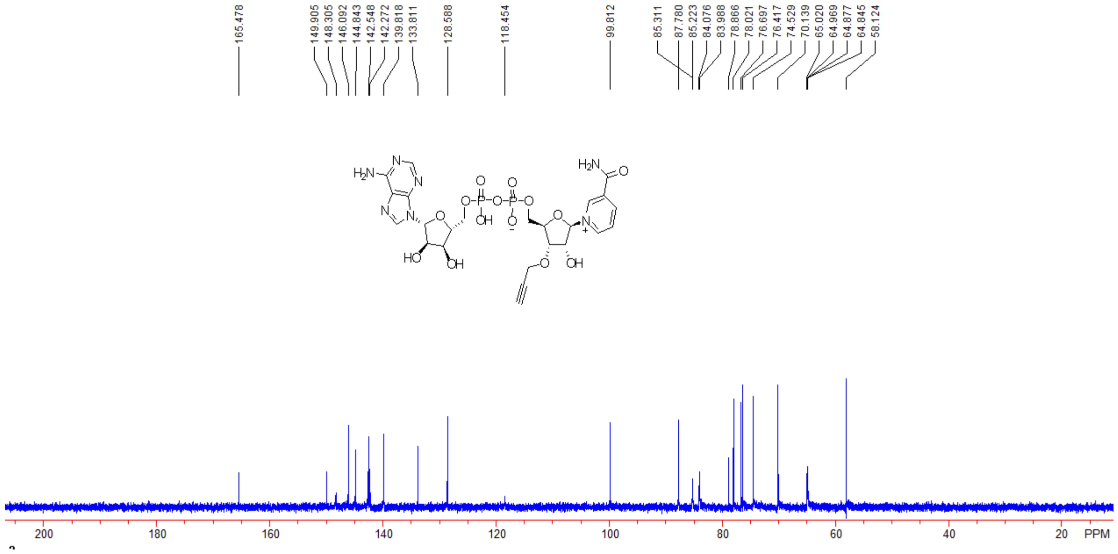

**Supplementary Figure 74**. $^{13}$C NMR spectrum of compound **2**.

**Synthesis of NAD+ analogue 3 (Supplementary Figure 5).**

To a stirred solution of compound **2-2** (3.62 g, 9.0 mmol) in anhydrous THF (30 mL) was added NaH (432 mg, 10.8 mmol, 1.2 eq, 60% dispersion in mineral oil) at 0°C followed by the addition of pent-4-yn-1-yl trifluoromethanesulfonate (2.92 g, 13.5 mmol, 1.5 eq) at the same temperature. Then the reaction mixture was allowed to warm to room temperature. After stirring at this temperature for 8 hours, the reaction mixture was quenched with saturated aqueous $NH_4Cl$ (20 mL) and extracted with EtOAc (3×50 mL). The combined organic layers were washed water (3×50 mL), dried over anhydrous $Na_2SO_4$, filtered and concentrated and purified by a flash column chromatography on silica gel to afford the desired compound **3-3** (1.69 g, 40%) as a colorless oil. $^1$H NMR (400 MHz, $CDCl_3$): δ 1.07 (s, 9H, 3$CH_3$), 1.81-1.88 (m, 2H, $CH_2$), 1.99 (t, 1H, $J$ = 2.8 Hz, CH), 2.30-2.36 (m, 2H, $CH_2$), 2.56 (d, 1H, $J$ = 8.0 Hz, OH), 3.35 (s, 3H, $OCH_3$), 3.65-3.85 (m, 5H, 2$CH_2$+CH), 4.01 (dd, 1H, $J$ = 10.0, 4.0 Hz, CH), 4.28-4.33 (m, 1H, CH), 4.91 (d, 1H, $J$ = 0.8 Hz, CH), 7.36-7.45 (m, 6H, ArH), 7.70-7.73 (m, 4H, ArH) (Supplementary Figure 75); $^{13}$C NMR (100 MHz, $CDCl_3$): δ 15.2, 19.3, 26.8, 28.3, 55.3, 64.6, 69.0, 69.2, 71.0, 82.7, 83.5, 84.6, 105.8, 127.66, 127.68, 129.63, 129.67, 133.39, 133.41, 135.6 (Supplementary Figure 76).

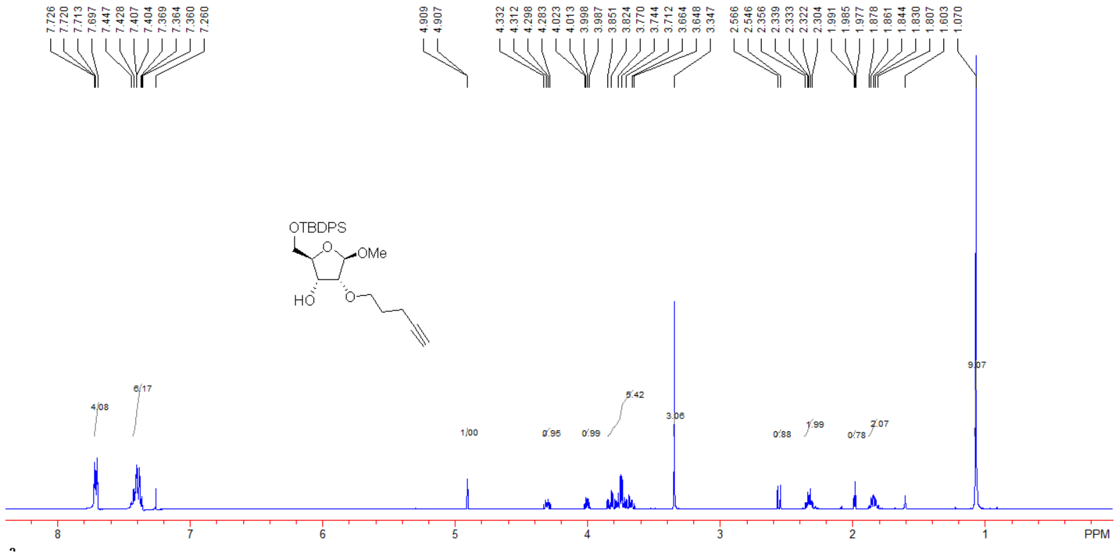


**Supplementary Figure 75**. $^1$H NMR spectrum of compound **3-3**.

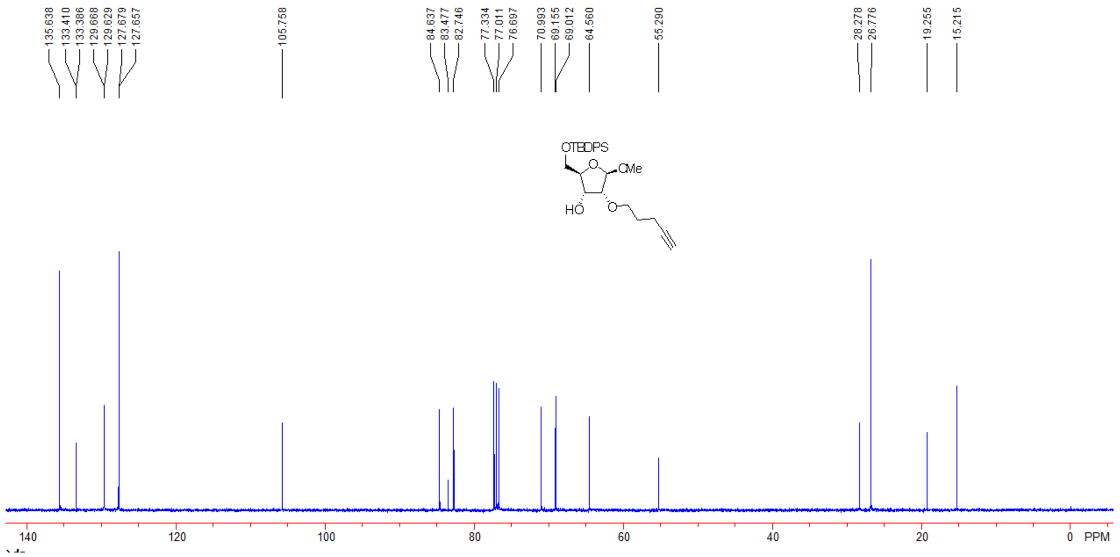


**Supplementary Figure 76**. $^{13}C$ NMR spectrum of compound **3-3**.

To a 0°C solution of compound **3-3** (1.17 g, 2.50 mmol) in anhydrous THF (25 mL) was added AcOH (225 mg, 3.75 mmol, 1.5 eq) followed by the addition of TBAF (3.75 mL, 3.75 mmol, 1.0 M in THF, 1.5 eq). Then the reaction mixture was allowed to warm to room temperature. After stirring at this temperature for 14 hours, the reaction mixture was concentrated under reduced pressure to give a residue. The residue was purified by a flash column chromatography on silica gel to afford the desired compound **3-4** (524 mg, 91%) as a colorless oil. $^1H$ NMR (400 MHz, $CDCl_3$): δ 1.78-1.85 (m, 2H, $CH_2$), 1.98 (t, 1H, $J$ = 2.4 Hz, CH), 2.28-2.32 (m, 2H, $CH_2$), 2.77 (d, 1H, $J$ = 8.0 Hz, OH), 3.40 (s, 3H, $OCH_3$), 3.58-3.69 (m, 2H, $CH_2$), 3.73-3.79 (m, 3H, $CH_2$+CH), 4.01-4.04 (m, 1H, CH), 4.22-4.27 (m, 1H, CH), 4.88 (d, 1H, $J$ = 1.2 Hz, CH) (Supplementary Figure 77); $^{13}C$ NMR (100 MHz, $CDCl_3$): δ 15.2, 28.2, 55.7, 63.1, 69.1, 69.3, 70.9, 83.1, 83.4, 85.6, 106.5 (Supplementary Figure 78).

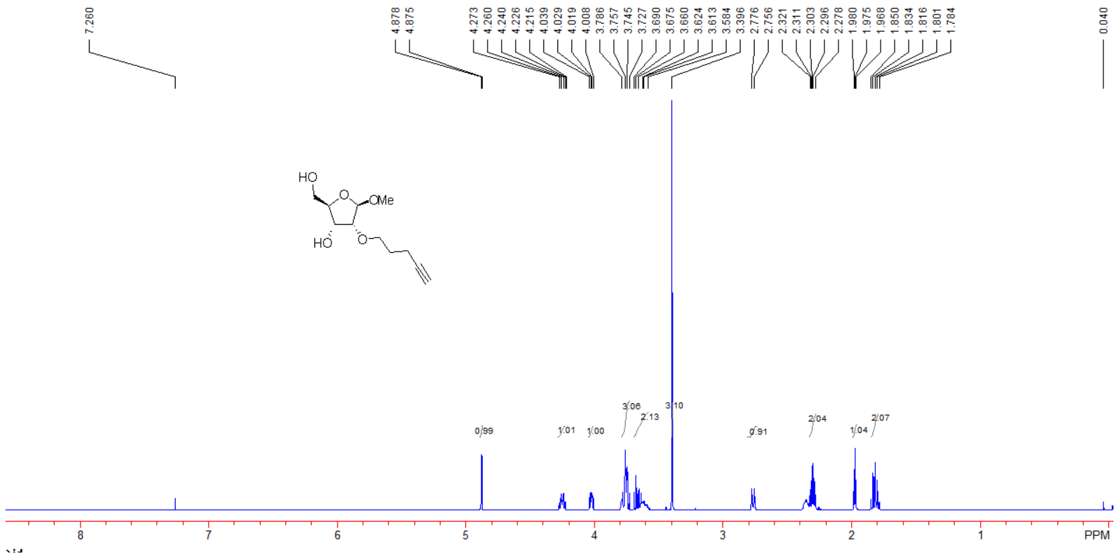


**Supplementary Figure 77**. $^{1}$H NMR spectrum of compound **3-4**.

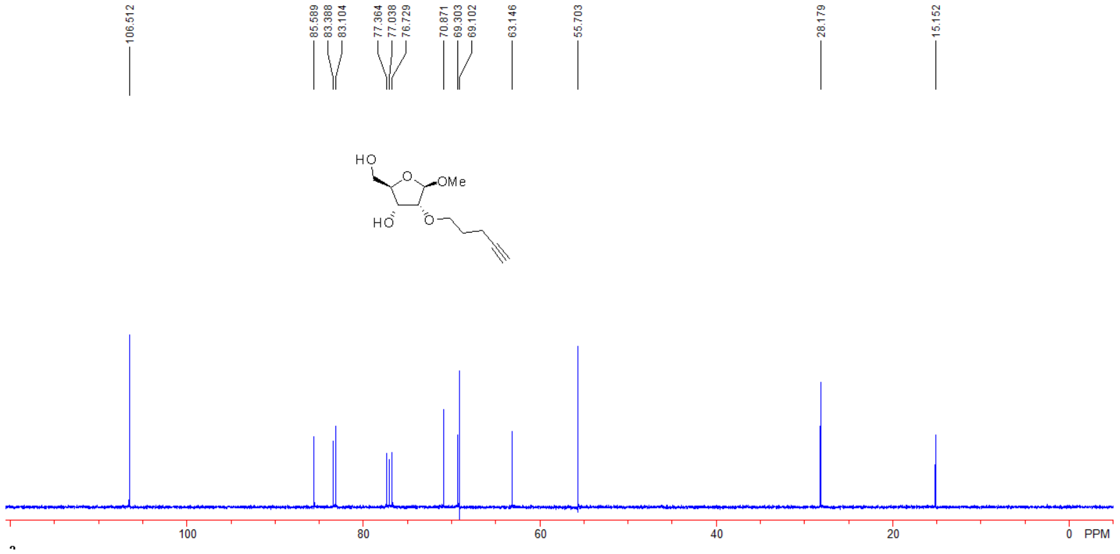


**Supplementary Figure 78**. $^{13}$C NMR spectrum of compound **3-4**.

To a solution of compound **3-4** (460 mg, 2.0 mmol) in a mixture of anhydrous DCM (10 mL) and anhydrous pyridine (10 mL) was added BzCl (691 µL, 6.0 mmol, 3 eq) at 0°C. Then the reaction mixture was allowed to warm to room temperature. After stirring for 24 hours, the reaction was quenched with MeOH (10 mL) and the mixture was concentrated under reduced pressure to give a residue. The residue was dissolved in EtOAc (50 mL), and the organic phase was washed successively with saturated aqueous $CuSO_4$ (3×50 mL), brine (50 mL), dried over anhydrous $Na_2SO_4$, filtered, concentrated and purified by a flash column chromatography on silica gel to afford the desired compound **3-5** (702 mg, 80%) as a colorless oil. $^{1}$H NMR (400

MHz, $CDCl_3$): δ 1.68-1.75 (m, 2H, $CH_2$), 1.84 (t, 1H, $J$ = 2.4 Hz, CH), 2.12-2.23 (m, 2H, $CH_2$), 3.37 (s, 3H, $OCH_3$), 3.57-3.62 (m, 1H, $CH_2$), 3.65-3.70 (m, 1H, $CH_2$), 4.17 (d, 1H, $J$ = 4.8 Hz, CH), 4.45 (dd, 1H, $J$ = 11.6, 4.8 Hz, $CH_2$), 4.58-4.67 (m, 2H, $CH_2$+CH), 4.98 (s, 1H, CH), 5.48 (dd, 1H, $J$ = 6.6, 4.8 Hz, CH), 7.37-7.41 (m, 2H, ArH), 7.43-7.47 (m, 2H, ArH), 7.52-7.60 (m, 2H, ArH), 8.04-8.05 (m, 2H, ArH), 8.06-8.07 (m, 2H, ArH) (Supplementary Figure 79); $^{13}C$ NMR (100 MHz, $CDCl_3$): δ 14.9, 28.6, 55.2, 64.8, 68.6, 69.4, 73.9, 78.6, 81.3, 83.5, 106.8, 128.3, 128.5, 129.4, 129.7, 129.8, 133.1, 133.4, 165.9, 166.3 (Supplementary Figure 80).

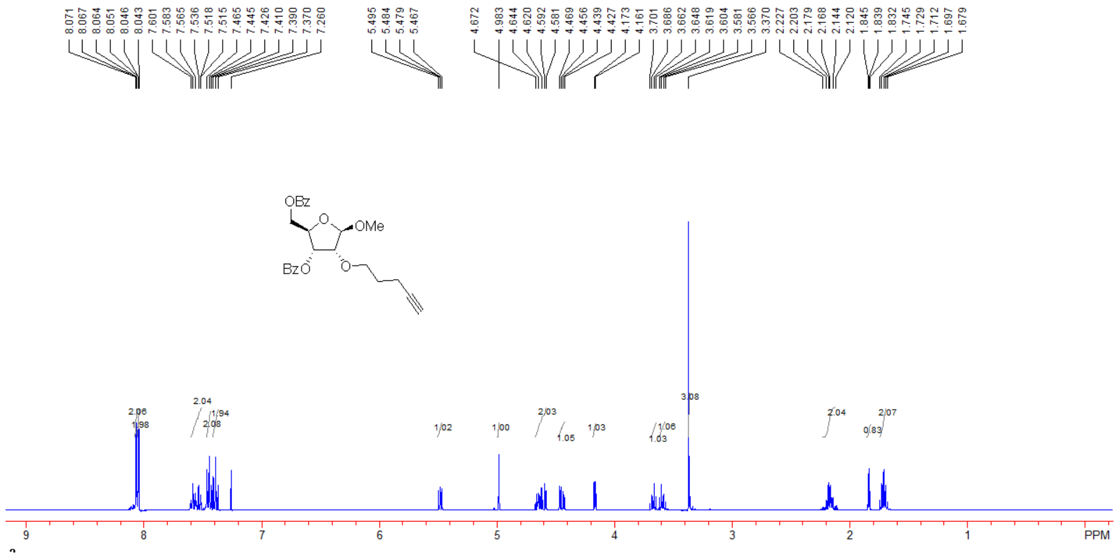

**Supplementary Figure 79**. $^{1}H$ NMR spectrum of compound **3-5**.

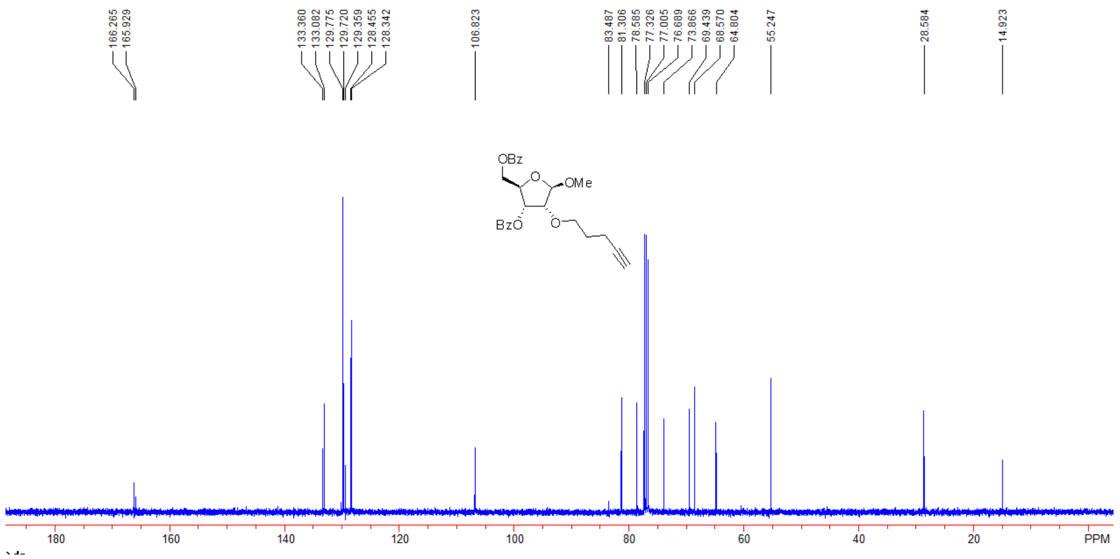

**Supplementary Figure 80**. $^{13}C$ NMR spectrum of compound **3-5**.

Compound **3-5** (570 mg, 1.3 mmol) was dissolved in a mixture of TFA/$H_2O$ (9/1, 15 mL) and the resulting mixture was stirred at room temperature until the reaction complete (monitoring by TLC). Then the reaction was diluted with DCM (60 mL) and the solution was added dropwise to a stirred mixture of ice and saturated aqueous $NaHCO_3$. Solid $NaHCO_3$ was added during the addition to maintain a pH of 7. The mixture was extracted with DCM (3×50 mL), and the combined organic extracts was washed with $H_2O$ (50 mL), brine (50 mL), dried over anhydrous $Na_2SO_4$, filtered, concentrated to give a residue. The residue was dissolved in pyridine (15 mL) and cooled to 0°C. $Ac_2O$ (0.5 mL) was added dropwise and then the resulting mixture was allowed to warm to room temperature. After stirring for 6 hours, the reaction was quenched with MeOH (10 mL) and the mixture was concentrated under reduced pressure to give a residue. The residue was dissolved in EtOAc (50 mL), and the organic phase was washed successively with saturated aqueous $CuSO_4$ (3×50 mL), brine (50 mL), dried over anhydrous $Na_2SO_4$, filtered, concentrated and purified by a flash column chromatography on silica gel to afford the corresponding compound **3-6** (473 mg, 78%) as a colorless oil. $^1$H NMR (400 MHz, $CDCl_3$) of one isomer: δ 1.68-1.74 (m, 2H, $CH_2$), 1.84 (t, 1H, *J* = 2.8 Hz, CH), 1.97 (s, 3H, $CH_3$), 2.14-2.19 (m, 2H, $CH_2$), 3.57-3.62 (m, 1H, $CH_2$), 3.71-3.76 (m, 1H, $CH_2$), 4.27 (d, 1H, *J* = 5.2 Hz, CH), 4.42-4.47 (m, 1H, $CH_2$), 4.6 8-4.74 (m, 2H, $CH_2$+CH), 5.45 (dd, 1H, *J* = 7.2, 4.8 Hz, CH), 6.23 (s, 1H, CH), 7.37-7.41 (m, 2H, ArH), 7.44-7.47 (m, 2H, ArH), 7.52-7.56 (m, 1H, ArH), 7.58-7.62 (m, 1H, ArH), 8.04-8.07 (m, 4H, ArH) (Supplementary Figure 81); $^{13}$C NMR (100 MHz, $CDCl_3$): δ 14.9, 21.0, 28.4, 63.7, 68.7, 69.5, 72.6, 79.5, 80.7, 83.4, 99.1, 128.4, 128.5, 129.0, 129.6, 129.7, 129.8, 133.2, 133.6, 166.96, 166.05, 169.6 (Supplementary Figure 82).

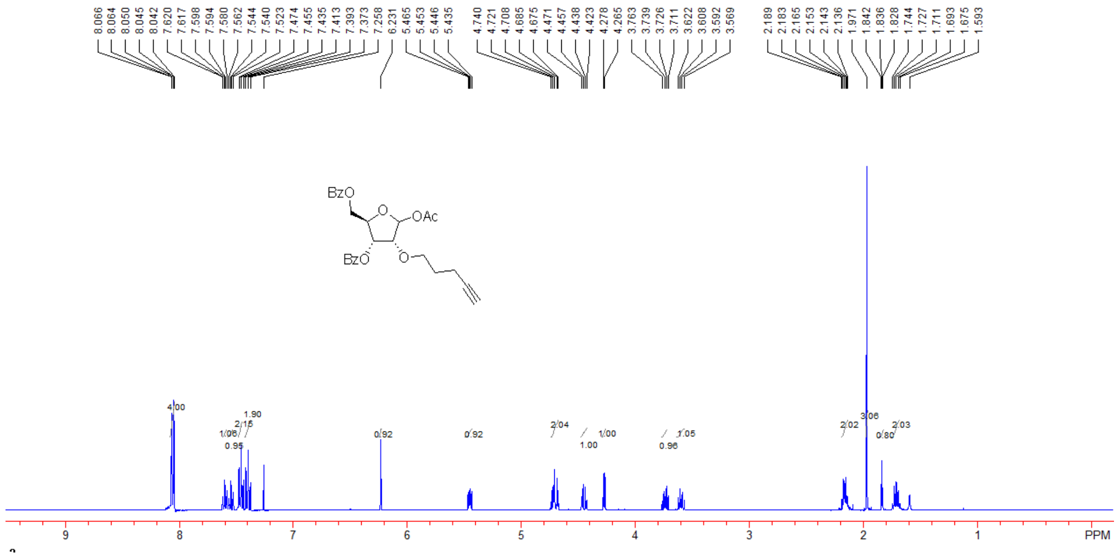

**Supplementary Figure 81**. $^{1}$H NMR spectrum of compound **3-6**.

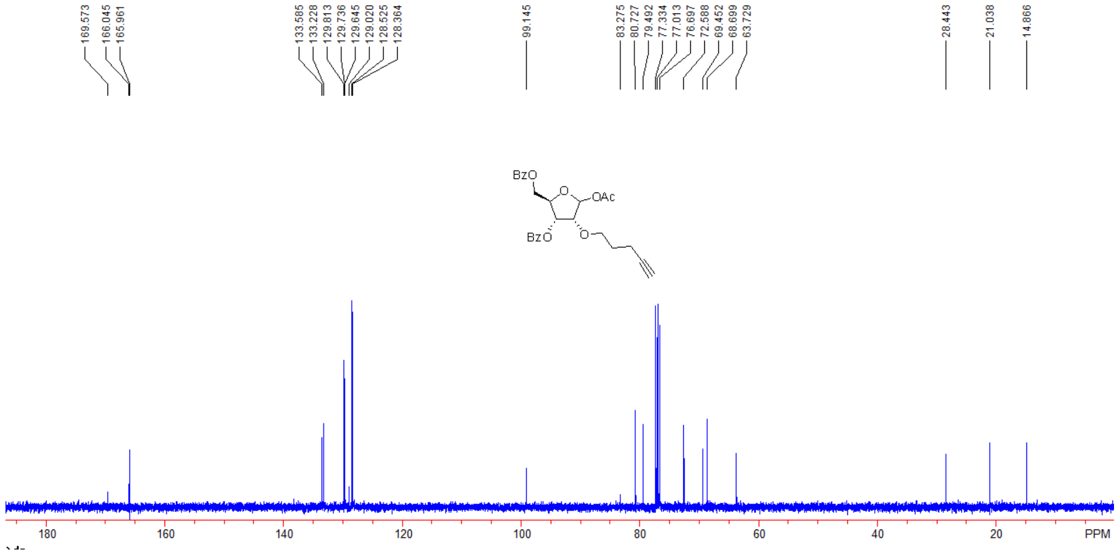

**Supplementary Figure 82**. $^{13}$C NMR spectrum of compound **3-6**.

To a stirred solution of compound **3-6** (327 mg, 0.70 mmol) and nicotinamide (128 mg, 1.05 mmol, 1.5 eq) in anhydrous $CH_3CN$ (15 mL), TMSOTf (506 μL, 2.8 mmol, 4.0 eq) was added dropwise at 0°C and the reaction mixture was stirred for 2 hours at the same temperature. Then the reaction was quenched with MeOH (1 mL) and the mixture was concentrated under reduced pressure to give a residue. The residue was purified by a flash column chromatography on silica gel to afford the crude product which was then purified via preparative HPLC (C18-A column, 150×10.0 mm, 5 μm) (mobile phase A: 0.1% formic acid (aq), mobile B: 0.1% formic acid in acetonitrile; flow rate = 2.0 mL min$^{-1}$; 0-2 min: 0-4% B, 2-4 min: 4-10% B, 4-6 min: 10-20%

B, 6-12 min: 20-50% B, 12-17 min: 50-100% B, 17-20 min: 100-0% B). Fractions containing the desired product were concentrated and lyophilized to yield the **3-7** (271 mg, 57% yield) as a colorless solid. $^{1}$H NMR (400 MHz, $CD_3OD$): δ 1.66-1.73 (m, 2H, $CH_2$), 2.07 (t, 1H, $J$ = 2.4 Hz, CH), 2.17-2.22 (m, 2H, $CH_2$), 3.61-3.67 (m, 1H, $CH_2$), 3.83 (dt, 1H, $J$ = 9.2, 5.6 Hz, $CH_2$), 4.54 (dd, 1H, $J$ = 6.4, 5.3 Hz, CH), 4.84 (dd, 1H, $J$ = 12.4, 3.6 Hz, $CH_2$, overlap with water), 4.90 (dd, 1H, J = 12.4, 5.2 Hz, $CH_2$, overlap with water), 5.12-5.15 (m, 1H, CH), 5.92 (dd, 1H, $J$ = 4.8, 2.4 Hz, CH), 6.51 (d, 1H, $J$ = 6.4 Hz, CH), 7.45-7.49 (m, 2H, ArH), 7.53-7.58 (m, 2H, ArH), 7.61-7.65 (m, 1H, ArH), 7.67-7.72 (m, 1H, ArH), 7.98-8.01 (m, 2H, ArH), 8.15-8.18 (m, 2H, ArH), 8.28 (dd, 1H, $J$ = 8.0, 6.4 Hz, ArH), 9.05 (dd, 1H, $J$ = 8.0, 1.2 Hz, ArH), 9.29 (d, 1H, $J$ = 6.4 Hz, ArH), 9.58 (s, 1H, ArH) (Supplementary Figure 83); $^{13}$C NMR (100 MHz, $CD_3OD$): δ 15.6, 29.3, 64.8, 70.3, 71.3, 72.9, 83.8, 85.3, 86.2, 99.3, 129.6, 129.84, 129.86, 130.7, 130.9, 134.9, 135.0, 136.0, 141.6, 143.5, 147.0, 164.6, 166.7, 167.6 (Supplementary Figure 84).

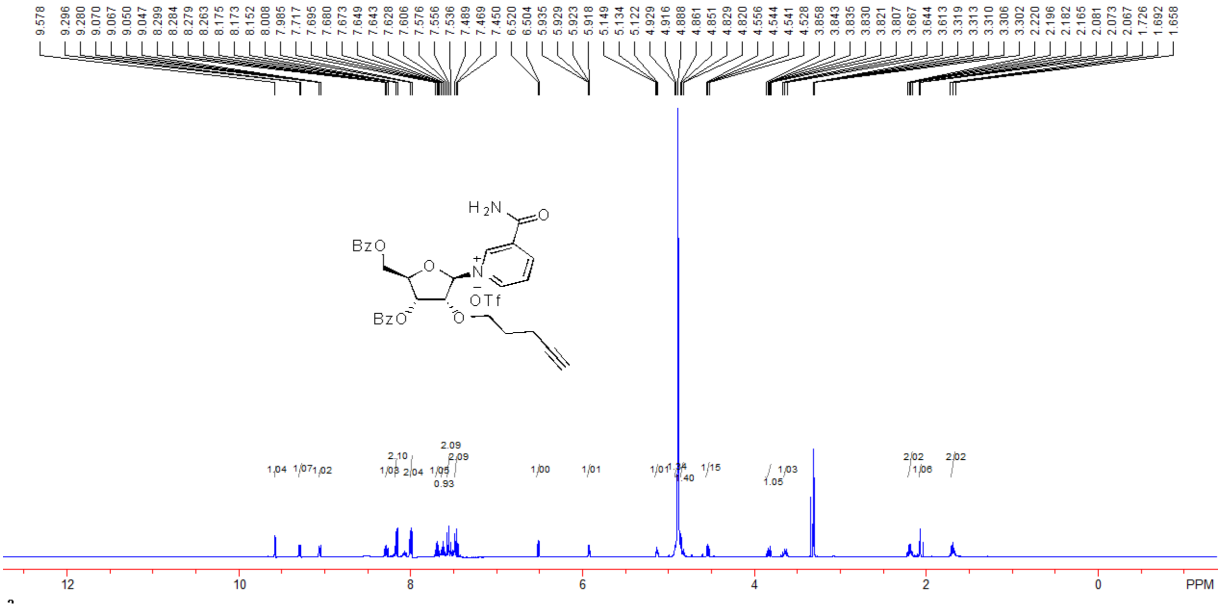


**Supplementary Figure 83**. $^{1}$H NMR spectrum of compound **3-7**.

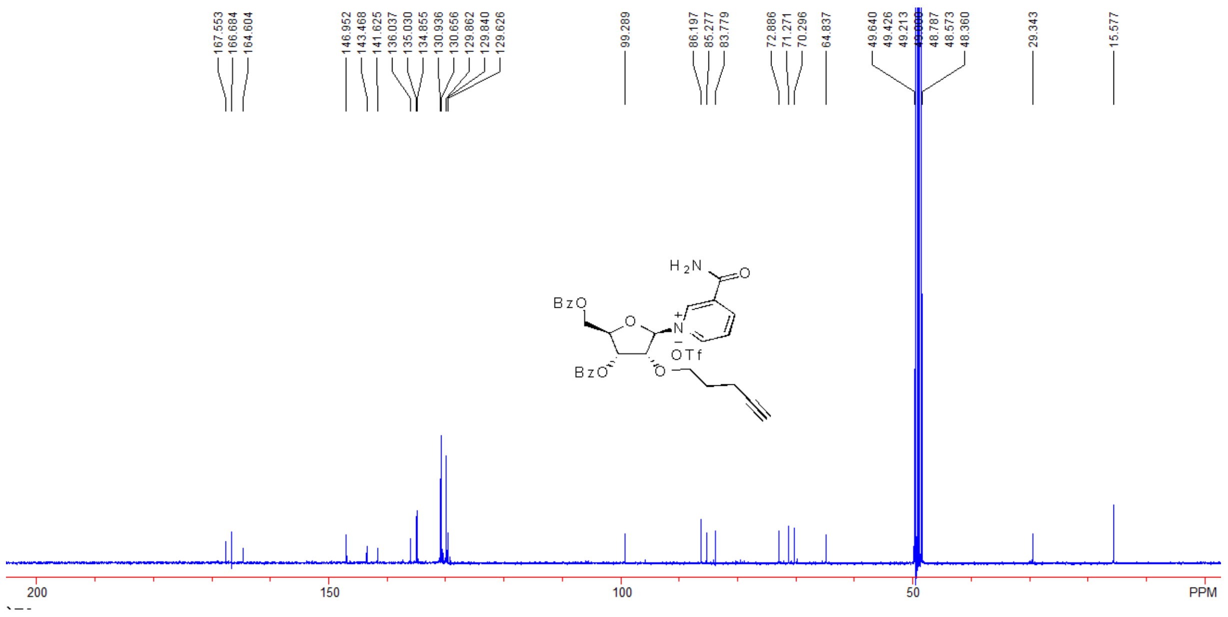

**Supplementary Figure 84**. $^{13}C$ NMR spectrum of compound **3-7**.

Compound (**3-7**) (204 mg, 0.3 mmol) was dissolved in ammonia (15 mL, 7 N in MeOH) and the reaction was stirred at 4°C for 20 hours. The reaction was concentrated under reduced pressure and the crude product was purified via preparative HPLC (C18-A column, 150×10.0 mm, 5 μm) (mobile phase A: 0.1% formic acid (aq), mobile B: 0.1% formic acid in acetonitrile; flow rate = 2.0 ml $min^{-1}$; 0-2 min: 0-4% B, 2-4 min: 4-10% B, 4-6 min: 10-20% B, 6-12 min: 20-50% B, 12-14 min: 50-0% B. Fractions containing the desired product were concentrated and lyophilized to yield the NR3 (99 mg, 70% yield) as a colorless solid. $^{1}H$ NMR (400 MHz, $D_2O$): δ 1.70-1.79 (m, 1H, $CH_2$), 1.80-1.90 (m, 1H, $CH_2$), 2.25 (t, 1H, $J$ = 2.4 Hz, CH), 2.29-2.33 (m, 2H, $CH_2$), 3.64-3.70 (m, 1H, $CH_2$), 3.82-3.89 (m, 2H, $CH_2$+$CH_2$), 4.01 (dd, 1H, $J$ = 12.4, 2.8 Hz, $CH_2$), 4.30 (dd, 1H, $J$ = 5.2, 4.4 Hz, CH), 4.51-4.54 (m, 2H, 2CH), 6.29 (d, 1H, $J$ = 5.2 Hz, CH), 8.28 (dd, 1H, $J$ = 8.0, 6.4 Hz, ArH), 8.99 (dt, 1H, $J$ = 8.0, 1.6 Hz, ArH), 9.26 (d, 1H, $J$ = 6.0 Hz, ArH), 9.57 (s, 1H, ArH) (Supplementary Figure 85); $^{13}C$ NMR (100 MHz, $D_2O$): δ 14.1, 27.1, 60.2, 68.7, 69.4, 69.7, 84.4, 84.9, 88.9, 98.2, 128.3, 133.9, 140.3, 142.4, 145.6, 165.6 (Supplementary Figure 86).

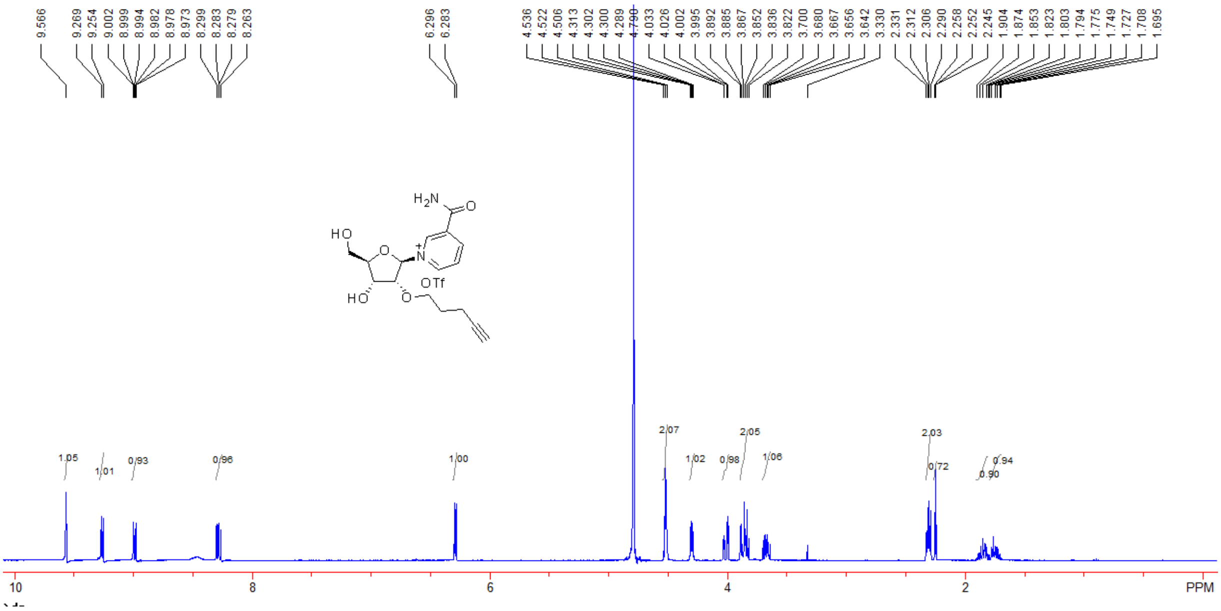

**Supplementary Figure 85**. $^{1}H$ NMR spectrum of compound NR3.

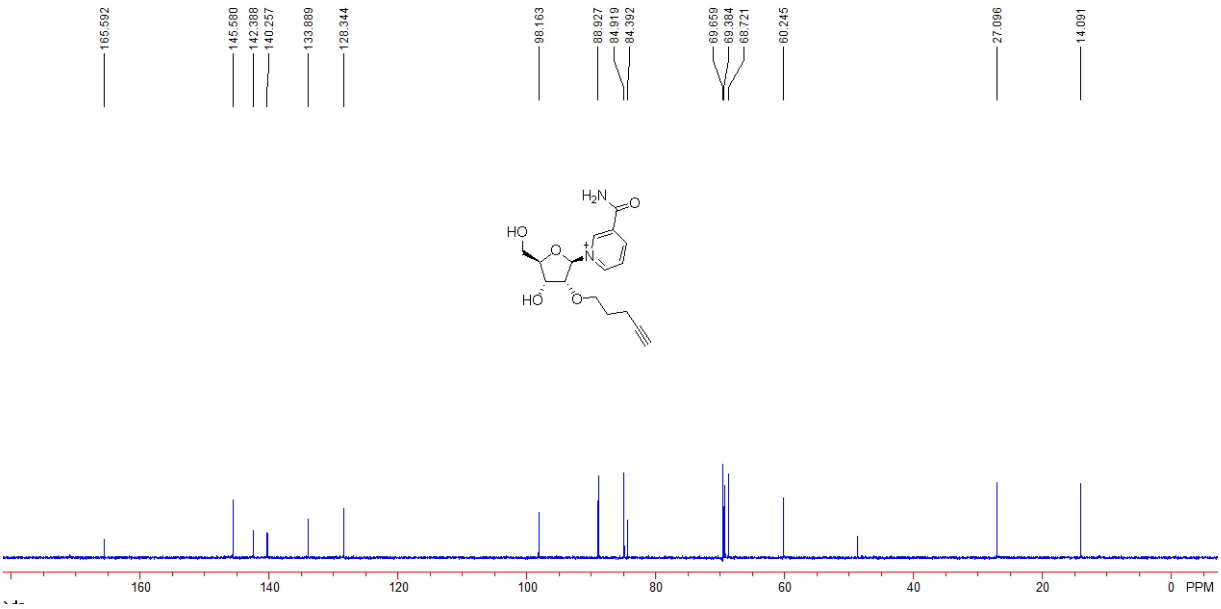

**Supplementary Figure 86**. $^{13}C$ NMR spectrum of compound NR3.

To a stirred solution of compound NR3 (94 mg, 0.2 mmol) in trimethylphosphate (2 mL) was added $P(O)Cl_3$ (130 μL, 1.4 mmol, 7 eq) at 0°C and the resulting mixture was stirred at 0°C for 6 h. A few drops of $H_2O$ were then added to quench the reaction. Trimethylphosphate was removed by extraction with methylene chloride (3×20 ml). The aqueous layer was concentrated *in vacuo* and the crude product was the crude product was purified via preparative HPLC (C18-A column, 150×10.0 mm, 5 μm) (mobile phase A: 0.1% formic acid (aq), mobile B: 0.1% formic acid in acetonitrile; flow rate = 2.0 ml min$^{-1}$; 0-2 min: 0-4% B, 2-4 min: 4-10% B, 4-6 min: 10-

20% B, 6-12 min: 20-50% B, 12-14 min: 50-0% B. Fractions containing the desired product were concentrated and lyophilized to yield the NMN3 (55 mg, 69% yield) as a colorless solid. $^{1}$H NMR (400 MHz, $D_2O$): δ 1.69-1.78 (m, 1H, $CH_2$), 1.80-1.90 (m, 1H, $CH_2$), 2.24 (t, 1H, $J$ = 2.4 Hz, CH), 2.29-2.34 (m, 2H, $CH_2$), 3.63-3.69 (m, 1H, $CH_2$), 3.84 (dt, 1H, $J$ = 9.6, 5.6 Hz, $CH_2$), 4.12 (ddd, 1H, $J$ = 12.0, 4.8, 2.4 Hz, $CH_2$), 4.28 (ddd, $J$ = 12.0, 4.4, 2.4 Hz, $CH_2$), 4.37 (dd, 1H, $J$ = 6.0, 5.2 Hz, CH), 4.62 (dd, 1H, $J$ = 4.8, 1.2 Hz, CH), 4.67-4.69 (m, 1H, CH), 6.25 (d, 1H, $J$ = 6.4 Hz, CH), 8.29 (dd, 1H, $J$ = 8.0, 6.4 Hz, ArH), 8.98 (dt, 1H, $J$ = 8.0, 1.6 Hz, ArH), 9.27 (d, 1H, $J$ = 6.4 Hz, ArH), 9.43 (s, 1H, ArH) (Supplementary Figure 87); $^{13}$C NMR (100 MHz, $D_2O$): δ 14.1, 27.1, 64.1 (d, $J$ = 4.7 Hz), 69.3, 69.6, 69.7, 84.4, 85.1, 88.7 (d, $J$ = 9.2 Hz), 98.4, 128.5, 133,8, 139.7, 142.2, 145.9, 165.7 (Supplementary Figure 88).

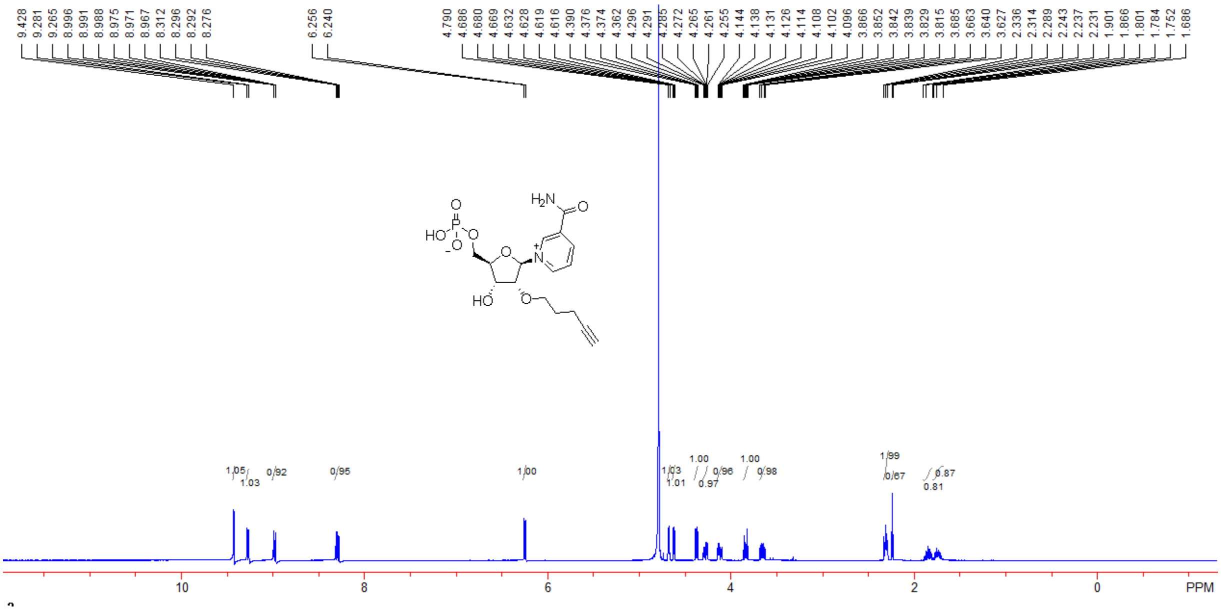

**Supplementary Figure 87**. $^{1}$H NMR spectrum of compound NMN3.

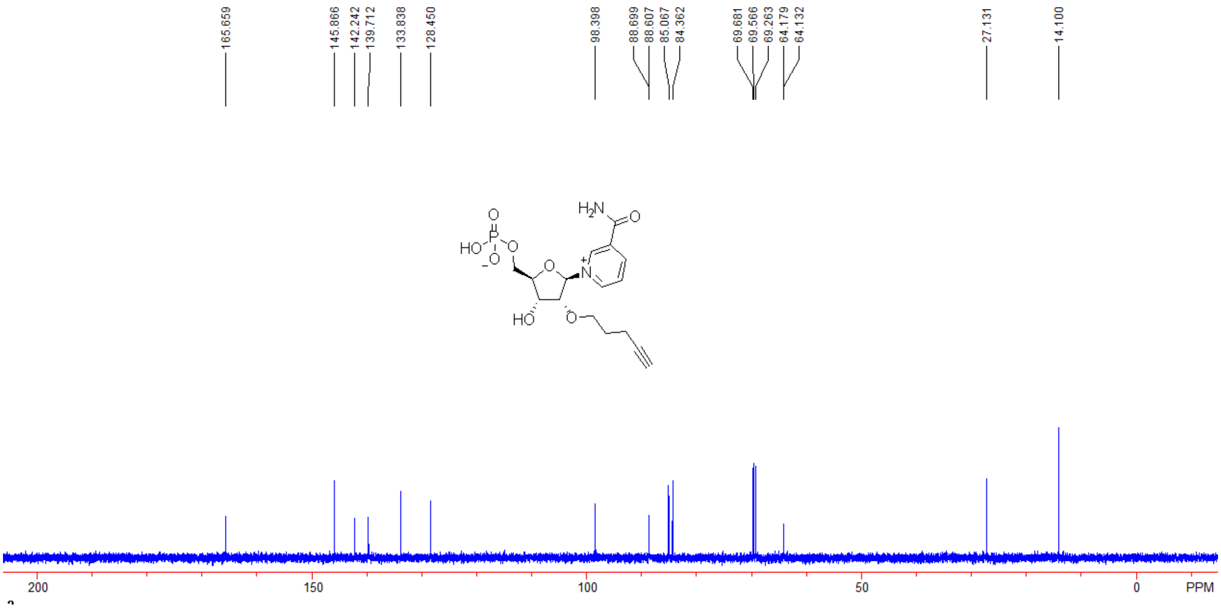


**Supplementary Figure 88**. $^{13}C$ NMR spectrum of compound NMN3.

To a stirred solution of adenosine 5′-monophosphate (5′-AMP) (52 mg, 0.15 mmol, 1.5 eq) in dried DMF (2 mL) were added 1,1-carbonyldiimidazole (CDI) (63 mg, 0.50 mmol, 5 eq) and triethylamine (23 µL, 0.16 mmol. 1.6 eq). The reaction mixture was stirred at room temperature for 8 hours, and then quenched with 0.20 ml dried methanol. The solvent was then removed under vacuum and the residue was coevaporated 3 times each with 1.00 ml of dried DMF. The activated 5′-AMP was dissolved in dried DMF (1 mL) and compound (NMN3) (40 mg, 0.10 mmol) was added. After stirring at room temperature for 4 days, $H_2O$ (5 mL) was added to quench the reaction at 0°C. The resulting mixture was continued stirring at room temperature for 24 hours. The reaction was then concentrated *in vacuo* and the crude product was purified via preparative HPLC (C18-A column, 150×10.0 mm, 5 µm) (mobile phase A: 0.1% formic acid (aq), mobile B: 0.1% formic acid in acetonitrile; flow rate = 2.0 ml min$^{-1}$; 0-2 min: 0-4% B, 2-4 min: 4-10% B, 4-6 min: 10-20% B, 6-12 min: 20-50%, 12-14 min: 50-0% B). Fractions containing the desired product were concentrated and lyophilized to yield the NAD$^+$ analogue **3** (33 mg, 45% yield) as a colorless solid. $^1H$ NMR (400 MHz, $D_2O$): δ 1.64-1.74 (m, 1H, $CH_2$), 1.76-1.86 (m, 1H, $CH_2$), 2.21 (t, 1H, $J$ = 2.8 Hz, CH), 2.25-2.30 (m, 2H, $CH_2$), 3.58-3.63 (m, 1H, $CH_2$), 3.78-3.83 (m, 1H, $CH_2$), 4.17-4.26 (m, 3H, $CH_2$+$CH_2$), 4.33-4.38 (m, 3H, $CH_2$+CH), 4.51 (dd, 1H, $J$ = 4.8, 3.6 Hz, CH), 4.62-4.63 (m, 2H, 2CH), 4.75 (t, 1H, $J$ = 4.8 Hz, CH), 6.13 (d, 1H, $J$ = 5.6 Hz, CH), 6.19 (d, 1H, $J$ = 6.0 Hz, CH), 8.28 (dd, 1H, $J$ = 8.0, 6.4 Hz, ArH), 8.38

(s, 1H, ArH), 8.59 (s, 1H, ArH), 8.94 (dd, 1H, $J$ = 8.0, 1.2 Hz, ArH), 9.25 (d, 1H, $J$ = 6.4 Hz, ArH), 9.39 (s, 1H, ArH) (Supplementary Figure 89); $^{13}$C NMR (100 MHz, $D_2O$): δ 14.1, 27.1, 64.87-64.92 (m), 65.00-65.06 (m), 69.3, 69.6, 70.2, 74.4, 83.97-84.01 (m), 84.05-84.08 (m), 84.3, 84.9, 87.6, 88.2-88.3 (m), 98.3, 118.4, 128.6, 133.8, 139.8, 142.0, 142.3, 145.7, 145.9, 148.4, 150.6, 165.4 (Supplementary Figure 90); HRMS (ESI) for $C_{26}H_{32}N_7O_{14}P_2Na_2^{+1}$ (M+2Na-H)$^+$: Calcd.: 774.1278 Da; Obs: 774.1265 Da.

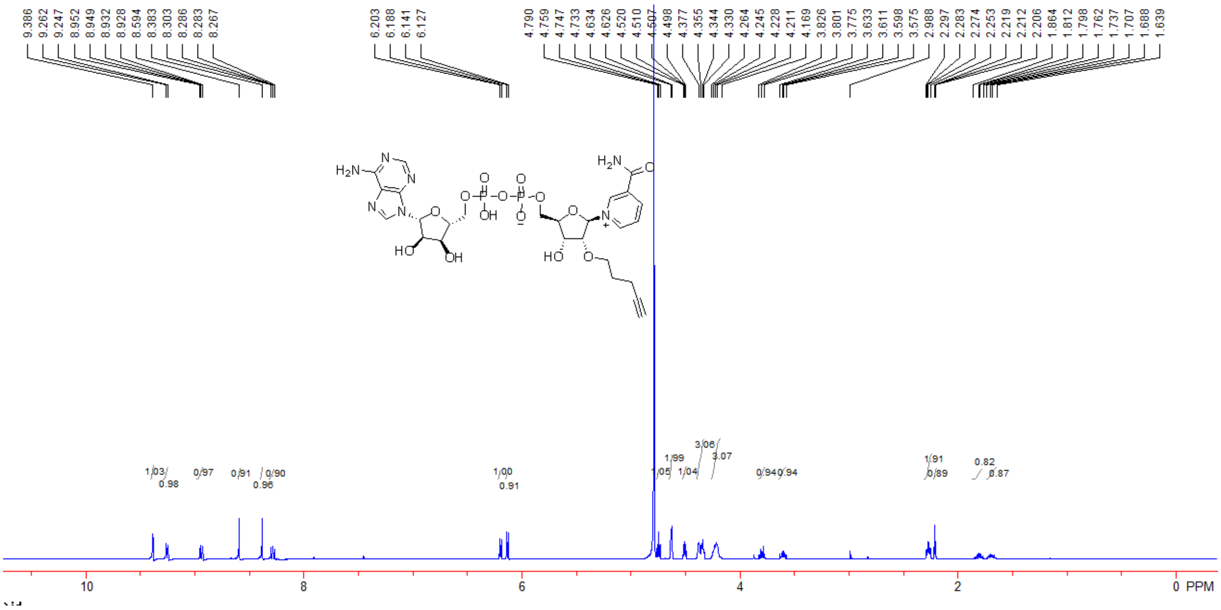


**Supplementary Figure 89**. $^1$H NMR spectrum of compound **3**.

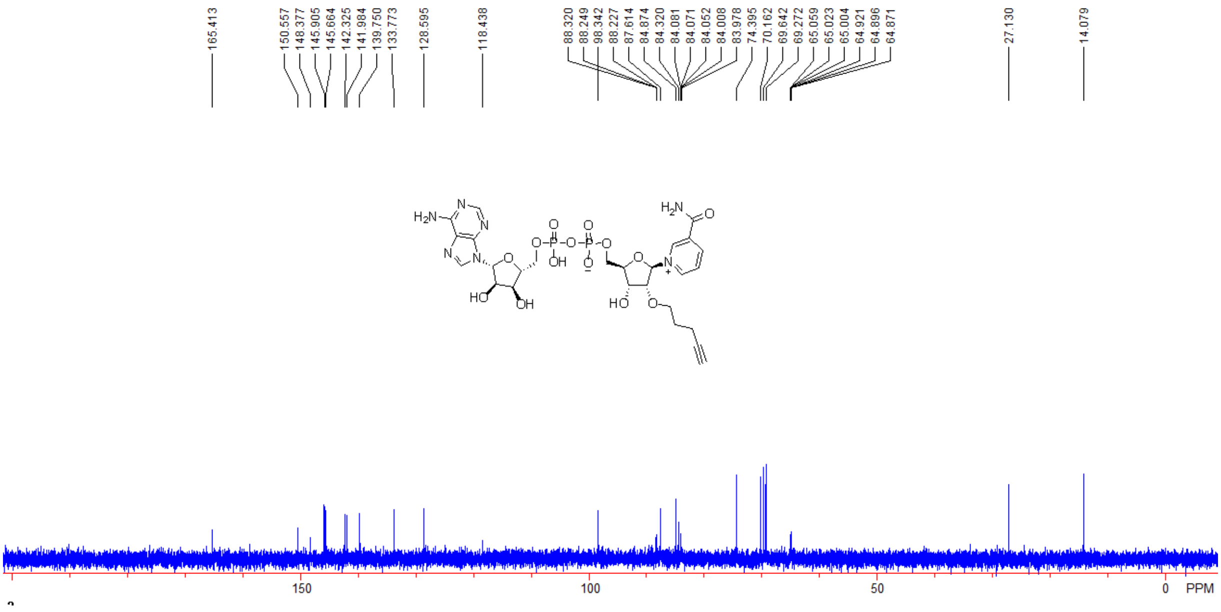


**Supplementary Figure 90**. $^{13}$C NMR spectrum of compound **3**.

**Synthesis of NAD$^+$ analogue 4 (Supplementary Figure 6).**

To a stirred solution of compound **2-2** (3.62 g, 9.0 mmol) in anhydrous THF (30 mL) was added NaH (432 mg, 10.8 mmol, 1.2 eq, 60% dispersion in mineral oil) at 0ºC followed by the addition of pent-4-yn-1-yl trifluoromethanesulfonate (2.92 g, 13.5 mmol, 1.5 eq) at the same temperature. Then the reaction mixture was allowed to warm to room temperature. After stirring at this temperature for 6-8 hours, the reaction mixture was quenched with saturated aqueous $NH_4Cl$ (20 mL) and extracted with EtOAc (3×50 mL). The combined organic layers were washed water (3×50 mL), dried over anhydrous $Na_2SO_4$, filtered and concentrated and purified by a flash column chromatography on silica gel to afford the desired product **4-2** (1.27 g, 30%) as a colorless oil. $^1$H NMR (400 MHz, $CD_3Cl$): δ 1.07 (s, 9H, $3CH_3$), 1.75-1.83 (m, 2H, $CH_2$), 1.95 (t, 2H, $J$ = 2.4 Hz, CH), 2.21-2.35 (m, 2H, $CH_2$), 3.31 (s, 3H, $OCH_3$), 3.57-3.65 (m, 2H, $CH_2$), 3.70-3.80 (m, 2H, $CH_2$), 4.07-4.09 (m, 3H, 3CH), 4.86 (s, 1H, CH), 7.36-7.45 (m, 6H, ArH), 7.68-7.70 (m, 4H, ArH) (Supplementary Figure 91); $^{13}$C NMR (100 MHz, $CD_3Cl$): δ 15.1, 19.3, 26.8, 28.1, 55.1, 64.6, 68.9, 69.1, 73.5, 79.5, 81.9, 83.3, 108.3, 127.68, 127.70, 129.70, 129.74, 133.3, 135.59, 135.61 (Supplementary Figure 92).

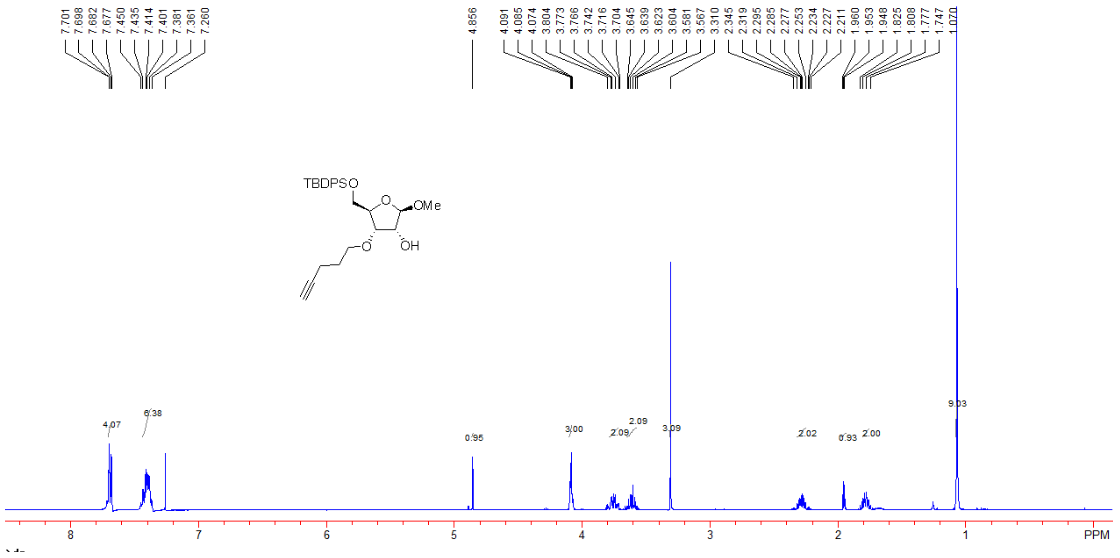


**Supplementary Figure 91**. $^1$H NMR spectrum of compound **4-2**.

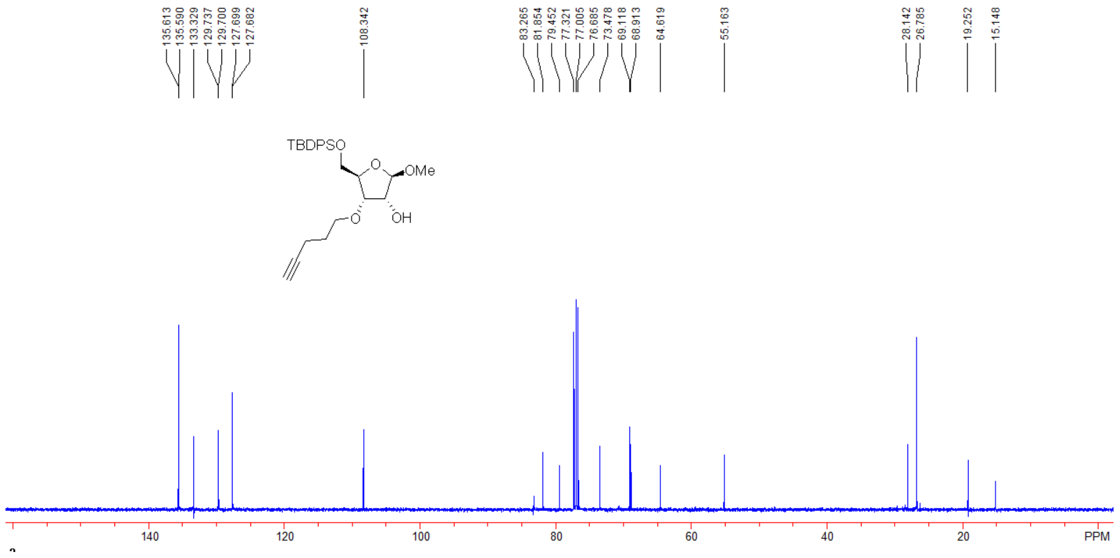

**Supplementary Figure 92**. $^{13}C$ NMR spectrum of compound **4-2**.

To a 0ºC solution of compound **4-2** (1.17 g, 2.5 mmol) in anhydrous THF (25 mL) was added AcOH (225 mg, 3.75 mmol, 1.5 eq) followed by the addition of TBAF (3.75 mL, 3.75 mmol, 1.0 M in THF, 1.5 eq). Then the reaction mixture was allowed to warm to room temperature. After stirring at this temperature for 14 hours, the reaction mixture was concentrated under reduced pressure to give a residue. The residue was purified by a flash column chromatography on silica gel to afford the corresponding compound **4-3** (461 mg, 80%) as a colorless oil. $^{1}H$ NMR (400 MHz, $CD_3Cl$): δ 1.74-1.86 (m, 2H, $CH_2$), 1.98 (t, 2H, $J$ = 2.8 Hz, CH), 2.27-2.34 (m, 2H, $CH_2$), 3.40 (s, 3H, $OCH_3$), 3.58-3.70 (m, 3H, $CH_2$+$CH_2$), 3.81 (dd, 1H, $J$ = 11.6, 2.4 Hz, $CH_2$), 4.07-4.14 (m, 3H, 3CH), 4.86 (s, 1H, CH) (Supplementary Figure 93); $^{13}C$ NMR (100 MHz, $CD_3Cl$): δ 15.0, 28.0, 55.7, 63.0, 69.1, 69.2, 73.5, 78.4, 82.5, 83.1, 108.9 (Supplementary Figure 94).

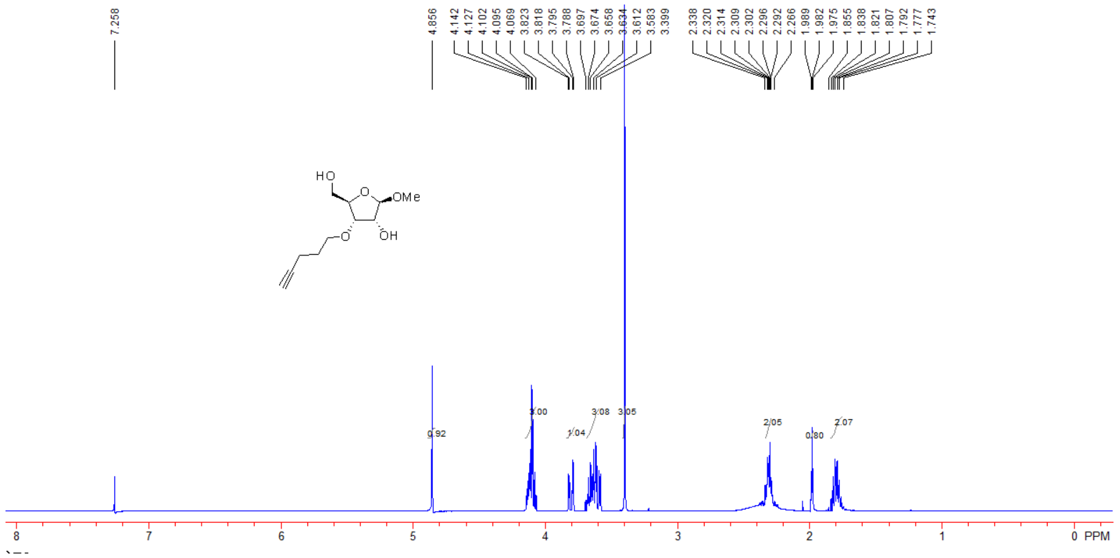


**Supplementary Figure 93**. $^{1}$H NMR spectrum of compound **4-3**.

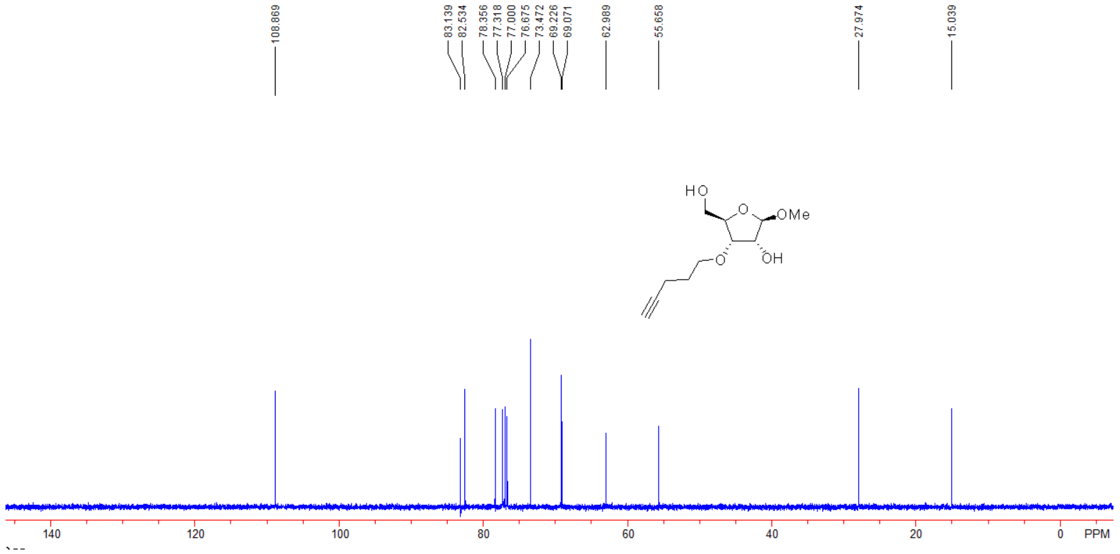


**Supplementary Figure 94**. $^{13}$C NMR spectrum of compound **4-3**.

To a solution of **4-3** (460 mg, 2.0 mmol) in a mixture of anhydrous DCM (10 mL) and anhydrous pyridine (10 mL) was added BzCl (691 µL, 6.0 mmol, 3 eq) at 0°C. Then the reaction mixture was allowed to warm to room temperature. After stirring for 24 hours, the reaction was quenched with MeOH (10 mL) and the mixture was concentrated under reduced pressure to give a residue. The residue was dissolved in EtOAc (50 mL), and the organic phase was washed successively with saturated aqueous $CuSO_4$ (3×50 mL), brine (50 mL), dried over anhydrous $Na_2SO_4$, filtered, concentrated and purified by a flash column chromatography on silica gel to afford the desired compound **4-4** (658 mg, 75%) as a colorless oil. $^{1}$H NMR (400 MHz, $CD_3Cl$):

δ 1.63-1.70 (m, 2H, $CH_2$), 1.82 (t, 2H, *J* = 2.4 Hz, CH), 2.12-2.17 (m, 2H, $CH_2$), 3.36 (s, 3H, $OCH_3$), 3.54-3.59 (m, 1H, $CH_2$), 3.65-3.71 (m, 1H, $CH_2$), 4.30 (dd, 1H, *J* = 7.2, 4.0 Hz, CH), 4.41-4.45 (m, 2H, CH+$CH_2$), 4.65 (dd, 1H, *J* = 13.2, 5.2 Hz, $CH_2$), 5.02 (s, 1H, CH), 5.49 (d, 1H, *J* = 4.0 Hz, CH), 7.44-7.48 (m, 4H, ArH), 7.56-7.61 (m, 2H, ArH), 8.08 (d, 2H, *J* = 7.6 Hz, ArH), 8.12 (d, 2H, *J* = 7.2 Hz, ArH) (Supplementary Figure 95); $^{13}C$ NMR (100 MHz, $CD_3Cl$): δ 15.0, 28.5, 55.2, 64.7, 68.6, 69.6, 74.1, 78.5, 79.1, 83.4, 106.3, 128.4, 128.5, 129.5, 129.7, 129.8, 129.9, 133.1, 133.4, 165.5, 166.4 (Supplementary Figure 96).

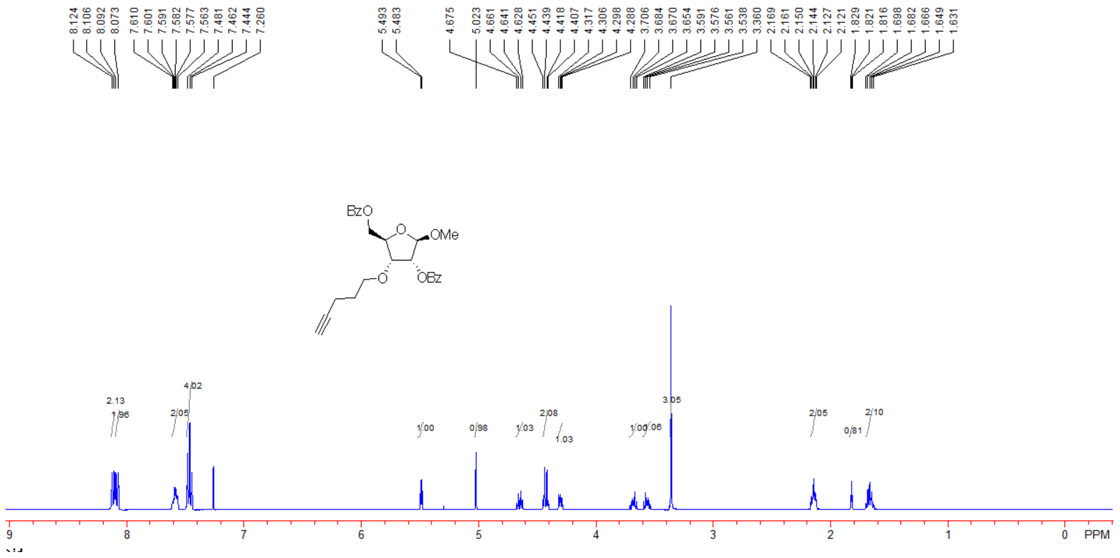


**Supplementary Figure 95**. $^{1}H$ NMR spectrum of compound **4-4**.

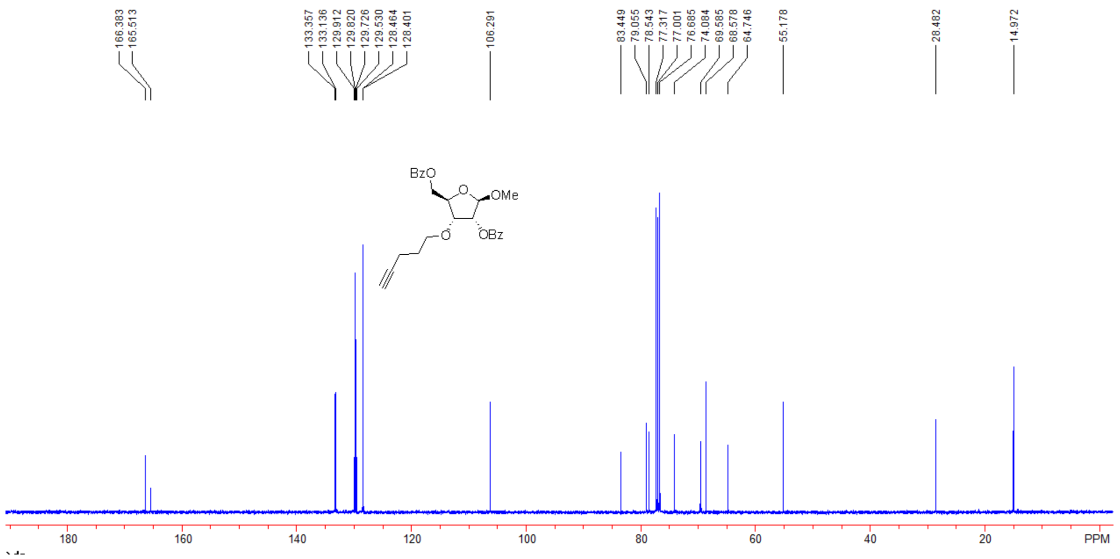


**Supplementary Figure 96**. $^{13}C$ NMR spectrum of compound **4-4**.

Compound **4-4** (570 mg, 1.3 mmol) was dissolved in a mixture of TFA/$H_2O$ (9/1, 15 mL) and the resulting mixture was stirred at room temperature until the reaction complete (monitoring by TLC). Then the reaction was diluted with DCM (60 mL) and the solution was added dropwise to a stirred mixture of ice and saturated aqueous $NaHCO_3$. Solid $NaHCO_3$ was added during the addition to maintain a pH of 7. The mixture was extracted with DCM (3×50 mL), and the combined organic extracts was washed with $H_2O$ (50 mL), brine (50 mL), dried over anhydrous $Na_2SO_4$, filtered, concentrated to give a residue. The residue was dissolved in pyridine (15 mL) and cooled to 0°C. $Ac_2O$ (0.5 mL) was added dropwise and then the resulting mixture was allowed to warm to room temperature. After stirring for 6 hours, the reaction was quenched with MeOH (10 mL) and the mixture was concentrated under reduced pressure to give a residue. The residue was dissolved in EtOAc (50 mL), and the organic phase was washed successively with saturated aqueous $CuSO_4$ (3×50 mL), brine (50 mL), dried over anhydrous $Na_2SO_4$, filtered, concentrated and purified by a flash column chromatography on silica gel to afford the corresponding compound **4-5** (497 mg, 82%) as a colorless oil. $^1$H NMR (400 MHz, $CD_3Cl$) of one isomer: δ 1.63-1.70 (m, 2H, $CH_2$), 1.83 (t, 2H, $J$ = 2.8 Hz, CH), 1.96 (s, 3H, $CH_3$), 2.12-2.17 (m, 2H, $CH_2$), 3.60 (dt, 1H, $J$ = 9.2, 6.0 Hz, $CH_2$), 3.72 (dt, 1H, $J$ = 9.2, 5.5 Hz, $CH_2$), 4.30 (dd, 1H, $J$ = 8.4, 4.4 Hz, CH), 4.42-4.48 (m, 2H, $CH_2$+CH), 4.70 (dt, 1H, $J$ = 13.2, 2.4 Hz, $CH_2$), 5.57 (d, 1H, $J$ = 4.4 Hz, CH), 6.31 (s, 1H, CH), 7.43-7.48 (m, 4H, ArH), 7.56-7.62 (m, 2H, ArH), 8.06-8.12 (m, 4H, ArH) (Supplementary Figure 97); $^{13}$C NMR (100 MHz, $CD_3Cl$): δ 14.9, 20.9, 28.4, 63.7, 68.6, 69.8, 73.7, 77.6, 78.0, 83.3, 98.4, 128.4, 128.5, 129.1, 129.68, 129.73, 129.8, 133.2, 133.5, 165.2, 166.1, 168.9 (Supplementary Figure 98).

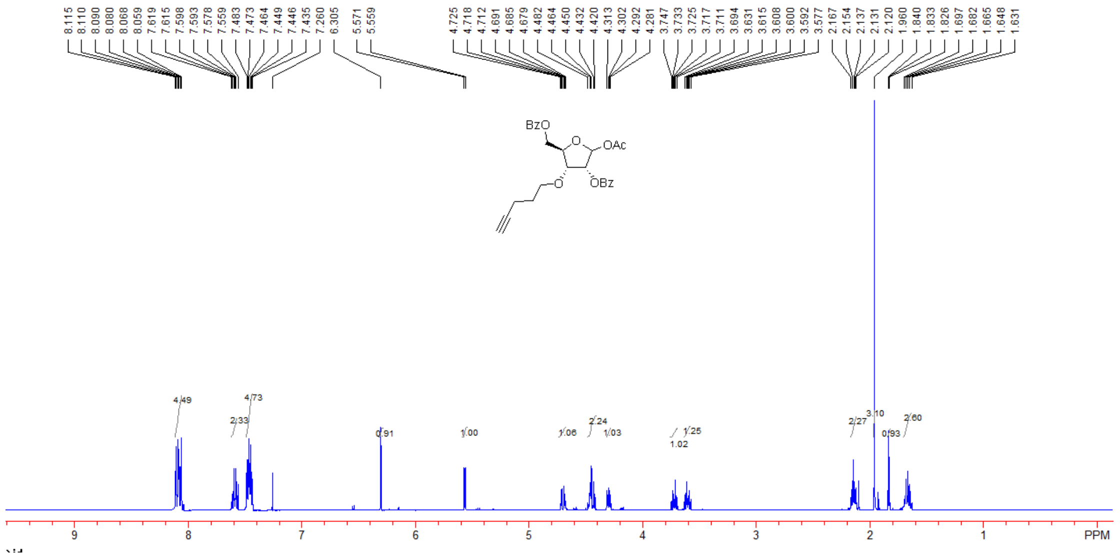


**Supplementary Figure 97**. $^{1}H$ NMR spectrum of compound **4-5**.

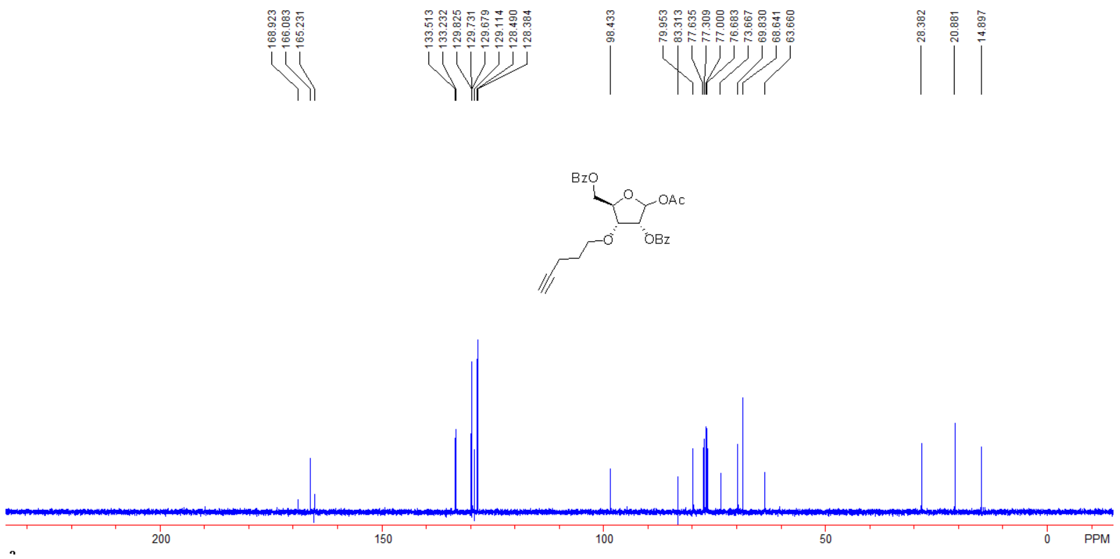


**Supplementary Figure 98**. $^{13}C$ NMR spectrum of compound **4-5**.

Compound **4-5** (327 mg, 0.70 mmol) was dissolved in toluene (10 mL) and cooled to 0°C. HBr (33% (wt) in acetic acid) (257 mg, 1.05 mmol, 1.5 eq) was added dropwise and the reaction was stirred at 0°C for 5 hours. After the starting material was consumed, the reaction was concentrated under reduced pressure to give a residue. The residue was azeotroped with toluene (3×20 mL) to remove remaining acetic acid and dried *in vacuo*. The crude product and nicotinamide (103 mg, 0.84 mmol, 1.2 eq) was dissolved in $CH_3CN$ (20 mL). The reaction was stirred under Ar gas at room temperature for 24 hours. The reaction was concentrated *in vacuo* (the temperature was kept below 35°C) and purified by a flash column chromatography on silica

gel to afford the corresponding compound **4-6** (303 mg, 71%) as a colorless solid. $^{1}$H NMR (400 MHz, $CD_3OD$): δ 1.67-1.74 (m, 2H, $CH_2$), 2.08 (t, 1H, *J* = 2.4 Hz, CH), 2.19 (td, 2H, *J* = 6.8, 2.4 Hz, $CH_2$), 3.70 (t, 2H, *J* = 6.0 Hz, $CH_2$), 4.54-4.55 (m, 1H, CH), 4.87-4.95 (m, 3H, $CH_2$+CH), 5.91-5.94 (m, 1H, CH), 6.76 (s, 1H, CH), 7.51 (t, 2H, *J* = 8.0 Hz, ArH), 7.57 (t, 2H, *J* = 8.0 Hz, ArH), 7.63-7.67 (m, 1H, ArH), 7.69-7.73 (m, 1H, ArH), 8.06-8.08 (m, 2H, ArH), 8.18-8.20 (m, 2H, ArH), 8.24 (dd, 1H, *J* = 8.0, 6.4 Hz, ArH), 9.04 (d, 1H, *J* = 8.0 HZ, ArH), 9.40 (d, 1H, *J* = 6.4 HZ, ArH), 9.66 (s, 1H, ArH) (Supplementary Figure 99); $^{13}$C NMR (100 MHz, $CD_3OD$): δ 15.6, 29.7, 64.1, 70.0, 70.7, 76.8, 78.2, 84.0, 84.5, 99.7, 129.9, 130.65, 130.68, 130.7, 131.1, 134.8, 135.3, 136.0, 142.1, 143.8, 146.8, 164.9, 167.3, 167.5 (Supplementary Figure 100).

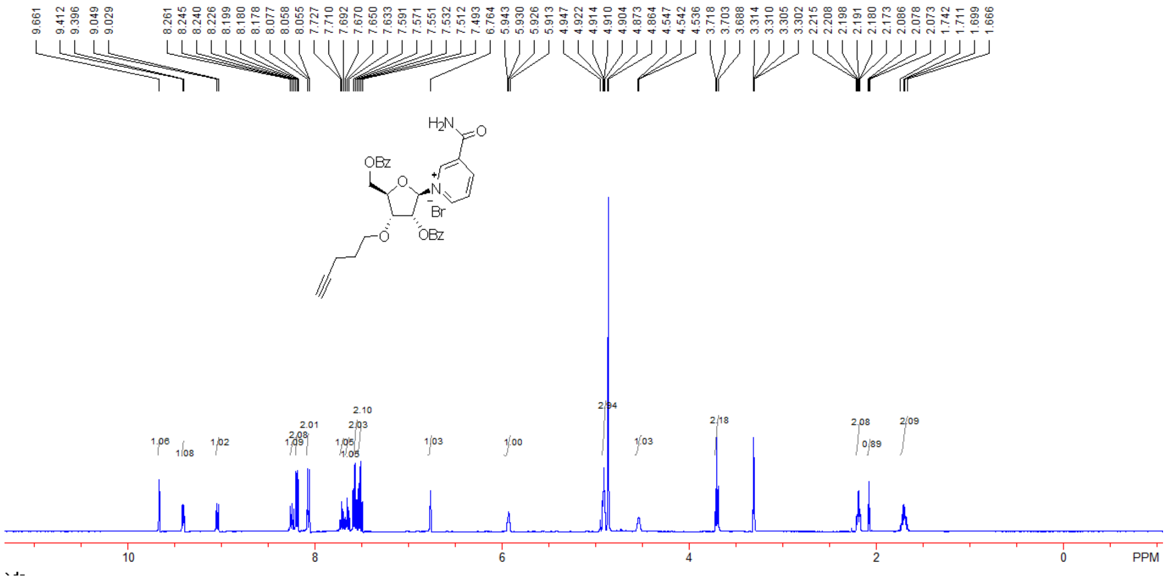


**Supplementary Figure 99**. $^{1}$H NMR spectrum of compound **4-6**.

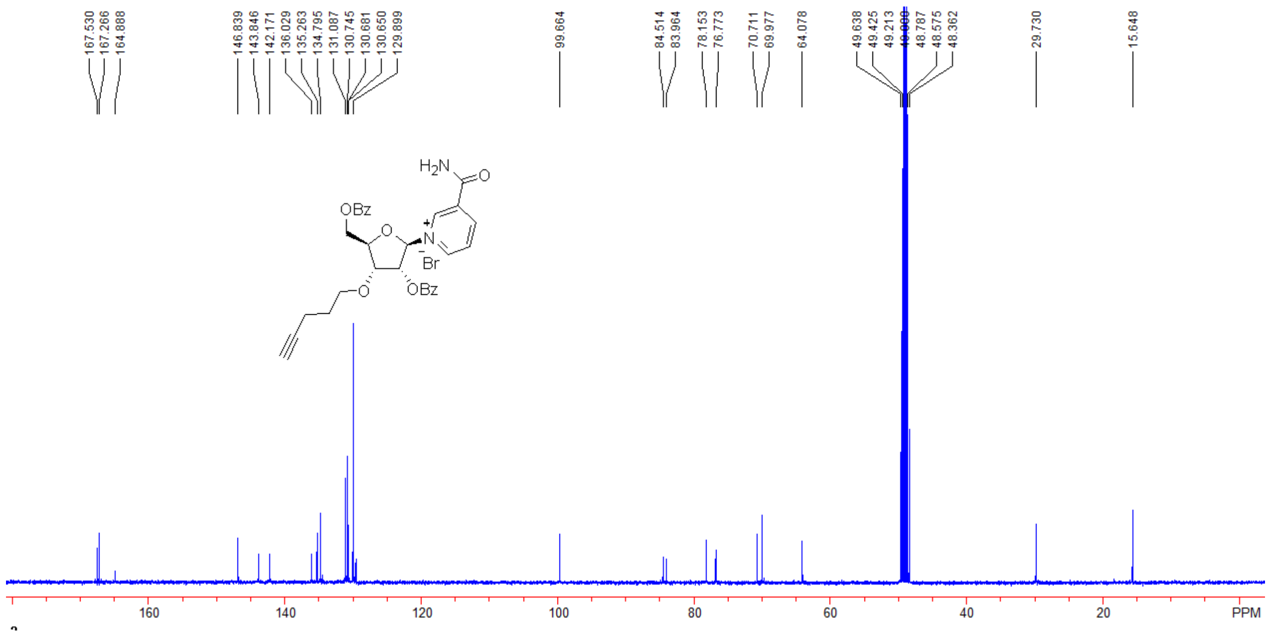


**Supplementary Figure 100**. $^{13}C$ NMR spectrum of compound **4-6**.

General procedure for the synthesis of NR4: Compound **4-6** (244 mg, 0.40 mmol) was dissolved in ammonia (20 mL, 7 N in MeOH) and the reaction was stirred at 0°C for 48 hours. The reaction was concentrated under reduced pressure and the crude product was dissolved in MeOH (0.5 mL). Addition of ethyl ether (10 mL) resulted in ppt of the desired product. The procedure was repeated five times to yield the corresponding desired product NR4 (106 mg, 66%) as a colorless solid**.** $^{1}H$ NMR (400 MHz, $D_2O$): δ 1.79-1.86 (m, 2H, $CH_2$), 2.32-2.36 (m, 3H, CH+$CH_2$), 3.72-3.81 (m, 2H, $CH_2$), 3.91 (dd, 1H, $J$ = 12.8, 3.6 Hz, $CH_2$), 4.08 (dd, 1H, $J$ = 12.8, 2.8 Hz, $CH_2$), 4.16 (t, 1H, $J$ = 4.8 Hz, CH), 4.55-4.58 (m, 1H, CH), 4.66 (t, 1H, $J$ = 4.8 Hz, CH), 6.28 (d, 1H, $J$ = 4.8 Hz, CH), 8.29 (dd, 1H, $J$ = 8.4, 6.8 Hz, ArH), 8.99 (d, 1H, $J$ = 8.4 Hz, ArH), 9.27 (d, 1H, $J$ = 6.8 Hz, ArH), 9.62 (s, 1H, ArH) (Supplementary Figure 101); $^{13}C$ NMR (100 MHz, $D_2O$): δ 14.3, 27.4, 60.1, 69.3, 69.5, 76.1, 76.9, 84.8, 85.7, 100.1, 128.3, 133.9, 140.3, 142.6, 145.6, 165.7 (Supplementary Figure 102).

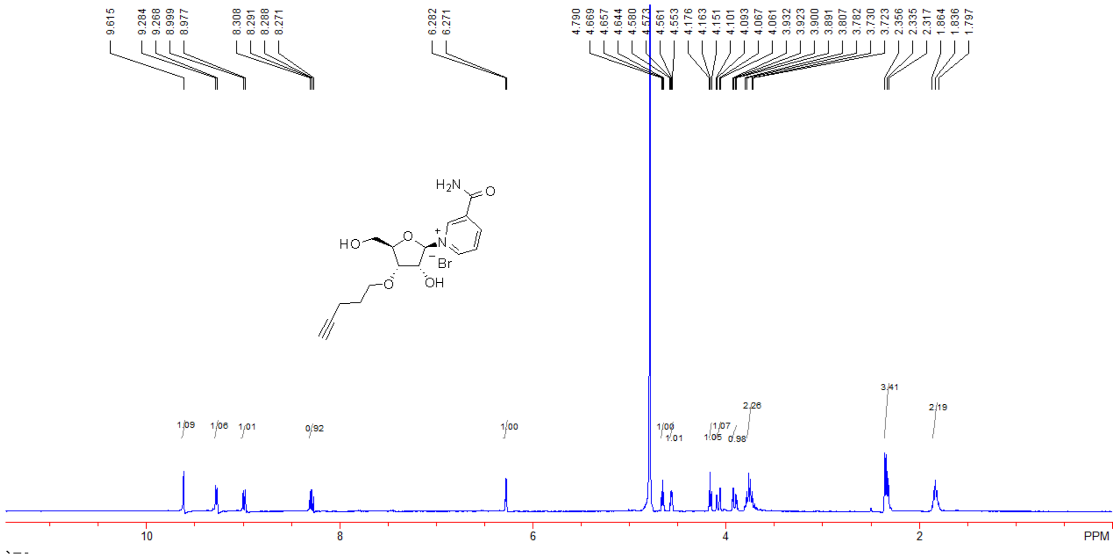


**Supplementary Figure 101**. $^{1}H$ NMR spectrum of compound NR4.

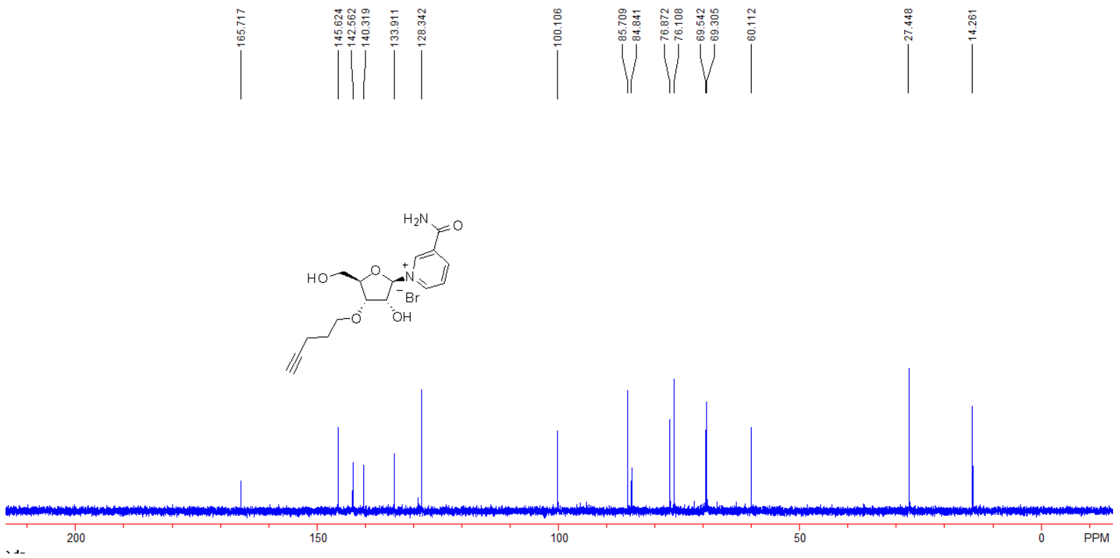


**Supplementary Figure 102**. $^{13}C$ NMR spectrum of compound NR4.

General procedure for the synthesis of compound NMN4: To a stirred solution of NR4 (88 mg, 0.22 mmol) in trimethylphosphate (2 mL) was added $P(O)Cl_3$ (143 µL, 1.54 mmol, 7 eq) at 0ºC and the resulting mixture was stirred at 0ºC for 6 hours. A few drops of $H_2O$ were then added to quench the reaction. Trimethylphosphate was removed by extraction with ethyl ether (3×20 ml). The remaining trimethylphosphate was removed by a second extraction with THF (5 ml). The aqueous layer was concentrated *in vacuo* and the crude product was dissolved in MeOH (0.5 mL). Addition of ethyl ether (10 mL) resulted in ppt of the desired product. The procedure was repeated four times to yield the desired product NMN4 (59 mg, 67%) as a colorless solid.

$^{1}H$ NMR (400 MHz, $D_2O$): δ 1.79-1.86 (m, 2H, $CH_2$), 2.30-2.34 (m, 3H, CH+$CH_2$), 3.78 (t, 2H, *J* = 6.4 Hz, $CH_2$), 4.12-4.17 (m, 1H, $CH_2$), 4.22 (dd, 1H, *J* = 4.8, 2.4 Hz, CH), 4.30-4.35 (m, 1H, $CH_2$), 4.63 (t, 1H, *J* = 4.8 Hz, CH), 4.73 (t, 1H, *J* = 2.4 Hz, CH), 6.20 (d, 1H, *J* = 4.8 Hz, CH), 8.28 (dd, 1H, *J* = 8.0, 6.4 Hz, ArH), 8.97 (d, 1H, *J* = 8.0 Hz, ArH), 9.25 (d, 1H, *J* = 6.4 Hz, ArH), 9.43 (s, 1H, ArH) (Supplementary Figure 103); $^{13}C$ NMR (100 MHz, $D_2O$): δ 14.3, 27.4, 64.4 (d, *J* = 4.5 Hz), 69.3, 69.5, 76.8, 78.2, 85.3 (d, *J* = 9.8 Hz), 100.0, 128.4, 133.9, 139.8, 142.4, 145.9, 165.7 (Supplementary Figure 104).

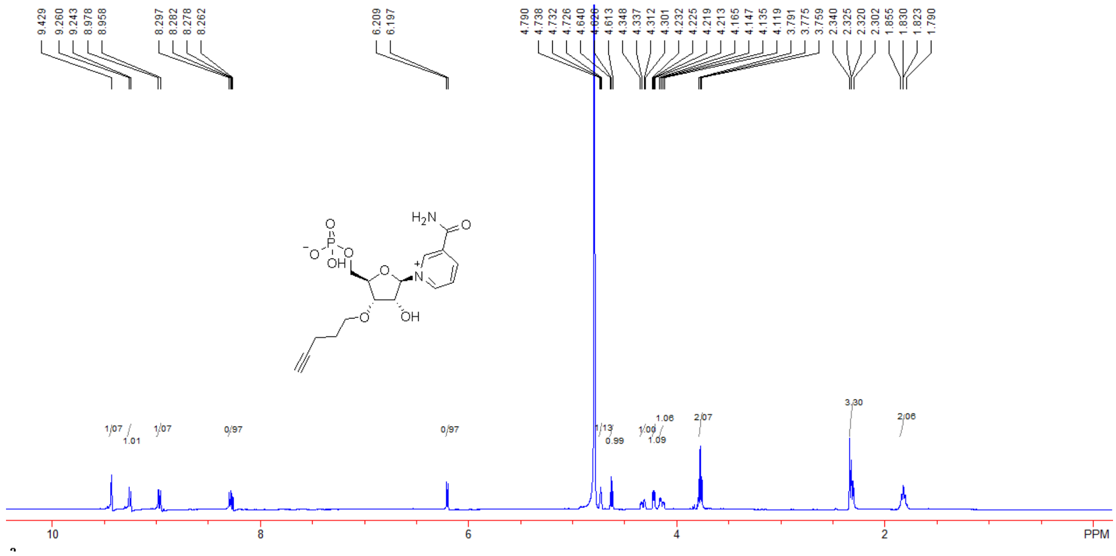


**Supplementary Figure 103**. $^{1}H$ NMR spectrum of compound NMN4.

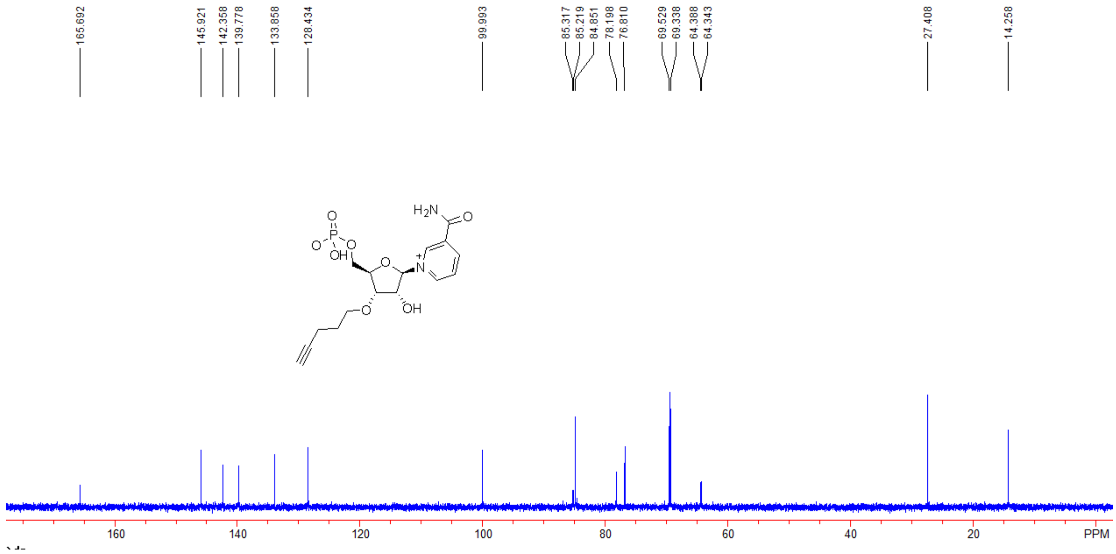


**Supplementary Figure 104**. $^{13}C$ NMR spectrum of compound NMN4.

To a stirred solution of adenosine 5′-monophosphate (5′-AMP) (52 mg, 0.15 mmol, 1.5 eq) in dried DMF (2 mL) were added 1,1-carbonyldiimidazole (CDI) (63 mg, 0.50 mmol, 5 eq) and triethylamine (23 μL, 0.16 mmol. 1.6 eq). The reaction mixture was stirred at room temperature for 8 hours, and then quenched with 0.20 ml dried methanol. The solvent was then removed under vacuum and the residue was coevaporated 3 times each with 1.00 ml of dried DMF. The activated 5′-AMP was dissolved in dried DMF (1 mL) and compound (NMN4) (40 mg, 0.10 mmol) was added. After stirring at room temperature for 4 days, $H_2O$ (5 mL) was added to quench the reaction at 0°C. The resulting mixture was continued stirring at room temperature for 24 hours. The reaction was then concentrated *in vacuo* and the crude product was purified via preparative HPLC (C18-A column, 150×10.0 mm, 5 μm) (mobile phase A: 0.1% formic acid (aq), mobile B: 0.1% formic acid in acetonitrile; flow rate = 2.0 ml $min^{-1}$; 0-2 min: 0-4% B, 2-4 min: 4-10% B, 4-6 min: 10-20% B, 6-12 min: 20-50%, 12-14 min: 50-0% B). Fractions containing the desired product were concentrated and lyophilized to yield the $NAD^+$ analogue **4** (34 mg, 47% yield) as a colorless solid. $^1H$ NMR (400 MHz, $D_2O$): δ 1.79-1.83 (m, 2H, $CH_2$), 2.30-2.35 (m, 3H, CH+$CH_2$), 3.77 (t, 2H, $J$ = 6.0 Hz, $CH_2$), 4.20-4.29 (m, 4H, $CH_2$+CH), 4.39-4.43 (m, 2H, $CH_2$), 4.52 (t, 1H, $J$ = 4.8 Hz, CH), 4.64 (t, 1H, $J$ = 5.2 Hz, CH), 4.72-4.75 (m, 2H, 2CH), 6.13-6.17 (m, 2H, 2CH), 8.28-8.31 (m, 1H, ArH), 8.41 (s, 1H, ArH), 8.61 (s, 1H, ArH), 8.95 (d, 1H, $J$ = 8.0 Hz, ArH), 9.27 (d, 1H, $J$ = 6.0 Hz, ArH), 9.42 (s, 1H, ArH) (Supplementary Figure 105); $^{13}C$ NMR (100 MHz, $D_2O$): δ 14.3, 27.4, 65.00-65.05 (m), 65.10-65.17 (m), 69.3, 69.5, 70.1, 74.5, 76.9, 78.4, 84.0 (d, $J$ = 8.8 Hz), 84.8, 85.3 (d, $J$ = 8.0 Hz), 87.7, 100.0, 118.4, 128.6, 133.8, 139.8, 142.2, 142.5, 145.1, 146.0, 148.3, 150.1, 165.5 (Supplementary Figure 106); HRMS (ESI) for $C_{26}H_{34}N_7O_{14}P_2^{+1}$ $(M)^+$: Calcd.: 730.1639 Da; Obs: 730.1631 Da.

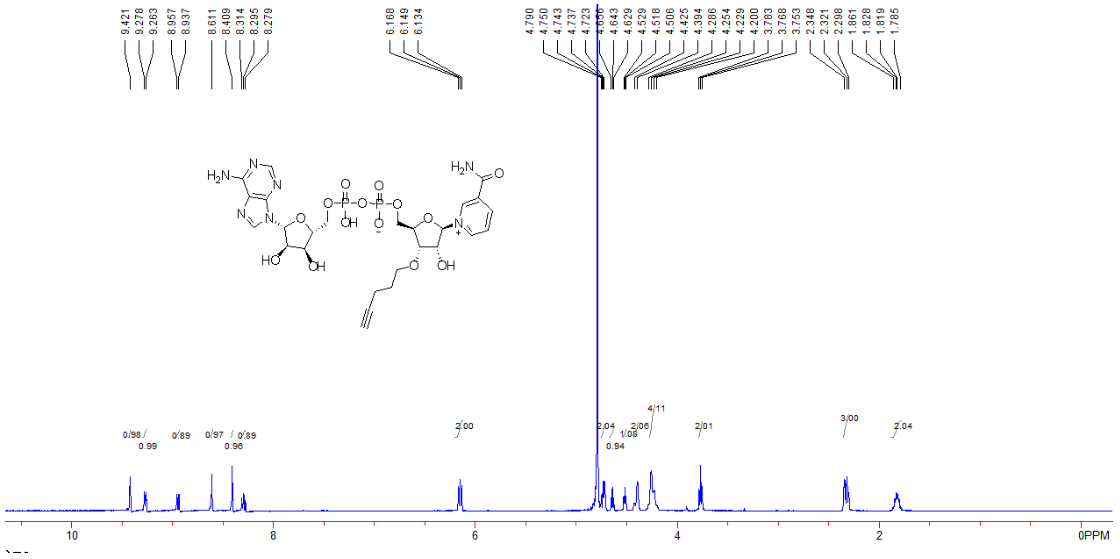

**Supplementary Figure 105**. $^{1}$H NMR spectrum of compound **4**.

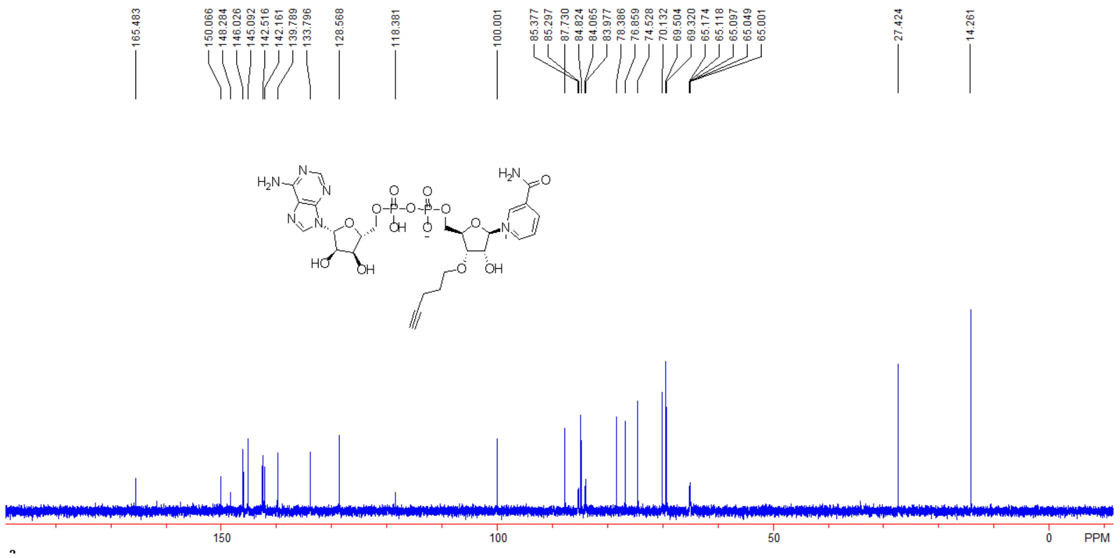

**Supplementary Figure 106**. $^{13}$C NMR spectrum of compound **4**.

**Synthesis of NAD$^+$ analogue 5 (Supplementary Figure 7).**

The compound **1-2** (1.21 g, 3.0 mmol) was dissolved in DCM followed by the addition of pyridine (711 mg, 9.0 mmol, 3 eq) and $Tf_2O$ (1.3 g, 4.5 mmol, 1.5 eq) at 0°C. The reaction mixture was allowed to warm to room temperature. After stirring at this temperature for 4 hours, the reaction mixture was diluted with EtOAc (100 mL) and quenched with water (2 mL), and the organic phase was washed with water (5×50 mL), dried over anhydrous $Na_2SO_4$, filtered and concentrated to give a residue. The residue was purified by a flash column chromatography on silica gel to afford the desired compound **5-1** (1.29 g, 80%) as a colorless oil. $^1$H NMR (400 MHz, $CDCl_3$): δ 0.98-1.10 (m, 28H, $8CH_3$+4CH), 3.36 (s, 3H, $OCH_3$), 3.87 (dd, 1H, $J$ = 12.8, 7.2 Hz, $CH_2$), 3.99-4.05 (m, 2H, $CH_2$+CH), 4.63 (dd, 1H, $J$ = 7.2, 4.4 Hz, CH), 4.93 (s, 1H, CH), 4.98 (d, 1H, $J$ = 4.4 Hz, CH) (Supplementary Figure 107); $^{13}$C NMR (100 MHz, $CDCl_3$): δ 12.68, 12.72, 13.1, 13.2, 16.68, 16.71, 16.87, 16.90, 17.27, 17.32, 17.4, 55.2, 63.8, 71.7, 81.3, 88.9, 103.8, 118.6 (q, $J$ = 317.0 Hz) (Supplementary Figure 108).

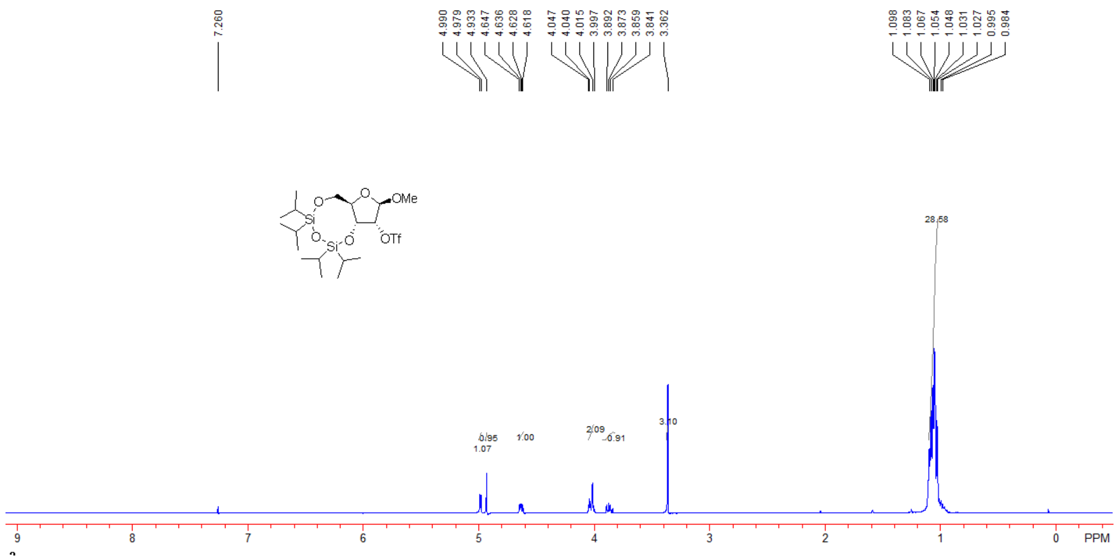


**Supplementary Figure 107**. $^1$H NMR spectrum of compound **5-1**.

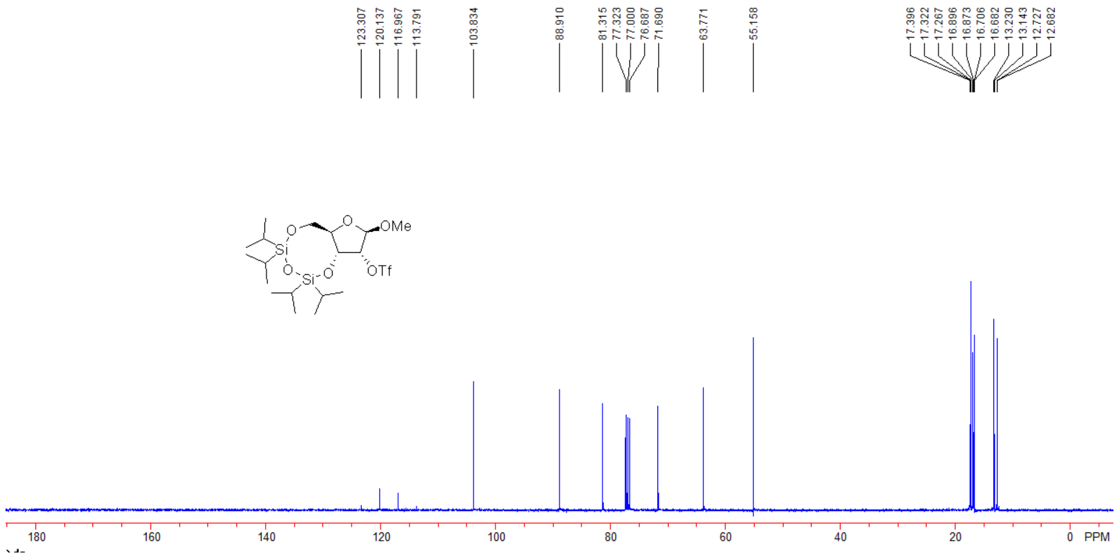


**Supplementary Figure 108**. $^{13}$C NMR spectrum of compound **5-1**.

The compound **5-1** (1.24 g, 2.3 mmol) was dissolved in DMF (20 mL) and $NaNO_2$ (793 mg, 11.5 mmol, 5 eq) was added to the mixture at r.t. Then the resulting mixture was heated to 35°C. After stirring at this temperature for 12 hours, the reaction mixture was diluted with EtOAc (100 mL), and the organic phase was washed with water (5×50 mL), dried over anhydrous $Na_2SO_4$, filtered and concentrated to give a residue. The residue was purified by a flash column chromatography on silica gel to afford the desired product **5-2** (440 mg, 47%) as a colorless oil. $^1$H NMR (400 MHz, $CDCl_3$): δ 0.99-1.11 (m, 28H, $8CH_3$+4CH), 2.25 (d, 1H, $J$ = 9.6 Hz, OH), 3.40 (s, 3H, $OCH_3$), 3.76 (dd, 1H, $J$ = 10.8, 8.8 Hz, $CH_2$), 3.83-3.87 (m, 1H, CH), 3.96 (dd, 1H, $J$ = 10.8, 3.2 Hz, $CH_2$), 4.10-4.15 (m, 1H, CH), 4.21 (dd, 1H, $J$ = 7.6, 6.0 Hz, CH), 4.74 (d, 1H, $J$ = 4.0 Hz, CH) (Supplementary Figure 109); $^{13}$C NMR (100 MHz, $CDCl_3$): δ 12.5, 12.8, 13.3, 13.4, 16.97, 16.99, 17.05, 17.38, 17.40, 17.44, 17.53, 55.2, 66.0, 78.7, 79.6, 82.1, 101.3 (Supplementary Figure 110).

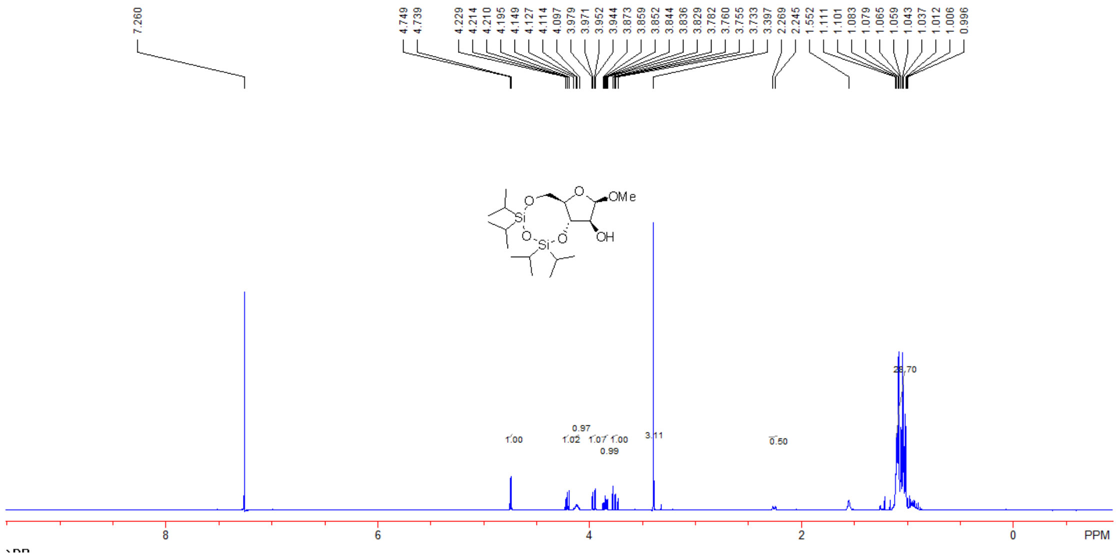


**Supplementary Figure 109**. $^{1}$H NMR spectrum of compound **5-2**.

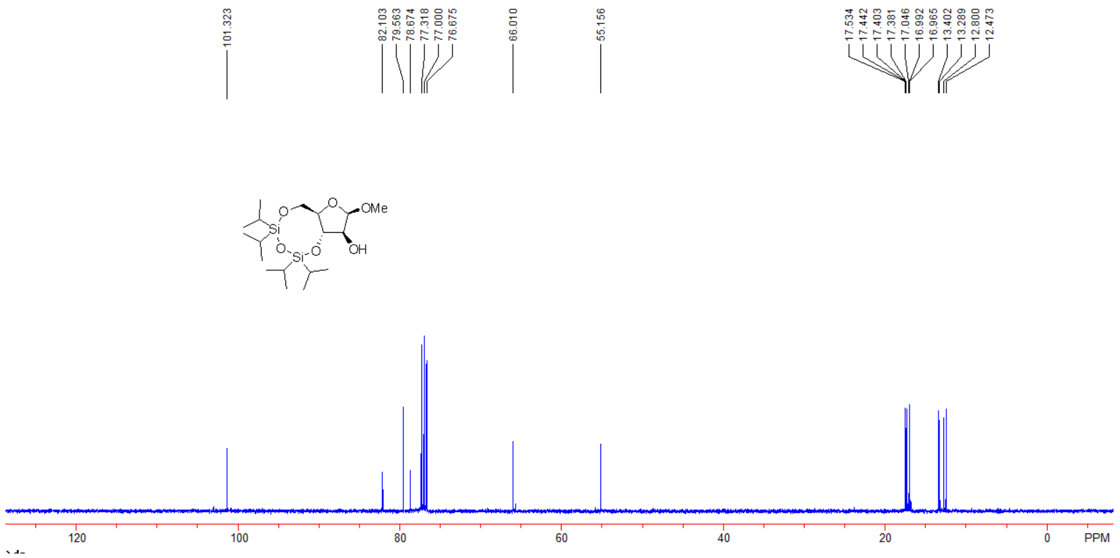


**Supplementary Figure 110**. $^{13}$C NMR spectrum of compound **5-2**.

To a stirred solution of compound **5-2** (407 mg, 1.0 mmol) in DCM (15 mL) were added pyridine (237 mg, 3.0 mmol, 3 eq) and $Tf_2O$ (433 mg, 1.5 mmol, 1.5 eq) at 0°C. The reaction mixture was allowed to warm to room temperature. After stirring at this temperature for 4 hours, the reaction mixture was diluted with EtOAc (100 mL), and the organic phase was washed with water (5×50 mL), dried over anhydrous $Na_2SO_4$, filtered and concentrated to give a residue. The residue was purified by a flash column chromatography on silica gel to afford the desired compound **5-3** (442 mg, 82%) as a colorless oil. $^{1}$H NMR (400 MHz, $CDCl_3$): δ 0.99-1.11 (m, 28H, $8CH_3$+4CH), 3.40 (s, 3H, $OCH_3$), 3.81 (dd, 1H, *J* = 10.8, 9.2 Hz, $CH_2$), 3.90-3.95 (m, 1H, CH), 3.98 (dd, 1H, *J* = 10.8, 3.2 Hz, $CH_2$), 4.70 (dd, 1H, *J* = 7.6, 5.6 Hz, CH), 4.92 (d, 1H, *J* =

4.4 Hz, CH), 4.97 (dd, 1H, $J$ = 7.6, 4.4 Hz, CH) (Supplementary Figure 111); $^{13}$C NMR (100 MHz, $CDCl_3$): δ 12.4, 12.8, 13.18, 13.24, 16.69, 16.72, 16.76, 16.77, 17.31, 17.33, 17.37, 17.48, 55.4, 66.0, 76.0, 81.3, 88.9, 99.3, 118.5 (q, $J$ = 317.9) (Supplementary Figure 112).

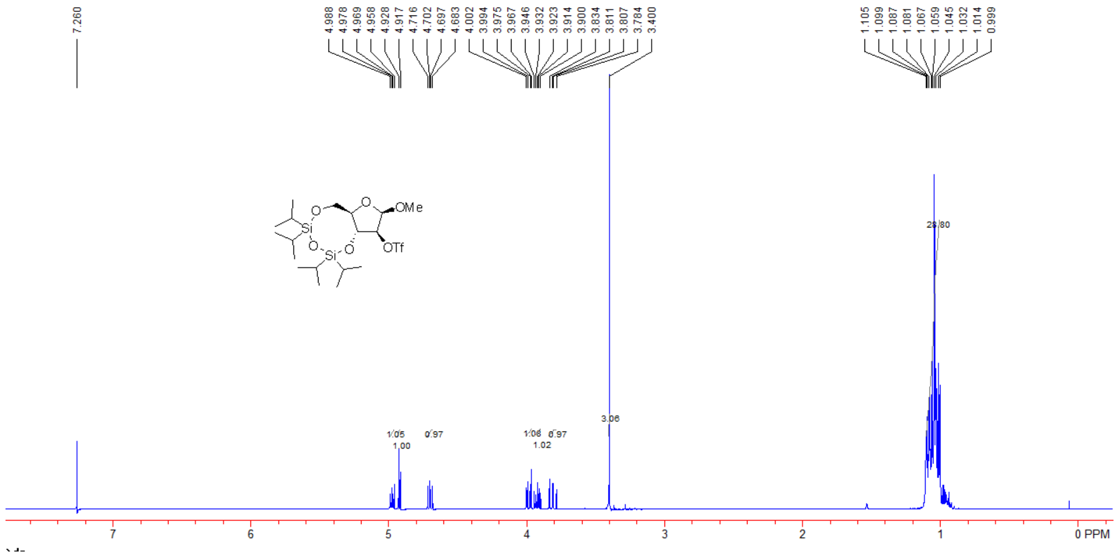


**Supplementary Figure 111**. $^1$H NMR spectrum of compound **5-3**.

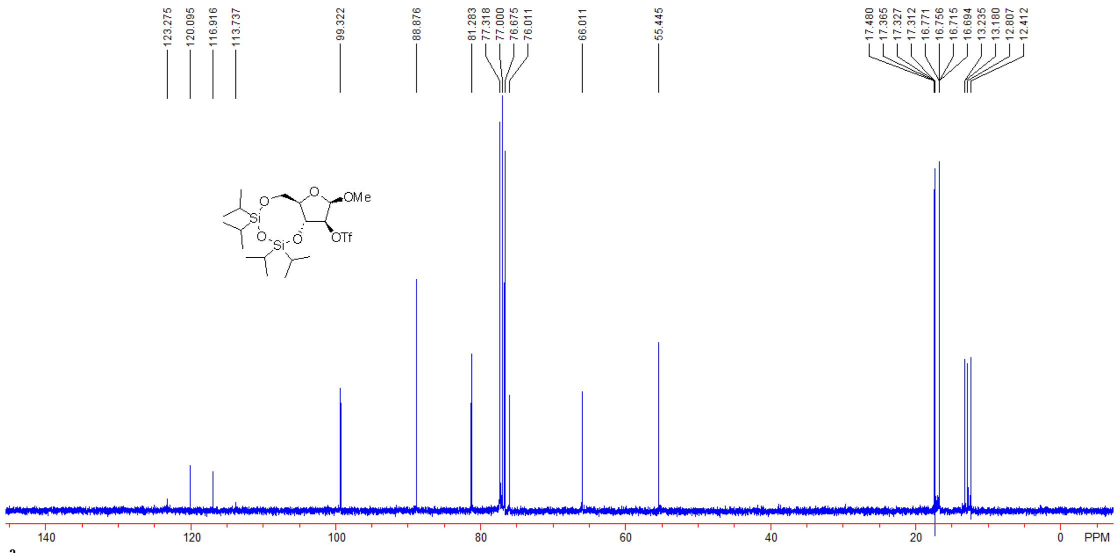


**Supplementary Figure 112**. $^{13}$C NMR spectrum of compound **5-3**.

The compound **5-3** (808 mg, 1.5 mmol) was dissolved in DMF (20 mL) and $NaN_3$ (488 mg, 7.5 mmol, 5 eq) was added to the mixture at room temperature. After stirring at 100°C for 18 hours, the reaction mixture was diluted with EtOAc (100 mL), and the organic phase was washed with water (5×50 mL), dried over anhydrous $Na_2SO_4$, filtered and concentrated to give a residue. The residue was purified by a flash column chromatography on silica gel to afford the desired

product **5-4** (376 mg, 58%) as a colorless oil. $^1$H NMR (400 MHz, $CDCl_3$): δ 1.01-1.11 (m, 28H, $8CH_3$+4CH), 3.29 (s, 3H, $OCH_3$), 3.79 (dd, 1H, *J* = 12.0, 8.4 Hz, $CH_2$), 3.88 (d, 1H, *J* = 5.2 Hz, CH), 3.99-4.04 (m, 2H, CH+$CH_2$), 4.61 (s, 1H, CH), 4.74 (dd, 1H, *J* = 7.2, 5.2 Hz, CH) (Supplementary Figure 113); $^{13}$C NMR (100 MHz, $CDCl_3$): δ 12.7, 13.2, 13.3, 16.84, 16.85, 17.1, 17.27, 17.30, 17.34, 17.36, 17.45, 54.8, 65.0, 67.0, 75.8, 81.7, 105.2 (Supplementary Figure 114).

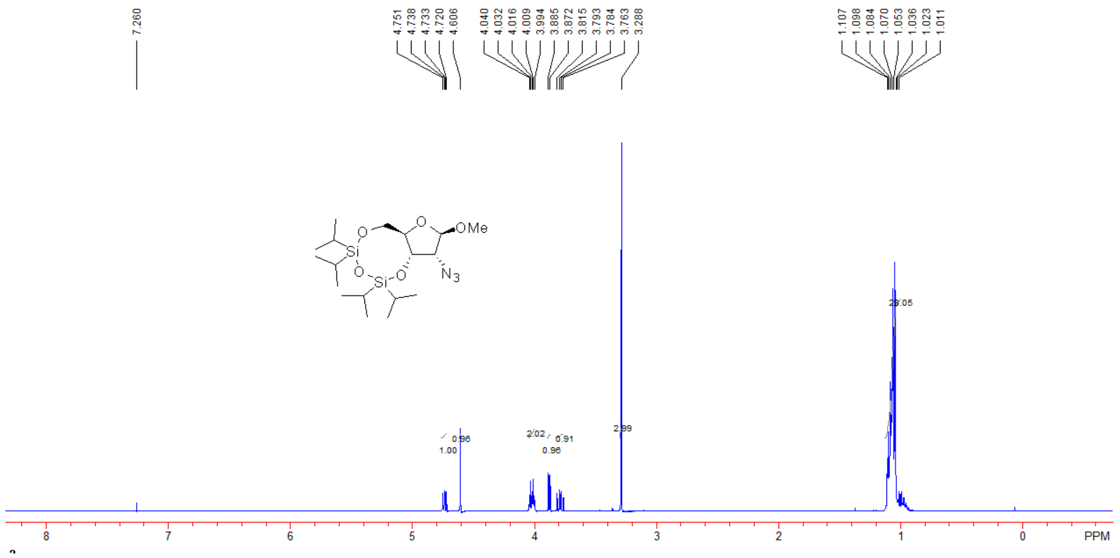

**Supplementary Figure 113**. $^1$H NMR spectrum of compound **5-4**.

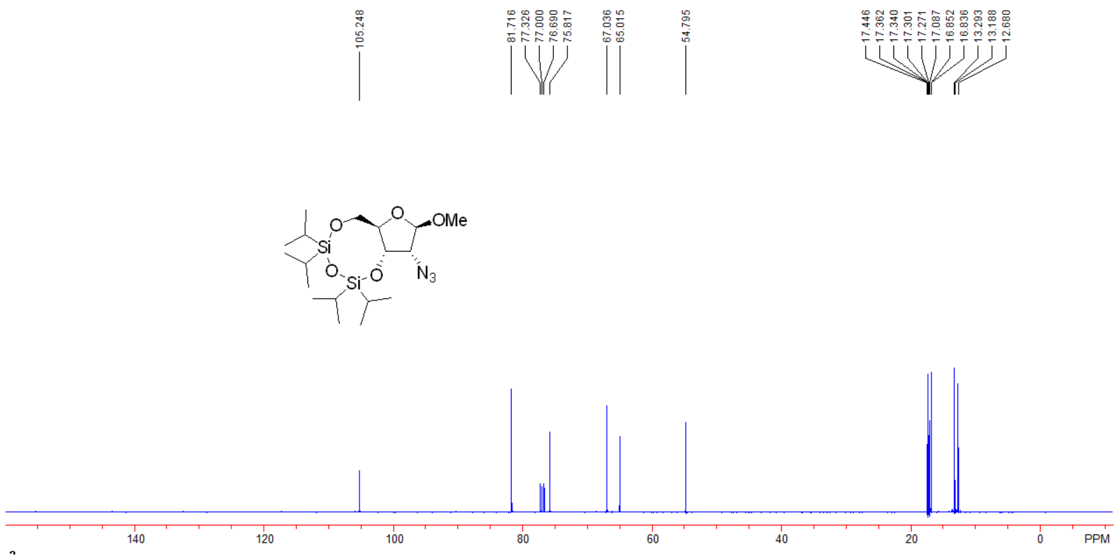

**Supplementary Figure 114**. $^{13}$C NMR spectrum of compound **5-4**.

To a 0°C solution of compound **5-4** (345 mg, 0.8 mmol) in anhydrous THF (1 mL) was added AcOH (72 mg, 1.2 mmol, 1.5 eq) followed by the addition of TBAF (1.2 mL, 1.2 mmol, 1.0 M

in THF, 1.5 eq). Then reaction mixture was allowed to warm to room temperature. After stirring at this temperature for 10 hours, the reaction mixture was concentrated under reduced pressure to give a residue. The residue was dissolved in a mixture of anhydrous DCM (10 mL) and anhydrous pyridine (10 mL) was added BzCl (277 μL, 2.4 mmol, 3 eq) at 0°C. Then the reaction mixture was allowed to warm to room temperature. After stirring for 24 hours, the reaction was quenched with MeOH (10 mL) and the mixture was concentrated under reduced pressure to give a residue. The residue was dissolved in EtOAc (50 mL), and the organic phase was washed successively with saturated aqueous $CuSO_4$ (3×50 mL), brine (50 mL), dried over anhydrous $Na_2SO_4$, filtered , concentrated and purified by a flash column chromatography on silica gel to afford the desired compound **5-5** (229 mg, 72% yield) as a colorless oil. $^1$H NMR (400 MHz, $CDCl_3$): δ 3.36 (s, 3H, $OCH_3$), 4.32 (d, 1H, $J$ = 5.2 Hz, CH), 4.48 (dd, 1H, $J$ = 13.2, 6.4 Hz, $CH_2$), 4.60-4.65 (m, 2H, CH+$CH_2$), 4.95 (s, 1H, CH), 5.72 (t, 1H, $J$ = 7.2, 5.2 Hz, CH), 7.38 (t, 2H, $J$ = 8.0 Hz, ArH), 7.46 (t, 2H, $J$ = 7.6 Hz, ArH), 7.51-7.55 (m, 1H, ArH), 7.58-7.62 (m, 1H, ArH), 8.05 (d, 2H, $J$ = 8.0 Hz, ArH), 8.08 (d, 2H, $J$ = 7.6 Hz, ArH) (Supplementary Figure 115); $^{13}$C NMR (100 MHz, $CDCl_3$): δ 55.3, 64.6, 65.3, 74.2, 78.6, 106.7, 128.3, 128.51, 128.54, 129.58, 129.63, 129.9, 133.1, 133.7 (Supplementary Figure 116).

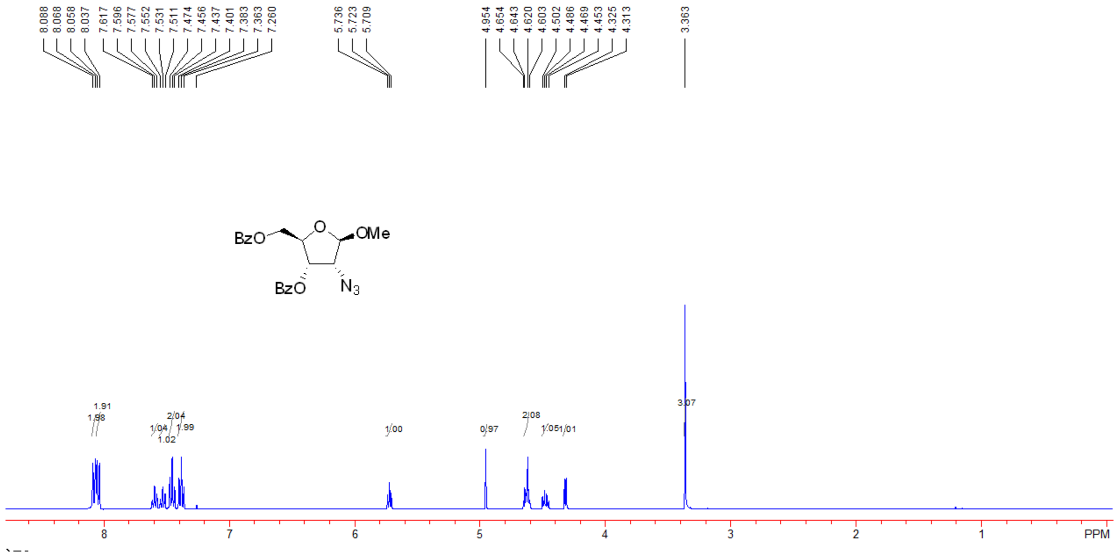


**Supplementary Figure 115**. $^1$H NMR spectrum of compound **5-5**.

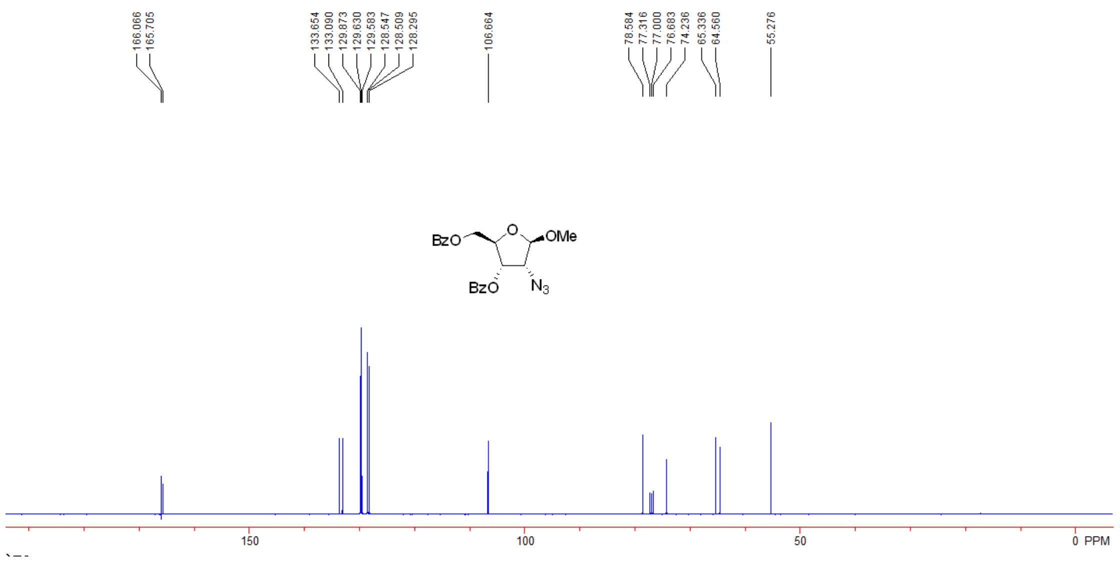


**Supplementary Figure 116**. $^{13}$C NMR spectrum of compound **5-5**.

To a stirred solution of compound **5-5** (199 mg, 0.5 mmol) in a mixture of AcOH (4 mL) and $Ac_2O$ (1.0 mL) was add *conc.* $H_2SO_4$ (40 μL) at 0°C. The resulting mixture was stirred at the same temperature until the reaction complete (monitoring by TLC, about 20 min). Then the reaction was diluted with DCM (60 mL) and the solution was added dropwise to a stirred mixture of ice and saturated aqueous $NaHCO_3$. Solid $NaHCO_3$ was added during the addition to maintain a pH of 7. The mixture was extracted with DCM (3×50 mL), and the combined organic extracts was washed with $H_2O$ (50 mL), brine (50 mL), dried over anhydrous $Na_2SO_4$, filtered, concentrated to give a residue. The residue was purified by a flash column chromatography on silica gel to afford the desired compound **5-6** (174 mg, 82% yield) as a colorless oil. $^1$H NMR (400 MHz, $CDCl_3$) of two isomers: δ 1.97 (s, 3H, $CH_3$), 2.23 (s, 3H, $CH_3$), 3.93 (dd, 1H, $J$ = 11.8 Hz, $CH_2$), 4.45-4.49 (m, 2H, $CH_2$), 4.62-4.63 (m, 2H, 2CH), 4.68-4.73 (m, 3H, 2CH+$CH_2$), 5.66 (dd, 1H, $J$ = 6.8, 2.4 Hz, CH), 5.71 (dd, 1H, $J$ = 6.8, 4.8 Hz, CH), 6.20 (s, 1H, CH), 5.58 (d, 1H, $J$ = 4.8 Hz, CH), 7.39 (t, 2H, $J$ = 8.0 Hz, ArH), 7.44-7.51 (m, 6H, ArH), 7.53-7.65 (m, 4H, ArH), 8.00 (dd, 1H, $J$ = 8.0, 0.8 Hz, ArH), 8.03-8.10 (m, 4H, ArH), 8.12-8.14 (m, 2H, ArH) (Supplementary Figure 117); $^{13}$C NMR (100 MHz, $CDCl_3$): δ 20.9, 21.2, 60.5, 63.5, 63.7, 65.0, 72.95, 72.98, 79.5, 83.3, 95.8, 98.9, 128.2, 128.4, 128.56, 128.60, 128.62, 128.8, 129.3, 129.5, 129.6, 129.7, 130.0, 133.3, 133.4, 133.8, 133.9, 165.8, 165.9, 169.1, 169.5 (Supplementary Figure 118).

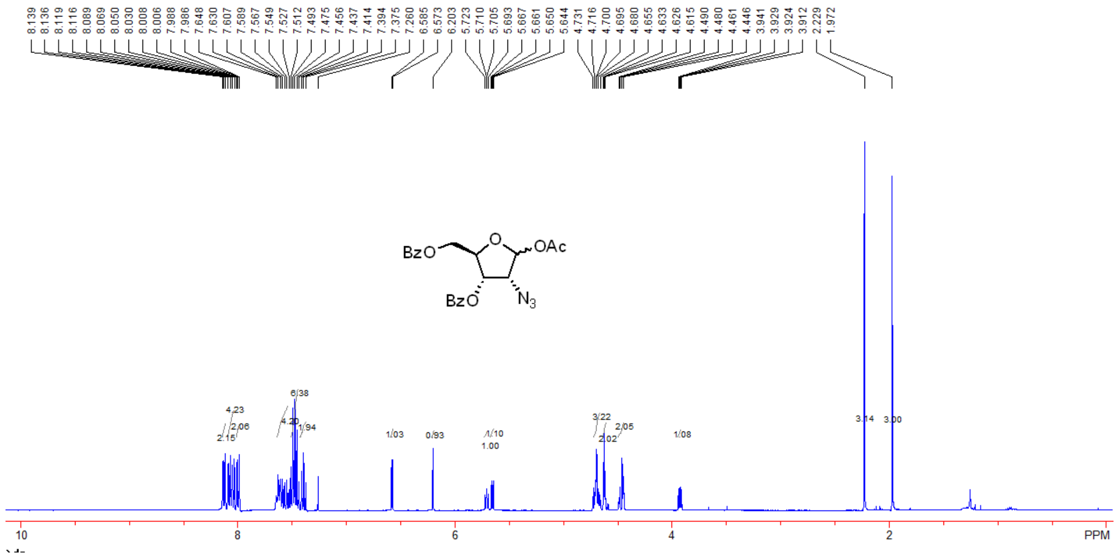


**Supplementary Figure 117**. $^{1}$H NMR spectrum of compound **5-6**.

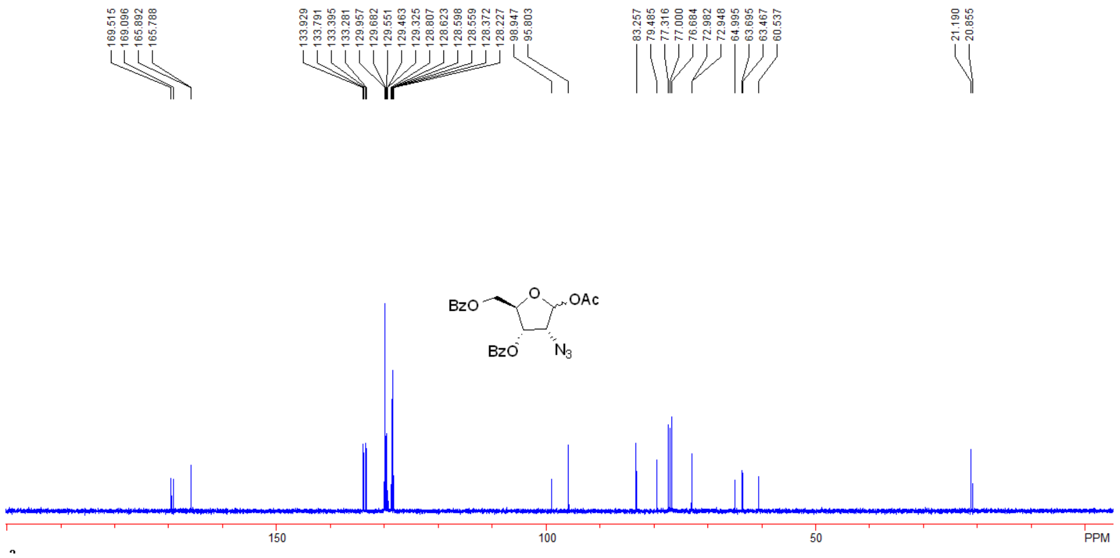


**Supplementary Figure 118**. $^{13}$C NMR spectrum of compound **5-6**.

To a stirred solution of compound **5-6** (298 mg, 0.70 mmol) and nicotinamide (128 mg, 1.05 mmol, 1.5 eq) in anhydrous $CH_3CN$ (15 mL), TMSOTf (506 μL, 2.8 mmol, 4.0 eq) was added dropwise at 0°C and the reaction mixture was stirred for 2 hours at the same temperature. Then the reaction was quenched with MeOH (1 mL) and the mixture was concentrated under reduced pressure to give a residue. The residue was purified by a flash column chromatography on silica gel to afford the crude product which was then purified via preparative HPLC (C18-A column, 150×10.0 mm, 5 μm) (mobile phase A: 0.1% formic acid (aq), mobile B: 0.1% formic acid in acetonitrile; flow rate = 2.0 ml min$^{-1}$; 0-2 min: 0-4% B, 2-4 min: 4-10% B, 4-6 min: 10-20% B, 6-12 min: 20-50% B, 12-17 min: 50-100% B, 17-20 min: 100-0% B). Fractions containing the

desired product were concentrated and lyophilized to yield the **5-7** (170 mg, 38% yield) as a colorless solid. $^{1}$H NMR (400 MHz, $CD_3OD$): δ 4.85 (dd, 1H, *J* = 12.4, 3.6 Hz, $CH_2$), 4.92 (dd, 1H, *J* = 12.4, 4.8 Hz, $CH_2$), 5.04 (t, 1H, *J* = 5.6 Hz, CH), 5.09-5.13 (m, 1H, CH), 5.96 (dd, 1H, *J* = 6.0, 4.0 Hz, CH), 6.44 (d, 1H, *J* = 5.6 Hz, CH), 7.47 (t, 2H, *J* = 8.0 Hz, ArH), 7.56 (t, 2H, *J* = 8.0 Hz, ArH), 7.61-7.65 (m, 1H, ArH), 7.68-7.73 (m, 1H, ArH), 8.00-8.03 (m, 2H, ArH), 8.15-8.18 (m, 2H, ArH), 8.27 (dd, 1H, *J* = 8.0, 6.4 Hz, ArH), 9.05-9.08 (m, 1H, ArH), 9.33 (d, 1H, *J* = 6.4 Hz, ArH), 9.60 (s, 1H, ArH) (Supplementary Figure 119); $^{13}$C NMR (100 MHz, $CD_3OD$): δ 64.5, 68.4, 73.9, 85.2, 99.5, 129.6, 129.83, 129.86, 129.89, 130.5, 130.7, 131.1, 134.8, 135.2, 136.1, 142.1, 144.0, 147.3, 164.7, 166.8, 167.6 (Supplementary Figure 120).

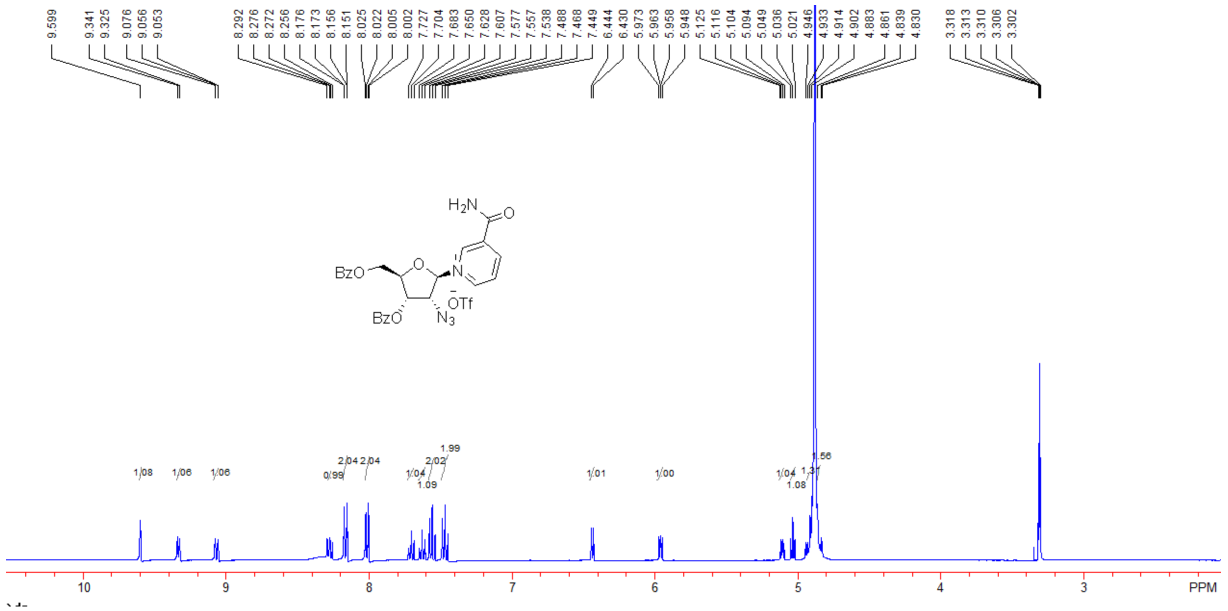


**Supplementary Figure 119**. $^{1}$H NMR spectrum of compound **5-7**.

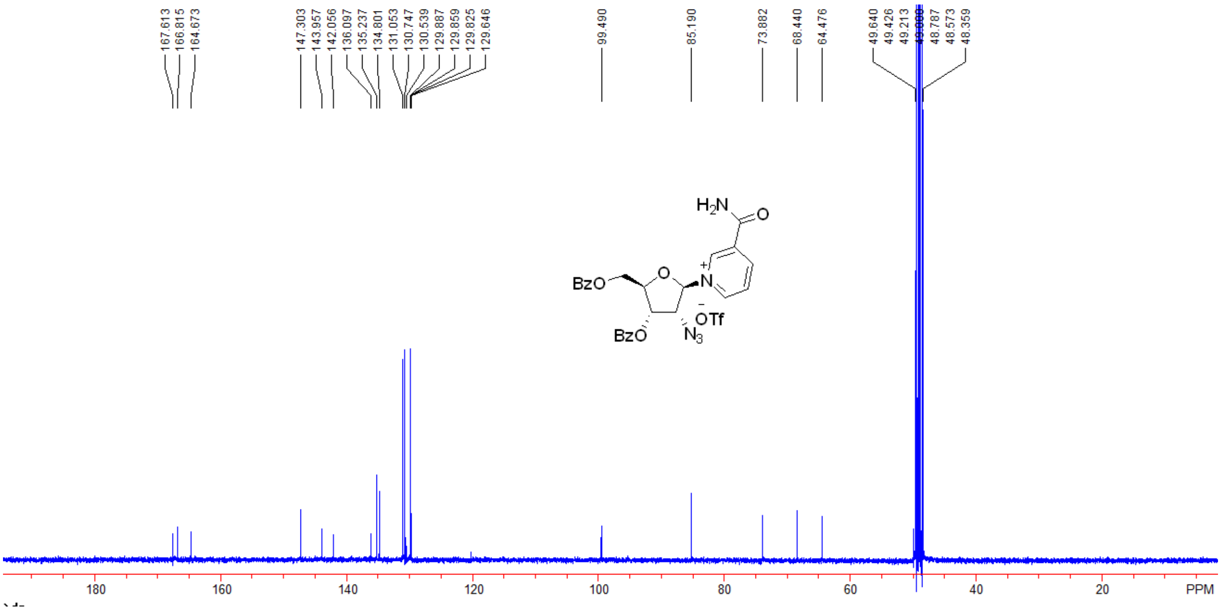


**Supplementary Figure 120**. $^{13}$C NMR spectrum of compound **5-7**.

Compound (**5-7**) (159 mg, 0.25 mmol) was dissolved in ammonia (15 mL, 7 N in MeOH) and the reaction was stirred at 4ºC for 20 hours. The reaction was concentrated under reduced pressure and the crude product was purified via preparative HPLC (C18-A column, 150×10.0 mm, 5 µm) (mobile phase A: 0.1% formic acid (aq), mobile B: 0.1% formic acid in methanol; flow rate = 2.0 ml min$^{-1}$; 0-2 min: 0-4% B, 2-4 min: 4-10% B, 4-6 min: 10-20% B, 6-12 min: 20-50% B, 12-14 min: 50-0% B. Fractions containing the desired product were concentrated and lyophilized to yield the NR5 (55 mg, 51% yield) as a colorless solid. $^{1}$H NMR (400 MHz, $D_2O$): δ 3.90 (dd, 1H, $J$ = 13.2, 3.2 Hz, $CH_2$), 4.07 (dd, 1H, $J$ = 13.2, 2.4 Hz, $CH_2$), 4.44-4.47 (m, 1H, CH), 4.62-4.66 (m, 2H, 2CH), 6.28 (d, 1H, $J$ = 3.2 Hz, CH), 8.27 (dd, 1H, $J$ = 8.0, 6.0 Hz, ArH), 8.98 (dt, 1H, $J$ = 8.0, 1.6 Hz, ArH), 9.29 (d, 1H, $J$ = 6.0 Hz, ArH), 9.65 (d, 1H, $J$ = 1.6 Hz, ArH) (Supplementary Figure 121); $^{13}$C NMR (100 MHz, $D_2O$): δ 59.5, 68.2, 69.9, 87.4, 98.1, 119.5 (q, $J$ = 314.4 Hz), 128.4, 134.0, 140.6, 142.8, 145.9, 165.6 (Supplementary Figure 122).

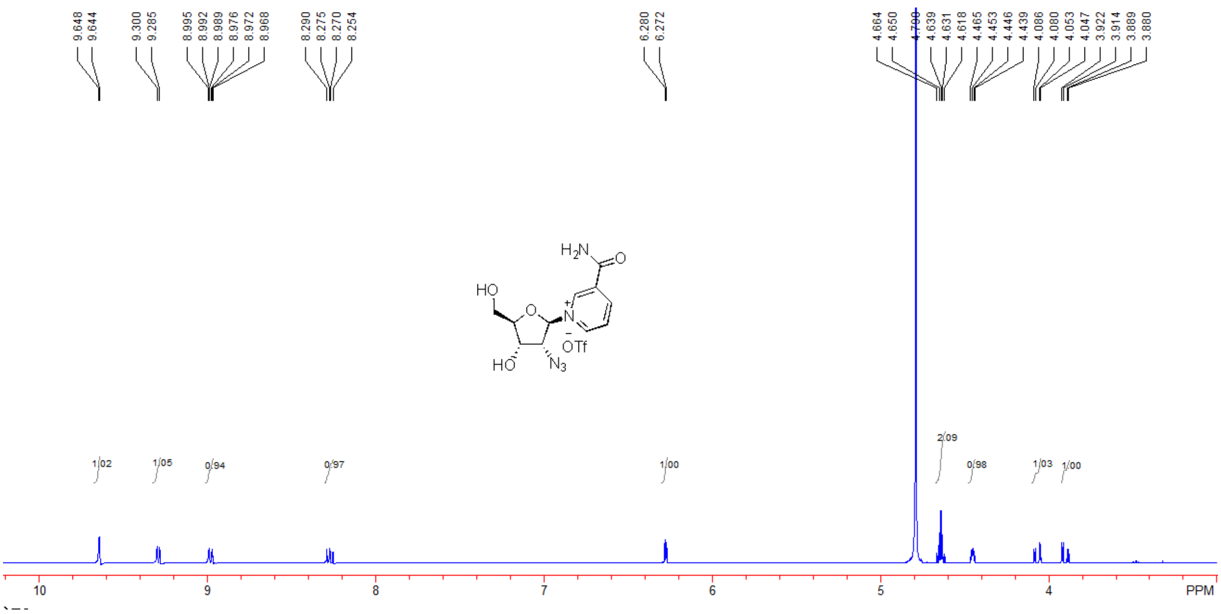


**Supplementary Figure 121**. $^{1}H$ NMR spectrum of compound NR5.

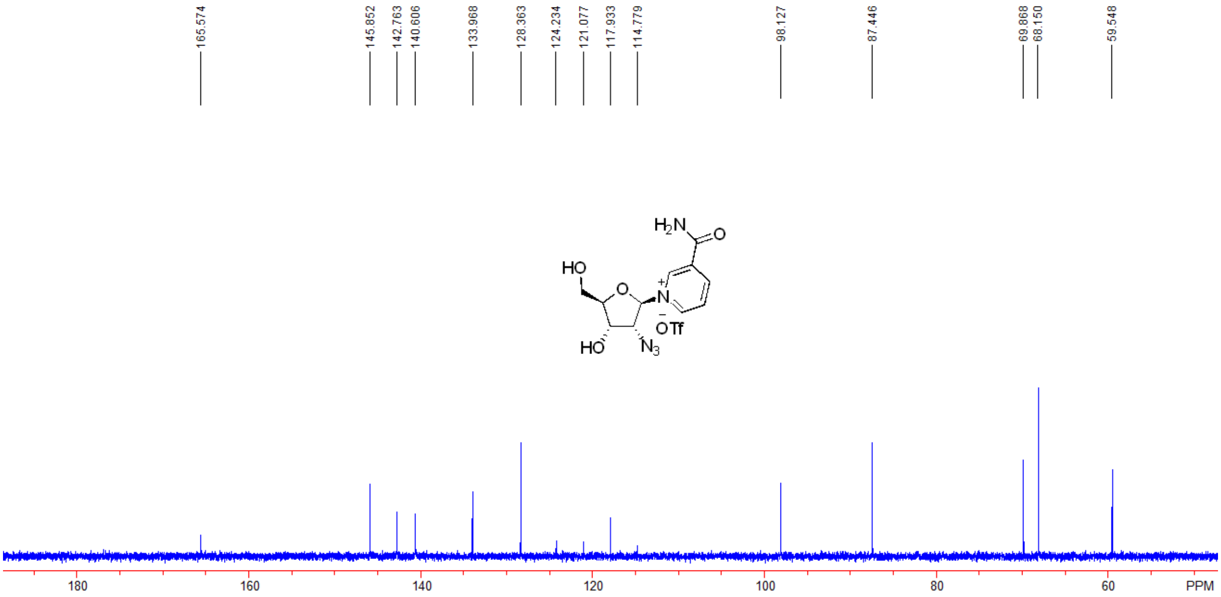


**Supplementary Figure 122**. $^{13}C$ NMR spectrum of compound NR5.

To a stirred solution of compound NR5 (52 mg, 0.12 mmol) in trimethylphosphate (1.5 mL) was added $P(O)Cl_3$ (78 μL, 0.84 mmol, 7 eq) at 0°C and the resulting mixture was stirred at 0°C for 6 hours. A few drops of $H_2O$ were then added to quench the reaction. Trimethylphosphate was removed by extraction with methylene chloride (3×20 ml). The aqueous layer was concentrated *in vacuo* and the crude product was purified via preparative HPLC (C18-A column, 150×10.0 mm, 5 μm) (mobile phase A: 0.1% formic acid (aq), mobile B: 0.1% formic acid in methanol; flow rate = 2.0 ml min$^{-1}$; 0-2 min: 0-4% B, 2-4 min: 4-10% B, 4-6 min: 10-

20% B, 6-12 min: 20-50% B, 12-14 min: 50-0% B. Fractions containing the desired product were concentrated and lyophilized to yield the NMN5 (23 mg, 54% yield) as a colorless solid. $^{1}H$ NMR (400 MHz, $D_2O$): δ 4.14 (ddd, 1H, $J$ = 12.0, 4.8, 2.0 Hz, $CH_2$), 4.33 (ddd, 1H, $J$ = 12.0, 4.0, 2.4 Hz, $CH_2$), 4.62-4.65 (m, 1H, CH), 4.69 (t, 1H, $J$ = 5.2 Hz, CH), 4.74 (dd, 1H, $J$ = 5.2, 3.2 Hz, CH), 6.25 (d, 1H, $J$ = 5.6 Hz, CH), 8.30 (dd, 1H, $J$ = 8.4, 6.4 Hz, ArH), 8.99 (dt, 1H, $J$ = 8.4, 1.2 Hz, ArH), 9.31 (d, 1H, $J$ = 6.4 Hz, ArH), 9.49 (d, 1H, $J$ = 1.2 Hz, ArH) (Supplementary Figure 123); $^{13}C$ NMR (100 MHz, $D_2O$): δ 63.7 (d, $J$ = 4.7 Hz), 68.2, 71.1, 87.4 (d, $J$ = 9.4 Hz), 98.3, 128.5, 134.0, 140.1, 142.6, 146.2, 165.6 (Supplementary Figure 124).

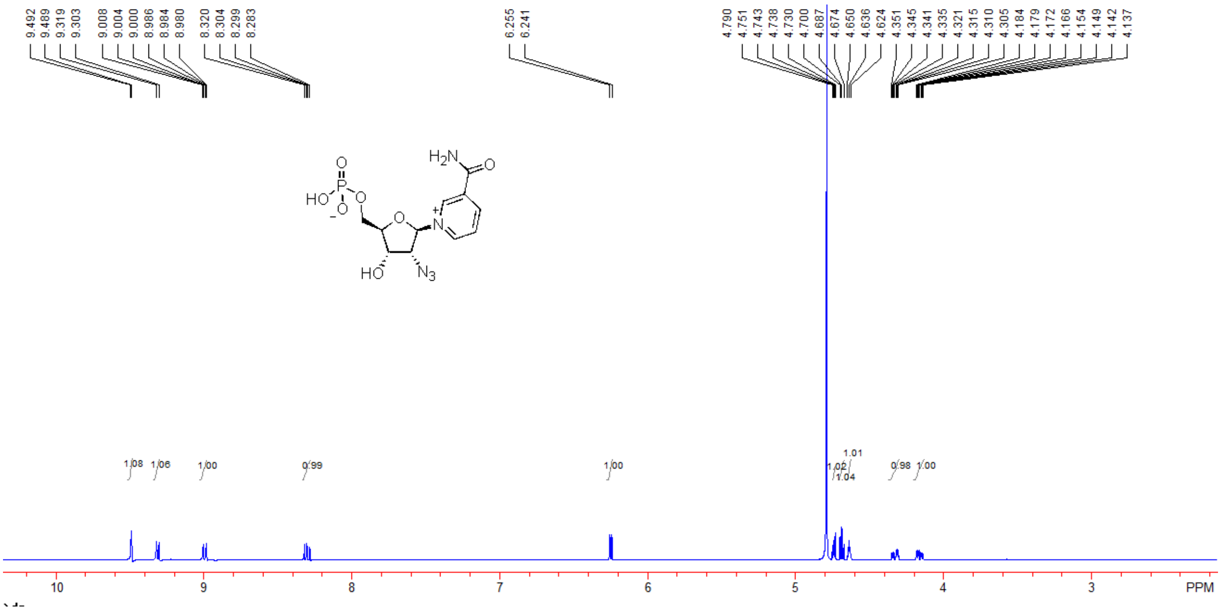


**Supplementary Figure 123**. $^{1}H$ NMR spectrum of compound NMN5.

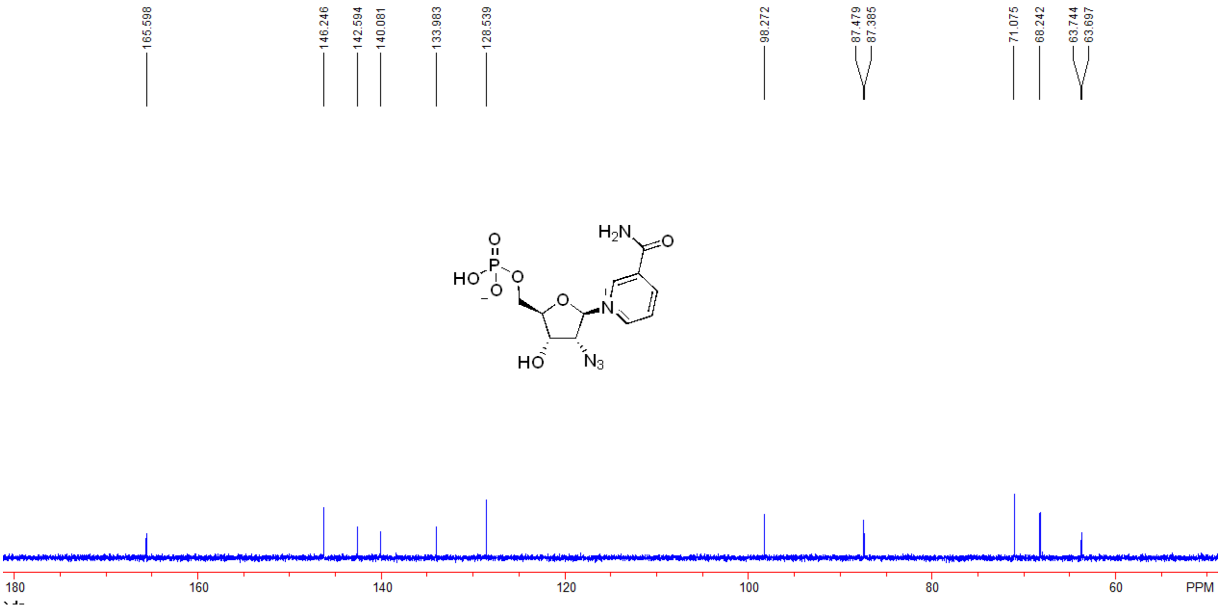


**Supplementary Figure 124**. $^{13}C$ NMR spectrum of compound NMN5.

To a stirred solution of adenosine 5′-monophosphate (5′-AMP) (52 mg, 0.15 mmol, 1.5 eq) in dried DMF (2 mL) were added 1,1-carbonyldiimidazole (CDI) (63 mg, 0.50 mmol, 5 eq) and triethylamine (23 µL, 0.16 mmol. 1.6 eq). The reaction mixture was stirred at room temperature for 8 hours, and then quenched with 0.20 ml dried methanol. The solvent was then removed under vacuum and the residue was coevaporated 3 times each with 1.00 ml of dried DMF. The activated 5′-AMP was dissolved in dried DMF (1 mL) and compound NMN5 (36 mg, 0.10 mmol) was added. After stirring at room temperature for 4 days, $H_2O$ (5 mL) was added to quench the reaction at 0°C. The resulting mixture was continued stirring at room temperature for 24 hours. The reaction was then concentrated *in vacuo* and the crude product was purified via preparative HPLC (C18-A column, 150×10.0 mm, 5 µm) (mobile phase A: 0.1% formic acid (aq), mobile B: 0.1% formic acid in acetonitrile; flow rate = 2.0 ml min$^{-1}$; 0-2 min: 0-4% B, 2-4 min: 4-10% B, 4-6 min: 10-20% B, 6-12 min: 20-50%, 12-14 min: 50-0% B). Fractions containing the desired product were concentrated and lyophilized to yield the $NAD^+$ analogue **5** (29 mg, 42% yield) as a colorless solid. $^1H$ NMR (400 MHz, $D_2O$): δ 4.18-4.27 (m, 3H, $CH_2$+$CH_2$), 4.38-4.42 (m, 2H, $CH_2$+CH), 4.50-4.52 (m, 1H, CH), 4.58 (br, 1H, CH), 4.73-4.78 (m, 2H, 2CH), 6.13 (d, 1H, $J$ = 5.2 Hz, CH), 6.20 (d, 1H, $J$ = 5.6 Hz, CH), 8.29 (dd, 1H, $J$ = 8.0, 6.4 Hz, ArH), 8.39 (s, 1H, ArH), 8.59 (s, 1H, ArH), 8.93-8.96 (m, 1H, ArH), 9.29 (d, 1H, $J$ = 6.4 Hz, ArH), 9.45 (s, 1H, ArH) (Supplementary Figure 125); $^{13}C$ NMR (100 MHz, $D_2O$): δ

64.4, 65.1, 68.1, 70.2, 70.8, 74.4, 83.9-84.1 (m), 87.06-87.15 (m), 87.6, 98.2, 128.7, 133.9, 140.9, 142.7, 145.6, 146.3, 150.5, 165.3 (Supplementary Figure 126); HRMS (ESI) for $C_{21}H_{25}N_{10}O_{13}P_2Na_2^{+1}$ $(M+2Na-H)^+$: Calcd.: 733.0873 Da; Obs: 733.0887 Da.

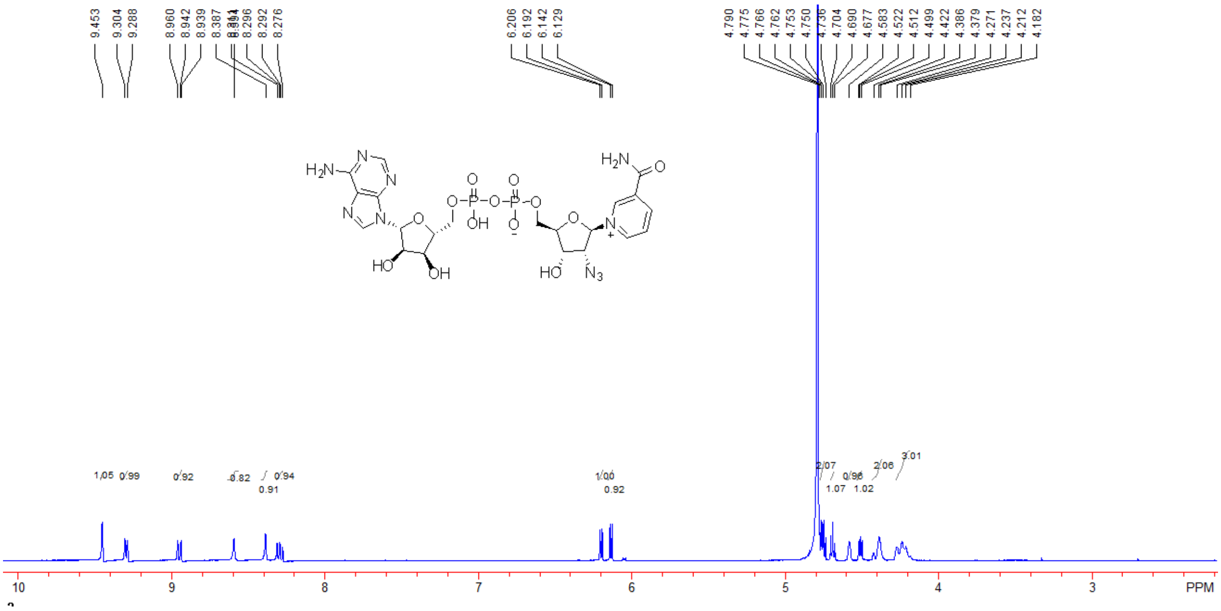


**Supplementary Figure 125**. $^{1}$H NMR spectrum of compound **5**.

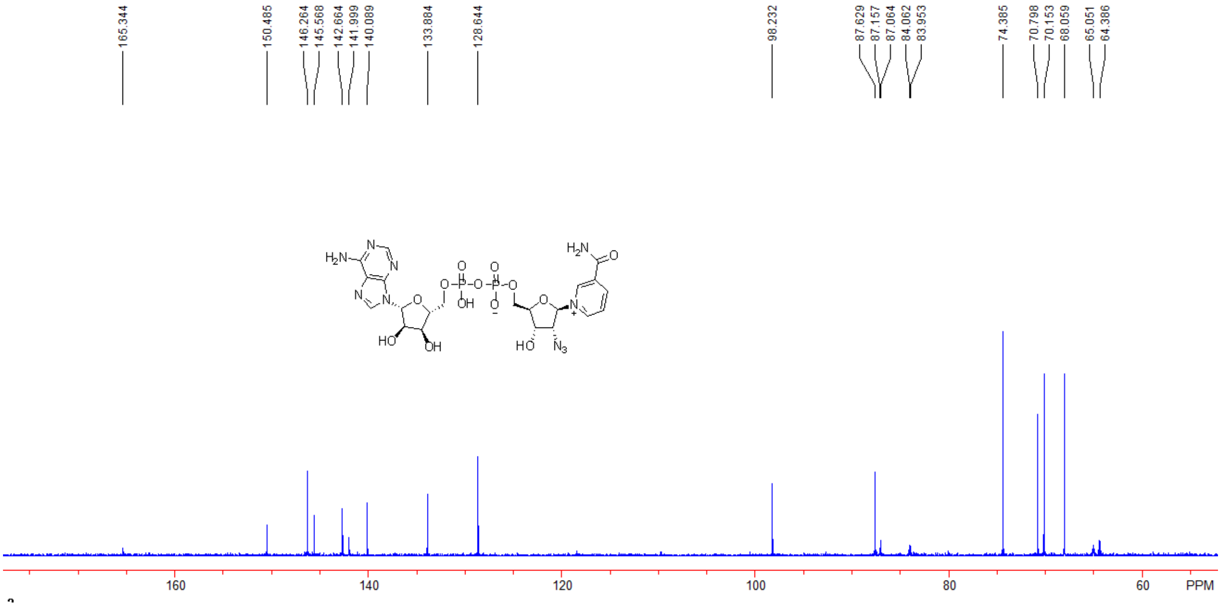


**Supplementary Figure 126**. $^{13}$C NMR spectrum of compound **5**.

**Synthesis of $NAD^+$ analogue 6 (Supplementary Figure 8).**

To a stirred solution of compound **6-2** (prepared according to a reported method[1]) (499 mg, 1.7 mmol) in a mixture of DCM (10 mL) and anhydrous pyridine (10 mL) was added BzCl (592 μL, 5.1 mmol, 3 eq) at 0°C. Then the reaction mixture was allowed to warm to room temperature. After stirring for 24 hours, the reaction was quenched with MeOH (10 mL) and the mixture was concentrated under reduced pressure to give a residue. The residue was dissolved in EtOAc (50 mL), and the organic phase was washed successively with saturated aqueous $CuSO_4$ (3×50 mL), brine (50 mL), dried over anhydrous $Na_2SO_4$, filtered, concentrated and purified by a flash column chromatography on silica gel to afford the desired compound **6-3** (507 mg, 75%) as a colorless oil. $^1$H NMR (400 MHz, $CDCl_3$): δ 3.36 (s, 3H, $OCH_3$), 4.34 (dd, 1H, $J$ = 7.2, 4.4 Hz, CH), 4.46-4.50 (m, 2H, $CH_2$+CH), 4.66 (dd, 1H, $J$ = 13.2, 5.2 Hz, $CH_2$), 5.05 (s, 1H, CH), 5.51 (d, 1H, $J$ = 4.4 Hz, CH), 7.45-7.50 (m, 4H, ArH), 7.57-7.64 (m, 2H, ArH), 8.08-8.13 (m, 4H, ArH) (Supplementary Figure 127); $^{13}$C NMR (100 MHz, $CDCl_3$): δ 55.3, 61.2, 64.2, 76.7, 78.9, 106.1, 128.5, 128.6, 128.9, 129.6, 129.8, 130.0, 133.3, 133.7, 165.4, 166.2 (Supplementary Figure 128).

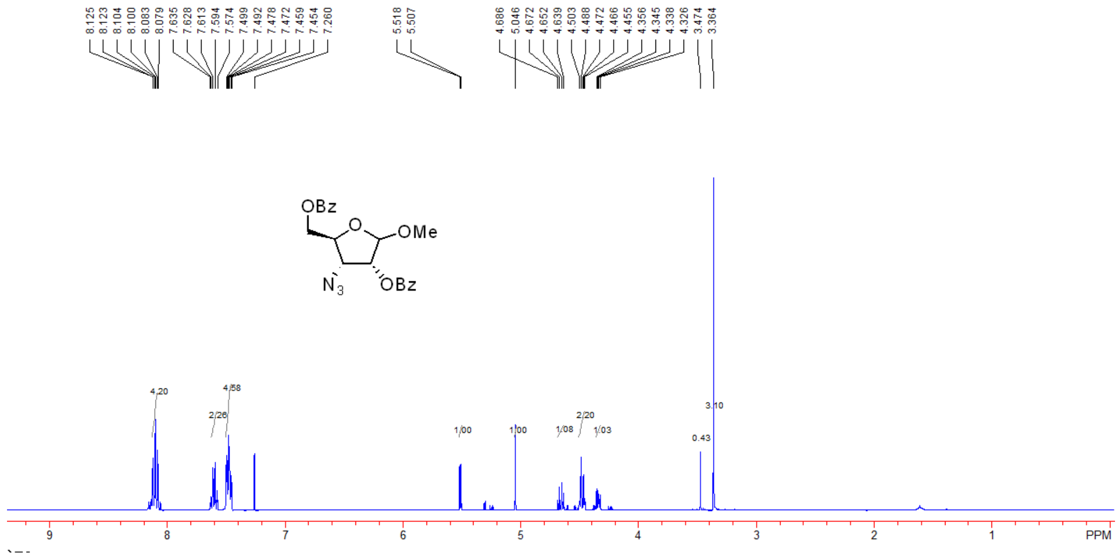


**Supplementary Figure 127**. $^1$H NMR spectrum of compound **6-3**.

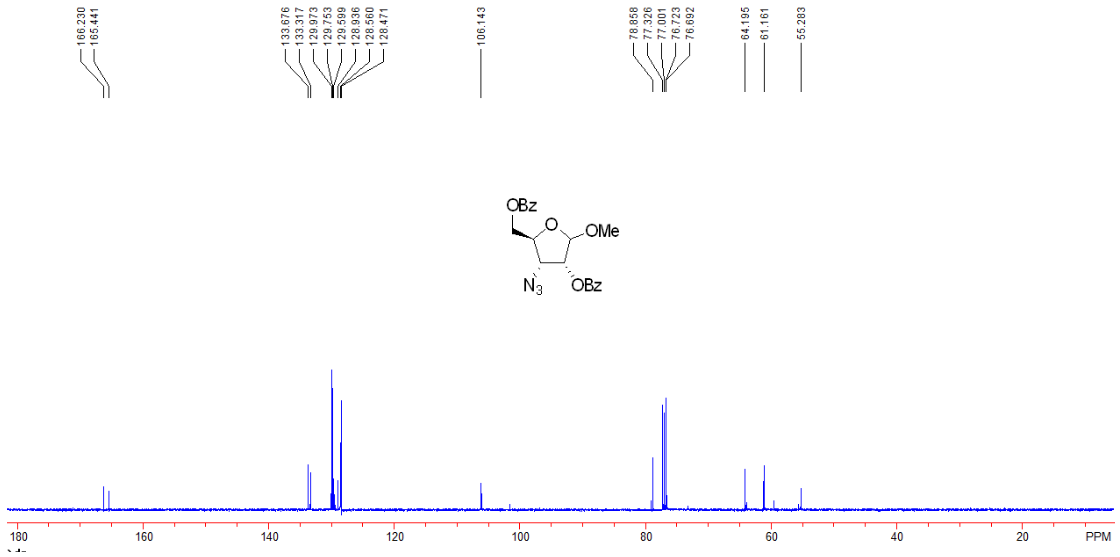


**Supplementary Figure 128**. $^{13}$C NMR spectrum of compound **6-3**.

To a stirred solution of compound **6-3** (477 mg, 1.2 mmol) in a mixture of AcOH (4 mL) and $Ac_2O$ (1.0 mL) was add *conc.* $H_2SO_4$ (100 µL) at 0ºC. The resulting mixture was stirred at the same temperature until the reaction complete (monitoring by TLC, about 20 min). Then the reaction was diluted with DCM (60 mL) and the solution was added dropwise to a stirred mixture of ice and saturated aqueous $NaHCO_3$. Solid $NaHCO_3$ was added during the addition to maintain a pH of 7. The mixture was extracted with DCM (3×50 mL), and the combined organic extracts was washed with $H_2O$ (50 mL), brine (50 mL), dried over anhydrous $Na_2SO_4$, filtered, concentrated to give a residue. The residue was purified by a flash column chromatography on silica gel to afford the desired compound **6-4** (408 mg, 80%) as a colorless oil. $^{1}$H NMR (400 MHz, $CDCl_3$) of one isomer: δ 1.95 (s, 3H, $CH_3$), 4.37 (dd, 1H, $J$ = 8.4, 4.4 Hz, CH), 4.47-4.52 (m, 2H, CH+$CH_2$), 4.73 (dd, 1H, $J$ = 13.2, 4.4 Hz, $CH_2$), 5.62 (d, 1H, $J$ = 4.4 Hz, CH), 6.32 (s, 1H, CH), 7.44-7.51 (m, 4H, ArH), 7.58-7.65 (m, 2H, ArH), 8.07-8.12 (m, 4H, ArH) (Supplementary Figure 129); $^{13}$C NMR (100 MHz, $CDCl_3$): δ 20.8, 60.5, 63.2, 76.2, 80.0, 98.2, 128.5, 128.6, 129.5, 129.7, 129.8, 130.0, 133.5, 133.9, 165.2, 166.0, 168.8 (Supplementary Figure 130).

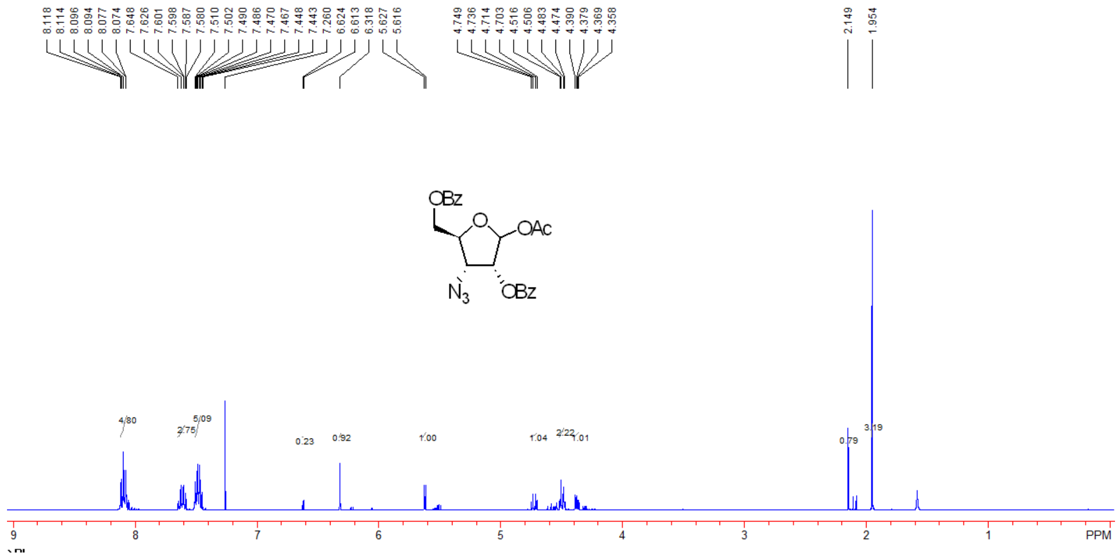

**Supplementary Figure 129**. $^1$H NMR spectrum of compound **6-4**.

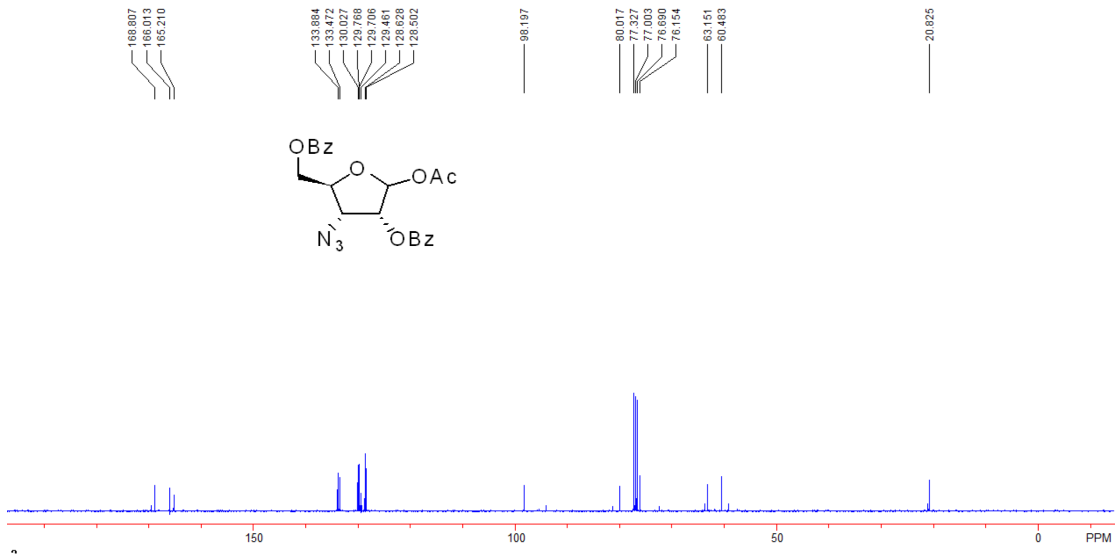

**Supplementary Figure 130**. $^{13}$C NMR spectrum of compound **6-4**.

Compound **6-4** (298 mg, 0.70 mmol) was dissolved in toluene (10 mL) and cooled to 0$^o$C. HBr (33% (wt) in acetic acid) (257 mg, 1.05 mmol, 1.5 eq) was added dropwise and the reaction was stirred at 0$^o$C for 5 hours. After the starting material was consumed, the reaction was concentrated under reduced pressure to give a residue. The residue was azeotroped with toluene (3×20 mL) to remove remaining acetic acid and dried *in vacuo*. The crude product and nicotinamide (103 mg, 0.84 mmol, 1.2 eq) was dissolved in $CH_3CN$ (20 mL). The reaction was stirred under Ar gas at room temperature for 24 hours. The reaction was concentrated *in vacuo* (the temperature was kept below 35$^o$C) and purified by a flash column chromatography on silica gel to afford the compound **6-5** (279 mg, 70%) as a colorless solid. $^1$H NMR (400 MHz,

CD3OD): δ 4.77 (m, 1H, CH), 4.89-4.95 (m, 3H, CH+CH2), 6.03-6.04 (m, 1H, CH), 6.69 (d, 1H, *J* = 1.6 Hz, CH), 7.49-7.53 (m, 2H, ArH), 7.56-7.60 (m, 2H, ArH), 7.63-7.68 (m, 1H, ArH), 7.70-7.75 (m, 1H, ArH), 8.06-8.09 (m, 2H, ArH), 8.19-8.21 (m, 2H, ArH), 8.24 (dd, 1H, *J* = 8.0, 6.4 Hz, ArH), 9.03-9.06 (m, 1H, ArH), 9.41 (d, 1H, *J* = 6.4 Hz, ArH), 9.66 (s, 1H, ArH) (Supplementary Figure 131); $^{13}$C NMR (100 MHz, CD3OD): δ 60.3, 63.8, 79.9, 84.3, 99.3, 129.56, 129.60, 129.9, 130.0, 130.5, 130.8, 131.2, 134.9, 135.5, 136.0, 142.3, 143.9, 147.0, 164.8, 167.3, 167.5 (Supplementary Figure 132).

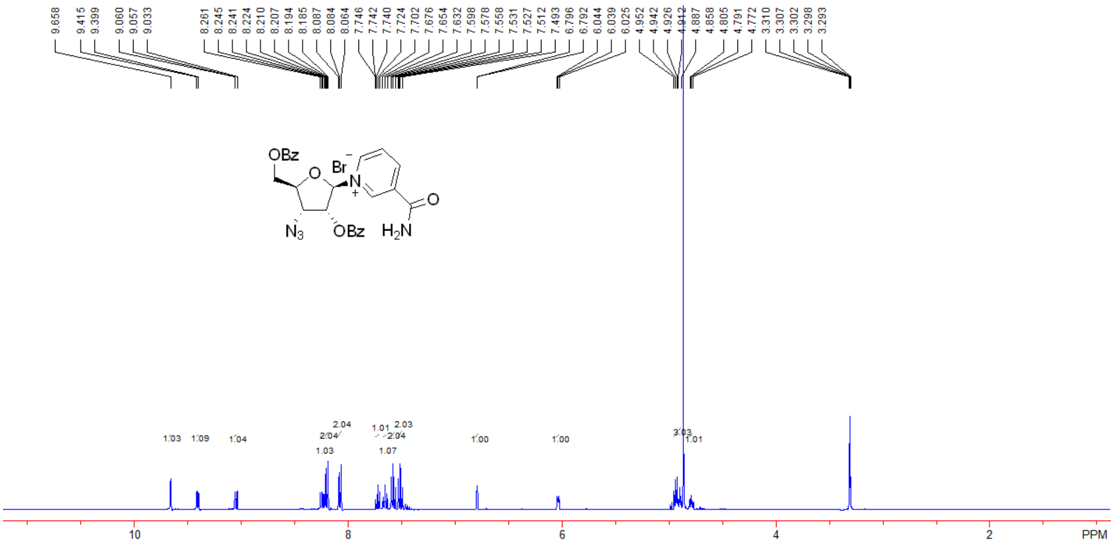


**Supplementary Figure 131**. $^{1}$H NMR spectrum of compound **6-5**.

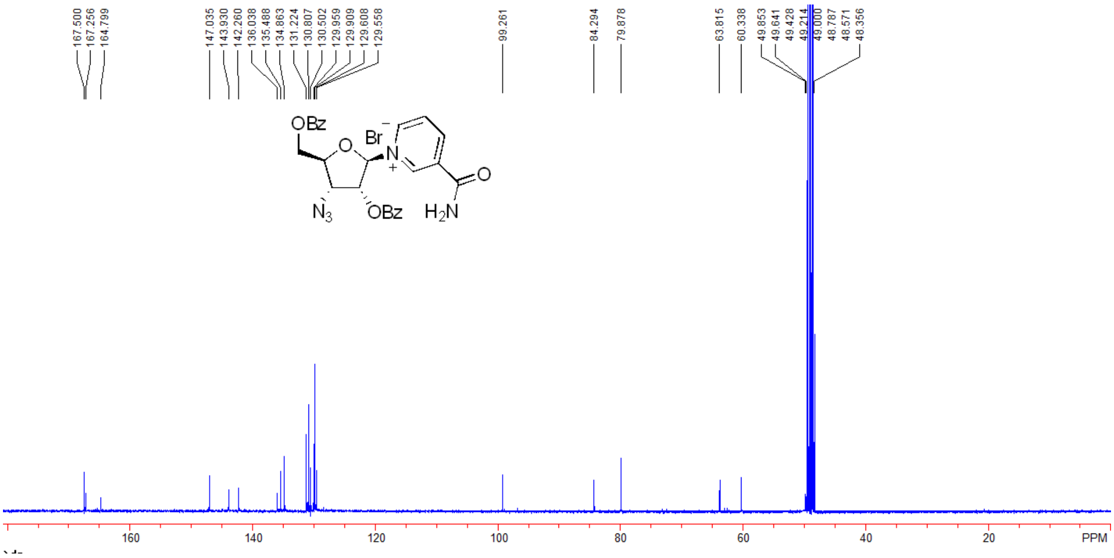


**Supplementary Figure 132**. $^{13}$C NMR spectrum of compound **6-5**.

Compound **6-5** (256 mg, 0.45 mmol) was dissolved in ammonia (18 mL, 7 N in MeOH) and the reaction was stirred at -10°C for 48 hours. The reaction was concentrated under reduced pressure and the crude product was dissolved in MeOH (0.5 mL). Addition of ethyl ether (10 mL) resulted in ppt of the desired product. The procedure was repeated four times to yield the desired product NR6 (102 mg, 63%) as a colorless solid. $^1$H NMR (400 MHz, $D_2O$): δ 3.91 (dd, 1H, *J* = 13.2, 2.8 Hz, $CH_2$), 4.08 (dd, 1H, *J* = 13.2, 2.8 Hz, $CH_2$), 4.35 (t, 1H, *J* = 5.2 Hz, CH), 4.50-4.52 (m, 1H, CH), 4.79 (m, 1H, CH, overlap with solvent residue peak), 6.27 (d, 1H, *J* = 4.0 Hz, CH), 8.28 (dd, 1H, *J* = 8.0, 6.8 Hz, ArH), 8.98 (d, 1H, *J* = 8.0 Hz, ArH), 9.27 (d, 1H, *J* = 6.8 Hz, ArH), 9.61 (s, 1H, ArH) (Supplementary Figure 133); $^{13}$C NMR (100 MHz, $D_2O$): δ 60.0, 60.7, 77.6, 85.5, 99.5, 128.3, 133.9, 140.3, 142.6, 145.7, 165.7 (Supplementary Figure 134).

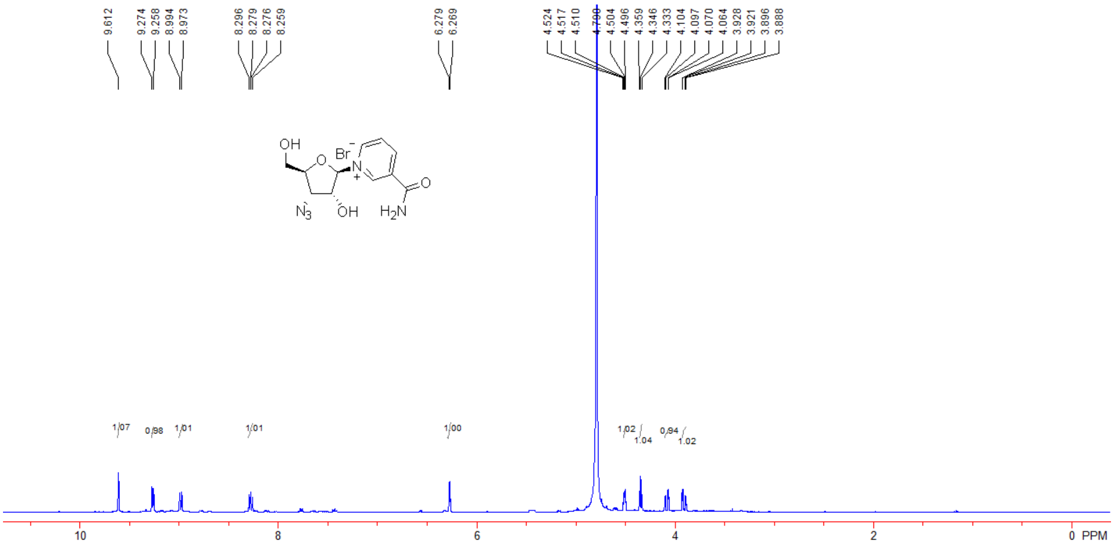


**Supplementary Figure 133**. $^1$H NMR spectrum of compound NR6.

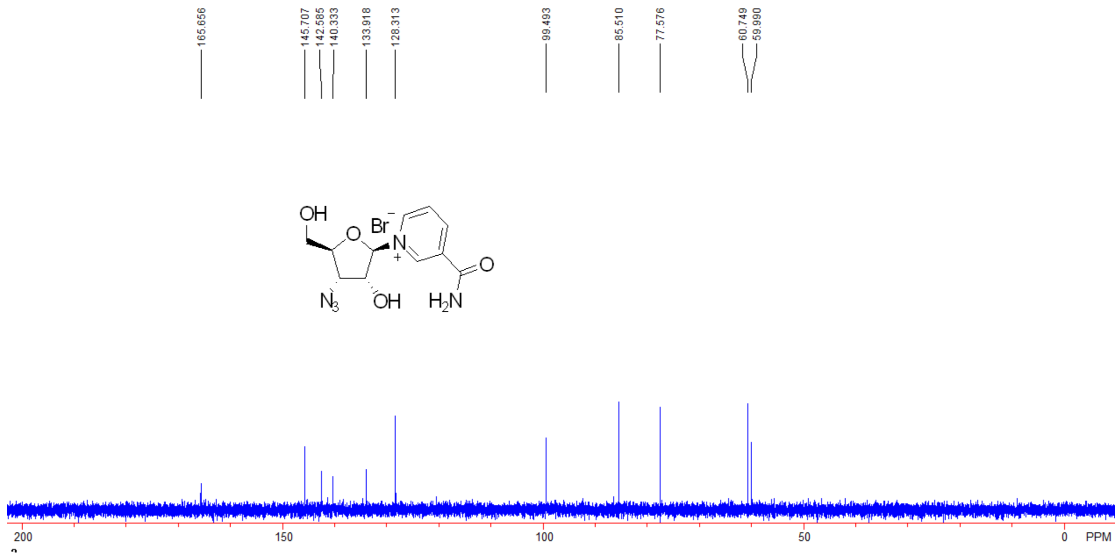


**Supplementary Figure 134**. $^{13}$C NMR spectrum of compound NR6.

To a stirred solution of compound NR6 (97 mg, 0.27 mmol) in trimethylphosphate (2 mL) was added $P(O)Cl_3$ (175 µL, 1.89 mmol, 7 eq) at 0°C and the resulting mixture was stirred at 0°C for 6 hours. A few drops of $H_2O$ were then added to quench the reaction. Trimethylphosphate was removed by extraction with ethyl ether (3×20 ml). The remaining trimethylphosphate was removed by a second extraction with THF (5 ml). The aqueous layer was concentrated *in vacuo* and the crude product was dissolved in MeOH (0.5 mL). Addition of ethyl ether (10 mL) resulted in ppt of the desired product. The procedure was repeated three times to yield the desired product NMN6 (70 mg, 72%) as a colorless solid**.** $^1$H NMR (400 MHz, $D_2O$): δ 4.15 (ddd, 1H, $J$ = 12.0, 4.8, 2.4 Hz, $CH_2$), 4.32 (ddd, 1H, $J$ = 12.0, 4.4, 2.4 Hz, $CH_2$), 4.49 (dd, 1H, $J$ = 5.6, 2.8 Hz, CH), 4.64 (t, 1H, $J$ = 2.4 Hz, CH), 4.83 (t, 1H, $J$ = 5.6 Hz, CH, overlap with solvent residue peak), 6.22 (d, 1H, $J$ = 5.6 Hz, CH), 8.29 (dd, 1H, $J$ = 8.0, 6.0 Hz, ArH), 8.98 (d, 1H, $J$ = 8.0 Hz, ArH), 9.26 (d, 1H, $J$ = 6.0 Hz, ArH), 9.44 (s, 1H, ArH) (Supplementary Figure 135); $^{13}$C NMR (100 MHz, $D_2O$): δ 62.3, 64.3 (d, $J$ = 4.7 Hz), 77.8, 85.1 (d, $J$ = 8.7 Hz), 99.3, 128.5, 133.9, 139.8, 142.4, 146.0, 165.7 (Supplementary Figure 136).

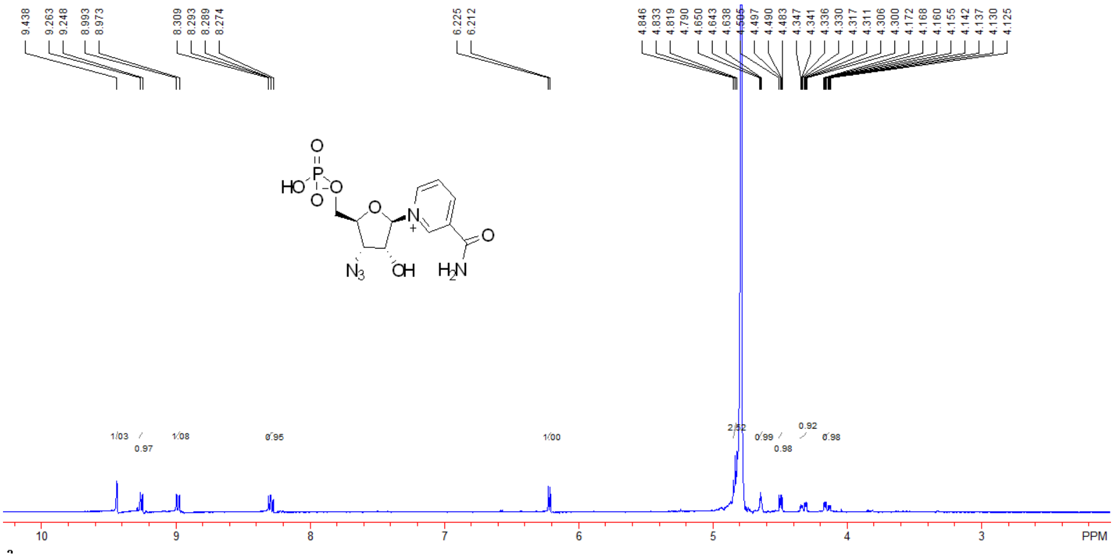


**Supplementary Figure 135**. $^{1}$H NMR spectrum of compound NMN6.

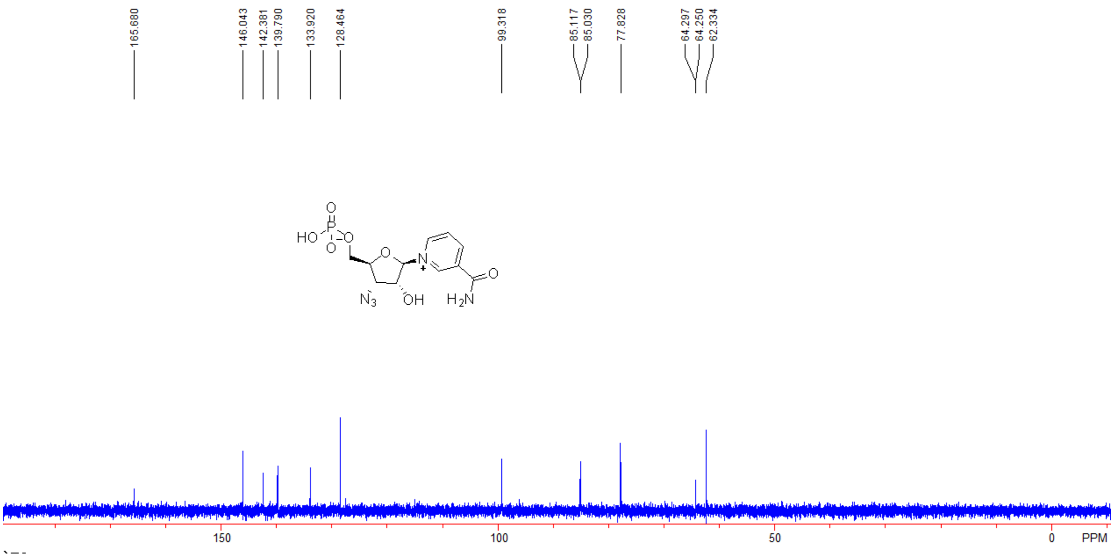


**Supplementary Figure 136**. $^{13}$C NMR spectrum of compound NMN6.

To a stirred solution of adenosine 5′-monophosphate (5′-AMP) (52 mg, 0.15 mmol, 1.5 eq) in dried DMF (2 mL) were added 1,1-carbonyldiimidazole (CDI) (63 mg, 0.50 mmol, 5 eq) and triethylamine (23 µL, 0.16 mmol. 1.6 eq). The reaction mixture was stirred at room temperature for 8 hours, and then quenched with 0.20 ml dried methanol. The solvent was then removed under vacuum and the residue was coevaporated 3 times each with 1.00 ml of dried DMF. The activated 5′-AMP was dissolved in dried DMF (1 mL) and compound NMN6 (36 mg, 0.10 mmol) was added. After stirring at room temperature for 4 days, $H_2O$ (5 mL) was added to quench the reaction at 0°C. The resulting mixture was continued stirring at room temperature for 24 hours. The reaction was then concentrated *in vacuo* and the crude product was purified

via preparative HPLC (C18-A column, 150×10.0 mm, 5 μm) (mobile phase A: 0.1% formic acid (aq), mobile B: 0.1% formic acid in acetonitrile; flow rate = 2.0 ml min$^{-1}$; 0-2 min: 0-4% B, 2-4 min: 4-10% B, 4-6 min: 10-20% B, 6-12 min: 20-50%, 12-14 min: 50-0% B). Fractions containing the desired product were concentrated and lyophilized to yield the NAD$^{+}$ analogue **6** (31 mg, 45% yield) as a colorless solid. $^{1}$H NMR (400 MHz, $D_2O$): δ 4.18-4.27 (m, 3H, $CH_2$+CH), 4.38-4.41 (m, 2H, $CH_2$), 4.49-453 (m, 2H, 2CH), 4.59 (br, 1H, CH), 4.72 (t, 1H, $J$ = 5.6 Hz, CH), 4.84 (t, 1H, $J$ = 5.6 Hz, CH), 6.12 (d, 1H, $J$ = 5.6 Hz, CH), 6.17 (d, 1H, $J$ = 5.6 Hz, CH), 8.27-8.30 (m, 1H, ArH), 8.39 (s, 1H, ArH), 8.58 (s, 1H, ArH), 8.94 (d, 1H, $J$ = 8.0 Hz, ArH), 9.25 (d, 1H, $J$ = 6.4 Hz, ArH), 9.40 (s, 1H, ArH) (Supplementary Figure 137); $^{13}$C NMR (100 MHz, $D_2O$): δ 62.3, 65.0-65.1 (m), 70.1, 74.5, 77.7, 84.0 (d, $J$ = 8.3 HZ), 84.9 (d, $J$ = 8.9 Hz), 87.7, 99.3, 118.3, 128.6, 133.8, 139.8, 142.2, 142.5, 145.0, 146.1, 148.2, 149.9, 165.4 (Supplementary Figure 138); HRMS (ESI) for $C_{21}H_{27}N_{10}O_{13}P_2^{+}$ (M)$^{+}$: Calcd.: 689.1234 Da; Obs: 689.1226 Da.

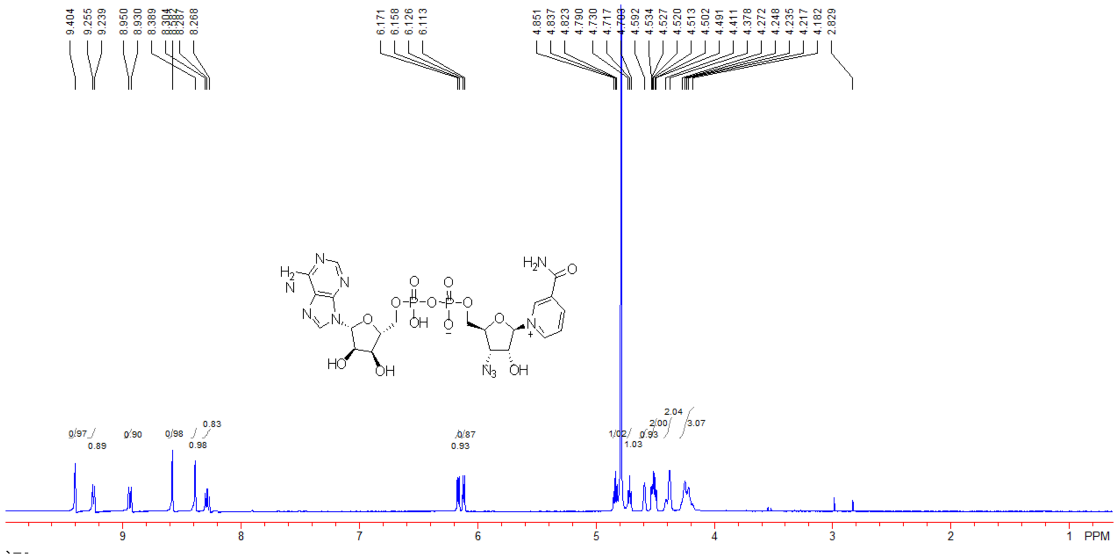


**Supplementary Figure 137**. $^{1}$H NMR spectrum of compound **6**.

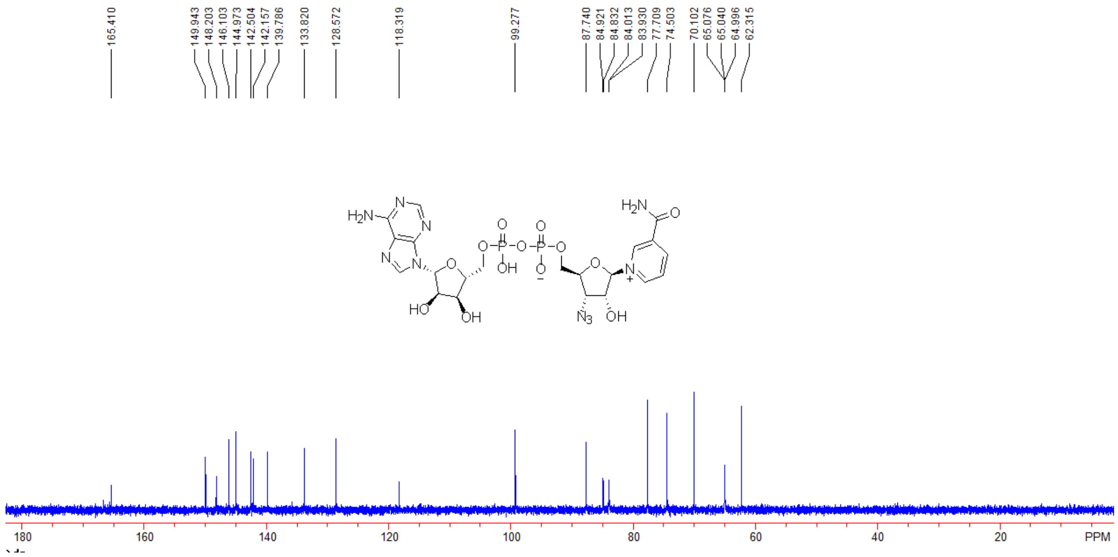

**Supplementary Figure 138**. $^{13}$C NMR spectrum of compound **6**.

**Synthesis of 6-alkyne-NAD$^+$ (6-a-NAD$^+$).**

6-a-NAD$^+$ is a known compound and prepared according to a procedure in the literature.[2] $^1$H NMR (400 MHz, $D_2O$): δ 2.75 (t, 1H, *J* = 2.4 Hz, CH), 4.18-4.25 (m, 4H, $CH_2$+CH), 4.36-4.39 (m, 3H, $CH_2$+CH), 4.44-4.46 (m, 1H, CH), 4.51-4.55 (m, 2H, 2CH), 4.58 (br, 1H, CH), 4.75-4.78 (m, 1H, CH), 6.14 (d, 2H, *J* = 5.6 Hz, 2CH), 8.24-8.28 (m, 1H, ArH), 8.40 (s, 1H, ArH), 8.59 (s, 1H, ArH), 8.91 (d, 1H, *J* = 8.0 Hz, ArH), 9.23 (d, 1H, *J* = 6.8 Hz, ArH), 9.39 (s, 1H, ArH) (Supplementary Figure 139).

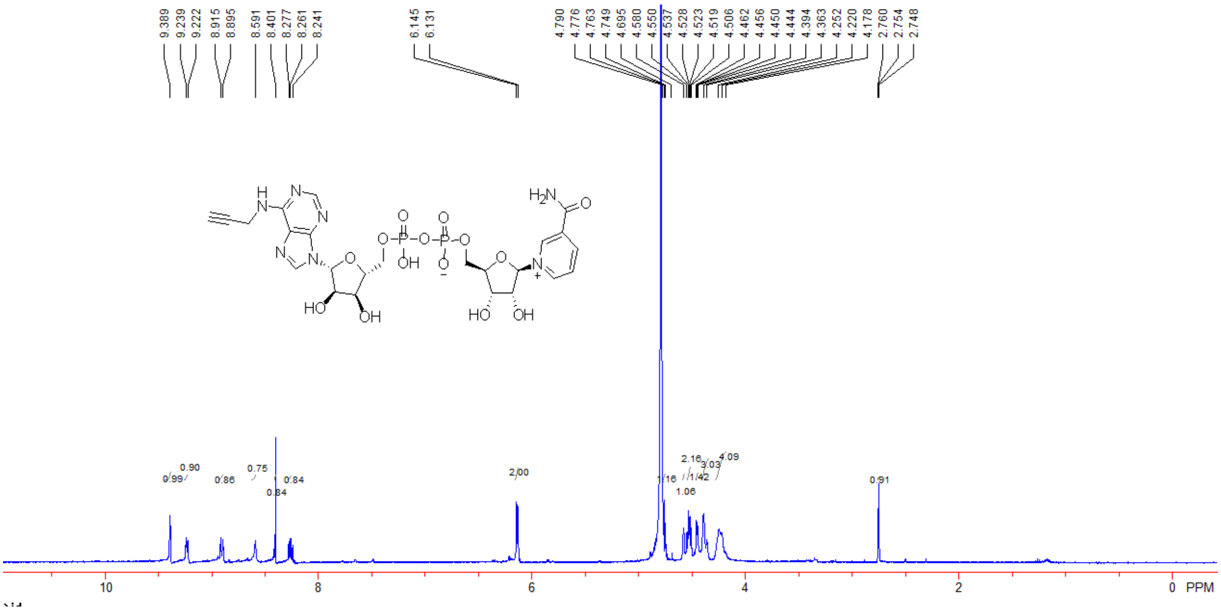

**Supplementary Figure 139**. $^1$H NMR spectrum of compound 6-a-NAD$^+$.

**Synthesis of 2-alkyne-NAD$^+$ (2-a-NAD$^+$).**

2-a-NAD$^+$ is a known compound and prepared according to a procedure in the literature.[3] $^1$H NMR (400 MHz, $D_2O$): δ 3.51 (s, 1H, CH), 4.18-4.28 (m, 4H, 2$CH_2$), 4.37-4.41 (m, 3H, 3CH), 4.46 (br, 1H, CH), 4.50 (t, 1H, $J$ = 4.4 Hz, CH), 4.78 (1H, CH, overlap with solvent residue peak), 5.94 (d, 1H, $J$ = 5.6 Hz, CH), 5.97 (d, 1H, $J$ = 4.8 Hz, CH), 8.20 (dd, 1H, $J$ = 8.0, 6.4 Hz, ArH), 8.47 (br, 2H, ArH), 8.87 (d, 1H, $J$ = 8.0 Hz, ArH), 9.04 (d, 1H, $J$ = 6.4 Hz, ArH), 9.29 (s, 1H, ArH) (Supplementary Figure 140).

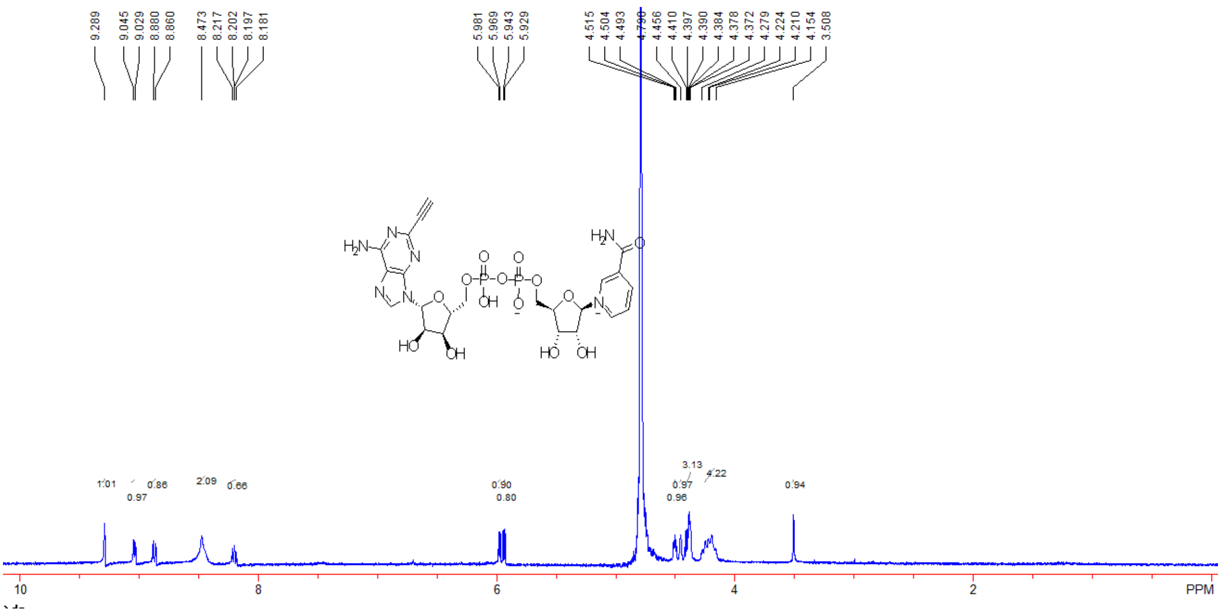

**Supplementary Figure 140**. $^1$H NMR spectrum of compound 2-a-NAD$^+$.

**Supplementary References**


1. Arya A*, et al.* Chemoenzymatic synthesis of 3'-deoxy-3'-(4-substituted-triazol-1-YL)-5-methyluridine. *Nucleosides, nucleotides & nucleic acids* **32**, 646-659 (2013).
2. Du J, Jiang H, Lin H. Investigating the ADP-ribosyltransferase activity of sirtuins with NAD analogues and 32P-NAD. *Biochemistry* **48**, 2878-2890 (2009).
3. Wang Y, Rösner D, Grzywa M, Marx A. Chain-Terminating and Clickable NAD+ Analogues for Labeling the Target Proteins of ADP-Ribosyltransferases. *Angewandte Chemie International Edition* **53**, 8159-8162 (2014).
