# Supplementary material for: A ribose-functionalized NAD+ with unexpected high activity and selectivity for protein poly-ADP-ribosylation
Source: Nat Commun. 2019 Sep 13;10:4196. doi: 10.1038/s41467-019-12215-4 (PMC6744458; doi:10.1038/s41467-019-12215-4)
Supplement: Supplementary file 3 — Reporting Summary [file 41467_2019_12215_MOESM3_ESM.pdf]

nature research

Corresponding author(s): Yong Zhang, Ph.D.

Last updated by author(s): Aug 16, 2019

Reporting Summary

Nature Research wishes to improve the reproducibility of the work that we publish. This form provides structure for consistency and transparency in reporting. For further information on Nature Research policies, see Authors & Referees and the Editorial Policy Checklist.

Statistics

For all statistical analyses, confirm that the following items are present in the figure legend, table legend, main text, or Methods section.

n/a	Confirmed	
☐	☒	The exact sample size (*n*) for each experimental group/condition, given as a discrete number and unit of measurement
☐	☒	A statement on whether measurements were taken from distinct samples or whether the same sample was measured repeatedly
☐	☒	The statistical test(s) used AND whether they are one- or two-sided<br>*Only common tests should be described solely by name; describe more complex techniques in the Methods section.*
☒	☐	A description of all covariates tested
☒	☐	A description of any assumptions or corrections, such as tests of normality and adjustment for multiple comparisons
☐	☒	A full description of the statistical parameters including central tendency (e.g. means) or other basic estimates (e.g. regression coefficient) AND variation (e.g. standard deviation) or associated estimates of uncertainty (e.g. confidence intervals)
☐	☒	For null hypothesis testing, the test statistic (e.g. *F*, *t*, *r*) with confidence intervals, effect sizes, degrees of freedom and *P* value noted<br>*Give P values as exact values whenever suitable.*
☒	☐	For Bayesian analysis, information on the choice of priors and Markov chain Monte Carlo settings
☒	☐	For hierarchical and complex designs, identification of the appropriate level for tests and full reporting of outcomes
☒	☐	Estimates of effect sizes (e.g. Cohen's *d*, Pearson's *r*), indicating how they were calculated

Our web collection on statistics for biologists contains articles on many of the points above.

Software and code

Policy information about availability of computer code

Data collection	HPLC data were collected by the software of MassLynx V4.0. Immunoblot data were collected using a ChemiDoc Touch Imaging System (Bio-Rad Laboratories, Inc, Hercules, CA). Raw data from devices (NMR and cell imaging) were collected as described in the Methods section.
Data analysis	NMR data analysis was performed using NUTS 2005 and MestreNova. Immunoblot data were analyzed by Image Lab software (Bio-Rad Laboratories, Inc, Hercules, CA) and quantified using ImageJ (https://imagej.nih.gov/ij/index.html). LAS X software (Leica Microsystems Inc., Buffalo Grove, IL) was used to process confocal images from Leica SP8 confocal laser scanning microscope. Statistical analyses were performed using GraphPad Prism (GraphPad Software, La Jolla, CA).

For manuscripts utilizing custom algorithms or software that are central to the research but not yet described in published literature, software must be made available to editors/reviewers. We strongly encourage code deposition in a community repository (e.g. GitHub). See the Nature Research guidelines for submitting code & software for further information.

Data

Policy information about availability of data

All manuscripts must include a data availability statement. This statement should provide the following information, where applicable:

- Accession codes, unique identifiers, or web links for publicly available datasets
- A list of figures that have associated raw data
- A description of any restrictions on data availability

The data that support the findings of this study are available from the corresponding author upon request.

Field-specific reporting

Please select the one below that is the best fit for your research. If you are not sure, read the appropriate sections before making your selection.

☒ Life sciences ☐ Behavioural & social sciences ☐ Ecological, evolutionary & environmental sciences

For a reference copy of the document with all sections, see nature.com/documents/nr-reporting-summary-flat.pdf

Life sciences study design

All studies must disclose on these points even when the disclosure is negative.

Sample size	For in vitro experiments, at least 3 independent experiments were conducted, typically with at least 3 or 4 biological replicates. For the confocal imaging analysis, 10 high power fields were randomly selected from each slide of every independent experiment.
Data exclusions	No results were excluded for any studies described in this work.
Replication	Experimental findings reported in this manuscript were reliably reproduced.
Randomization	In the confocal imaging analysis, high power fields were randomly selected from the slices
Blinding	All samples were processed in the same manner regardless of their origin.

Reporting for specific materials, systems and methods

We require information from authors about some types of materials, experimental systems and methods used in many studies. Here, indicate whether each material, system or method listed is relevant to your study. If you are not sure if a list item applies to your research, read the appropriate section before selecting a response.

Materials & experimental systems

n/a	Involved in the study	
☐	☒	Antibodies
☐	☒	Eukaryotic cell lines
☒	☐	Palaeontology
☒	☐	Animals and other organisms
☒	☐	Human research participants
☒	☐	Clinical data

Methods

n/a	Involved in the study	
☒	☐	ChIP-seq
☒	☐	Flow cytometry
☒	☐	MRI-based neuroimaging

Antibodies

Antibodies used	Anti-PAR monoclonal antibody (clone: 10H, from Santa Cruz Biotechnology sc-56198); Streptavidin-HRP (from R&D Systerm, Catalog No. DY998); Anti-His6 antibody (clone: HIS.H8, from Thermo Fisher Scientific MA1-21315); Anti-GAPDH antibody (clone: GA1R, from Thermo Fisher Scientific MA5-15738); Fc-WWE antibody (EMD Millipore: MABE1031); anti-mouse antibody-HRP conjugate (Thermo Fisher Scientific: G-21040); anti-rabbit antibody-HRP conjugate (Thermo Fisher Scientific: G-21234); anti-COX IV (clone: 3E11, from Cell Signaling Technology #4844); anti-Histone 2A.Z (polyclonal, from Cell Signaling Technology product #2718S); anti-PARP1 antibody (clone: 46D11, from Cell Signaling Technology product #2718S); anti-PARP2 antibody (clone: F-3, from Santa Cruz Biotechnology sc-393310).
Validation	All antibodies used in this study were provided validation statements for the species and application or relevant citations on the manufactures' websites.

Eukaryotic cell lines

Policy information about cell lines

Cell line source(s)	Human cervical cancer cell HeLa was obtained from the American Type Culture Collection (ATCC) (Manassas, VA).
Authentication	We have authenticated this cell line based on the morphology under microscope.
Mycoplasma contamination	HeLa cells were tested negative for mycoplasma by Charles River (Wilmington, MA).
Commonly misidentified lines (See ICLAC register)	No commonly misidentified cell lines were used.
